# Construction of Molecular Subtype and Prognosis Prediction Model of Osteosarcoma Based on Aging-Related Genes

**DOI:** 10.1155/2022/8177948

**Published:** 2022-09-16

**Authors:** Chunli Dong, Yindi Sun, Ying Zhang, Bianni Qin, Tao Lei

**Affiliations:** ^1^Department of Anesthesiology and Operation, The Second Affiliated Hospital of Xi'an Jiaotong University, Xi'an, China; ^2^Pain Ward of Orthopedics Department of TCM, Honghui Hospital, Xi'an Jiaotong University, Xi'an, China

## Abstract

**Background:**

Osteosarcoma (OS) is a rare form of malignant bone cancer that is usually detected in young adults and adolescents. This disease shows a poor prognosis owing to its metastatic status and resistance to chemotherapy. Hence, it is necessary to design a risk model that can successfully forecast the OS prognosis in patients.

**Methods:**

The researchers retrieved the RNA sequencing data and follow-up clinical data related to OS patients from the TARGET and GEO databases, respectively. The coxph function in R software was used for carrying out the Univariate Cox regression analysis for deriving the aging-based genes related sto the OS prognosis. The researchers conducted consistency clustering using the ConcensusClusterPlus R package. The R software package ESTIMATE, MCPcounter, and GSVA packages were used for assessing the immune scores of various subtypes using the ssGSEA technique, respectively. The Univariate Cox and Lasso regression analyses were used for screening and developing a risk model. The ROC curves were constructed, using the pROC package. The performance of their developed risk model and designed survival curve was conducted, with the help of the Survminer package.

**Results:**

The OS patients were classified into 2 categories, as per the aging-related genes. The results revealed that the Cluster 1 patients showed a better prognosis than the Cluster 2 patients. Both clusters showed different immune microenvironments. Additional screening of the prognosis-associated genes revealed the presence of 5 genes, i.e., ERCC4, GPX4, EPS8, TERT, and STAT5A, and these data were used for developing the risk model. This risk model categorized the training set samples into the high- and low-risk groups. The patients classified into the high-risk group showed a poor OS prognosis compared to the low-risk patients. The researchers verified the reliability and robustness of the designed 5-gene signature using the internal and external datasets. This risk model was able to effectively predict the prognosis even in the samples having differing clinical features. Compared with other models, the 5- gene model performs better in predicting the risk of osteosarcoma.

**Conclusion:**

The 5-gene signature developed by the researchers in this study could be effectively used for forecasting the OS prognosis in patients.

## 1. Introduction

An osteosarcoma is a malignant form of tumor affecting the bones. It originates in the mesenchymal tissue and is expressed in the proximal tibia and distal femur tissues. OS shows a unique characteristic feature, wherein the tumor cells tend to directly form bone-like tissue or immature bones [1]. Osteosarcoma is mostly noted in adolescents and people below the age of 20 years, with a high degree of malignancy and easy pulmonary metastasis [2]. Studies have found that there is a close correlation between the rapid growth of adolescent bones and the onset and progression of osteosarcoma [3]. With the development of clinical diagnosis technology and surgical treatment technology, the nonmetastatic OS patients showed a better 5-year overall survival rate of 60–70% [4]; however, the patients with recurring or metastatic osteosarcoma showed a 5-year overall survival rate of only 20% [5]. Therefore, researchers need to determine the important regulatory targets for the occurrence and metastasis of osteosarcoma and develop new prognostic markers for osteosarcoma patients.

Cell aging is a generally stable state in which cells stop going through the cell cycle as a result of changes in the external microenvironment or internal gene expression and inactivation and lose their capacity to multiply indefinitely [6]. In the past, researchers have noted that cellular aging was related to tumorigenesis, tumor development, and escape therapy. In the early stage of tumorigenesis, the inflammatory reaction is conducive to the elimination of aging and mutant cells and the prevention and inhibition of tumorigenesis [7]. However, during the later stages of tumor development, there is a change in the inflammatory microenvironment, which consists primarily of the growth factors and inflammatory molecules that are secreted by aging cells, which can induce the epithelial-mesenchymal conversion of the tumor cells and promote migration, proliferation, invasion, and the metastasis of tumor cells [8]. Hence, the researchers need to study the potential role of aging in tumorigenesis and development.

In this study, the researchers acquired the RNA-Seq data of 85 osteosarcoma patients using the TARGET database and classified the data into 2 groups based on the aging-related genes. Depending on the genes significantly related to prognosis, they further constructed a 5-gene risk model that included genes like TERT, GPX4, ERCC4, EPS8, and STAT5A. This 5-gene risk model is effective in forecasting the OS prognosis of the patients.

## 2. Materials and Methods

### 2.1. Analysis Process

**Figure 1:**
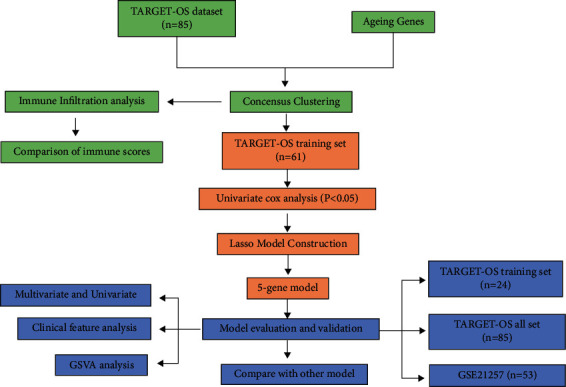
Analysis of the flow chart.

Figure 1 presents the analytical flow chart used in this paper.

### 2.2. Source and Pretreatment of Data

The researchers retrieved the RNA-Seq data for the osteosarcoma (OS) patients along with their clinical follow-up data from the TARGET database. They also downloaded the Gene Expression Omnibus (GEO) data from the GEO database and selected the GSE21257 chip data in addition to the lifespan of the OS patients. This data set included the expression data of 53 tissue samples.

The RNA-Seq data of 85 TARGET-OS cases were processed as follows: (1) eliminating all samples that did not contain the clinical follow-up data, (2) discarding the samples that did not present the overall survival data, and (3) eliminating the samples that did not reflect the patients' status.

The researchers processed the data sets for the 53 GEO patients as follows: (1) eliminating samples that did not include the clinical follow-up data, (2) discarding all samples that did not present the overall survival, and (3) eliminating the samples that did not reflect the patients' status. After the pretreatment of the two groups of data, the TARGET-OS included 85 samples, comprising 302 genes (Supplement Table 1), while the GSE21257 consisted of 53 samples that were included in the external, independent verification dataset. Table 1 describes the clinical information for the population sample.

### 2.3. Molecular Typing of the Genes Based on the Aging-Related Genes

Firstly, the researchers extracted the gene expression profiles of the 302 aging-linked genes from the TARGET database. Then, they used the coxph function in R software for carrying out the Univariate Cox regression analysis for deriving the genes that were linked to the disease prognosis in the OS patients. Then, the TARGET-OS samples were clustered using the ConcensusClusterPlus R software package (distance parameter was Euclidean, ClusterAlg parameter was km), and heat maps were drawn based on the prognostic genes. The survival curve of osteosarcoma was drawn based on OS data.

For determining the correlation between different molecular subtypes and immune scores, the researchers used the ESTIMATE function in the R software GSVA package for assessing the 3 immune scores, i.e., ImmuneScore, StromalScore, and ESTIMATEScore. They used the MCPcounter for determining the scores of 10 different immune cells. Then, they implemented the ssGSEA technique using the GSVA package for scoring 28 immune cells [9]. Lastly, they compared all variations occurring in the immune scores for the numerous molecular subtypes.

### 2.4. Constructing a Prognostic Risk Model as per the Aging-Related Genes

The researchers retrieved the expression profiles of the aging-linked genes that could affect the OS prognosis, from the TARGET database. All the 85 TARGET samples were categorized into the training and validation sets, in the 7 : 3 ratio. For improving clinical detection, the researchers also used the Lasso regression and the Akaike Information Criterion (AIC) for reducing the number of genes that could be included in the model. The researchers noted that the Lasso regression [10] offered a better and refined model as it helped in building a penalty function that allowed the researchers to compress a few coefficients and set the value of particular coefficients to 0. As a result, it benefitted from subset contraction. It was seen to be a biased estimator that could handle complex data collinearity. It may effectively implement variable selection while calculating the parameters and could address the multicollinearity issue during regression analysis. To fit the number of parameters, the researchers have used the AIC.

The MASS package's stepAIC technique begins with the most difficult model and gradually eliminates variables to lower AIC. The model showed a better performance when the AIC value was lower. It demonstrates that this model showed a satisfactory degree of fitting with lesser parameters. Finally, the researchers used the survival analysis and ROC curves in the training set for assessing the model performance.

### 2.5. Verification of the Risk Model

The researchers tested the risk model with the help of varying data sets, as follows: (1) the ROC curve was constructed using the pROC package to assess the prognosis model's performance, and (2) the survival curve was generated using the Survminer program to assess the prognosis model's capacity to differentiate between the high- and low-risk patients.

### 2.6. Relationship between RiskScore and Pathway

The researchers used the R software GSVA package for determining the correlation between the RiskScore values of various samples and their bioactivities. For this purpose, they carried out the ssGSEA analysis for determining the ssGSEA scores of various biological functions for every sample. Then, they determined the correlation between all biological functions and the risk scores and selected the functions showing a correlation >0.35.

### 2.7. RiskScores and the Clinical Features for Constructing the Forest Map

The statistical results of several study components can be easily and intuitively displayed on the forest map. A valid line perpendicular to the *X*-axis (often at coordinates *x* = 1 or 0) is taken as the center in the standard form of a forest map, like that in a plane rectangular coordinate system. The magnitude of the effect and its 95% confidence interval are shown for each study as a number of line segments parallel to the *X*-axis. RiskScore and the associated clinical factors were assessed by Univariate and Multivariate Cox Regression analyses and displayed by forest map to establish the model's independence in clinical applications and for integrating the clinical information.

### 2.8. Statistical Analyses and Testing of the Proposed Hypotheses

The statistical analysis technique in R 3.6 provides a foundation for all statistical comparisons used in this study, as well as for testing the hypothesis that the groups displayed a statistically significant difference.

## 3. Results

### 3.1. Molecular Typing Based on Aging-Related Genes

**Figure 2:**
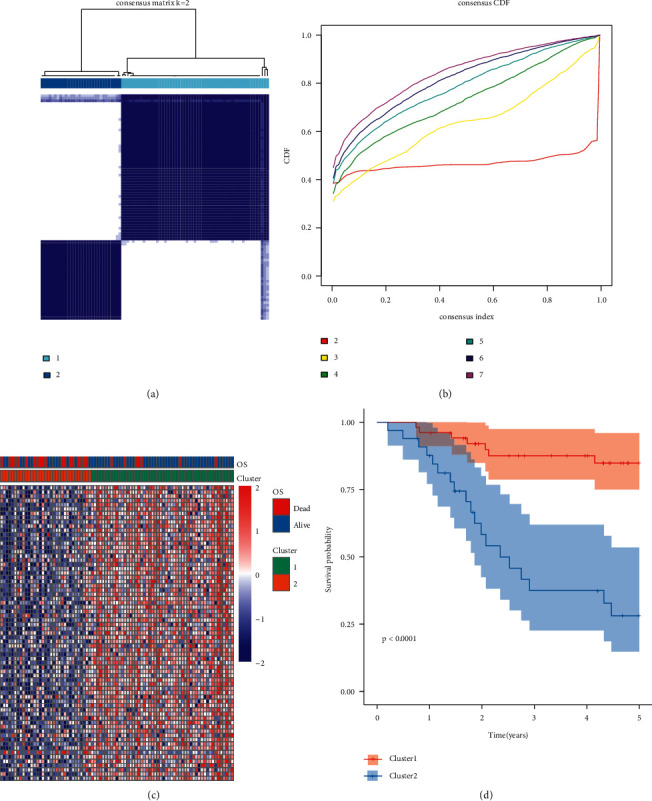
(a) Consistency matrix heat map if *k* = 2. (b) Cumulative distribution of the Cluster consistency. (c) Cluster heat map of the prognosis-linked genes. (d) KM plots for the OS of the subgroup patients retrieved from target.

The researchers used the Univariate Cox analysis on TARGET expression profile data encompassing 302 aging genes and identified 91 prognosis-related genes (Supplement Table 2). Consensus Clustering analysis was carried out using the gene expression profile linked to prognosis. The ideal number of clusters is two, as can be seen from the CDF diagram (Figure 2(b)). At the same time, it is clear from the consistency matrix's heat map that the sample's clustering performance when *k* = 2 is quite favorable (Figure 2(a)). Heat maps were used to display the clustering results. When compared to Cluster 2, Cluster 1 showed significantly higher expression of most of the prognosis-related genes. Additionally, Figure 2(c) showed that the samples for the patients who had expired were concentrated in the Cluster 2 subtype. The researchers plotted the survival curves for the two molecular subtypes. The findings demonstrated that Cluster 1 patients had a statistically and significantly better prognosis than Cluster 2 patients (Figure 2(d), *P* 0.001).

### 3.2. Comparative Analysis of the Immune Scores and the Matrix Scores between Both the Molecular Subtypes

**Figure 3:**
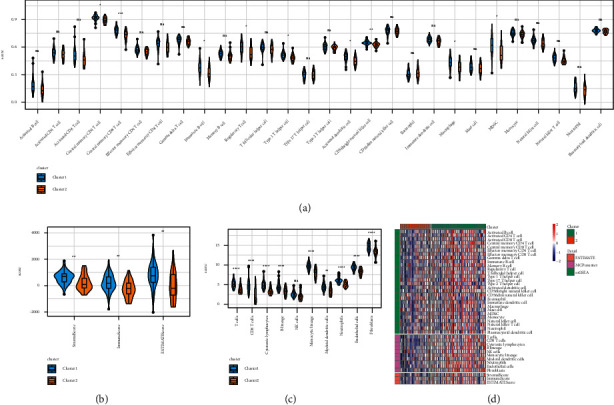
(a) Comparison of ssGSEA immune scores between different molecular subtypes. (b) Comparison of the calculated immune scores between different molecular subtypes. (c) Comparison of the MCPcounter immune scores noted between various molecular subtypes. (d) Heat map comparison of the immune scores using 3 immune software packages between different molecular subtypes.

The analysis outcomes for three R packets are displayed in the violin diagram. According to the ssGSEA results, the immune scores in Cluster 1 are statistically higher compared to Cluster 2, in terms of central memory CD8 T cells, activated B cells, central memory CD4 T cells, regulatory T cells, Type 1 T helper cells, activated dendritic cells, macrophages, CD56 bright natural killer cells, and MDSC (Figure 3(a)). The Estimate results indicated that the immune scores in Cluster 1 derived from the StromalScore and ESTIMATEScore were seen to be significantly higher compared to those displayed in Cluster 2 (Figure 3(b)). According to MCPcounter data, Cluster 1 had an immunological score that was statistically greater than Cluster 2 in terms of T cells, monocytic lineage, B lineage, CD8 T cells, cytotoxic lymphocytes, neutrophils, myeloid dendritic cells, endothelial cells, and fibroblasts (Figure 3(c)).  Figure 3(d) displays the heat maps for the three immunological scores.

### 3.3. Designing a Prognostic Risk Model That Was Based on the Aging-Related Genes

#### 3.3.1. Randomly Grouping the Samples

Keep the aging-related gene expression profiles from the TARGET dataset that affects prognosis. The 85 TARGET samples were separated into the training and validation sets, in a 7 : 3 ratio, and all samples were assessed using the Chi-square test of clinically relevant indicators. The training and the validation sets did not display any significant differences in the values of variables such as OS, age, or gender. The results are shown in Table 2.

#### 3.3.2. Training Set Univariate Cox and Multivariate Cox Risk Analysis

**Figure 4:**
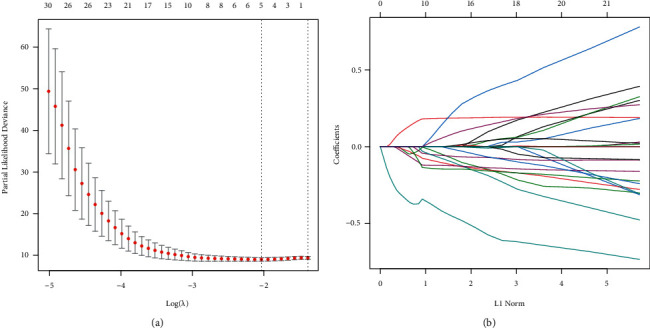
(a) Change track of every independent variable, where the *X*-axis denotes the log value of an independent variable (lambda), while the *Y*-axis denotes the coefficient of an independent variable. (b) The confidence interval included under every lambda.

**Figure 5:**
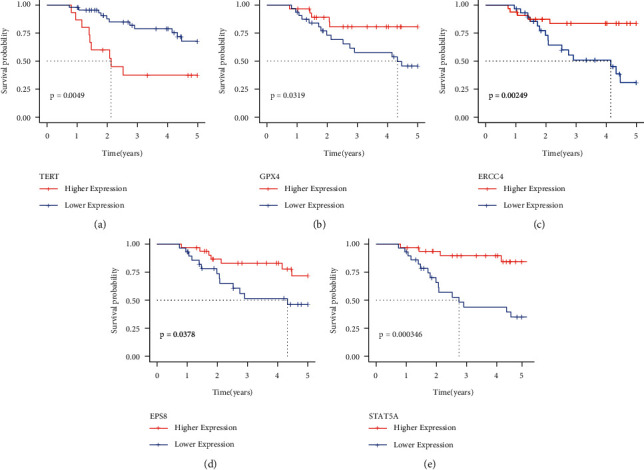
KM curves of 5 genes derived from the TARGET training set.

**Figure 6:**
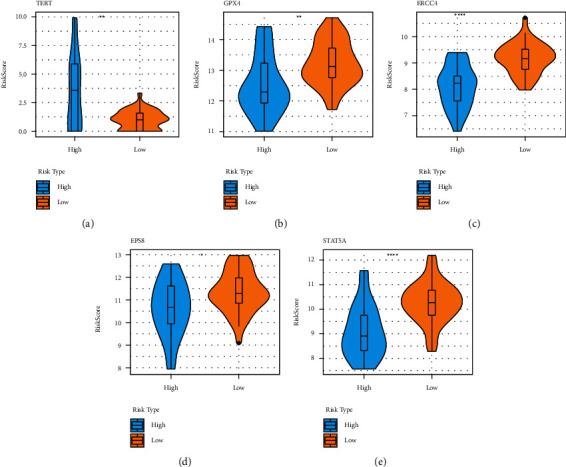
Expression levels of the above-mentioned 5 genes that were categorized into the high- and low-risk groups.

Each aging-related gene and the survival data were examined using Univariate Cox analysis on the training set data. 34 genes with a significant difference were obtained using the R package survival coxph function, with *p* < 0.05 set as a filtering criterion (Supplement Table 3). It is vital to reduce the range of aging-linked genes while keeping a high level of accuracy since the vast number of genes makes clinical detection difficult. These 34 genes were analyzed using the Lasso Cox regression analysis and the Akaike Information Criteria (AIC) using the R software glmnet package to further minimize the no. of genes in this risk model.  Figure 4(a) displays the changing track for every independent variable. It is clear that if the lambda value steadily increased, there is a similar increase in the no. of independent variable coefficients tending toward 0. Tenfold cross-validation is used to construct the model.  Figure 4(b) illustrates the analysis of the confidence intervals under every lambda. The model performs best when lambda = 0.1306079, as shown in the figure. Five genes are chosen as the target genes for additional investigation when lambda = 0.1306079. These five genes are STAT5A, GPX4, ERCC4, EPS8, and TERT.  Figure 5 displays the prognostic KM curve for the five genes. These five genes were used for significantly dividing the TARGET training set samples between the high and low-risk groups (*p* < 0.05). TERT is strongly expressed in high-risk groups, while GPX4, ERCC4, EPS8, STAT5A, and other important genes are expressed at low levels in high-risk groups, depending on the comparison of expression levels of these genes in these groups (Figure 6).

#### 3.3.3. Construction and Evaluation of the Risk Model

The following risk model scoring formula was used for the above-mentioned five genes:

(1) $$\begin{array}{c} \text{RiskScore}=\text{TERT}\times 0.12-GPX4\times 0.03-ERCC4\times 0.26-EPS8\times 0.019-\text{STAT}5A\times 0.1. \end{array}$$

**Figure 7:**
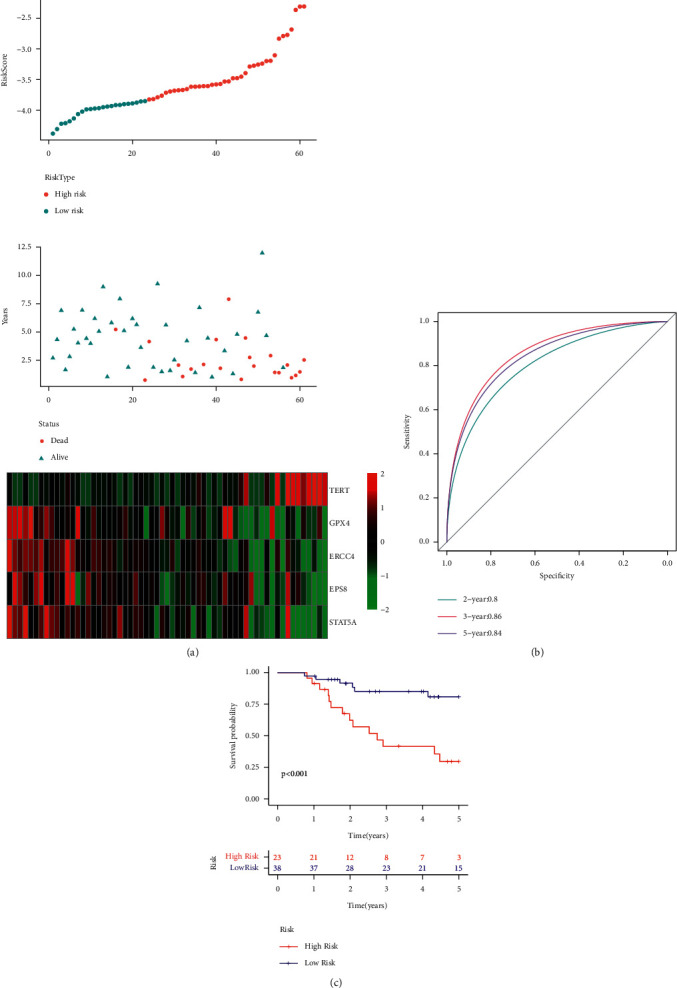
(a) RiskScore, survival status, survival time, and the expression of 5 genes retrieved from the TARGET training set. (b) ROC curve and the AUC of the novel 5-gene signature. (c) Distribution of the KM survival curves for the 5-gene signature included in the TARGET training set.

Here, the researchers calculated the RiskScore for every sample depending on the expression of 5 genes and then plotted the RiskScore distribution of all samples as described in Figure 7(a). The results presented in the figure showed that the OS of samples with a high RiskScore is smaller compared to the OS of samples with a low RiskScore, implying that the samples having a high RiskScore display a bad prognosis. The researchers further analyzed the expression variations of the 5 genes, based on their increase in the risk values, and noted that the high TERT expression was related to high risk, which was a risk factor. Additionally, ROC analysis on the RiskScore prognostic classification was carried out using the R software package of time ROC.  Figure 7(b) illustrates the respective classification effectiveness of 2-, 3-, and 5-year prognosis prediction. It is clear from this figure that the new risk model has a significant Area Under the Curve (i.e., AUC) value. The Risk score was then treated using the z-score, and the samples with z-score values > 0 were categorized into the high-risk group, while samples with z-score values < 0 were categorized into the low-risk group. Thus, 38 samples were placed in the low-risk group, while 23 samples were placed in the high-risk group. The KM curves (Figure 7(c)) indicated that both the risk groups displayed a significantly different prognosis (*p* < 0.001).

### 3.4. Verification of the Risk Model

#### 3.4.1. Internal Data Sets to Verify the Robustness of This 5-Gene Signature

**Figure 8:**
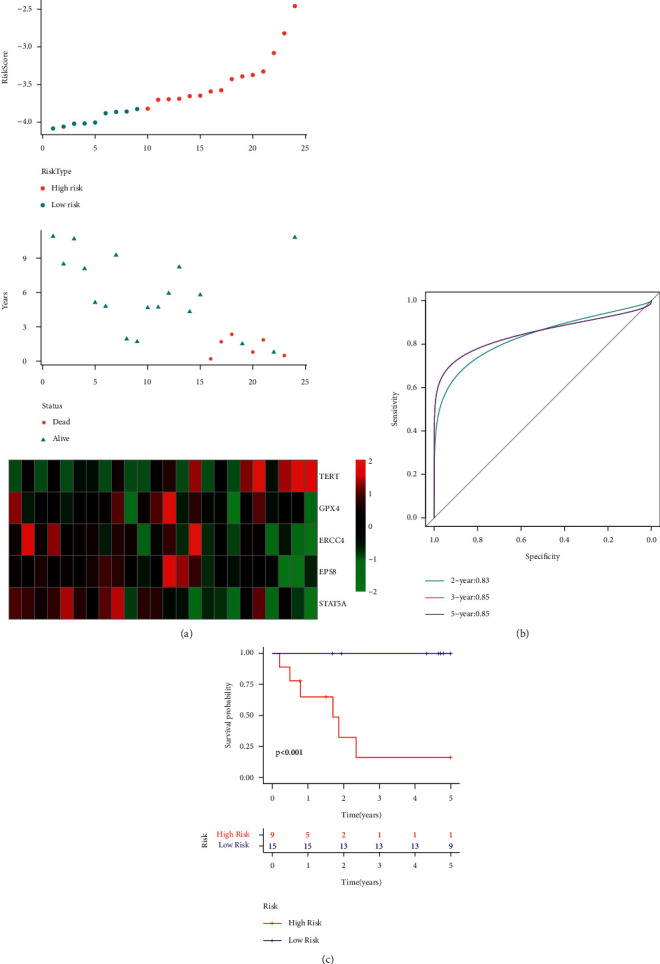
(a) RiskScore, survival status, survival time, and the expression of 5 genes retrieved from the TARGET test set. (b) ROC curve and AUC of the novel 5-gene signature. (c) Distribution of KM survival curves for the 5-gene signature included in the TARGET test set.

The researchers utilized the same model and the coefficients as the training dataset in the TARGET validation set and for all data sets to estimate the reliability of the constructed risk model. They determined the RiskScore for each sample and plotted the sample's RiskScore distribution.  Figure 8(a) depicts the distribution of RiskScores for the TARGET verification set. The results presented in the figure indicate that the OS of samples with a high RiskScore is shorter compared to that displayed by the samples with a low RiskScore, indicating that the samples having a high RiskScore show a worse prognosis. The researchers further analyzed the expression variations of the 5 genes, based on their increase in the risk values, and noted that the high TERT expression was related to high risk, which was a risk factor. On the other hand, the higher expression of ERCC4, GPX4, EPS8, and STAT5A was seen to be associated with low risk, which acted as a protective factor. These results were similar to those displayed by the samples in the TARGET training set. Additionally, ROC analysis on the RiskScore prognostic categorization was carried out using the R software package time ROC.  Figure 8(b) illustrates the respective classification effectiveness of 2-, 3-, and 5-year prognosis prediction. The RiskScore was then treated using the z-score, and samples having z-score values > 0 were categorized into the high-risk group, while samples with z-score values < 0 were categorized into the low-risk group. Thus, 15 samples were placed in the low-risk group, while 9 samples were placed in the high-risk group. The KM curves (Figure 8(c)) indicated that both the risk groups showed a significantly different prognosis (*p* < 0.001).

**Figure 9:**
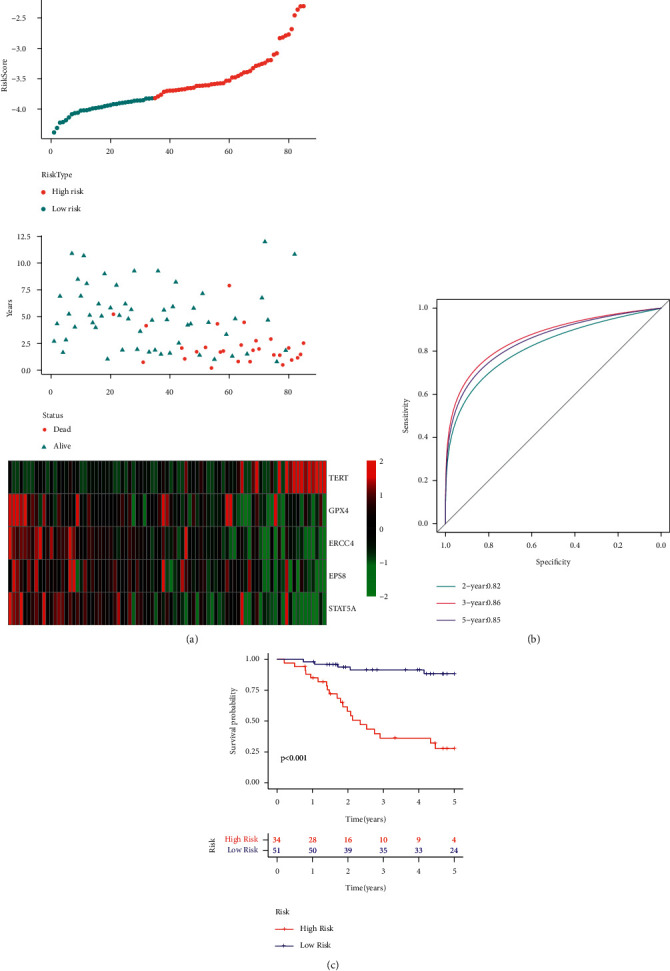
(a) RiskScore, survival status, survival time, and expression of 5 genes retrieved from all TARGET datasets. (b) ROC curve and AUC of the novel 5-gene signature. (c) Distribution of KM survival curves for the 5-gene signature included in the TARGET datasets.

Figure 9(a) presents the RiskScore distribution of all samples in the TARGET datasets. The results presented in the figure indicated that the OS of the samples with high RiskScore is shorter compared to that displayed by the samples with a low RiskScore, indicating that the samples having a high RiskScore show a worse prognosis. The researchers further analyzed the expression variations of the 5 genes, based on their increase in the risk values, and noted that the high TERT expression was related to high risk, which was a risk factor. On the other hand, the higher expression of ERCC4, GPX4, EPS8, and STAT5A was seen to be associated with low risk, which acted as a protective factor. These results were similar to those displayed by the samples in the TARGET training set. Additionally, ROC analysis on the RiskScore prognostic classification was carried out using the R software package time ROC.  Figure 9(b) illustrates the respective classification effectiveness of 2-, 3-, and 5-year prognosis prediction. The results indicated that the risk model showed a higher AUC value. The RiskScore was then treated using the z-score, and samples with z-score values > 0 were categorized into the high-risk group, while samples with z-score values < 0 were categorized into the low-risk group. Thus, 51 samples were placed in the low-risk group, while 34 samples were placed in the high-risk group. The KM curves (Figure 9(c)) indicated that both the risk groups showed a significantly different prognosis (*p* < 0.001).

#### 3.4.2. External Data Sets to Verify the Reliability and Robust Nature of the 5-Gene Signature

**Figure 10:**
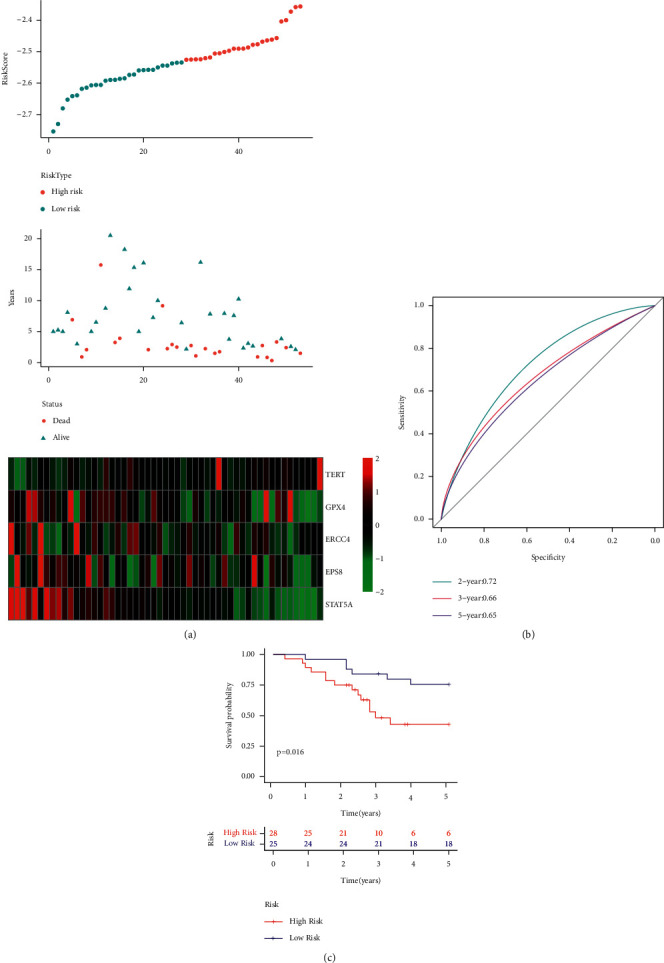
(a) RiskScore, survival status, survival time, and expression of 5 genes retrieved from the independent validation data set GSE21257. (b) ROC curve and the novel AUC of the 5-gene signature. (c) Distribution of the KM survival curves for the 5-gene signature included in the independent validation data set GSE21257.

For analyzing the external independent verification dataset, i.e., GSE21257, the researchers used the same newly constructed risk model and coefficients as used in the training set for estimating the RiskScore values of every sample. They plotted the RiskScore distribution of these samples in Figure 10(a). The results presented in the figure indicated that the OS of the samples with high RiskScore is shorter compared to that displayed by the samples with a low RiskScore, indicating that the samples having a high RiskScore show a worse prognosis. The researchers further analyzed the expression variations of the 5 genes, based on their increase in the risk values, and noted that the high TERT expression was related to high risk, which was a risk factor. On the other hand, the higher expression of ERCC4, GPX4, EPS8, and STAT5A was seen to be associated with low risk, which acted as a protective factor. These results were similar to those displayed by the samples in the TARGET training set. Additionally, ROC analysis on the RiskScore prognostic classification was carried out using the R software package time ROC.  Figure 10(b) illustrates the respective classification effectiveness of 2-, 3-, and 5-year prognosis prediction. The results indicated that the risk model showed a higher AUC. The RiskScore was then treated using the z-score, and samples having z-score values > 0 were categorized into the high-risk group, while samples with z-score values < 0 were categorized into the low-risk group. Thus, 25 samples were placed in the low-risk group, while 28 samples were placed in the high-risk group. The KM curves (Figure 10(c)) indicated that both the risk groups showed a significantly different prognosis (*p* < 0.001).

### 3.5. Risk Model and Prognosis Analysis of the Clinical Characteristics Displayed by the Samples

**Figure 11:**
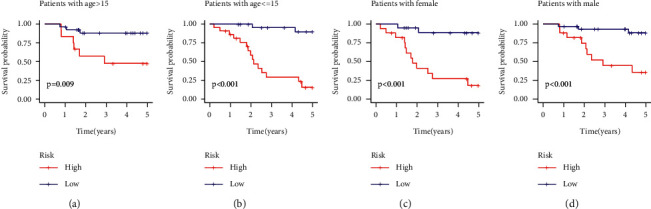
(a) KM curves for the high- and the low-risk groups that included patients more than 15 years of age. (b) KM curves for the high-risk and low-risk groups that included patients less than 15 years of age. (c) KM curves for the high- and low-risk groups that included female patients. (d) KM curves for the high- and low-risk groups that included male patients.

The researchers carried out the survival analysis of both the risk groups, based on their RiskScore values, in the samples that were categorized using different clinical features. The results showed that the novel 5-gene signature could significantly differentiate between the age and the gender of all patients categorized into the high-risk and low-risk groups, respectively (Figure 11, (p) < 0.01). The results also indicated that the developed risk model displayed a good predictive ability even if the samples displayed differential clinical characteristics.

### 3.6. Relationship between the RiskScores and Pathway

**Figure 12:**
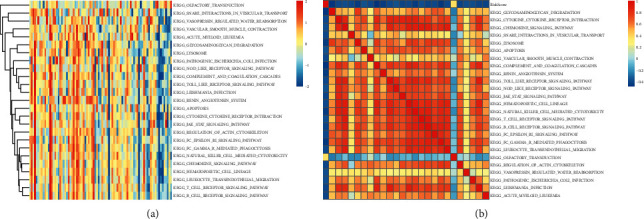
(a) Clustering of the correlation coefficients noted between the RiskScore and the KEGG pathway, where the RiskScore correlation was >0.35. (b) Variations in the ssGSEA score of the KEGG pathway having a correlation <0.35 in every sample with increasing RiskScore. The *X*-axis denotes the sample, where the RiskScore value increased from left to right.

While analyzing the correlation between the RiskScore values and the biological functions, the researchers noted that 24 KEGG pathways showed a negative correlation with the RiskScore value of the samples, whereas 1 KEGG pathway was positively correlated with the RiskScore value (Figure 12(a)). Then, they selected these KEGG pathways to carry out a Cluster analysis based on their different enrichment scores.  Figure 12(b) presents the results of this analysis, and it was noted that out of the 25 pathways, the KEGG_JAK_STAT_SIGNALING_PATHWAY, KEGG_NATURAL_KILLER_CELL_MEDIATED_CYTOTOXICITY, KEGG_NATURAL_KILLER_CELL_MEDIATED_CYTOTOXICITY, KEGG_TOLL_LIKE_RECEPTOR_SIGNALING_PATHWAY, KEGG_CYTOKINE_CYTOKINE_RECEPTOR_INTERACTION, and a few other pathways got suppressed when the RiskScore value increased.

### 3.7. Relationship between RiskScore Values and the Clinical Characteristics of the Patients

**Figure 13:**
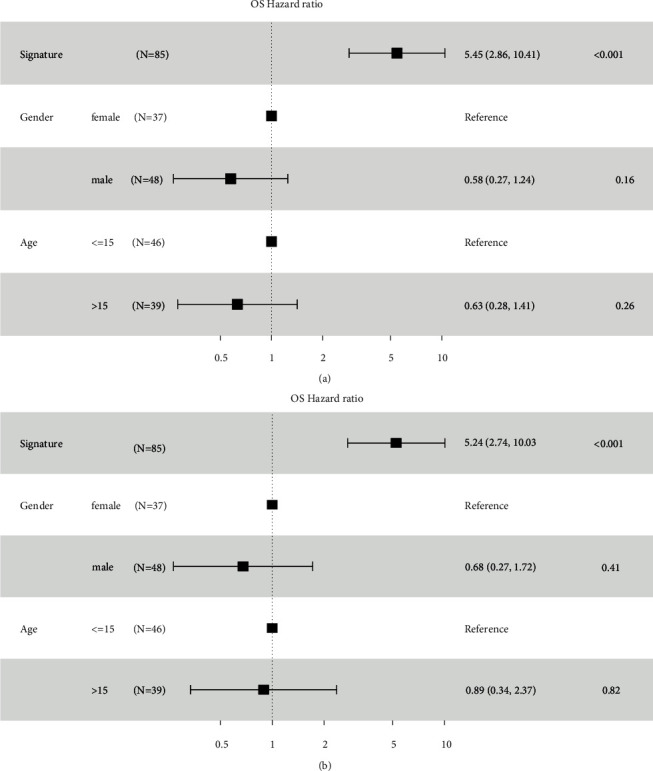
(a) Univariate analysis based on the newly constructed 5-gene signature, age, and gender of the patients. (b) Multivariate analysis based on the novel 5-gene signature, age, and gender of the patients included in the study.

For determining the robustness of the new 5-gene signature model during clinical applications, the researchers used the complete clinical data presented in the TARGET dataset for Univariate and Multivariate Cox Regression analyses. They displayed the results using a forest map. The forest map showed a RiskScore value of HR = 5.45, 95%CI = 2.86–10.41, *p* < 0.001 (Figure 13(a)) during Univariate analysis, while the RiskScore value during Multivariate analysis was HR = 5.24, 95%CI = 2.74–10.03, *pp* < 0.001 (Figure 13(b)). The results proved that the newly developed 5-gene signature showed a good prediction performance during clinical applications.

### 3.8. Comparison between the Risk Model and Other Models

**Figure 14:**
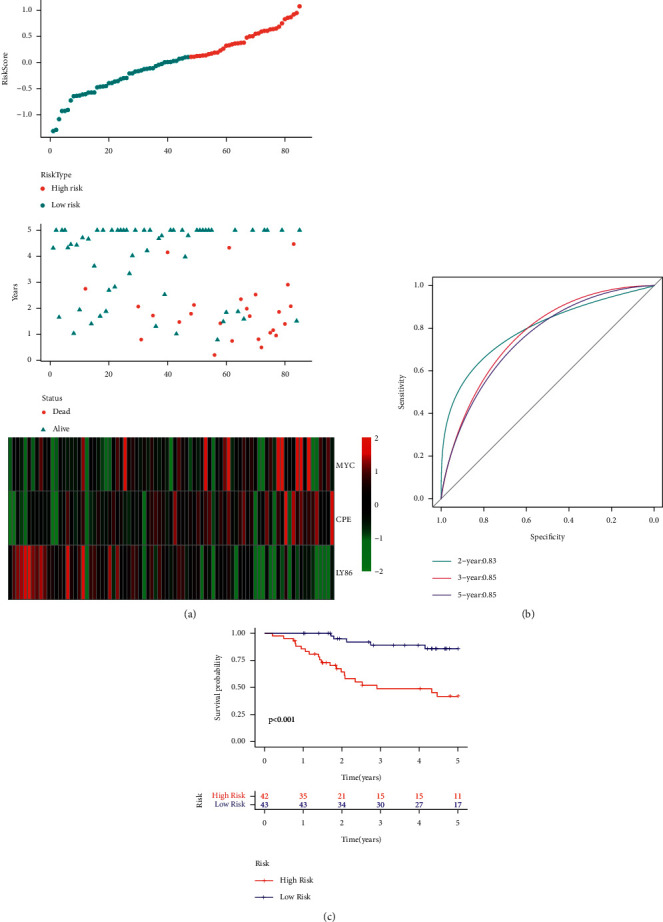
(a) RiskScore, survival status, survival time, and expression of 3 genes retrieved from all the TARGET datasets. (b) ROC curve and AUC of the 3-gene signature. (c) Distribution of KM survival curves for the 3-gene signature included in all the TARGET datasets.

**Figure 15:**
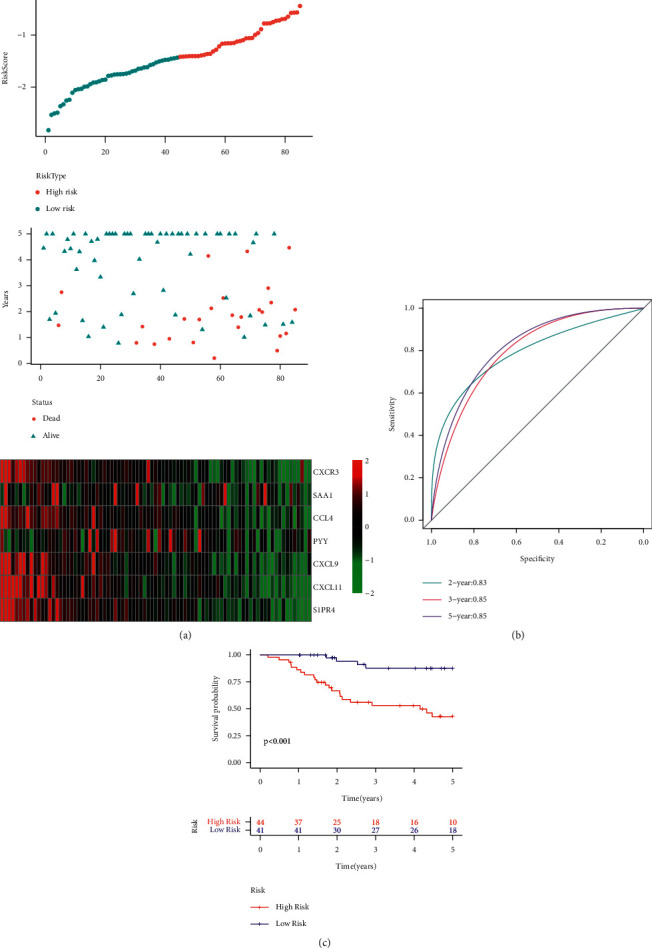
(a) RiskScore, survival status, survival time, and expression of 7 genes retrieved from all the TARGET datasets. (b) ROC curve and AUC of the 7-gene signature. (c) Distribution of KM survival curves for the 7-gene signature included in all the TARGET datasets.

After reviewing all the literature, the researchers selected 2 prognosis-based risk models: a 3-gene signature [11] and a 7-gene signature [12], for comparing the performance of the newly constructed 5-gene signature. For ensuring a fair model comparison, they determined the RiskScore of every OS sample included in the TARGET database, using a single technique, based on the analogous genes included in the models. The RiskScore was then treated using the z-score, and samples with z-score values > 0 were categorized into the high-risk group, while samples with z-score values < 0 were categorized into the low-risk group. The researchers then estimated the OS prognosis difference between both groups.  Figure 14 and Figure 15 present the ROC and the OS-KM curves for both models, respectively. The results indicated that both the models showed lower AUC values, for the 2-, 3- or 5-years, compared to our model. Our model used a rational gene number and displayed a better performance. Additionally, it was noted that the 2 models could also effectively differentiate between both the risk group samples (*p* < 0.001).

## 4. Discussion

The irreversible stall in cell division is known as cell senescence. Typically, under duress or with time, the cell cycle and DNA replication slow down, while the normal physiological functions and the cell proliferative capacity deteriorate, and these changes are accompanied by morphological and functional changes, abnormalities in metabolism, and a deterioration of the immune system [13]. Aging is characterized molecularly by the accumulation of gene mutations, epigenetic alterations, aberrant mitochondrial function, decreased expression of cell cycle regulators, higher expression of cell cycle inhibitors and aging-related genes, decreased efficiency of DNA, RNA, and protein synthesis, and suppressed expression of genes involved in DNA damage repair [14]. Tumorigenesis and aging are closely related, as they promote and influence one another. Tumorigenesis is a natural outcome of aging to some extent, and aging is a significant risk factor for tumorigenesis [15].

Currently, the free radical theory [16] and telomere theory are the two most widely accepted theories of aging. Eukaryotic cells have a unique structure called a telomere at the end of their chromosomes that can build telomere DNA using its internal RNA as a template to preserve telomere length and allow unrestricted cell division [17]. Recent research has shown that only germ cells and hematopoietic stem cells display telomerase activity. On the other hand, 85–90% of the tumor cells have telomerase activity, indicating that telomerase and tumor development are closely linked. Osteosarcoma is a very prevalent and malignant form of bone tumor that occurs in adolescents and young children. Its prevalence has increased in the past few years. The prognosis is really poor, the degree of malignancy is very high, and the development is quicker. Earlier studies have noted that telomerase is overexpressed in malignant bone tumors rather than benign bone tumors, indicating a bad prognosis [19]. Following telomere inhibitor therapy, the telomere length in osteosarcoma drastically decreased and telomerase activity reduced [20]. As a result, osteosarcoma incidence and development are tightly linked to genes associated with aging.

In this research, we initially classified osteosarcoma into two subgroups based on genes associated with aging and its prognosis. Cluster 1 showed a significantly better disease prognosis than Cluster 2. It has been discovered that as we age, immune cells become less responsive to antigen stimulation, and the body's immune defenses become less effective, which can initiate the onset and development of tumors. As a result, we examined the infiltration of immune cells between various subtypes. The results revealed that the immunological microenvironment varied significantly between the two subtypes, which may account for the difference in survival rates between the patients in these two subtypes. Then, a risk model based on genes associated with aging and prognosis was created, and finally, a 5-gene signature containing TERT, GPX4, ERCC4, EPS8, and STAT5A was generated. Telomere Reverse Transcriptase (TERT) was a catalytic subunit of telomerase that shows a biological activity. It is crucial for maintaining the telomere length of telomerase, which allows eukaryotic cells to grow indefinitely [21]. TERT promoter mutations are linked to higher mRNA expression and telomerase activity in a range of tumors, according to studies [22]. For instance, TERT promoter mutations increase the expression of the TERT gene, and gene polymorphisms are linked to prostate cancer invasion and a bad prognosis [23, 24]. Numerous malignant cancers, including thyroid carcinoma, head and neck cancer, cervical cancer, and urothelial carcinoma, have been linked to elevated TERT expression [25–28]. Osteosarcoma cells that are resistant to cisplatin exhibit high levels of TERT expression. During cisplatin treatment of the osteosarcoma cells, the TERT is transported from the nucleus to the mitochondrial cells and is subjected to cisplatin treatment [29]. The prognosis of osteosarcoma patients can be predicted using a 6-gene signature (including TERT) that is constructed based on the apoptosis-linked genes [30]. Glutathione peroxidase 4 (GPX4) is commonly considered a useful indicator of iron death and is crucial for maintaining oxidative homeostasis. The GPX4 protein is responsible for removing lipid peroxide. Lipid peroxide breaks the oxidation balance after GPX4 inactivation, disrupts membrane integrity, and induces iron death [31]. GPX4 acts as an oncogene and is highly expressed in many malignant tumors [32–34]. Since tumor samples have low methylation in the GPX4 promoter region, low methylation and high histone acetylation may lead to the overexpression of GPX4 in tumor cells. Reduced GPX4 protein levels in osteosarcoma result in iron death and enhanced cisplatin sensitivity [36]. ERCC4 is a very crucial molecule involved in Nucleotide Excision Repair (NER). Breast cancer risk is enhanced by the Rs13181 polymorphism of the ERCC4 gene [37]. ERCC4 is linked to the onset or progression of bladder cancer [38], gastric cancer [39], oral cancer [40], and colorectal cancer [41]. Earlier studies have shown that the ERCC4 gene is differentially expressed in osteosarcoma and normal tissues [42]. It is also seen that the concentration of ERCC4 mRNA in peripheral blood cells is correlated with the response of osteosarcoma to chemotherapy [43]. One of the critical kinase-active substrates of the Epidermal Growth Factor Receptor (EGFR) is called Epidermal growth factor receptor Pathway Substrate 8 (EPS8). EPS8 is a signal molecule that regulates many signaling pathways and biological activities in the cells. It also helps in maintaining cell proliferation, differentiation, and survival. The overexpression of EPS8 can be noted in many types of cancers like pancreatic, colorectal cancer, oral squamous cell carcinoma, esophageal squamous cell carcinoma, adenocarcinoma, cervical squamous cell carcinoma, etc., as it was seen to be closely related to tumor occurrence, progression, invasion, and sensitivity to chemotherapy [44–47]. One of the STAT5 subtypes is known as Signal Transducer and Activator of Transcription 5A (STAT5A). Nasopharyngeal carcinoma, breast cancer, prostate cancer, leukemia, and other malignancies can occur owing to abnormal STAT5 activation and overexpression [48, 49]. Since overexpression of the activated STAT5A promotes cell cycle progression whereas STAT5A inactivation inhibits it, researchers concluded that STAT5A could be crucial in the development of tumors [50]. Patients with osteosarcoma with a low STAT5A expression show a poor prognosis [51]. In the past, none of the researchers have thoroughly studied the five genes involved in osteosarcoma. Future investigation is necessary to confirm the mechanism of the five genes that contribute to the onset and progression of osteosarcoma. The researchers used the newly constructed risk model for estimating the risk scores for every sample, and then depending on their risk score values, they categorized the samples into high- and low-risk groups. The high-risk group patients showed a poor prognosis compared to the low-risk group patients. They also determined the robustness and reliability of their risk model using the internal and external validation sets. The results indicated that the risk model had a good performance. In comparison to the osteosarcoma risk models published in the past, the 5-gene signature risk model developed in this study showed a better risk prediction.

Nevertheless, there are still some limitations to this research. First, we only applied osteosarcoma samples and could not carry out strict intergroup condition control, which may lead to deviation and lack of verification of real clinical data. Secondly, the protein level and specific biological function were not verified. Third, the role of the relevant signal pathways screened in osteosarcoma is not clear. Fourth, the small sample size in the current research necessitates more research to reinforce and confirm the stability of the risk model. Further research and trials in the field of molecular biology are warranted.

## 5. Conclusions

The 5-gene risk model that was constructed using the aging-linked genes could precisely predict the prognosis of osteosarcoma patients and assist in making proper clinical decisions.

## Figures and Tables

**Table 1:** Sample information table.

Clinical features	TARGET-OS	GSE21257
*OS*		
0	56	30
1	29	23

*Gender*		
Male	48	34
Female	37	19

*Age*		
≤15	46	21
＞15	39	32

**Table 2:** Sample grouping information.

Clinical features	TCGA-all	TCGA-test	TCGA-train	*P* value
*Age*				
≤15	46	12	34	0.813
>15	39	12	27	

*Gender*				
Female	37	11	26	0.979
Male	48	13	35	

*OS*				
0	56	17	39	0.726
1	29	7	22	

## Data Availability

The data used to support the findings of this study are available from the corresponding author upon reasonable request.

## Supplementary Materials

Supplementary Materials Supplement Table 1: TARGET-OS dataset including 85 samples, comprising 302 genes. Supplement Table 2: 91 prognosis-related genes using TARGET expression profile data. Supplement Table 3: 34 aging-related genes with a significant difference.
