# Supplementary material for: Construction of Molecular Subtype and Prognosis Prediction Model of Osteosarcoma Based on Aging-Related Genes
Source: J Oncol. 2022 Sep 16;2022:8177948. doi: 10.1155/2022/8177948 (PMC9507679; doi:10.1155/2022/8177948)
Supplement: Supplementary Materials — Supplement Table 1: TARGET-OS dataset including 85 samples, comprising 302 genes. Supplement Table 2: 91 prognosis-related genes using TARGET expression profile data. Supplement Table 3: 34 aging-related genes with a significant difference. [file 8177948.f1.docx]

**Supplement Table 1. TARGET-OS dataset including 85 samples, comprising 302 genes**

TARGET-40-PASUUH-01A TARGET-40-PAUTWB-01A TARGET-40-0A4I0S-01A TARGET-40-PARJXU-01A TARGET-40-PAPWWC-01A TARGET-40-PAUUML-01A TARGET-40-PAMHLF-01A TARGET-40-PAUBIT-01A TARGET-40-PASFCV-01A TARGET-40-PAKXLD-01A TARGET-40-0A4I4O-01A TARGET-40-0A4I48-01A TARGET-40-PASEFS-01A TARGET-40-PARKAF-01A TARGET-40-PATUXZ-01A TARGET-40-PAMHYN-01A TARGET-40-PASEBY-01A TARGET-40-PANMIG-01A TARGET-40-PAKFVX-01A TARGET-40-PASNZV-01A TARGET-40-0A4I4M-01A TARGET-40-PAMYYJ-01A TARGET-40-0A4I0Q-01A TARGET-40-0A4I42-01A TARGET-40-PALECC-01A TARGET-40-PAMLKS-01A TARGET-40-PANSEN-01A TARGET-40-PALHRL-01A TARGET-40-PANZHX-01A TARGET-40-PALKDP-01A TARGET-40-0A4I65-01A TARGET-40-0A4I6O-01A TARGET-40-PALFYN-01A TARGET-40-PATPBS-01A TARGET-40-PAPXGT-01A TARGET-40-0A4HLD-01A TARGET-40-PAUYTT-01A TARGET-40-0A4I0W-01A TARGET-40-PARFTG-01A TARGET-40-0A4HX8-01A TARGET-40-PASSLM-01A TARGET-40-PATKSS-01A TARGET-40-PASYUK-01A TARGET-40-PAPNVD-01A TARGET-40-PATMPU-01A TARGET-40-PAPIJR-01A TARGET-40-PAMEKS-01A TARGET-40-PAVDTY-01A TARGET-40-PANGPE-01A TARGET-40-PAMJXS-01A TARGET-40-PAKZZK-01A TARGET-40-PALWWX-01A TARGET-40-PASKZZ-01A TARGET-40-0A4HXS-01A TARGET-40-PATEEM-01A TARGET-40-PAVECB-01A TARGET-40-PALKGN-01A TARGET-40-PASRNE-01A TARGET-40-PARGTM-01A TARGET-40-PAUVUL-01A TARGET-40-PANGRW-01A TARGET-40-PAVCLP-01A TARGET-40-PAPKWD-01A TARGET-40-PATAWV-01A TARGET-40-PARBGW-01A TARGET-40-PANXSC-01A TARGET-40-0A4HMC-01A TARGET-40-PAUXPZ-01A TARGET-40-PATJVI-01A TARGET-40-PAMRHD-01A TARGET-40-PALZGU-01A TARGET-40-PANZZJ-01A TARGET-40-PATMXR-01A TARGET-40-PATMIF-01A TARGET-40-PANPUM-01A TARGET-40-PARDAX-01A TARGET-40-PAUTYB-01A TARGET-40-PAMTCM-01A TARGET-40-0A4HY5-01A TARGET-40-PAVALD-01A TARGET-40-0A4I8U-01A TARGET-40-0A4I9K-01A TARGET-40-0A4I3S-01A TARGET-40-PANVJJ-01A TARGET-40-0A4I5B-01A

ZMPSTE24 11.67021364 11.30263892 12.32164629 10.55362929 12.07981809 10.7984718 11.88798213 11.93626986 12.41018632 10.5980525 10.87958325 11.68606275 11.80009099 10.75154406 10.88264305 11.85720347 11.75321675 11.77107674 11.78504371 12.23541594 12.00246344 12.47522678 12.31259938 12.4372319 11.27786855 10.2911707 11.9893945 10.01820018 11.56700537 12.72152646 12.16018658 12.15671514 9.77478706 12.97513146 12.49385545 10.32305476 10.86573327 11.95819018 11.906139 11.02167404 11.86495992 11.07614753 11.20884399 9.187352073 11.81618368 10.52649924 10.50382574 9.826548487 12.16302064 12.74609438 11.45018025 12.15165083 10.66533592 12.50655553 13.10966744 11.28713519 9.878050913 11.84862294 11.05866825 11.23361968 11.29519496 11.9225844 11.357552 10.78626963 11.8622499 10.5980525 11.72877085 10.09539702 11.78422586 11.2772874 11.95710204 11.17180229 12.17959813 12.32192809 11.74357219 11.38208359 12.00912879 12.39767463 11.65239686 12.24911345 13.08015131 12.3089078 12.41811614 12.32220984 12.69435789

YWHAZ 16.12676496 14.41078257 15.10697181 14.53205148 14.80926571 13.27568804 14.7055244 14.74383512 15.28511319 12.46454575 15.82291931 15.01148935 16.76133047 13.5115056 12.99665002 14.43697175 14.08098403 14.4134296 14.82962365 15.22204016 15.00956622 14.7137087 14.70897608 14.45500607 15.05816088 12.47167521 14.7652861 13.43475852 15.19086637 15.56388146 15.41976272 14.73100075 13.32825417 15.39463016 13.96758653 12.62067804 13.51026967 15.69653291 13.89339634 14.64092274 15.22539575 14.07497766 14.47573343 12.97154355 14.91904952 14.55686525 14.8128291 13.47649308 15.18712006 14.98236975 15.00991607 15.18967015 13.1310531 15.35548875 15.51656185 14.10041596 14.20487677 14.71376239 13.7534257 15.26850545 14.76689119 14.01985131 14.46243837 14.96149491 15.66597428 14.34679072 15.03977585 12.70022295 13.31924873 14.97719047 15.37873513 13.60130653 15.30563429 15.7511779 14.99612036 14.03264845 15.2931483 15.53606505 14.82396426 14.40540742 16.2101386 15.03299274 15.6369361 13.79796543 15.06907209

XRCC6 13.12541347 12.76735685 13.6471211 13.36864287 14.36686859 11.84627391 12.5943246 13.81788303 13.14178784 11.66977089 14.03591597 13.91335694 14.69620687 12.48406762 11.81698362 12.7477734 13.07514484 13.4046094 13.87967897 13.48230378 12.98388429 14.41646769 14.28857774 13.73036399 13.56450678 13.44138803 13.07815081 13.36057278 13.50233458 14.40161287 13.57447527 12.99841414 11.37232095 14.08289748 13.84038455 11.83605036 11.79116289 12.41256985 12.97566887 12.19690944 13.66555799 12.01785233 12.41151099 11.34374092 13.7361905 12.8019104 11.99010396 12.71617641 13.35933777 14.44430315 12.84156435 13.86360554 12.38720958 13.49822593 14.36946103 12.6976192 13.10377955 12.40167953 13.17383326 13.20411259 13.60002903 12.65351688 12.67595703 14.23959853 12.3426303 13.76673594 13.86756833 11.15418521 13.37368083 12.7084373 13.04882741 13.0409746 13.31755394 14.61798393 13.56212315 12.16961188 12.55074679 14.60964047 13.51014602 13.81398155 14.39740724 13.26722561 14.80079881 13.13683095 13.71059122

XRCC5 13.1083611 12.32979634 14.13691103 12.31203206 14.00140819 11.87229011 13.41864325 14.01863487 13.33998915 13.09572667 13.65720678 12.90895548 14.59578196 12.73280339 12.80170836 12.94836723 12.77334529 12.92053895 13.46441812 13.73396861 13.2179577 13.68387187 14.32860481 12.92053895 13.77252078 13.0202855 12.75384351 12.60848586 13.5024589 14.08729708 13.50593558 12.92888844 11.93036775 13.76269347 14.13209803 11.82217246 13.36029843 13.12331312 13.58777752 13.06743436 13.67683861 13.11958962 12.61746747 11.72238064 14.17289625 12.2629757 12.02410078 11.94763694 14.08762858 13.32881515 12.88168756 13.74504401 11.89254282 14.48545199 14.69875892 12.13346335 12.1341054 13.41256985 13.04131692 12.91569268 13.58249485 13.22430321 13.72568578 12.59362455 13.27859465 12.98886217 13.79512648 11.33539035 12.49685378 12.88817245 13.51434421 12.19905882 14.07313738 14.85870954 13.94873224 12.56033283 13.60987129 13.81508304 13.68068917 12.48356389 14.36529721 14.31642297 14.8962851 13.32319553 13.20105181

XPA 7.930737338 8.30833903 9.652844973 7.714245518 9.390168956 8 8.129283017 9.252665432 8.447083226 7.22881869 9.057991723 8.154818109 9.668884984 7.864186145 7.73470962 8.262094845 8.129283017 8.791162889 9.098032083 8.370687407 8.262094845 9.37721053 10.00281502 8.103287808 8.784634846 7.77478706 9.403012024 8.599912842 8.577428828 8.654636029 7.483815777 9.407267764 7.087462841 9.970105891 9.569855608 6.108524457 7.900866808 8.45532722 7.845490051 7.727920455 9.73809226 8.370687407 8.654636029 6.700439718 9.328674927 8.434628228 7.882643049 7.129283017 8.566054038 8.672425342 8.915879379 9.503825738 7.451211112 8.836050355 9.710806434 8.405141463 7.118941073 8.30833903 7.451211112 9.881113961 8.84862294 9.197216693 8.700439718 8.829722735 8.897845456 9.174925683 9.247927513 8.45532722 8.77148947 8.285402219 9.658211483 8.098032083 8.682994584 9.231221181 9.177419538 7.392317423 8.499845887 9.831307244 9.539158811 8.581200582 11.39285404 7.727920455 9.159871337 9.105908509 9.252665432

WRN 8.016808288 8.14974712 10.21431912 8.271463028 9.733015322 9.567956075 8.930737338 11.08878824 8.960001932 8.758223215 9.041659152 8.654636029 10.99152185 7.539158811 8.864186145 9.116343961 8.654636029 9.786269628 9.087462841 9.873444113 8.603626345 10.40407714 10.27961058 9.887220615 8.584962501 7.882643049 9.505811554 7.442943496 9.686500527 9.847057346 9.816983623 9.491853096 8.005624549 10.66977089 9.616548844 7.6794801 10.51274046 10.06608919 9.579315938 9.808964175 10.07012094 10.42416629 9.09011242 8.388017285 8.994353437 8.022367813 8.388017285 7.50779464 9.957102042 9.799281622 9.240791332 9.987264012 8.781359714 10.20212382 10.92109709 9.878050913 6.87036472 9.556506055 9.527477006 10.85486838 9.581200582 9.243173983 10.43358544 9.529430554 8.087462841 9.638435914 9.204571144 6.988684687 8.758223215 8.696967526 9 9.233619677 10.42836017 10.60825482 9.394462695 9.815383296 9.426264755 10.71338651 10.75988818 10.00140819 10.56224242 9.816983623 10.77231457 9.64385619 9.784634846

VEGFA 12.13538865 12.51274046 14.25539746 13.81977993 11.76859788 13.88931382 11.46862404 13.59642273 11.83802206 12.33622759 17.58818758 12.84842733 12.73872562 12.46480098 12.96217303 12.79116289 12.85135865 13.42953751 12.90989308 11.93405939 10.74146699 12.3818133 15.9371439 15.15129408 16.51190704 10.24555271 12.33901487 12.23062093 10.26561505 13.83160415 12.659327 15.31075477 10.22641219 13.3878827 11.55889898 13.35603924 12.87055737 14.6111401 11.89481776 11.47826403 14.04302728 13.86698909 10.04984855 9.917372079 15.01850448 13.47104009 11.42992974 13.20655654 11.5703301 11.61654884 11.6105635 14.18603684 12.8255551 11.57695666 11.17055104 10.68737568 12.64565843 13.32572707 11.61470984 10.98299357 11.712527 12.14752294 11.80815977 10.32530546 12.83209885 11.21735191 14.01532793 11.88111396 10.68999797 12.88130519 11.75363462 10.63026713 14.11032017 14.8992625 12.59875041 11.68255574 12.61148594 14.62188017 14.71037597 12.45095346 14.28699085 12.08978149 13.21674586 12.69283342 11.97835296

VCP 13.6972934 12.97853172 14.30121037 14.37870127 14.31522034 12.93129155 13.96135925 14.74151965 14.24926163 12.21916852 13.20747196 13.65329295 13.25576626 12.78769854 12.98690862 14.49335512 13.13008787 13.65194861 14.11064643 14.51539185 13.72568578 14.75900391 14.48740043 13.02340784 13.53308634 14.20258301 13.63220004 13.00422047 14.39794197 14.74567432 14.26759139 14.00729012 12.05256805 14.44423844 13.92453418 11.19967234 12.76735685 13.61723786 12.81438219 12.44992242 13.3835693 13.94141447 13.7903485 11.55170826 15.01244995 12.9279631 13.55506839 12.73492127 14.42790206 14.68999797 13.48041156 14.79431432 12.24822409 14.08804285 14.18278229 13.16081685 13.27917527 13.88034881 13.63651134 13.45069577 14.18053081 14.26018448 14.27525154 13.60049371 14.06035818 13.45417075 13.83940065 12.39927792 12.96163056 12.74336181 14.67325386 13.67496462 14.09358262 15.63120548 14.60449532 12.69978932 13.62707784 14.74782584 14.48167332 13.97656412 14.73492127 14.58361169 14.58590145 14.20457114 14.7374058

UCP3 5.832890014 5.727920455 6.807354922 3.459431619 5.209453366 5.64385619 5.523561956 5.584962501 4 4.392317423 6.06608919 5.321928095 5.357552005 4.523561956 6.794415866 5.64385619 5.285402219 6.321928095 6 6.129283017 5.321928095 9.238404739 6.108524457 5.882643049 5.392317423 1.584962501 6.129283017 4.459431619 5.087462841 7.266786541 4.459431619 6.06608919 3.321928095 6.87036472 5.584962501 6.768184325 5.700439718 4.321928095 4.807354922 5.857980995 6.044394119 4.247927513 8.511752654 5.95419631 6.392317423 5.169925001 4.754887502 2 5.727920455 6.169925001 6.539158811 6.06608919 4.087462841 8.266786541 7.409390936 4.459431619 4.857980995 5.807354922 3.700439718 7.247927513 5.129283017 5.044394119 5 6.266786541 4.523561956 6.442943496 5.169925001 6.794415866 7.562242424 5.523561956 6.285402219 5.392317423 7.483815777 5.044394119 6.794415866 5.95419631 5.426264755 5.247927513 7.459431619 5.044394119 7.761551232 5.754887502 6.906890596 6 6.409390936

UCP2 11.93221475 11.65910396 12.20762447 7.491853096 11.15291858 9.204571144 9.887220615 10.51569984 11.90501086 7.636624621 9.808964175 12.84725314 10.06743436 7.930737338 9.805743872 11.40194612 12.23032072 9.276124405 10.509775 12.04268537 12.60848586 11.52552081 10.30378075 10.25856603 10.70303839 11.6926155 9.744833837 10.28193003 11.28540222 12.07045642 12.21280033 9.958552715 10.45224124 11.10590851 13.13955135 8.550746785 7.622051819 11.11634396 10.55554777 10.87805091 10.25620869 7.531381461 11.67727919 9.658211483 11.64385619 11.30720081 7.339850003 8.686500527 10.45224124 10.67154107 11.30549179 10.69696753 9.245552706 10.12023788 11.87958325 9.126704473 12.94909716 10.29347165 9.22881869 10.24079133 11.08480839 11.40087944 11.40141288 10.38370429 10.55650605 11.71467483 11.82932634 9.20701432 9.816983623 11.06743436 11.95310515 10.28771238 9.991521846 12.83940065 9.99859043 11.38046107 12.2737956 10.0768156 9.972979786 8.005624549 12.74083483 11.52405192 12.44112862 10.15481811 11.19721669

UCP1 0 0 0 1 3.700439718 0 1.584962501 0 0 0 0 0 6.857980995 1 4.807354922 1.584962501 2.321928095 0 2 0 0 1.584962501 1 0 0 1.584962501 0 0 2.584962501 0 0 0 0 0 1.584962501 4.087462841 0 0 0 2.807354922 2.584962501 1 0 3.169925001 1.584962501 0 1 0 1.584962501 2.807354922 2 1.584962501 0 2 3.807354922 1 0 2.584962501 0 4.087462841 0 3.321928095 1.584962501 0 2.584962501 3.169925001 0 0 0 2 0 0 1 1 1.584962501 1.584962501 2.321928095 0 3 0 4.857980995 1.584962501 3.321928095 0 1.584962501

UCHL1 10.23242093 8.951284715 6.129283017 12.55818152 10.68999797 12.36276574 12.74945047 4.906890596 10.50878516 7.459431619 9.971543554 12.55242895 13.54387766 10.16616308 12.40806432 8.933690655 12.84960057 7.971543554 13.70498432 12.64363075 12.35645197 7.876516947 11.80130419 13.72430067 11.46964182 14.53241681 9.674192268 10.71080643 13.31444163 10.72536626 13.07146236 9.909893084 8.647458426 12.66222331 13.89415461 11.41837972 9.912889336 9.893301531 12.79238361 12.42940674 10.15860969 12.55026581 10.10328781 12.11958962 12.08314687 11.48028532 9.994353437 10.74399286 12.35149141 14.13081185 10.71767642 11.8474489 5.426264755 12.01436924 14.06802248 10.71080643 11.87036472 13.17461365 10.93295289 13.69816204 12.14146856 12.36823361 12.47952767 6 13.96235381 11.02859678 6.442943496 10.81217731 4.321928095 9.84862294 11.75238065 10.52747701 13.03101191 11.32136443 12.71746223 12.04371086 12.98903964 14.44002562 11.7477734 13.33105688 13.69130733 11.05528244 15.71799765 9.262094845 12.93276839

UBE2I 12.20242997 11.85057755 12.75279876 11.197831 12.27321281 10.69087101 11.66844183 12.10230382 11.47876962 10.77807713 11.90951811 12.53576138 12.82952455 10.03066714 11.12282799 12.12702704 11.39499851 11.6617781 12.93350625 11.81898154 12.39526635 12.00140819 12.59339112 12.14051027 12.14593215 12.39070637 11.11048331 11.71381608 12.07414146 13.19367935 12.37612539 11.3426303 10.34762137 12.38963134 11.74735383 9.957102042 12.07915141 11.70994538 11.06810648 11.77725532 12.60455323 11.04165915 11.31967212 9.751544059 11.15797845 11.84588203 10.48582931 11.1711768 12.41256985 12.57837269 11.3089078 12.61608931 11.17492568 12.43514934 12.09440763 10.98441846 11.37068741 11.99541385 10.95419631 10.85953479 12.56914358 11.85564717 11.17367714 11.25561875 11.74483384 11.8482317 12.95437809 11.10787091 11.01192607 11.39553414 12.16081685 11.30777003 11.47370575 12.79664804 12.58848041 11.07547915 12.46352437 13.53940118 12.09440763 11.55985504 13.87996608 11.84549005 12.77272695 11.96072599 11.87344411

UBB 16.07439237 14.66511381 14.89197352 13.55566759 16.71886729 12.69152544 14.02045914 16.16555477 14.90998681 13.50717522 15.28911832 14.72967385 9.724513853 12.70108982 12.51422091 15.05485865 14.31387503 14.33880601 15.35062846 14.77272695 14.83688866 15.38869003 15.32744739 14.3698018 14.77761492 14.6880317 14.10394343 15.85362633 15.34921211 15.4723418 15.28127806 13.68387187 13.62399577 14.93041395 15.62485256 12.3984765 14.7815134 14.26436929 13.8207773 12.01959073 14.37102788 14.32228027 13.99885483 12.38559281 14.4778215 14.55818152 13.40341153 14.56593508 15.4394738 14.29892174 14.97226185 15.24380621 12.74483384 16.32196332 15.66403055 13.58766033 15.67037963 14.85394303 13.74745873 13.80876312 14.66749967 14.28352249 15.00299077 14.74520161 14.38963134 14.94480355 15.08393633 13.07798397 14.15339369 14.30684493 14.26422266 14.20457114 14.71938882 16.79505036 14.71440652 13.42206477 14.41917017 16.79319686 14.21120389 14.09943 16.47326181 15.18305378 15.84605349 14.14815877 15.8735883

TXN 13.20166449 11.31288296 12.81738343 12.44579084 13.49934763 11.48733762 11.71123677 12.23870328 12.24822409 11.36796071 12.40247917 12.16176174 12.08181628 11.07347215 11.59198977 12.22158712 12.1842555 12.53818893 13.98761931 13.27335853 12.82892983 12.79786414 13.48972255 11.99506047 12.7903485 13.41626974 12.22701419 13.21401549 14.1027139 12.79745889 12.84352853 13.05765334 11.13506795 13.51459078 13.67154107 11.59665567 11.72238064 12.36166968 11.54158066 10.43358544 12.41098127 12.5085376 12.31769525 11.23601419 13.52601011 11.90538701 11.57648435 11.73174329 13.20975796 13.29576893 12.69348696 13.81908136 11.00772811 12.75279876 13.17570548 11.79116289 11.73555602 12.18394547 11.47319838 12.86708565 12.79258697 12.40753333 13.72993932 11.6022349 12.87344411 12.53284291 12.22911922 11.10000522 12.43775207 12.21006248 13.73756424 12.91363743 11.72536626 13.10263189 12.50457074 10.99717948 12.4540422 14.92100408 12.03307881 13.24955793 14.28352249 10.8963324 13.48242984 12.74903138 12.38559281

TRPV1 2.584962501 4.459431619 4.700439718 2.584962501 0 3.169925001 3.700439718 4.169925001 1 3.807354922 5.087462841 4.087462841 3.321928095 3.700439718 4.523561956 2.584962501 3.700439718 4.754887502 1 2.584962501 3.321928095 4.087462841 5.523561956 4.087462841 2.807354922 2 5.169925001 2.321928095 2 4.321928095 3.459431619 4.087462841 0 4.169925001 2.807354922 5.087462841 3.459431619 3.807354922 1.584962501 3.807354922 3 3.169925001 2.807354922 3.321928095 4 3.321928095 2.321928095 1 1.584962501 3.169925001 3.321928095 3.321928095 3.321928095 5.247927513 5.672425342 3.700439718 3.459431619 3.459431619 2 0 2.321928095 5.169925001 2.584962501 4.459431619 1 3.906890596 3.906890596 1.584962501 3.321928095 3.459431619 2.584962501 1.584962501 4 4.321928095 4.700439718 3.321928095 3.169925001 3.321928095 2.321928095 3.807354922 6.882643049 3.807354922 6.087462841 5.169925001 3.700439718

TRAP1 11.15355203 10.91662592 12.9710046 9.129283017 12.07815081 10.3858624 10.4429435 10.83447105 10.34651373 10.22641219 10.36741475 11.32699117 12.57151565 10.10459875 9.269126679 11.22339841 10.56605404 10.64205169 11.35974956 11.4252159 10.93663794 11.22339841 12.47421294 11.23601419 10.76570049 13.10574485 10.11634396 10.22400167 11.63163181 10.99152185 11.41256985 9.726218159 8.8008999 12.07547915 10.97010589 8.495855027 9.398743692 10.20701432 9.905387005 10.2179577 11.13955135 10.04165915 9.920352855 9.276124405 10.54978467 10.85096815 9.25502857 10.18115226 11.11178774 11.79968636 10.02513956 10.71166697 10 11.63526466 10.42731284 9.705632387 9.50779464 10.33427329 10.76735685 10.82813648 11.32755264 11.08148344 11.45121111 10.12799432 11.40087944 10.49585503 11.86573327 9.517669388 10.14847658 9.897845456 10.67771964 10.23601419 11.41679753 11.47573343 11.55937709 9.47370575 10.18982456 12.40487546 12.1589252 10.98797452 13.074476 10.85642553 11.14274528 10.96938652 11.56605404

TPP2 12.16961188 10.29691621 12.40540742 10.85486838 10.9403136 9.469641817 10.32530546 11.30206767 10.76404222 9.194756854 11.27903014 11.68211676 11.10721708 10.61746747 11.20640391 10.35424938 9.861086906 10.56033283 10.6891244 12.11374217 10.89860139 10.83447105 11.33035672 11.59944798 10.88493365 10.23122118 9.908392621 9.539158811 11.69217958 13.04148804 11.74693414 10.24317398 8.487840034 12.98174565 10.84862294 10.77066389 10.24079133 10.47775827 10.56414949 11.87190524 12.31032876 9.754887502 11.06676193 10.12799432 12.39580187 10.33091688 9.876516947 9.108524457 10.87498135 11.61976146 10.89102419 11.6926155 10.08347933 10.32530546 12.66200072 9.033423002 8.758223215 11.08081753 10.71080643 9.533329732 12.06002035 10.31854281 10.7968508 11.35700209 10.9403136 10.31061278 10.8917837 9.824958741 10.37829486 11.62296694 12.9242558 9.169925001 12.53600432 11.712527 12.31684718 11.68562484 11.38154295 13.44901964 10.8587581 9.601770788 12.2647358 10.68999797 11.44449729 11.09341756 11.96434087

TP73 5.584962501 3.321928095 6.754887502 4.169925001 5.882643049 7.209453366 3.807354922 2.807354922 3.584962501 6.044394119 5.247927513 4.807354922 9.016808288 2.584962501 6.539158811 2.321928095 4.523561956 5.044394119 3.700439718 8.696967526 3.906890596 10.30720081 5.523561956 4 4.523561956 3.169925001 5.672425342 2 3.700439718 8.092757141 5.584962501 2.807354922 2.807354922 6.321928095 2.807354922 8.804131021 4.247927513 5.087462841 5.977279923 11.57270023 10.18239435 8.164906927 4.392317423 6.375039431 4.523561956 2 0 1 3.321928095 4.321928095 4 5.209453366 3 9.375039431 4.95419631 3.169925001 1.584962501 5 1 4.169925001 5.584962501 5.357552005 6.06608919 4.392317423 6.64385619 3.459431619 7.882643049 6.965784285 8.6794801 6.918863237 4 4.754887502 5.392317423 4.523561956 8.199672345 6.06608919 6.988684687 11.5112585 5.129283017 8.731319031 7.312882955 6.06608919 6.523561956 4.584962501 5.209453366

TP63 8.375039431 5.614709844 10.93663794 6.894817763 6 5.321928095 7.714245518 7.169925001 7.011227255 5.169925001 4.906890596 8.396604781 9.622051819 4.584962501 6.523561956 8.686500527 7.247927513 5.614709844 6.459431619 6.303780748 8.873444113 6.930737338 11.6617781 6.087462841 4.087462841 2 6.303780748 5.807354922 10.63843591 6.169925001 8.285402219 6.22881869 6.426264755 9.951284715 6.247927513 7.741466986 7 9.21916852 8.807354922 10.04848687 9.002815016 4.584962501 8.696967526 7.665335917 6 7.882643049 5 7.266786541 6.64385619 7.366322214 9.493855449 5.857980995 7.033423002 8.417852515 9.554588852 8.921840937 8.204571144 4.64385619 7.022367813 7.339850003 8.797661526 7.977279923 5.781359714 7.011227255 4.392317423 7.807354922 7.826548487 8.703903573 6.781359714 7.876516947 9.276124405 5.672425342 7.636624621 7.781359714 6.807354922 5 6.14974712 9.620219826 8.948367232 9.37721053 9.753216749 8.348728154 7.592457037 6.321928095 6.64385619

TP53BP1 10.77561028 9.936637939 11.89973454 10.70390357 11.34429591 9.84862294 11.08746284 12.00351791 11.13635034 9.049848549 10.98868469 10.32867493 12.31118066 9.616548844 9.758223215 10.30720081 9.640244936 10.36850646 10.58683979 10.95419631 10.05528244 11.18797059 12.30463652 10.24911345 9.527477006 9.926295995 10.21916852 8.810571635 10.62844554 11.93957921 10.83762793 10.83289001 8.503825738 11.28713519 10.37937837 9.098032083 9.951284715 10.73216743 9.980139578 10.52943055 10.95201316 9.722807531 10.21431912 8.658211483 11.12734954 9.759888183 10.74062404 7.965784285 10.26561505 11.37612539 10.12799432 11.80413102 10.18982456 10.5137276 11.2708793 9.727920455 10.12670447 10.26795708 10.66977089 10.03617361 10.80896417 10.28308835 10.60917874 10.90237511 10.33315535 9.961449694 10.44811631 8.915879379 9.677719642 9.129283017 9.914385132 9.453270634 11.07948478 11.89595389 11.63344937 9.988684687 9.06608919 12.33287573 11.01052811 11.4943556 12.59058705 11.03411115 12.90444646 11.52601011 11.74062404

TP53 9.092757141 13.94836723 11.99964774 6.599912842 9.782998209 12.57270023 11.71639079 15.47011865 9.392317423 11.25443815 11.9068906 10.6329952 10.69783636 7.665335917 7.727920455 8.471675214 9.011227255 8.285402219 10.04848687 10.10328781 10.83762793 9.177419538 13.56033283 11.67330908 9.914385132 7.294620749 8.247927513 7.554588852 12.05697634 9.390168956 12.9436137 11.24911345 9.422064766 13.10623576 9.842350343 14.01026584 16.54772298 10.09934781 9.938109326 11.65955 11.57317378 13.11780541 11.00562455 16.01373703 12.26561505 9.62935662 8.033423002 17.5896292 9.560332834 12.00070427 12.3693247 8.64385619 8.54689446 9.577428828 12.95310515 15.54239712 9.908392621 9.46760555 7.8008999 16.3356346 9.682994584 14.3358788 8.781359714 7.238404739 9.856425529 9.63481105 9.908392621 14.35810171 9.2644426 9.45532722 12.17367714 9.964340868 8.744833837 11.11113567 9.902375114 11.29691621 16.58336195 8.159871337 7.622051819 14.26216827 15.71352077 12.09275714 10.31174832 8.707359132 10.68474862

TOP3B 4.857980995 5.209453366 6.339850003 4.523561956 6.409390936 5.169925001 5.523561956 6.714245518 5.64385619 3.807354922 5.832890014 2.321928095 6.894817763 4.584962501 4.321928095 4.321928095 5 6.14974712 4.247927513 6.169925001 3.584962501 5.672425342 6.339850003 5.554588852 4.584962501 3.321928095 5.523561956 3.169925001 5.169925001 6.375039431 5.087462841 5.209453366 2.807354922 6.491853096 5.426264755 4.700439718 2 4.584962501 5.584962501 3.700439718 5.392317423 5.754887502 5.426264755 3 6.108524457 5.614709844 3.700439718 4.459431619 5 6.22881869 4.95419631 5.727920455 4.247927513 5.491853096 7.614709844 4 4.459431619 5.392317423 5.523561956 5.169925001 5.247927513 5.64385619 5.247927513 5.832890014 4.459431619 3.807354922 6.022367813 4.247927513 4.857980995 4.459431619 5.426264755 4.087462841 6.339850003 6.614709844 6.357552005 4.906890596 4.459431619 5.392317423 6.285402219 6.087462841 7.459431619 5.857980995 6.741466986 4.754887502 6.700439718

TOP2B 11.17492568 11.84784036 13.09060867 12.11080953 13.02496648 11.47623991 12.18363538 12.36303963 12.35727707 11.34872815 12.33650656 11.95492329 13.90256354 11.51668495 12.42074976 11.7874945 11.78545247 12.65620138 12.38127256 12.91419824 11.87036472 13.59537405 12.58214198 11.53332973 12.08480839 10.74315139 12.68977963 9.903881846 11.77478706 13.8922582 12.20792945 12.47218312 10.51076417 13.46658634 13.23017058 10.75321675 11.82177398 12.28366717 11.54303182 11.53040634 12.69827058 11.85096815 12.00772811 10.36413466 13.10656294 12.02997735 11.95492329 10.70303839 12.74273048 12.40753333 12.17242751 13.47649308 11.77190208 12.12509054 13.38383927 12.1582941 10.86727874 12.42574042 12.15703107 12.38370429 13.07614753 11.96866679 12.26502894 12.98992663 11.81097322 12.2708793 13.02980485 11.19352536 12.24881706 11.67154107 12.5464122 11.97584797 13.10787091 12.8637991 12.9235132 11.82773965 11.19475685 13.26253534 12.42757475 13.20273604 13.56104923 12.93479659 14.184178 12.36358725 12.75070699

TOP2A 13.04490635 12.0950673 12.11731843 12.08447624 13.04695345 12.914572 13.19982568 13.0764816 12.64250303 12.68671937 14.0313566 12.97046544 14.77468412 11.38801729 11.55362929 12.60733031 13.45095346 13.29088282 12.72664392 13.93856883 13.5190219 13.80826035 14.08563843 12.22037833 13.09226162 12.25207404 13.78422586 11.74315139 13.14226664 14.87958325 13.75905594 13.57754684 11.08148344 14.47541679 13.23391921 11.83011903 13.15006458 13.99788513 12.39954497 14.19521839 14.71386977 12.57813678 12.42888355 11.78217919 14.07973477 11.27612441 13.18766137 10.89860139 14.03668877 13.66666784 12.28424575 13.1086878 12.20487677 12.78463485 14.21682163 12.34457332 11.16176174 12.18021998 11.88836274 13.20975796 12.36139553 12.33817925 12.71681946 14.68096382 11.2137118 12.85408918 12.33203655 11.85369942 11.73935871 12.53843146 13.40660363 12.99559051 13.44462669 13.86766485 13.80634823 12.85096815 11.91513245 15.64540513 13.95954921 14.07230012 14.29138657 12.48909532 14.18177344 13.37408855 13.90670273

TOP1 11.19352536 12.13185696 12.78033471 9.638435914 11.77930897 11.20884399 11.32811389 12.02513956 12.19167615 10.71424552 11.45224124 11.51520703 14.10238584 11.17554955 11.9199806 12.01993816 12.1049263 11.8181827 11.50382574 12.49360531 11.48532619 12.53405966 12.22821744 12.43462823 11.6926155 11.08480839 12.07881795 9.843921051 11.57127862 12.40779885 11.46403515 11.80372753 9.552669098 13.00650141 12.71746223 10.61194694 10.56128795 11.08679969 11.04302728 11.31628153 12.51742334 11.62479546 11.17741954 10.31854281 12.93018292 11.56271943 11.61700823 10.05663772 12.21977355 12.47269084 11.0721326 12.42416629 11.36468186 12.74251997 12.06406908 11.13378441 10.50680344 11.99399979 12.09275714 12.14784089 12.02167404 12.05154884 12.16270602 12.62502386 11.12023788 12.27233818 13.12815547 10.31628153 11.89102419 11.60733031 12.25944905 10.51668495 12.46071185 12.81297947 12.19260105 11.18673329 11.99717948 12.24763088 12.06002035 10.9822806 12.97888918 11.49285462 12.56295787 11.79279029 12.95365083

TNF 6.741466986 3 6.044394119 2.321928095 4.169925001 1.584962501 3.169925001 4 4.584962501 0 6.539158811 5.754887502 2.321928095 2.584962501 3.321928095 4.087462841 4.64385619 3 3.321928095 6.108524457 5.977279923 3.169925001 4.95419631 2.321928095 4.523561956 2.807354922 4.087462841 2.321928095 3.807354922 3.807354922 3 4.754887502 4.247927513 5.044394119 3.459431619 1 2.321928095 4 1.584962501 3.700439718 4.169925001 3.169925001 6.857980995 2 5.129283017 5.459431619 2 5.044394119 4.95419631 4.087462841 5.209453366 4.321928095 3 2.807354922 3.700439718 1 4.459431619 6 2 2.584962501 2.584962501 1 3 2.584962501 4.700439718 4.584962501 5.321928095 0 2.584962501 2 4.64385619 3.906890596 5.882643049 6.554588852 2.584962501 3.169925001 5.930737338 2 5.523561956 0 6.209453366 3.906890596 4.95419631 5.169925001 4.584962501

TGFB1 13.73545025 14.27917527 15.09160067 13.83506349 15.37751558 12.71166697 13.76279726 14.70644206 14.39720667 12.16867212 14.44352636 15.44621099 11.45481335 12.79258697 14.72520647 14.95542288 14.23646272 14.97311436 15.27273912 13.96542357 14.38242139 14.89320671 14.05001867 15.14286491 14.63956682 13.30862345 13.28178517 13.07112712 14.35011044 15.33852748 15.36969957 11.88874325 12.16584915 13.85330956 14.33336503 13.37204883 12.73237945 15.68414591 13.93055255 14.2209073 14.40560686 13.57471178 12.6183855 13.05985141 14.16176174 14.19905882 11.92518352 13.46989615 15.66044165 14.41065009 13.73386272 14.07539558 13.72845201 14.52521491 14.5703301 14.24101487 13.75175325 13.48997336 13.18146288 13.61401961 15.6417131 15.00965369 13.17882044 11.8004955 14.25015035 13.92870342 14.63605812 13.00615073 13.3885555 13.19444908 14.10058022 14.31642297 14.81678368 14.97167826 14.24644372 14.13314221 14.63730412 14.72712275 14.87934393 15.012319 15.54800908 15.60845698 16.36961438 13.46135154 14.16859378

TFDP1 11.46760555 10.31967212 11.9403136 12.58777752 10.36413466 11.94580958 10.71080643 11.38478375 11.05934446 9.878050913 11.96289601 13.17023805 11.37612539 9.233619677 11.99823782 10.80735492 10.21067134 12.33483193 11.50034397 12.52943055 11.01820018 12.46454575 12.22701419 9.807354922 11.61194694 9.071462363 11.72152646 10.58214198 12.40912571 15.53530576 12.41943355 11.58261245 9.221587121 13.94562672 11.16176174 11.9794252 12.32867493 11.7883105 10.57648435 12.90782954 13.10787091 10.49884921 11.38640142 11.32474311 12.38559281 9.965784285 9.885696373 8.50779464 11.62753388 11.8494051 11.73428623 13.46326892 10.72024426 11.45224124 13.26209485 10.73555602 10.38370429 10.69522829 9.815383296 10.70649602 12.42888355 11.52209107 11.07079181 12.73661333 11.56224242 10.73809226 10.06339508 11.25620869 11.17804233 11.87459719 13.3579643 10.41045135 13.68068917 11.74861218 13.47104009 12.25089054 12.41362793 15.58669321 12.06541613 10.52845411 12.34151882 10.83209885 12.74924094 11.87305955 11.93847694

TFAP2A 9.025139562 9.645658432 6.794415866 6.569855608 10.22520744 6.906890596 6.129283017 7.14974712 5.169925001 9.792790294 10.30035256 8.495855027 8.46760555 7.894817763 6.714245518 8.071462363 7 4.247927513 7.965784285 9.129283017 7.592457037 9.116343961 11.36850646 8.885696373 8.044394119 5.882643049 7.22881869 9.599912842 9.124121312 5 9.73809226 5.614709844 7.238404739 6.459431619 7.46760555 7.882643049 6.614709844 9.22881869 7.129283017 11.40886044 2.807354922 10.30035256 7.888743249 6.87036472 5.321928095 9.025139562 3.169925001 9.250298418 10.3553511 4.392317423 5.285402219 3.807354922 4.392317423 10.47065887 6.108524457 4.247927513 10.39446269 8.027905997 10.33985 6.14974712 8.447083226 4.459431619 4.087462841 2.321928095 6.584962501 10.203348 8.353146825 4.64385619 6.906890596 8.361943774 9.517669388 5.700439718 9.693486957 7.209453366 9.607330314 6.459431619 9.714245518 11.71295682 9.044394119 7.672425342 12.65262093 7.882643049 6.977279923 6.247927513 8.936637939

TERT 2 1 1.584962501 0 0 6.087462841 0 0 1 3.584962501 1 1.584962501 4.754887502 0 6 0 1 3.700439718 1 1 0 1 9.73978061 2 0 5.700439718 1.584962501 0 0 2 1 0 0 2 1 5.285402219 0 2 0 3.700439718 1 5.044394119 0 5.700439718 1 0 0 0 1 0 0 1.584962501 1 1.584962501 1.584962501 1 0 6.209453366 1 5.882643049 2 2 9.202123824 0 4.807354922 2.807354922 1 0 0 0 1 0 1.584962501 0 5.491853096 1 9.933690655 3.321928095 5.882643049 4.523561956 2 1 3.321928095 1.584962501 3

TERF2 10.57553925 10.69957245 11.6105635 10.03204573 11.4003458 8.982993575 10.27961058 11.90162116 10.8153833 9.581200582 10.2772874 11.1376316 11.65015421 9.187352073 9.605479518 10.83841608 10.31174832 10.62479546 11.17055104 11.40939094 11.52061868 11.62342429 10.9076418 11.05392588 10.62479546 10.21067134 10.29462075 9.4325419 10.68650053 10.88340699 11.11178774 10.39874369 8.77807713 11.41943355 11.82456103 9.105908509 10.21674586 10.60084211 10.43462823 10.40301202 11.05460432 10.68474862 10.07547915 9.033423002 11.32361776 10.14338321 9.292321633 10.27146303 10.98085361 10.89254282 10.033423 10.81938079 9.224001674 10.97799537 11.3376219 10.72792045 10.63117706 10.58308277 10.75655632 9.535275377 11.05460432 10.22881869 10.76071995 10.44604941 10.69696753 10.60455323 11.28944258 9.379378367 10.92629599 9.924812504 11.85642553 9.605479518 10.29920802 11.57222651 10.81137469 9.670656249 10.4807902 12.10558118 10.94836723 11.22520744 11.90162116 10.84313591 11.34040649 10.47167521 11.01402047

TERF1 10.28077077 10.11113567 10.49385545 9.483815777 10.90312868 9.296916207 10.4918531 11.07414146 10.43567026 8.413627929 10.64925618 10.04165915 11.92629599 8.960001932 9.22881869 10.85018684 10.54303182 10.08081753 9.97441459 10.25502857 10.28193003 10.54592977 10.97584797 10.05256805 10.1176431 9.303780748 11.14847658 8.948367232 10.18982456 10.74819285 11.01680829 10.54496443 8.710806434 10.79360331 11.10590851 8.016808288 8.731319031 10.9076418 10.07547915 10.53430288 10.68825031 9.243173983 10 8.262094845 10.41468524 9.605479518 9.539158811 8.710806434 10.28424575 10.52454172 10.77066389 10.51668495 9.182394353 9.726218159 11.3553511 10.5849625 9.577428828 10.27496047 8.939579214 10.75905594 10.01262454 10.09803208 13.26707927 10.51274046 9.960001932 9.882643049 10.81137469 8.625708843 9.361943774 10.2772874 10.7903485 9.52160044 9.62935662 10.68562484 10.82654849 9.899356923 10.40620501 11.29519496 10.49884921 10.84784036 12.57270023 9.824958741 11.8989792 10.88569637 10.78463485

TCF3 11.73893668 11.21006248 12.31203206 11.51619248 12.22821744 12.31061278 11.9918761 14.22302125 11.61700823 11.63571812 11.49085088 12.93313737 13.75102095 10.23601419 11.67860014 10.65999589 10.50481899 12.12412131 12.46786024 12.9130764 12.13314221 12.37095979 12.79238361 11.64340528 11.18611424 11.72877085 11.7903485 11.37286506 11.26971112 12.12379809 13.00158412 12.58072965 9.987264012 12.27204652 11.52698821 10.6617781 11.9432474 13.00316651 11.77355135 12.36221782 12.60060985 13.41071633 10.55074679 8.693486957 12.37666806 11.31571566 10.63208641 10.70390357 11.72792045 12.45943162 11.07079181 13.07815081 10.95782756 12.84235034 11.32192809 11.47623991 11.28886607 11.76942464 11.53770375 10.99081308 11.11243951 12.52037314 11.88988417 10.16867212 11.98584194 11.58824615 12.5137276 10.51569984 10.76155123 11.09407769 13.36673201 11.07146236 12.11146174 12.76238204 12.49934763 11.78217919 12.69174352 13.93921188 12.65776504 12.8482317 13.03891899 12.16050175 12.71724801 11.47826403 12.59712143

TBP 8.682994584 8.696967526 9.839203788 5.614709844 9.428360173 7.076815597 9.361943774 7.888743249 8.651051691 7.523561956 9.353146825 9.787902559 10.32755264 7.426264755 8.442943496 8.252665432 8.118941073 9.187352073 9.84862294 9.696967526 8.810571635 8.965784285 9.933690655 8.596189756 9.116343961 9.038918989 8.060695932 8.54303182 8.95419631 10.36303963 9.283088353 8.622051819 7.199672345 9.647458426 8.810571635 8.491853096 10.03204573 9.548821908 7.924812504 9.01402047 9.955649908 7.900866808 8.682994584 6.882643049 9.154818109 8.54303182 8.87958325 8.50779464 9.686500527 9.247927513 8.710806434 9.247927513 7.54689446 8.820178962 9.62935662 7.948367232 8.344295908 8.611024797 7.169925001 7.857980995 8.988684687 8.74819285 9.661778098 9.519636253 8.224001674 8.451211112 9.698704667 7.894817763 9.25502857 8.768184325 9.82336724 8.768184325 8.87036472 10.04575966 9.955649908 8.144658243 9.052568051 10.18115226 9.581200582 10.24317398 11.02375435 8.84862294 11.09011242 8.988684687 9.459431619

TAF1 10.62388149 10.65642486 11.41943355 9.970105891 10.51076417 10.29691621 11.28540222 12.07347215 11.09077405 10.57080444 11.11504365 11.10394343 12.42442876 10.39124359 10.51766939 11.00562455 10.56890615 11.14082977 11.74146699 11.42153891 9.826548487 10.42101286 12.14911199 10.91513245 10.54496443 8.897845456 10.92035286 9.126704473 10.86341196 11.9225844 10.93663794 11.22037833 9.172427509 11.64925618 11.37177664 9.324180547 10.21067134 10.86573327 10.64745843 10.82336724 12.1382718 10.87036472 10.37503943 8.562242424 11.17741954 10.65374078 10.55074679 9.052568051 11.02444712 11.53235593 10.36959735 10.9822806 10.4918531 11.45738088 11.9690267 10.27612441 9.681238412 10.41468524 10.70390357 8.864186145 10.98655315 10.40301202 11.10197567 11.50878516 9.970105891 11.09803208 10.83447105 9.54303182 10.73555602 10.62935662 10.85486838 10.2772874 11.14465824 12.20701432 11.95855272 10.906139 10.36632221 11.3586512 11.10983065 11.28828934 12.61861492 11.46250227 12.06272077 11.73216743 11.32923574

SUN1 11.72280753 12.05562137 13.3416578 11.63481105 12.73047014 10.65105169 11.994707 11.37991982 11.97835296 10.09275714 12.96144969 12.54930337 12.95455985 11.36905201 11.75988818 11.18549492 11.27029533 12.23361968 11.99964774 12.25738784 11.215533 12.94800213 12.78790256 11.95019135 11.94727165 11.27961058 11.44604941 10.67065625 10.88874325 12.27350423 11.68781306 11.75279876 10.169925 12.53600432 12.57648435 10.93147623 11.47623991 12.02859678 11.48179943 11.33706434 12.39740724 11.84352853 10.83762793 10.45327063 12.79907921 11.98655315 10.28077077 10.02097994 12.36796071 11.89708913 12.3877481 12.82634986 10.82017896 11.83051521 12.71102162 11.16050175 9.505811554 11.3426303 10.64565843 11.93847694 12.54061241 11.67198327 12.59781979 12.09539702 11.2039597 12.10328781 11.94178124 11.05866825 10.86031105 11.23840474 12.15987134 10.47471995 13.25841881 13.14990586 12.65082737 11.88073144 12.79745889 13.53418127 12.48658366 12.03651707 13.59584022 13.00351791 12.73555602 13.25944905 11.63979289

SUMO1 11.32192809 10.34872815 12.07581338 11.09407769 12.64633369 9.821773982 11.78586111 11.82495874 11.33706434 9.525520809 11.57459353 10.81297947 12.81598363 10.54785851 10.61654884 11.02097994 10.8864587 10.8963324 11.9918761 12.2974895 11.00842862 11.47370575 12.53867395 11.10852446 11.90312868 11.45789384 10.39553414 11.45789384 11.65060302 11.64790807 10.86805085 10.85252951 9.812177306 12.28106067 12.2227949 9.599912842 11.05866825 10.5849625 11.68781306 10.92851838 11.14847658 11.22158712 11.28944258 10.08746284 12.57175264 10.74062404 10.96794671 9.189824559 11.77313921 11.87920032 11.06272077 11.76362735 10.19844504 12.47699929 13.3081968 10.52061868 10.29347165 11.71681946 10.88874325 10.63571812 11.70735913 11.26150731 11.46862404 10.43879185 11.86650621 11.29404631 11.891404 9.28771238 11.00702727 11.41309898 11.67375074 10.41996018 11.98974928 12.22007597 12.34012827 10.22158712 12.31938988 12.53211237 11.29634268 9.981567282 12.21492619 11.93774162 13.03101191 11.48633225 11.11439305

STUB1 11.04575966 10.81778312 11.5674808 8.968666793 10.95346896 9.182394353 10.17492568 11.30549179 9.667111542 9.396604781 10.43671154 10.93516505 10.45121111 9.044394119 9.651051691 10.80654962 9.743151394 9.700439718 11.14529533 9.997179481 10.03891899 10.44708323 10.94763694 10.53040634 10.14847658 11.73513288 9.749869427 10.3376219 10.55842071 11.45481335 10.71596199 9.321928095 8.581200582 10.39231742 10.20823436 8.531381461 10.6183855 9.368506462 9.73470962 8.962896005 9.622051819 10.25502857 9.632995197 8.154818109 9.438791853 10.93663794 8.64385619 10.10066234 10.59618976 11.0175044 9.124121312 11.0768156 9.315149562 10.66533592 10.15355203 9.485829309 9.995767151 9.689997971 9.86727874 9.722807531 9.751544059 10.42940674 9.571752644 9.670656249 10.44397954 10.01541505 11.99399979 9.753216749 8.665335917 9.326429487 10.07547915 9.483815777 8.948367232 11.24079133 10.90237511 9.301496195 10.39981196 10.79441587 11.23421868 10.38694025 12.11276528 11.84901407 10.42836017 10.1176431 10.70476824

STK11 11.23122118 11.67109873 12.49835061 10.31854281 11.83130724 11.2137118 11.86650621 12.77148947 11.27844946 10.91587938 11.10525378 12.30549179 11.52601011 9.688250309 10.52747701 10.47065887 10.49285462 11.69783636 11.94909716 11.60362634 11.32474311 12.21219237 12.07948478 10.76071995 11.13121391 10.57931594 11.45840661 11.35810171 11.14338321 11.78176951 11.77643303 11.69348696 10.19475685 12.0970445 12.09143539 9.857980995 11.942881 11.63026713 11.38370429 11.12992693 11.24911345 12.10361566 10.66266838 9.269126679 11.44811631 11.38262403 9.912889336 11.02306125 10.99506047 11.99116751 11.03204573 12.17710804 10.42626475 11.669328 11.11958962 10.96794671 11.14911199 10.70303839 10.86805085 10.91960824 10.93442804 12.15323534 10.90237511 10.33873638 11.23062093 11.63662462 12.4812949 10.06339508 10.51569984 10.82017896 12.06507949 10.60362634 10.86108691 12.06844242 11.51076417 11.24911345 11.84077792 12.12412131 12.12315143 11.45584091 12.81177606 11.69696753 11.97046544 11.21006248 11.88264305

STAT5B 10.64835758 11.09803208 12.14402087 9.417852515 11.52747701 10.29576893 10.43775207 10.2772874 10.46250227 9.932214752 10.68650053 11.10263189 12.41098127 8.894817763 8.033423002 10.61930296 10.12412131 11.50183718 10.51865316 10.91064273 10.83447105 10.7548875 12.509775 9.702172685 10.6635581 9.537218401 10.91886324 9.839203788 11.49884921 12.34789814 11.268542 11.09803208 8.839203788 11.27496047 11.87421293 8.640244936 10.89405985 11.58355293 8.988684687 11.17679648 11.08148344 9.194756854 10.94690627 8.54689446 10.32305476 10.29347165 10.03066714 8.54689446 10.99223026 10.76983784 10.55458885 9.805743872 10.25502857 10.84235034 11.46964182 10.74735383 10.11634396 9.857980995 9.485829309 9.45532722 10.37286506 10.5943246 9.388017285 10.40726776 9.719388821 10.82813648 10.87651695 10.00702727 9.736401931 9.984418459 10.52845411 10.73640193 10.19229281 11.23062093 11.54448152 9.465566405 9.8008999 11.92295599 10.49285462 10.84392105 12.4540422 9.409390936 11.59245704 10.98441846 11.57459353

STAT5A 10.07547915 10.38262403 12.19167615 8.438791853 10.92555444 7.77478706 10.14847658 10.01402047 10.10983065 8.661778098 9.262094845 11.02097994 8.252665432 8.321928095 7.761551232 9.948367232 9.475733431 10.26209485 10.48280796 10.13955135 10.52943055 9.776433032 11.57459353 8.658211483 9.503825738 8.588714636 10.56510208 8.539158811 11.19721669 10.19721669 9.525520809 10.68999797 9 10.84392105 11.51766939 7.209453366 8.603626345 10.56128795 8.906890596 10.09407769 9.68474862 7.900866808 10.83051521 8.209453366 9.238404739 10.20089861 8.13442632 8.266786541 9.603626345 10.42206477 10.06204614 10.15860969 9.688250309 10.24555271 10.88417052 9.74819285 10.00140819 9.511752654 8.276124405 8.326429487 10.45532722 10.33539035 7.569855608 8.569855608 9.199672345 10.22881869 10.68299458 8.84862294 8.939579214 8.727920455 9.54689446 10.09803208 8.787902559 11.06204614 11.33371443 8.071462363 9.071462363 9.071462363 10.52356196 9.315149562 11.90914305 10.06474276 10.74651432 10.28540222 10.87267488

STAT3 12.26883437 12.89557528 14.06111801 13.09539702 13.37272905 12.55314928 12.58002297 12.43410693 13.12557491 12.78545247 13.14098949 13.20625127 12.8558418 11.82456103 11.13955135 13.45507031 12.14974712 13.53491077 13.52454172 13.38680556 12.73830341 14.00465939 14.90979935 13.83348311 13.34831321 11.93258387 13.78688219 11.98903964 13.36276574 13.85720347 12.70476824 14.14051027 11.35974956 13.86234677 13.95328707 11.12992693 11.95128471 13.4763665 11.49685378 12.28135051 13.91550596 11.15797845 12.85506312 10.60640521 13.17897601 12.37964912 12.31684718 11.46352437 14.0172434 13.69239756 12.29203399 13.47990654 12.77499291 13.61367438 13.574357 13.27554256 12.36986994 11.90839262 12.62616532 12.84490188 13.10966744 12.66333572 12.42442876 12.48557777 12.44320258 13.14354265 13.48771445 11.87113518 11.09407769 12.11080953 12.71660514 12.24970606 12.70043972 13.9127958 13.82217246 11.70476824 11.99364606 14.4519194 13.70530839 11.24079133 13.91970134 12.95873395 13.77231457 13.45827844 13.58096514

SSTR3 4.392317423 2.807354922 2 2 1.584962501 0 0 2.321928095 2 0 2.321928095 1 7.6794801 1 4.169925001 3.321928095 1 5.169925001 0 1.584962501 2 1 2.584962501 2.807354922 1.584962501 0 2 1.584962501 1.584962501 1 1.584962501 0 1 2 2.584962501 2 1 2.584962501 1.584962501 2 1.584962501 0 3.459431619 3.807354922 1.584962501 3 1 0 2.321928095 2.321928095 0 2 2.807354922 3.169925001 1.584962501 0 2 3.169925001 1.584962501 3.321928095 0 1 0 1.584962501 2.321928095 1.584962501 3.700439718 0 1.584962501 1 2.321928095 1.584962501 2 3.169925001 0 1.584962501 2.321928095 1.584962501 2 2 10.75655632 0 0 2 3.321928095

SST 0 0 0 0 0 0 0 0 0 0 0 0 2.321928095 0 4.807354922 1.584962501 1 0 0 1 0 0 0 0 2.584962501 0 0 0 1 4.087462841 0 0 0 0 0 2.321928095 1.584962501 1 0 2 0 0 0 1 0 0 0 0 0 0 1.584962501 0 0 0 2.807354922 0 2 0 0 1.584962501 0 0 0 0 1 2.321928095 2.321928095 0 0 1 0 1 1 0 1 0 1 1.584962501 0 0 1 0 1 1 1.584962501

SQSTM1 11.66088727 12.70692764 14.54145966 11.75863964 13.33887563 10.37395266 12.21188829 13.76890797 13.30049556 11.08812569 12.17866485 13.86495992 12.99753235 11.65776504 11.30777003 12.10033382 12.82694565 11.96938652 12.98744167 12.10295989 12.28308835 12.63956682 14.60767707 12.86011703 12.65082737 11.45378506 13.18673329 11.35590164 12.3426303 12.7548875 12.90180968 13.76911466 11.19721669 12.86147468 14.34637522 10.95782756 11.14974712 12.79197682 11.91326343 10.87036472 11.79279029 11.9436137 12.41732512 10.99788513 12.15766273 12.85408918 11.15228484 10.84392105 12.79502498 13.84568606 11.65776504 14.22573464 11.11243951 14.56301747 13.1584519 12.30178196 10.73216743 11.77478706 12.72003045 11.86263736 12.02859678 12.78258876 12.08514046 11.28944258 11.84666568 12.6379833 13.5044466 10.75321675 12.01506653 11.22821744 12.82873153 10.99788513 12.05460432 15.56599456 13.08796001 11.25502857 11.62296694 13.17632901 13.92573986 12.35121532 13.57009287 12.6794801 13.92787053 12.69609817 13.04148804

SPRTN 8.675957033 8.169925001 9.461479447 7.348728154 9.988684687 7.562242424 8.413627929 9.296916207 8.921840937 7.73470962 8.707359132 8.409390936 9.832890014 7.614709844 7.807354922 8.252665432 8.851749041 8.21916852 8.330916878 8.842350343 8.87036472 8.861086906 9.894817763 9.159871337 8.087462841 7.918863237 8.622051819 6.022367813 8.409390936 9.649256178 8.921840937 9.409390936 6.882643049 9.54689446 9.670656249 7.392317423 6.672425342 8.299208018 8.129283017 7.813781191 9.164906927 8.204571144 8.209453366 8.071462363 9.539158811 8.071462363 7.924812504 6.50779464 8.864186145 9.571752644 8.383704292 8.900866808 7.247927513 9.588714636 9.396604781 7.888743249 7.761551232 9.116343961 8.321928095 8.982993575 8.607330314 8.592457037 9.296916207 7.95419631 8.924812504 8.535275377 8.930737338 7.285402219 7.475733431 8.189824559 9.588714636 7.22881869 8.845490051 9.987264012 9.805743872 7.977279923 9.036173613 9.184875343 9.330916878 8.271463028 10.6926155 8.936637939 10.33427329 8.761551232 9.594324604

SP1 11.82296909 11.83644491 13.00965369 11.67639789 12.45712433 11.35480034 12.32220984 12.81277897 11.95673915 10.68123841 12.24138736 12.18889787 12.42101286 10.66266838 11.24198315 12.06676193 12.06608919 12.27117119 12.17990909 12.70649602 12.17835362 12.20945337 13.29390267 12.10361566 11.6956633 10.67595703 11.71810471 10.40939094 11.99964774 13.16128937 12.79136641 12.03686045 10.5372184 12.68101874 12.55985504 10.45840661 12.04028972 12.68277518 11.65910396 12.31486643 12.7281331 11.91101741 11.69130733 10.32642949 13.00457162 11.35590164 11.09011242 9.596189756 12.31118066 13.35727707 12.22641219 11.82057788 11.35314683 12.05018877 12.90501086 11.49135207 10.89254282 11.88912366 11.13955135 11.51963625 12.38667086 11.76321237 12.07045642 11.8622499 11.2179577 12.16081685 12.13057056 10.85174904 11.23900176 11.4003458 12.53357308 10.93442804 12.44320258 12.9275928 12.85311458 12.06440596 11.40247917 13.63651134 11.97226185 11.55794229 13.60060985 11.92035286 13.75279876 11.91438513 12.26209485

SOD2 11.9432474 11.26561505 13.99497211 12.76590764 11.69740201 10.65821148 12.57317378 12.76880462 12.76092781 10.43775207 11.84979602 13.33678548 13.28496865 10.82495874 10.87190524 11.28308835 11.05799172 12.70628016 12.31316648 12.71209705 11.99045857 12.30492167 14.20670915 11.63390341 13.69533706 11.25797706 11.92814822 11.05052891 13.0313566 13.08381171 11.54737656 13.5946745 11.07881795 13.11601899 12.59525748 12.46964182 9.909893084 11.67198327 11.10328781 11.27029533 12.02583167 10.64205169 13.72152646 9.167418146 11.45481335 12.2137118 11.54061241 9.594324604 12.48028532 12.29634268 13.35686458 14.6022929 11.26561505 13.29648609 12.41626974 11.64520808 9.890264277 11.97226185 11.43567026 11.12799432 12.5464122 13.20655654 11.81578354 12.6976192 11.169925 11.76777065 13.42035502 10.23002044 12.07981809 11.73640193 11.906139 11.75154406 11.9465408 14.01026584 12.38208359 10.87113518 11.94141447 13.26385602 15.28551059 12.7054164 12.69913863 12.56914358 13.28626896 12.63413037 12.87421293

SOD1 12.60848586 11.7198166 11.78790256 10.87036472 13.34886644 11.1176431 11.63344937 11.8761332 11.83802206 11.73724734 11.51471405 11.52209107 13.00035218 10.67419227 10.94690627 12.65015421 12.41045135 12.24138736 12.70649602 12.50457074 12.16679075 12.86650621 13.09885455 11.27786855 12.83051521 13.38882454 12.68321395 13.1283166 13.24480978 13.46135154 11.77066389 12.76051205 11.21310422 13.01419487 13.86949745 10.56224242 11.37340896 11.55650605 11.28135051 11.02859678 12.89368074 11.90463462 11.92407019 11.90312868 11.59572369 11.40939094 11.16302064 10.74735383 12.5992155 13.19737029 13.27248399 13.80685166 11.16679075 15.75397405 13.29691621 11.27029533 11.69435789 11.37883671 11.50630758 11.47623991 13.68507727 12.33706434 12.91045536 12.05358654 12.24792751 11.9854862 11.8474489 10.9248125 11.72536626 11.83447105 13.4318893 13.36850646 12.41309898 13.05290763 14.01384605 11.73893668 12.49360531 14.62633646 13.29404631 13.28525771 13.12379809 11.95164899 13.55566759 12.65374078 12.5115056

SOCS2 9.027905997 8.204571144 9.59058705 9.047123912 10.09671515 8.693486957 9.202123824 6.266786541 7.651051691 7.64385619 7.948367232 9.596189756 10.55937709 7.515699838 7.409390936 9.812177306 9.079484784 7.77478706 9.779719355 8.807354922 10.54592977 10.53332973 11.9068906 9.154818109 7.839203788 6.768184325 8.933690655 8.463524373 10.94836723 9.366322214 8.6794801 11.39124359 6.357552005 9.471675214 11.1382718 7.199672345 9.221587121 8.54689446 8.724513853 9.074141463 9.50779464 9.495855027 8.784634846 7.348728154 10.99293834 7.864186145 7.375039431 6.672425342 9.48984796 9.257387843 9.991521846 7.832890014 8.144658243 7.794415866 13.13602985 5.857980995 8.921840937 7.857980995 8.651051691 8.447083226 10.13955135 8.375039431 8.247927513 7.108524457 8.022367813 8.87036472 8.665335917 8.581200582 7.139551352 9.769837844 11.66088727 7.392317423 7.636624621 10.13185696 9.194756854 9.002815016 7.754887502 7.787902559 8.768184325 5.554588852 10.59152235 9.027905997 9.116343961 8.672425342 8.903881846

SNCG 10.68650053 7.971543554 6.894817763 4.700439718 9.703903573 3 5.807354922 6.247927513 6.06608919 3.169925001 6.392317423 8.252665432 10.4918531 6.129283017 5.930737338 6.375039431 8.011227255 5.807354922 8.531381461 6.64385619 9.751544059 7.54689446 6.700439718 5.700439718 7.108524457 6.129283017 5.169925001 7.672425342 7.936637939 6.754887502 6.50779464 2 7.06608919 6.087462841 9.162391329 4.247927513 5.614709844 7.483815777 8.038918989 4.459431619 6.357552005 5.781359714 7.592457037 6.044394119 6.129283017 7.686500527 6.303780748 6.754887502 6.614709844 6.95419631 6.584962501 5.906890596 5.426264755 6.539158811 8.54303182 5.584962501 7.781359714 6.942514505 5.247927513 4.857980995 8.625708843 6.569855608 5.044394119 4.392317423 8.174925683 7.21916852 8.233619677 5.672425342 7.741466986 6.95419631 9.74819285 6.303780748 6 10.76818432 7.348728154 6.357552005 8.392317423 4.95419631 5.357552005 3.169925001 9.136991112 4.523561956 7.592457037 5.392317423 7.839203788

SLC13A1 0 1 3 0 1.584962501 0 2 0 0 1 0 2 2.584962501 1 6.266786541 2.584962501 2.807354922 1.584962501 0 0 0 1 0 1 0 0 0 1 0 1 1 2.807354922 1 1.584962501 1 4.247927513 0 1.584962501 1 0 1.584962501 0 0 5.614709844 0 0 1.584962501 0 1 1 0 1 0 1 1 0 0 0 1 2.321928095 1 0 0 1.584962501 2.321928095 2.807354922 0 3.321928095 2 2.321928095 0 0 1 0 1 1.584962501 1 0 0 0 1.584962501 1 2 0 0

SIRT7 11.26150731 9.77148947 10.73301532 8.238404739 9.350939182 9.463524373 8.707359132 10.30833903 8.942514505 6.614709844 10.05934446 10.45429929 8.710806434 7.876516947 10.2467406 8.396604781 8.876516947 9.21916852 10.4807902 9.729620744 10.04439412 10.99788513 9.751544059 10.44501485 9.921840937 9.584962501 8.758223215 10.23122118 9.596189756 10.60455323 8.900866808 8.980139578 7.768184325 9.908392621 10.66088727 8.098032083 9.854868383 8.426264755 10.06204614 8.566054038 9.722807531 8.894817763 8.531381461 7.129283017 9.588714636 9.707359132 7.169925001 7.924812504 9.436711542 9.952741247 9.824958741 10.71596199 7.129283017 8.625708843 11.45429929 7.383704292 8.982993575 8.87958325 9.407267764 9.202123824 9.659995892 9.698704667 9.285402219 8.87036472 9.405141463 9.987264012 10.53235593 9.076815597 8.511752654 9.113742166 8.971543554 8.262094845 9.596189756 10.62205182 9.698704667 9.252665432 10.52061868 9.851749041 10.06339508 7.87036472 11.41785251 9.016808288 10.28655776 8.751544059 10.52258153

SIRT6 11.20762447 9.199672345 10.36303963 8.011227255 10.03891899 8.781359714 9.607330314 10.74986943 9.586839788 8.483815777 9.041659152 10.32979634 10.25266543 8.06608919 8.550746785 9.831307244 8.784634846 9.499845887 10.12412131 9.992938336 9.885696373 9.985841937 10.31061278 8.820178962 9.539158811 9.002815016 9.266786541 10.23720996 10.21916852 9.005624549 9.967226259 10.50878516 8.422064766 10.43671154 10.36084708 8.21916852 9.238404739 9.971543554 9.245552706 9.06608919 9.636624621 9.939579214 8.495855027 7.442943496 9.071462363 9.202123824 7.807354922 12.93737382 9.276124405 10.18735207 9.413627929 10.6128685 8.383704292 9.908392621 9.224001674 8.731319031 9.682994584 9.118941073 9.240791332 8.087462841 9.649256178 10.34983409 8.247927513 7.727920455 9.301496195 9.357552005 10.69522829 8.247927513 10.23720996 8.888743249 10.29806257 9.131856961 9.087462841 10.76901132 10.45224124 8.700439718 10.18363538 10.45121111 10.22761594 9.471675214 10.87574935 9.985841937 9.87036472 9.169925001 10.20579325

SIRT3 9.733015322 9.882643049 10.72451385 7.768184325 10.55842071 8.144658243 9.607330314 9.5980525 9.804131021 8.124121312 9.677719642 9.445014846 10.2179577 8.071462363 7.714245518 9.071462363 8.344295908 10.06608919 9.943979914 10.14082977 9.50779464 9.601770788 10.96000193 8.647458426 9.063395081 8.614709844 8.584962501 9.283088353 9.147204925 9.816983623 8.829722735 8.290018847 7.965784285 9.727920455 9.810571635 7.375039431 8.717676423 9.22881869 8.266786541 8.588714636 8.912889336 9.731319031 8.459431619 9.321928095 9.027905997 8.906890596 6.988684687 8.087462841 10.02652344 10.10197567 8.842350343 9.832890014 8.62935662 9.759888183 10.00842862 9.147204925 8.6794801 8.813781191 7.768184325 8.651051691 10.06069593 9.86727874 8.45532722 9.202123824 9.008428622 10.31854281 10.07280253 7.569855608 8.483815777 8.661778098 9.805743872 9.677719642 8.758223215 9.781359714 9.961449694 7.95419631 9.531381461 10.67330908 9.209453366 10.05392588 11.73131903 9.797661526 10.02236781 9.614709844 9.535275377

SIRT1 8.951284715 9.625708843 10.88722062 9.299208018 10.28886607 8.011227255 10.1176431 10.92777796 9.379378367 8.169925001 9.396604781 9.50779464 10.91886324 8.689997971 9.808964175 8.781359714 8.768184325 9.54689446 9.283088353 10.27496047 8.882643049 9.997179481 10.18115226 10.39124359 9.049848549 8.787902559 9.344295908 6.523561956 9.328674927 11.2974895 10.09275714 9.503825738 7.754887502 9.927777962 10.53527538 8.348728154 7.851749041 9.060695932 9.353146825 9.596189756 10.39124359 10.20089861 9.668884984 8.087462841 10.21067134 8.84862294 8.665335917 7.383704292 9.727920455 10.50581155 9.192292814 10.15101654 8.426264755 9.906890596 10.68474862 8.845490051 8.28077077 9.47370575 9.820178962 8.794415866 10.11504365 9.726218159 10.15860969 9.674192268 9.632995197 9.677719642 10.68825031 8.960001932 9.442943496 8.87958325 10.71596199 8.781359714 10.45018025 10.44708323 10.87805091 10.33985 8.942514505 11.50581155 9.83447105 9.233619677 10.6891244 9.079484784 11.92109709 9.129283017 10.14720492

SIN3A 11.36905201 11.07748336 11.85525783 11.70303839 11.99576715 11.82376528 11.64835758 11.69653291 11.30947635 9.948367232 11.81618368 11.97906787 12.09934781 10.44086917 10.60362634 11.53915881 11.55074679 10.93442804 11.80574387 11.942881 12.05460432 11.73851453 12.81197669 11.73597904 11.27844946 10.29576893 11.62159404 10.51963625 11.09011242 13.01906943 11.58590145 11.48784003 10.33427329 11.50581155 11.68343329 10.58683979 11.3586512 11.87382857 10.31061278 11.60316268 12.10066234 10.41468524 11.02721489 10.14974712 11.64385619 11.00912879 10.36194377 9.121533517 11.51619248 12.28395649 11.02097994 11.85213933 10.92629599 12.52674374 12.21613956 10.40514146 10.9505559 10.62844554 10.61930296 11.52454172 11.67683861 10.31741261 12.4000789 11.66311331 10.15355203 11.7903485 11.35590164 10.42940674 10.82097669 10.41996018 11.33427329 10.23242093 11.00351791 12.02340784 12.22400167 11.36468186 10.42626475 13.44772899 11.24317398 12.65284497 12.0590064 10.74483384 13.17570548 11.94214792 12.26912668

SHC1 13.51939054 13.81237789 14.229645 13.05069894 13.8215747 13.40221267 13.29174628 13.75143945 14.30206767 11.06406908 13.61067884 14.71623 12.22761594 12.92573986 14.24235539 12.44708323 13.54109662 14.11040174 13.11309098 14.18626903 14.25281324 13.3143 14.78866736 13.79532945 13.57423872 11.4419067 13.9235132 12.23451809 13.26751824 14.81142487 13.83713513 13.61574457 11.669328 14.25155638 14.1857272 12.53259944 12.95328707 14.80579424 12.32305476 12.94635803 13.41204051 13.95936808 12.42652685 11.97548976 13.31712993 12.97064518 13.46862404 13.33483193 13.9272224 13.9162527 14.04933807 14.06482695 13.67540578 14.23481743 14.36714169 13.61436477 13.40167953 13.40859511 13.1552926 12.28598011 13.20930105 13.81698362 12.29203399 12.35231935 13.20472396 14.06440596 14.23803148 13.29462075 11.68825031 13.57009287 14.56158629 12.47294463 13.25429051 14.49866225 13.32600808 13.23676166 14.11455573 13.36809717 13.04558904 14.03497087 15.80026798 14.81603364 13.92833331 13.98147809 14.30349538

SERPINE1 7.839203788 9.47370575 15.09967655 12.0610336 9.513727596 9.364134655 9.77478706 11.15608308 12.65463603 8.851749041 10.11113567 10.89784546 9.779719355 8.060695932 8.625708843 8.294620749 9.607330314 10.4807902 11.89557528 7.118941073 10.47065887 9.702172685 10.98013958 11.04371086 11.1382718 5.672425342 9.897845456 8.422064766 10.9076418 9.303780748 9.73470962 12.00035218 10.67154107 10.99223026 10.82892983 12.21674586 7.011227255 12.27408691 8.581200582 9.682994584 10.26209485 8.810571635 9.221587121 7.54689446 7.636624621 10.38909352 11.78953364 9.266786541 11.38801729 9.87036472 9.46760555 9.614709844 8.169925001 9.831307244 8.994353437 11.40247917 8.326429487 10.97297979 8.515699838 9.131856961 12.22430321 11.45172627 10.52061868 8.459431619 9.948367232 9.430452552 11.19414124 8.991521846 12.0664256 10.46760555 8.144658243 9.434628228 8.957102042 11.75070699 9.495855027 7.826548487 7.022367813 10.68737568 10.86186234 9.392317423 11.03204573 12.60269887 11.20518233 13.12638183 10.03891899

SDHC 11.81658371 11.2644426 12.57931594 11.5980525 12.7077905 9.342074668 11.00912879 12.50556348 12.56104923 10.49984589 11.4046094 12.43019117 12.92999806 11.08613623 11.59245704 11.16741815 11.45224124 11.33147682 12.67109873 12.87094261 12.10328781 12.86747181 13.05239823 11.79563384 12.00667672 11.6926155 11.61470984 11.39446269 12.49060021 11.65284497 12.00386923 12.56890615 10.26209485 13.16616308 12.14402087 10.96289601 10.8494051 12.35452489 10.99152185 11.35590164 11.60316268 11.88073144 11.67815996 10.22037833 12.24911345 11.30149619 11.98334993 10.92925841 12.46301341 12.35424938 11.9162527 13.24733418 10.18487534 12.12315143 13.21704891 11.78422586 11.22339841 11.36905201 11.30092449 12.05799172 11.79197682 12.04984855 12.40194612 11.03479896 11.90275194 11.59198977 12.47649308 10.52649924 11.59572369 11.53089398 12.25414285 11.46199095 11.55650605 13.5559072 12.13987106 11.30320995 11.71209705 12.24138736 11.93221475 12.90350531 13.45236995 11.40832974 12.83506349 12.63254088 12.01402047

S100B 4.321928095 4.584962501 5.906890596 2 3.906890596 2 3.459431619 3.169925001 6.022367813 4.087462841 4.523561956 3.807354922 7.076815597 3.169925001 4 4.087462841 2.584962501 3.906890596 2 5 6.285402219 5.357552005 6.614709844 2.321928095 6.475733431 2.807354922 2 5.247927513 5.781359714 1 3.906890596 8.721099189 4.64385619 4.523561956 4.857980995 3 1 3 4.906890596 3.169925001 7.400879436 2 7.033423002 2.584962501 7.118941073 4.169925001 1 2 4.857980995 4.247927513 4.321928095 5.857980995 1.584962501 5 5.426264755 3.321928095 5.857980995 4.392317423 3.169925001 1.584962501 6.06608919 6.62935662 3.321928095 1.584962501 7.247927513 4.64385619 4.523561956 2.584962501 7.044394119 3.321928095 5.044394119 3.584962501 5.95419631 5.807354922 4.247927513 3.906890596 5.491853096 6.727920455 8.098032083 2.321928095 4.95419631 4.700439718 11.88950396 4.459431619 4.754887502

RPA1 11.73935871 10.70303839 12.67904019 11.01122726 12.23092109 10.82416321 11.65687173 12.5557874 11.60130653 10.57742883 12.18208393 12.55889898 13.14815877 10.38046107 11.01680829 11.77107674 11.78463485 12.63072216 12.4395712 12.24911345 11.70433597 13.6110248 13.23391921 11.55985504 11.58965115 11.86418614 12.44630793 10.94836723 13.23571509 13.73439209 13.15671514 12.47902235 10.38370429 13.26927281 12.06474276 11.79482197 10.71080643 11.54158066 11.25679839 11.72706956 12.79786414 10.78463485 11.65284497 9.707359132 11.94690627 10.88798213 12.36796071 11.79441587 12.2182605 12.32277318 11.92109709 12.68977963 11.42416629 11.79400965 13.74178296 11.09077405 11.4152136 11.436191 11.91886324 10.98299357 12.30777003 12.25414285 12.0344551 11.72109919 10.91886324 11.76901132 12.10819772 10.32867493 11.56510208 12.29920802 11.92109709 11.65910396 12.9867309 13.1341054 12.75196241 11.59105477 12.07380685 14.53180787 12.20426546 13.13987106 13.65485975 12.33175671 13.64644621 12.77622739 12.49685378

RICTOR 8.668884984 9.645658432 11.29634268 9.45532722 9.326429487 10.203348 9.965784285 10.48381578 10.19229281 8.330916878 9.702172685 9.871905238 11.81698362 9.710806434 10.67065625 9.773139207 9.702172685 9.836050355 10.97226185 9.786269628 9.240791332 10.21188829 11.40514146 10.64565843 8.882643049 8.129283017 9.567956075 6.807354922 9.424166289 11.10787091 9.997179481 9.786269628 8.103287808 11.42574042 11.11699368 9.011227255 9.231221181 10.29806257 9.529430554 9.923327485 10.13570929 10.14210706 10.00281502 9.517669388 10.77066389 9.942514505 11.62570884 7.665335917 10.37395266 10.54785851 9.87036472 10.1959873 9.415741768 10.06608919 10.7984718 9.126704473 7.330916878 11.33594857 9.647458426 10.15228484 9.988684687 10.57175264 9.084808388 9.746514321 9.016808288 10.27961058 9.978710459 9.103287808 11.40514146 9.184875343 9.799281622 8.184875343 10.62844554 11.62890115 10.52943055 8.832890014 9.344295908 10.36084708 10.57553925 10.72962074 11.88073144 10.21067134 11.50581155 11.36850646 10.87805091

RGN 4.459431619 4.321928095 5.087462841 6.672425342 2.807354922 3 8.909893084 4.392317423 7.651051691 4.64385619 3.584962501 8.113742166 5.523561956 5.392317423 6.209453366 4.857980995 7.392317423 7.864186145 4.392317423 8.299208018 6.599912842 8.813781191 9.199672345 7.459431619 6.714245518 4.754887502 8.754887502 1 6.266786541 5.285402219 4.392317423 8.787902559 4.247927513 7.988684687 9.162391329 6.409390936 3.584962501 7.900866808 8.005624549 3.169925001 7.864186145 8.027905997 5.64385619 5.426264755 4.95419631 2 1.584962501 0 4.754887502 7.294620749 8.011227255 7.839203788 6.442943496 4.087462841 11.215533 7.768184325 3.169925001 5.614709844 1 7.266786541 8.471675214 6.95419631 6.965784285 2.584962501 5.459431619 4.247927513 4.584962501 6.475733431 3 5.392317423 7.330916878 7.400879436 4.321928095 3.321928095 7.312882955 6.392317423 5.832890014 3.169925001 7.942514505 8.17990909 5.882643049 4.700439718 9.481799432 7.434628228 4.754887502

RET 5.727920455 7.918863237 8.071462363 4.392317423 6.303780748 4.807354922 4.700439718 7.159871337 3.700439718 6.14974712 5.209453366 9.25502857 7.636624621 2.584962501 7.686500527 4.584962501 4.700439718 7.129283017 7.339850003 8.724513853 6.672425342 7.14974712 7.033423002 7.839203788 7.554588852 4.700439718 5.392317423 4 5.614709844 9.920352855 5.977279923 5.977279923 3.807354922 5.426264755 10.10852446 5.95419631 3.700439718 6.714245518 3.584962501 7.044394119 6.614709844 7.033423002 5.459431619 5.169925001 9.74819285 6.599912842 5.357552005 3.169925001 6.087462841 6.442943496 6.14974712 6.087462841 4.392317423 8.74819285 6.539158811 6.942514505 4.64385619 7.569855608 3.459431619 4.857980995 7.839203788 5.700439718 9.501837185 3.169925001 6.857980995 7.055282436 5.285402219 4.95419631 6.303780748 6.129283017 6.569855608 3.321928095 5.321928095 9.044394119 8.491853096 8.233619677 5.882643049 7.700439718 5.392317423 8 6.882643049 6.832890014 8.238404739 5.087462841 4.700439718

RELA 11.75655632 11.23302043 12.5553081 11.39016896 12.01959073 11.18797059 11.37991982 13.21750338 11.66222331 10.49884921 11.19967234 12.38505349 12.21188829 10.85564717 10.58871464 10.6891244 10.87344411 11.85408918 11.88187871 12.04882741 12.11080953 11.91699905 13.0110525 12.24584977 11.11829223 10.80976813 10.9794252 10.5943246 11.14784089 12.90782954 12.14497682 12.08547246 10.24911345 12.5512276 12.54906266 10.03891899 10.90388185 12.28077077 11.35204343 11.44035012 11.41362793 11.99823782 11.06877828 9.686500527 11.78340754 11.12541347 11.06810648 10.47775827 11.6379833 12.2737956 10.85174904 12.53794636 10.50779464 12.32530546 11.92666663 11.38424412 10.90162116 11.16490693 11.18053081 11.07881795 11.28886607 12.02721489 11.33091688 10.78708632 11.22400167 11.41256985 11.86302471 10.94251451 12.64520808 10.72366094 12.29949424 10.96938652 11.43983088 12.15323534 11.83526092 11.31684718 11.7232343 11.92740761 12.70130646 11.4003458 12.86341196 11.99612036 12.33650656 11.85564717 11.94800213

RECQL4 12.00562455 11.21249639 9.503825738 8.851749041 9.941047606 9.905387005 11.27844946 11.2911707 7.098032083 8.169925001 10.70303839 10.8008999 10.8587581 9.698704667 9.702172685 10.95855272 10.13057056 9.682994584 10.75655632 10.35204343 10.59058705 10.52552081 10.40087944 10.83762793 10.5574637 10.88722062 11.01820018 10.70822173 10.69522829 10.93442804 11.4278366 9.782998209 9.103287808 10.82575383 10.22400167 9.299208018 11.64970527 10.64024494 10.13442632 10.83841608 10.32979634 10.33985 8.479780264 7.294620749 11.09605624 10.21188829 7.442943496 11.29404631 10.56795608 11.54061241 10.9432474 11.22037833 8.86727874 8.6794801 10.32755264 10.11374217 11.28135051 9.710806434 9.853309555 12.09407769 9.126704473 11.10394343 10.28655776 10.25502857 9.06608919 11.19229281 11.15545073 8.86727874 9.95419631 10.30149619 11.47269084 10.19105921 12.39097501 12.34762137 11.02652344 11.26795708 9.698704667 12.60617384 10.84392105 11.69174352 13.06052707 10.19844504 11.59665567 10.57175264 12.15323534

RB1 10.36850646 10.62388149 11.9668659 7.491853096 10.88340699 9.645658432 9.714245518 11.35974956 11.18797059 8.842350343 10.857981 10.74651432 11.1091777 10.42206477 8.751544059 10.31854281 10.90162116 9.709083813 9.403012024 10.5372184 11.35590164 9.172427509 10.62935662 11.84549005 10.7968508 9.319672121 9.722807531 9.337621902 9.364134655 9.544964433 11.33203655 8.864186145 8.997179481 9.957102042 12.35121532 6.321928095 9.011227255 9.964340868 11.04439412 9.164906927 12.59642273 9.28077077 11.05799172 7.960001932 12.79968636 10.78463485 8.933690655 9.285402219 10.38262403 10.04848687 9.749869427 8.885696373 8.8008999 10.8864587 12.46709603 8.499845887 8.741466986 10.6128685 10.666224 10.39339046 9.579315938 10.10983065 10.58120058 9.700439718 10.98655315 11.11504365 11.48582931 7.62935662 10.75572215 11.21674586 9.758223215 10.64024494 10.02513956 12.51422091 9.2644426 9.328674927 9.537218401 8.487840034 9.533329732 7.74819285 12.45327063 11.06743436 10.73640193 9.299208018 11.67286728

RAE1 11.47370575 10.21067134 10.4807902 9.905387005 11.857981 9.321928095 10.4314976 11.41256985 10.77478706 9.938109326 10.62021983 11.17367714 12.55865987 9.379378367 8.658211483 10.71681946 10.39016896 10.95274125 10.80413102 11.11439305 11.44242519 11.58308277 11.97906787 10.83368075 11.22219114 11.5674808 10.47876962 11.18053081 11.07948478 11.00211178 11.16176174 10.42940674 9.136991112 11.71510401 11.96758653 9.118941073 10.88722062 10.45738088 9.603626345 11.07079181 11.45429929 10.36632221 9.342074668 10.3140167 10.98156728 10.80654962 10.08347933 8.77148947 10.69174352 11.68211676 10.48381578 11.69522829 9.618385502 10.35093918 12.83526092 10.04302728 10.25029842 10.2179577 10.64024494 11.83644491 11.49785184 10.53332973 11.35093918 10.86727874 10.41468524 11.45840661 11.37286506 9.144658243 10.66533592 9.903881846 10.59898297 10.66711154 10.4604559 12.12541347 11.32699117 9.519636253 11.05324713 12.67220432 10.95637616 11.31571566 12.36112133 10.203348 11.56176526 10.78217919 11.18735207

RAD52 8.139551352 8.64385619 9.957102042 6.857980995 10.56795608 7.721099189 9.607330314 8.618385502 8.417852515 6.781359714 8.682994584 9.744833837 9.903881846 7.303780748 7.46760555 8.262094845 7.768184325 8.625708843 7.569855608 8.375039431 8.584962501 9.972979786 9.221587121 8.675957033 7.139551352 7.451211112 8.636624621 7.584962501 7.539158811 8.539158811 7.754887502 8.45532722 5.930737338 10.10459875 7.960001932 7.622051819 7.577428828 8.724513853 8.108524457 9.335390355 8.816983623 7.900866808 8.098032083 6.794415866 9.17990909 7.906890596 7.971543554 7.87036472 8.271463028 8.97441459 8.927777962 9.317412614 7.994353437 8.854868383 9.350939182 8.033423002 7.988684687 8.317412614 7.357552005 7.247927513 8.479780264 7.14974712 8.129283017 8.14974712 7.129283017 9.036173613 7.375039431 6.686500527 9.079484784 7.768184325 7.569855608 7.400879436 8.375039431 9.337621902 9.73470962 8.820178962 8.396604781 9.344295908 8.076815597 8.266786541 9.654636029 7.584962501 10.01122726 8.049848549 8.54689446

RAD51 9.859534786 8.087462841 8.118941073 7.74819285 10.14465824 7.727920455 8.985841937 10.05663772 8.829722735 6.409390936 9.77148947 8.194756854 9.162391329 7.607330314 7.348728154 8.945443836 8.335390355 7.864186145 10.47876962 9.592457037 8.82336724 9.139551352 9.820178962 8.388017285 8.982993575 9.592457037 8.209453366 8.842350343 9.513727596 9.929258409 9.33315535 8.060695932 7.900866808 9.932214752 9.259743264 7.266786541 8.357552005 8.326429487 8.483815777 8.622051819 8.921840937 7.888743249 8.159871337 6.754887502 9.702172685 7.665335917 7.942514505 6.459431619 8.717676423 10.74399286 8.487840034 9.451211112 7.8008999 7.948367232 9.057991723 6.459431619 8.400879436 8.375039431 9.622051819 9.47370575 9.214319121 8.214319121 8.965784285 8.77807713 8.430452552 8.876516947 8.375039431 7.129283017 8.764871591 7.813781191 9.182394353 9.167418146 9.440869168 10.55937709 8.73470962 7.169925001 9.071462363 11.07614753 9.002815016 9.187352073 9.605479518 8.285402219 9.977279923 8.851749041 9.884170519

PYCR1 13.15655715 11.78708632 12.41494944 10.30606169 12.52061868 12.76404222 13.16663386 13.63094963 12.724727 11.87766757 12.98761931 12.6291289 12.07848442 12.24525558 12.64543328 13.02288792 11.36194377 12.83861304 14.39265283 13.30135329 11.59245704 12.45968776 14.34124082 12.71553306 12.82057788 12.04678297 11.61378946 12.43749201 11.41837972 13.20319504 12.23631323 12.29404631 11.69522829 12.68409111 11.46199095 9.509775004 11.62844554 12.65060302 13.10148331 11.88417052 11.41837972 13.28742381 10.10721708 9.810571635 12.16081685 12.15608308 11.41468524 12.04882741 12.61654884 13.77581601 12.40832974 12.96036401 11.21613956 12.89026428 12.27990072 11.94800213 12.36796071 12.10459875 12.78361217 13.32009538 12.72770778 13.16317792 12.94909716 11.54448152 12.81698362 10.81458247 12.9794252 11.37068741 10.8273427 12.20609861 12.11991379 11.93921188 11.96434087 13.41402451 13.30876563 12.50432245 12.49560523 14.96226342 12.8291281 12.81618368 14.68463906 12.78932986 12.06238349 11.89443885 13.66954946

PTPN11 11.57790084 11.86727874 13.65116384 11.53186878 12.47218312 11.41045135 12.23750875 12.58378795 12.65620138 11 11.43306377 11.84901407 13.73766986 11.19044202 10.60640521 12.44630793 11.84666568 12.80110206 11.94214792 12.70476824 11.73809226 12.69043456 13.24748253 12.4911015 12.00281502 10.62479546 12.27932039 10.45738088 12.64768327 13.54387766 12.74903138 12.8989792 10.3858624 13.29217782 11.8989792 10.68123841 11.52110964 12.3278333 11.10787091 11.90839262 12.80312207 12.13442632 12.02859678 10.04439412 13.29863541 12.01332267 11.96325736 10.34651373 13.05290763 12.87229011 11.58355293 13.06137119 11.57837269 12.31741261 13.58320032 11.75780667 10.87574935 12.16459272 12.05528244 11.52894242 12.28511319 12.21036695 11.95927751 11.87190524 12.0409746 11.38154295 12.44397954 10.04165915 11.05528244 11.357552 12.24228095 11.37177664 11.84901407 13.08248173 12.53697567 12.01541505 11.93700593 13.47801117 12.42600261 12.09836113 13.56854995 12.10754403 14.61827078 12.25738784 12.68452948

PTPN1 11.39874369 11.70043972 14.04473563 10.93369065 11.9436137 10.36413466 10.92184094 11.57742883 11.93110683 11.40194612 11.09341756 11.99964774 13.03600185 10.63117706 10.03617361 11.56367296 11.37829486 11.89064428 12.65441227 11.91849059 11.91251514 11.79075575 12.82714419 12.1382718 12.20640391 10.70822173 11.54785851 10.58025857 12.88836274 11.68387187 12.36468186 11.215533 9.799281622 13.31953101 12.38019047 10.15734694 11.37449615 11.41309898 9.939579214 11.50332886 12.33175671 11.2772874 11.08148344 9.457380879 11.98513037 11.99258434 11.74104558 12.13442632 11.95963976 12.93332182 10.87805091 12.19967234 10.52943055 11.55889898 12.5943246 11.66311331 10.60547952 11.36522885 12.16113188 12.088457 12.75697323 12.07347215 11.62296694 11.70735913 11.35369821 11.63934071 12.68693817 10.20701432 11.61240779 10.6329952 11.99152185 10.78299821 11.6635581 12.85700903 11.70649602 10.15101654 11.10459875 12.05697634 12.1382718 11.08746284 13.36112133 12.17710804 12.09209641 11.42048661 12.53454607

PTK2B 9.987264012 10.18363538 12.60964047 6.781359714 10.81378119 6.108524457 8.276124405 10.60917874 10.11634396 6.62935662 9.22881869 10.05392588 8.924812504 8.622051819 7.930737338 9.394462695 9.873444113 9.535275377 11.2366122 10.54689446 10.29576893 11.14402087 9.601770788 9.83447105 10.88111396 6.599912842 8.124121312 9.06608919 11.23242093 8.113742166 9.693486957 9.011227255 8.731319031 11.74819285 10.14338321 7.14974712 8.909893084 9.558420713 10.18487534 9.030667136 9.403012024 8.326429487 10.47065887 7.294620749 9.787902559 10.6183855 6.285402219 8.471675214 10.47167521 11.20151134 9.599912842 10.11504365 8.632995197 10.00702727 10.58120058 7.900866808 9.124121312 9.831307244 10.56510208 10.40832974 11.19905882 10.39339046 8.599912842 7.321928095 9.019590728 10.33873638 10.62844554 8.396604781 7.857980995 9.805743872 9.240791332 9.167418146 9.2644426 11.55650605 9.202123824 8.46760555 9.74819285 7.820178962 9.981567282 7.108524457 11.99964774 11.60084211 9.786269628 8.791162889 10.64745843

PTK2 11.68167766 11.64295422 11.77025093 10.76570049 12.44397954 11.06676193 11.99612036 11.93073734 11.51520703 10.59712143 12.16961188 12.16176174 13.32586758 10.75822321 11.25797706 12.04848687 11.5112585 11.91699905 11.26971112 11.77602172 11.81137469 11.6343573 13.22535809 12.22219114 11.39820926 10.79766153 12.25561875 10.27844946 11.55842071 12.77951418 12.58848041 11.41045135 10.12799432 12.1221809 12.52576548 9.499845887 11.29863541 12.62182295 11.57648435 11.94690627 11.62113611 11.31967212 11.12670447 9.700439718 12.4000789 11.04984855 11.30606169 11.07881795 11.65999589 11.95128471 12.29088282 11.78176951 10.84549005 12.43775207 12.67639789 12.0202855 10.98441846 11.62388149 10.75070699 12.86476651 11.51816136 11.45275603 11.9794252 11.79928162 11.46301341 11.74693414 12.02859678 10.63117706 11.01820018 11.40886044 13.22746553 11.51668495 13.08746284 12.51101135 12.42469119 11.87113518 10.45840661 13.61275334 11.63526466 12.8215747 13.71521128 11.59058705 14.06835844 12.60478486 12.23780747

PTGS2 6.906890596 5.700439718 8.103287808 12.98245888 5.209453366 3.169925001 4.584962501 5.584962501 7.14974712 8.764871591 8.569855608 6.599912842 8.038918989 5.95419631 7.238404739 4.392317423 5.426264755 5.614709844 5.64385619 4.95419631 7.392317423 3 11.50233458 7.247927513 6.209453366 1.584962501 7.707359132 5.129283017 7.826548487 6.303780748 7.754887502 11.33873638 2.807354922 8.375039431 5.491853096 9.451211112 5.129283017 7.857980995 5.357552005 6.06608919 5.727920455 8.768184325 5.930737338 5.727920455 8.897845456 6.700439718 5.357552005 5.727920455 6.988684687 6.807354922 7.108524457 8.375039431 8.103287808 7.700439718 7.700439718 6.95419631 6.022367813 6.426264755 7.64385619 6.459431619 6.62935662 9.948367232 5.930737338 1 8.596189756 5.129283017 5.807354922 2.807354922 4.523561956 7.924812504 4.087462841 4.087462841 7.06608919 9.405141463 6.807354922 7.531381461 4.64385619 6.022367813 11.21492619 5.554588852 6.700439718 5.247927513 12.2822197 4.523561956 8.797661526

PTEN 9.918863237 11.32530546 13.45494183 11.59851781 12.52698821 10.12541347 10.50183718 13.34651373 12.00281502 10.16239133 11.64655871 11.7077905 10.57648435 10.51471405 10.60547952 9.519636253 10.66888498 10.61930296 12.84705735 11.61010206 12.07848442 13.04780555 11.09077405 11.71037597 11.28193003 10.23122118 11.38370429 9.187352073 11.58402294 12.82674708 12.25827158 12.14146856 9.475733431 12.36495538 11.74819285 10.2467406 10.87344411 12.17211493 12.31769525 11.41468524 11.98441846 11.62250945 11.65150022 10.10590851 12.50233458 11.51520703 11.30435132 10.94763694 11.1792871 12.31288296 11.41785251 11.85720347 10.95710204 11.85720347 13.88731583 11.12541347 10.92332749 11.30320995 9.250298418 11.29059489 12.26033152 9.905387005 11.67595703 10.75070699 11.63707766 11.57506646 12.45738088 10.85953479 12.15101654 10.89102419 12.43540982 10.97226185 11.62250945 12.58308277 12.77045742 11.99859043 11.26385602 12.29863541 12.14465824 10.59152235 13.19967234 11.37721053 13.80201141 10.98513037 12.08480839

PSEN1 11.10328781 11.13442632 12.7084373 10.36303963 12.07112712 10.30378075 12.34012827 11.25797706 12.01367161 10.20945337 10.53915881 11.77272695 11.60825482 10.34540525 10.5137276 11.60037755 10.76238204 11.89557528 12.17180229 11.96866679 11.81297947 12.34983409 12.25915477 11.11504365 11.35810171 9.857980995 11.94214792 10.06069593 12.03617361 12.52135506 11.60408986 11.77766628 10.03479896 12.21886591 12.52331691 10.6110248 10.97513146 11.99859043 11.01332267 11.07146236 11.28193003 11.37395266 11.10787091 9.092757141 11.61010206 10.57931594 10.72195361 9.487840034 11.82177398 12.23332008 11.12799432 12.21735191 10.29920802 11.51076417 11.99576715 11.01402047 10.44914865 10.65553072 10.4325419 10.56985561 11.85720347 11.29059489 10.41362793 11.55170826 10.60640521 11.4236412 12.39473063 10.46658634 11.65150022 11.2848241 12.26971112 11.24019505 11.99364606 12.15987134 12.01332267 11.17554955 11.31514956 13.2038068 11.08613623 10.29576893 12.13602985 11.85174904 12.52209107 11.91214086 11.84077792

PROP1 0 0 1.584962501 1.584962501 1 2.584962501 0 1 1 1 0 4.087462841 3 0 4.700439718 2.807354922 2 0 1 0 0 1 0 0 1 0 1 0 1 0 0 0 0 0 1.584962501 4.807354922 0 2 0 1.584962501 1 1 0 3.459431619 1 0 1 0 0 1 0 0 1 1 3.169925001 0 1.584962501 1 0 4.392317423 0 0 1.584962501 0 1.584962501 1.584962501 2.584962501 0 0 2 0 0 0 1.584962501 3.169925001 0 0 1 0 1 7.247927513 1 3.584962501 0 0

PRKDC 12.00140819 14.24807581 13.48431942 13.4372319 12.88722062 14.05375622 13.8045344 14.90058382 13.8989792 12.91382439 13.54000692 14.54978467 15.93101447 12.82793808 12.95510498 14.58566677 13.71360131 14.25052049 13.12444446 14.1492708 13.41837972 15.27557893 14.61355928 13.31359165 13.45994385 12.31061278 15.32030697 12.10427113 14.08223222 15.37544676 15.1199543 14.44818085 11.93663794 15.15026296 14.01445642 12.78708632 12.33231633 13.95537747 13.28337779 13.73766986 14.83816983 13.34429591 13.37300105 11.5674808 14.39546719 12.52184578 14.27852206 12.61079417 14.53266031 13.99920729 15.24075407 14.41765476 13.31727128 14.87277106 14.57565742 14.13225872 12.27292133 13.64182597 13.88855301 14.06953368 12.99788513 13.67628769 14.28236451 14.13394491 11.83289001 13.5566258 14.4776318 12.13602985 12.1589252 13.61160121 12.90350531 13.35342254 14.30904996 15.13522831 14.5731146 14.55140787 13.14067003 15.90595104 14.4269199 14.1548972 15.75094246 13.50531536 15.24432667 14.72509994 14.46811489

PRKCD 10.13185696 10.10066234 12.23989682 7.044394119 9.968666793 7.87036472 8.977279923 10.5849625 10.09275714 7.357552005 10.28424575 10.71767642 9.749869427 8.194756854 10.45532722 10.36084708 9.324180547 9.283088353 9.971543554 10.65999589 10.77066389 10.14847658 9.939579214 9.878050913 10.27496047 8.247927513 9.810571635 9.142107057 10.87036472 7.994353437 10.34651373 8.682994584 8.826548487 10.79928162 10.72280753 7.392317423 7.417852515 10.18487534 9.754887502 9.052568051 9.154818109 8.471675214 9.971543554 7.781359714 10.10852446 10.09671515 7.515699838 9.257387843 9.627533884 10.24555271 10.33091688 10.78053977 8.523561956 9.991521846 10.36959735 9.417852515 11.23122118 9.45532722 9.665335917 9.398743692 10.48179943 9.794415866 9.463524373 9.638435914 9.4325419 10.2179577 11.00281502 7.77478706 8.787902559 9.64385619 10.91737208 9.271463028 11.07481046 11.38424412 9.62388149 9.310612782 9.874981348 9.842350343 9.797661526 8.581200582 11.03548645 9.821773982 11.91475884 8.997179481 10.15734694

PRKCA 10.74567432 10.30492167 11.99081308 10.91288934 11.49085088 12.21947107 10.66088727 10.32867493 11.77272695 9.566054038 11.17866485 12.23810614 11.04575966 10.14465824 12.7412563 9.599912842 10.42731284 11.4918531 13.17242751 11.38370429 10.51471405 11.34096276 12.25414285 12.63367641 11.47269084 10.40832974 11.78912605 8.658211483 11.02236781 13.84784036 11.49034951 12.31032876 10.15608308 11.50828999 11.05934446 10.25266543 10.04165915 12.01680829 9.667111542 11.34928123 11.63254088 10.28424575 9.700439718 11.50432245 11.41574177 9.831307244 10.38478375 8.54303182 11.72536626 11.30492167 10.50581155 12.18270471 9.868822555 10.09934781 10.48179943 11.98832965 9.419960178 10.54109662 10.27262978 9.049848549 11.38424412 10.91363743 10.43983088 10.5849625 10.37612539 9.952741247 11.35314683 10.18735207 11.72834571 10.6183855 9.405141463 9.905387005 14.53168605 10.63934071 11.16302064 10.78299821 10.56985561 13.04165915 10.99506047 9.663558104 11.75363462 11.50233458 13.5984015 12.1176431 10.29001885

PRDX1 14.50059295 12.94452906 13.42809841 12.2467406 14.4282293 11.99223026 12.63730412 12.78688219 13.39673856 11.63026713 13.074476 13.78299821 14.69152544 11.36796071 12.95201316 12.98174565 13.33776126 13.20869161 14.27605169 13.55362929 13.20609861 13.32502431 13.17586138 13.45879108 13.89548061 14.73402155 12.95328707 12.8482317 14.58865608 14.38471631 13.38140776 12.87459719 12.0768156 14.05756873 14.10876946 11.54448152 12.02202097 12.04234338 12.74041323 11.93036775 13.00369358 12.47395937 13.62308129 12.39419471 13.14847658 13.15797845 11.01471793 11.80574387 13.70660394 14.05299251 13.15939835 14.17211493 11.20089861 12.93774162 14.40600565 11.86689253 11.91886324 13.13040968 12.5574637 13.23959853 13.92916593 12.99700302 14.55919782 13.2816403 13.75593074 12.69913863 13.82187361 11.70692764 13.32951607 13.13651056 13.73343908 13.4487616 13.15608308 14.30798343 13.16757495 12.40141288 13.53101587 14.85467362 13.56343464 13.30933424 14.91592605 13.56248095 13.73978061 13.22836778 14.34207467

PPP1CA 13.53089398 12.14942959 13.24317398 10.99859043 13.58014077 11.37014248 12.02306125 13.73110685 12.60293079 10.78545247 12.12960501 12.90820495 12.51668495 12.01611184 11.5512276 12.25827158 12.05697634 12.32418055 13.24866884 13.0721326 12.546171 12.89102419 12.9199806 12.5844928 13.08281434 12.44863257 11.5844928 12.77766628 12.84450963 13.64182597 12.65910396 12.00457162 11.39016896 13.13169622 13.27029533 10.87805091 11.70692764 12.19936562 12.85116341 11.46454575 12.61240779 12.43175875 11.88874325 10.60825482 12.7368247 12.16459272 11.14529533 11.19290922 12.61907365 13.22911922 11.79603961 13.73735298 10.71166697 12.60663654 13.37856581 10.87881728 12.41969689 12.03376711 12.20518233 12.68759439 12.509775 12.95800888 12.38127256 11.98085361 12.54689446 12.53284291 12.90970561 11.4325419 13.28598011 11.7198166 13.75530489 11.78012962 12.60107434 13.81968016 12.6411486 12.357552 12.94635803 13.35851385 12.71424552 11.6891244 13.46824219 12.52991853 13.43658142 12.05968245 12.6891244

PPM1D 9.002815016 9.100662339 10.63481105 9.326429487 10.81297947 9.804131021 9.422064766 9.299208018 9.184875343 8.016808288 8.854868383 10.37177664 9.87036472 7.686500527 8.46760555 8.693486957 9.044394119 9.616548844 9.457380879 10.58871464 9.807354922 9.837627933 10.36194377 9.865733271 9.35974956 8.087462841 9.396604781 8.54689446 9.781359714 12.05188866 10.66533592 9.727920455 7.95419631 10.24079133 10.11894107 8.038918989 9.049848549 9.975847968 8.73809226 10.32305476 10.57080444 9.566054038 9.202123824 8.164906927 9.847057346 8.214319121 8.511752654 7.554588852 9.918863237 10.03754695 9 10.10590851 8.550746785 9.71596199 10.84392105 8.965784285 8.531381461 10.43358544 9.310612782 8.238404739 10.39231742 9.20701432 9.62388149 9.044394119 9.098032083 9.269126679 10.10590851 8.891783703 10.25266543 9.312882955 9.616548844 9.312882955 9.262094845 10.21188829 10.2737956 9.385862401 8.661778098 12.13538865 9.475733431 10.11634396 10.00982862 8.724513853 11.46403515 9.353146825 10.09275714

PPARGC1A 2.807354922 5.523561956 8.527477006 2.807354922 0 2.584962501 2.807354922 6.599912842 1.584962501 1.584962501 1.584962501 4.807354922 10.08878824 5.044394119 6.95419631 5.754887502 4.95419631 3 6.266786541 6.906890596 4.169925001 6.614709844 4.584962501 6.087462841 5.832890014 0 1.584962501 4.459431619 7.614709844 8.957102042 5.247927513 7.257387843 1 8.129283017 0 7.022367813 2.584962501 5.426264755 0 6.965784285 4.247927513 4.523561956 6.62935662 6.754887502 9.405141463 6.044394119 4.169925001 3 6 6.189824559 2 2.807354922 4.392317423 5.700439718 8.076815597 3.459431619 1 4.906890596 5.459431619 5.426264755 7.982993575 4 1 0 5.169925001 5.129283017 7.044394119 4.247927513 2.807354922 5.357552005 1.584962501 2 5.209453366 3.700439718 4.247927513 3.807354922 2.321928095 4.321928095 3.906890596 2 6.169925001 4.321928095 4.169925001 0 6.06608919

PPARG 8.873444113 10.96289601 9.915879379 7.539158811 10.50481899 8.214319121 8.076815597 8.124121312 9.930737338 2.584962501 9.693486957 10.24911345 10.43462823 8.876516947 6 10.5028318 10.14720492 7.930737338 8.379378367 9.607330314 10.66799854 10.39874369 7.894817763 7.876516947 9.20701432 5.857980995 8.438791853 9.499845887 9.781359714 6.686500527 10.05799172 7.238404739 5.930737338 9.355351096 11.95528665 8.405141463 7.22881869 9.756556323 7.348728154 8.092757141 9.821773982 6.584962501 8.566054038 6 6.781359714 9.453270634 6.754887502 7.64385619 9.729620744 8.903881846 8.700439718 8.285402219 8.303780748 8.214319121 10.73470962 6 11.0313566 8.951284715 8.625708843 7.864186145 10.68299458 8.169925001 7.139551352 6.285402219 8.810571635 8.77478706 9.169925001 7.569855608 10.20579325 9.709083813 10.06743436 6.569855608 6.754887502 10.22881869 8.588714636 6.50779464 8.86727874 8.820178962 4.700439718 8 11.31854281 7.794415866 8.569855608 8.257387843 9.296916207

PPARA 8.21916852 9.710806434 11.91438513 7.46760555 10.1711768 9.216745858 9.915879379 8.962896005 10.29691621 8.266786541 10.16364968 11.3426303 10.56700537 9.751544059 8.224001674 9.113742166 9.440869168 10.80976813 10.64925618 10.30149619 10 10.10328781 11.75238065 10.63481105 10.4807902 6.375039431 10.16867212 8.592457037 9.465566405 11.32080055 10.32755264 9.46760555 8.113742166 11.24317398 11.00281502 8.562242424 8.379378367 10.46352437 10.33873638 10.07815081 10.04302728 9.87036472 9.471675214 10.37395266 10.67330908 9.71596199 9.303780748 8.348728154 11.52454172 10.98085361 9.525520809 11.19905882 9.779719355 10.63026713 11.42888355 9.936637939 9.126704473 9.459431619 9.802516365 10.22641219 10.98370619 9.84862294 9.071462363 10.24436384 9.01402047 9.71596199 10.99223026 9.126704473 9.994353437 9.73809226 9.975847968 8.335390355 9.958552715 10.63390341 11.00772811 9.005624549 9.355351096 10.44501485 10.0768156 9.709083813 11.72152646 10.7984718 11.98832965 10.12670447 10.5526691

POU1F1 2 1.584962501 2 1 0 0 0 1.584962501 1.584962501 0 1.584962501 0 3.321928095 0 3 1.584962501 1 2 0 0 1 1 2.321928095 1 0 1 0 1.584962501 0 0 0 0 0 1.584962501 0 2.807354922 0 1 1 2.807354922 1.584962501 0 2 5.169925001 0 1 0 0 1.584962501 0 1 2 0 0 3.321928095 0 1 0 1 0 2.807354922 3.807354922 0 2.321928095 2.321928095 0 1 0 1.584962501 2 1.584962501 1 1 2.807354922 0 0 0 0 0 0 1.584962501 1.584962501 1.584962501 1 2

PON1 4.754887502 3.807354922 3.584962501 0 0 1 0 0 1 2.321928095 2.321928095 2.807354922 3.169925001 0 5.584962501 3.169925001 4.169925001 3.459431619 1.584962501 0 3.584962501 0 0 1.584962501 0 2.321928095 1.584962501 0 3.700439718 7.781359714 5.247927513 0 1 1 0 5.781359714 3.459431619 4.857980995 1.584962501 0 2 1 3.906890596 4.523561956 4.169925001 1.584962501 1.584962501 4.169925001 3 2 1 1 0 0 4.807354922 3 2 1 2.807354922 3.459431619 4.857980995 3 1 2.807354922 1.584962501 5.392317423 1 1.584962501 2 0 0 0 0 2 1.584962501 2.321928095 4.700439718 0 0 0 8.060695932 1 6.977279923 3.807354922 4.754887502

POLG 11.38208359 11.10983065 12.55218876 10.72024426 11.44035012 11.50878516 11.59011917 11.76155123 10.75572215 9.315149562 12.01367161 11.86069903 12.24495839 10.4918531 10.92184094 11.41626974 11.3392933 11.8077574 11.87843415 11.37014248 11.10459875 11.58543205 12.51840728 12.02583167 10.79766153 11.47623991 11.3553511 10.6329952 11.25856603 12.38262403 11.14146856 11.37883671 9.73978061 12.15608308 11.01611184 9.887220615 10.76071995 11.27786855 11.33203655 10.53138146 11.26502894 10.74146699 10.27029533 9.074141463 12.70692764 11.56414949 9.971543554 9.672425342 11.59572369 12.50457074 10.72536626 12.14879432 11.01192607 12.24525558 11.9199806 10.76735685 10.00281502 10.85486838 10.53040634 11.31797782 11.65910396 10.99010396 10.39231742 11.27612441 10.11113567 11.68474862 11.86534664 10.85720347 10.91662592 10.77971936 12.46377979 10.18735207 11.40087944 12.10131915 12.24525558 11.169925 11.17492568 12.86747181 11.25974326 12.12670447 12.38828642 10.83762793 12.39526635 11.81858218 12.51199967

POLD1 10.48381578 10.78790256 11.16050175 9.73809226 10.57837269 10.67419227 10.18858885 10.26795708 10.27029533 8.383704292 11.03823313 11.8029202 11.94471206 9.303780748 9.588714636 10.17242751 10.56414949 10.32755264 10.55170826 10.91139199 10.25029842 11.7548875 10.64295422 10.61010206 10.14847658 10.76404222 10.67507492 10.40087944 11.17679648 11.47218312 11.82654849 9.945443836 8.994353437 10.78299821 11.40779885 9.481799432 9.918863237 10.78871833 9.884170519 11.83841608 11.46862404 9.712527 9.596189756 9.511752654 10.27612441 9.957102042 9.677719642 8.77478706 10.45224124 11.24257869 9.933690655 11.46096776 9.364134655 10.42731284 11.36522885 10.20212382 11.16364968 8.933690655 9.586839788 10.24911345 10.21067134 11.436191 11.01192607 9.055282436 9.744833837 10.22037833 11.06541613 8.74819285 10.66266838 9.68474862 10.45635442 10.44501485 10.54206454 11.76901132 10.98085361 10.61930296 10.61562964 12.0385761 11.54206454 11.49884921 11.49735289 11.1176431 12.52772134 9.824958741 11.5464122

POLB 8.971543554 7.569855608 9.082149041 6.930737338 10.75739001 7.826548487 8.527477006 10.24555271 8.285402219 7.727920455 8.262094845 8.854868383 11.14911199 6.554588852 8.499845887 8.335390355 7.50779464 9.187352073 10.39446269 9.197216693 9.469641817 9.930737338 10.4604559 9.350939182 9.084808388 9.424166289 8.348728154 8.891783703 10.31174832 9.357552005 9.243173983 8.262094845 7.409390936 10 8.836050355 9.266786541 7.451211112 8.016808288 9.523561956 9.28771238 8.903881846 9.579315938 9.231221181 7.971543554 9.612868497 7.857980995 7.614709844 8.238404739 9.74819285 9.238404739 8.909893084 9.924812504 7.118941073 8.930737338 11.06608919 8.463524373 8.326429487 9.495855027 9.169925001 8.622051819 9.932214752 9.407267764 9.695228291 8.139551352 9.451211112 10.27029533 10.09539702 6.266786541 8.700439718 9.074141463 8.77478706 9.383704292 9.346513733 10.13699111 8.810571635 9.515699838 9.392317423 10.23122118 9.709083813 9.607330314 11.1401907 10.16741815 10.68299458 9.850186838 9.30833903

POLA1 9.493855449 9.366322214 10.18115226 9.813781191 10.23481743 9.586839788 10.24317398 10.01122726 10.25148241 8.888743249 9.86727874 10.59339112 10.83447105 8.417852515 9.837627933 10.14465824 9.948367232 10.35424938 9.346513733 11.54737656 9.807354922 12.08812569 10.85096815 10.09671515 9.853309555 8.247927513 9.513727596 10.31288296 10.87190524 11.13635034 11.98832965 9.695228291 8.84862294 12.06541613 11.2227949 9.584962501 10.62205182 9.731319031 9.594324604 10.25148241 12.18394547 9.62388149 9.409390936 8.550746785 10.01680829 9.019590728 9.216745858 8.603626345 10.79279029 10.82892983 10.48784003 11.06406908 9.990103964 10.19352536 11.92444139 8.353146825 8.299208018 9.124121312 9.047123912 9.930737338 10.55170826 10.64835758 10.32080055 11.53624722 9.440869168 10.1176431 9.030667136 8.640244936 10.34429591 9.523561956 11.02721489 9.301496195 10.46862404 10.28886607 10.86186234 10.17990909 9.857980995 12.52454172 10.41890673 10.55458885 12.50407411 10.12928302 11.44604941 11.32080055 9.97441459

PML 11.54882191 11.63843591 13.07948478 10.82336724 12.10819772 10.52160044 12.04371086 12.59269062 12.48331195 9.342074668 11.81177606 12.27554256 10.9248125 10.16490693 9.828136484 11.86069903 11.67639789 11.42574042 11.52649924 11.83920379 12.97387671 11.53770375 12.90651484 10.9403136 11.31174832 9.303780748 12.05358654 10.9076418 11.67330908 11.86147468 11.66666784 11.68956125 11.11699368 11.8837888 12.80231441 10.48179943 11.73343908 12.94836723 10.78135971 11.68035952 11.77478706 10.86573327 11.68387187 9.487840034 10.72280753 11.49934763 10.74735383 9.465566405 11.70390357 12.39446269 11.18611424 12.24168529 11.11829223 14.30798343 12.1692987 11.71810471 12.63117706 10.47573343 11.16741815 10.72195361 11.74819285 12.09605624 11.15860969 10.98299357 10.30263892 11.79522797 12.00140819 10.34762137 10.8494051 10.33427329 11.67330908 10.26795708 10.91513245 12.48959712 12.13955135 10.80735492 11.45018025 12.0950673 12.76466429 12.23332008 12.64745843 11.29634268 12.90444646 12.65508345 12.52796564

PMCH 3.459431619 2 3.807354922 1 0 0 1.584962501 0 0 1 0 0 0 1 0 1 1 0 1.584962501 1 1 1 2 0 0 1 0 0 3 1 0 0 1 1 0 2 0 0 0 0 2.584962501 0 1.584962501 0 2 3.700439718 2 1 2.321928095 0 0 1 0 2 0 0 0 1.584962501 1 0 1.584962501 1 0 2.807354922 3.584962501 0 2.807354922 0 3.169925001 1.584962501 0 2.584962501 0 1 2.321928095 1 2 1 1 0 2.584962501 1.584962501 1.584962501 2 0

PLCG2 9.519636253 9.784634846 11.31344994 5.857980995 10.41574177 7.247927513 8.392317423 9.060695932 9.583082768 7 8.317412614 10.70735913 9.453270634 7.8008999 7.768184325 9.172427509 10.40301202 8.62935662 9.865733271 10.23361968 10.09934781 10.03891899 9.744833837 8.519636253 9.527477006 7.076815597 9.129283017 7.95419631 10.59245704 8.430452552 8.820178962 9.818582177 7.839203788 10.15228484 12.43019117 8.550746785 8.348728154 9.005624549 9.076815597 9.033423002 7.707359132 9.924812504 9.824958741 7.894817763 8.854868383 9.319672121 7 10.89330153 9.247927513 9.847057346 9.83447105 9.727920455 7.339850003 9.428360173 10.33315535 8.710806434 8.960001932 9.513727596 8.50779464 7.14974712 9.47370575 8.813781191 9.577428828 7.159871337 8.851749041 9.513727596 9.703903573 8.016808288 7.794415866 8.784634846 10.28308835 8.519636253 8.303780748 10.64024494 9.463524373 7.876516947 9.13442632 7.266786541 8.573647187 8.422064766 10.86650621 11.26209485 9.736401931 8.396604781 10.28771238

PLAU 9.523561956 10.09671515 12.81758329 12.11731843 10.87651695 8.599912842 11.34429591 10.68387187 13.88750623 7.74819285 11.65642486 11.64970527 8.361943774 9.442943496 9.139551352 9.909893084 10.34762137 11.08480839 11.21249639 10.14593215 10.34983409 11.62205182 12.24138736 10.02513956 11.98334993 7.924812504 10.81778312 10.22158712 12.76942464 10.96722626 10.72706956 10.857981 12.69326914 11.89102419 10.70303839 10.97584797 10.52356196 13.56033283 10.71681946 9.082149041 10.2179577 8.991521846 12.29519496 7 10.38909352 11.47573343 12.84725314 9.552669098 11.62753388 11.53186878 11.15101654 13.76186284 9.893301531 8.527477006 10.15860969 12.0101784 11.29462075 10.71596199 9.977279923 9.419960178 11.62342429 13.10230382 9.131856961 12.42600261 11.37395266 10.56700537 11.62296694 9.605479518 10.99576715 9.843921051 11.98761931 11.17430154 10.24436384 12.71853288 10.61562964 8.463524373 11.02928723 12.57931594 12.45584091 7.982993575 12.08181628 9.988684687 12.02410078 12.17648485 10.87805091

PIN1 12.88931382 10.38262403 11.67860014 10.52061868 12.28626896 10.86263736 10.81618368 12.03891899 10.48784003 9.773139207 10.7984718 11.12670447 11.76071995 9.447083226 10.08878824 11.19660213 10.24792751 11.18735207 11.906139 11.10394343 10.2467406 11.45994385 12.92999806 10.15481811 10.95782756 11.54303182 10.2179577 11.72109919 11.58683979 11.18487534 10.94104761 11.34817487 10.09011242 11.51471405 11.3858624 9.326429487 11.51865316 10.35314683 10.7984718 10.17242751 11.54592977 11.75363462 10.25384748 9.28771238 10.6926155 10.56510208 10.02928723 9.440869168 10.43045255 11.60362634 10.16113188 11.77643303 9.724513853 11.56605404 12.08281434 9.529430554 11.10328781 10.4918531 10.58777752 12.40859511 10.97584797 11.59339112 11.02513956 9.124121312 11.20884399 10.73809226 11.3553511 10.23959853 11.3586512 10.12541347 11.5980525 10.39553414 10.58871464 12.39687232 12.52918651 10.8008999 11.44759986 12.29433356 11.93332182 11.02513956 11.86341196 12.04336911 11.82336724 10.09011242 11.91699905

PIK3R1 11.82813648 11.41098127 12.55410915 10.69435789 11.93147623 10.41890673 11.22400167 12.18858885 11.69435789 12.10656294 12.25650357 11.8061468 12.69021628 10.36959735 10.79441587 12.33343492 11.62935662 12.0871313 10.98797452 11.50233458 11.53576138 12.47446646 12.72344764 11.73724734 11.28424575 8.475733431 13.43606083 10.01541505 10.97656412 12.60686784 10.85174904 12.08048446 9.505811554 12.60432156 13.72557928 9.383704292 11.99612036 11.20945337 11.35204343 10.77066389 12.78626963 11.15038197 12.07881795 9.682994584 11.41574177 11.59712143 9.407267764 9.142107057 11.95746485 13.13362389 11.45840661 10.71510401 10.14974712 12.21188829 13.66544696 10.59618976 10.64385619 10.79360331 11.15734694 11.19414124 11.95201316 10.80896417 10.9708249 9.670656249 10.68562484 11.95528665 11.48582931 9.890264277 9.036173613 10.90538701 11.54399845 10.24436384 12.605248 12.51274046 11.7652861 11.04712391 10.11894107 13.94205626 11.30833903 11.24436384 13.24108938 11.59385794 13.94104761 10.76071995 12.80392929

PIK3CB 8.531381461 9.695228291 13.46058388 8.707359132 10.62479546 9.862637358 9.726218159 9.285402219 10.26561505 7.636624621 9.324180547 10.13570929 9.105908509 9.77148947 8.479780264 9.353146825 9.689997971 10.32305476 11.2997804 10.78463485 10.10459875 10.96217303 10.72280753 10.24792751 11.04439412 8.422064766 9.303780748 7.960001932 11.52894242 11.68999797 10.50581155 9.485829309 7.994353437 12.43123641 10.39874369 8.527477006 9.224001674 10.3376219 9.21916852 9.923327485 10.20089861 9.567956075 10.027906 9.162391329 10.5943246 10.44914865 9.189824559 7.768184325 10.97727992 11.44863257 9.269126679 9.977279923 9.485829309 9.50779464 10.9794252 9.37721053 7.339850003 10.35645197 10.75154406 9.73978061 11.28713519 10.65642486 9.33315535 9.266786541 10.13314221 9.882643049 11.27612441 9.192292814 8.924812504 9.700439718 9.654636029 8.95419631 10.4325419 10.67065625 10.92109709 10.44914865 9.667111542 11.73386272 9.493855449 10.66977089 11.59991284 11.11113567 11.63707766 9.539158811 10.25029842

PIK3CA 9.17990909 9.971543554 11.55889898 10.13314221 11.39874369 9.413627929 10.76570049 10.66799854 10.63571812 9.436711542 9.475733431 9.77478706 12.01262454 8.906890596 9.766528909 9.714245518 9.616548844 10.66799854 9.873444113 10.79360331 10.00140819 10.04575966 11.65999589 10.01262454 10.23361968 9.292321633 9.77478706 8.060695932 10.54012804 12.52429684 10.5028318 10.03479896 8.055282436 11.52747701 10.44501485 7.994353437 9.63481105 10.62935662 10.60917874 10.79360331 10.75822321 10.65731845 10.15608308 8.73809226 10.59618976 9.601770788 10.56033283 8.661778098 11.0202855 11.24495839 9.87958325 10.81378119 9.918863237 10.05528244 11.8989792 9.882643049 8.169925001 10.03754695 9.385862401 10.73131903 10.40726776 10.91811785 9.722807531 10.05934446 9.984418459 9.918863237 10.45738088 9.663558104 10.03066714 9.74819285 10.37503943 9.434628228 10.68299458 10.82892983 12.02928723 10.28540222 10.5849625 11.5512276 10.41151099 10.13185696 12.27961058 10.91811785 11.72238064 10.28771238 11.55842071

PEX5 10.60084211 10.1959873 11.12670447 10.41468524 13.69424905 9.355351096 10.94617524 11.74986943 10.70043972 9.396604781 9.791162889 11.18487534 11.91513245 9.970105891 8.62935662 10.13442632 9.816983623 10.60640521 11.18673329 10.85720347 10.16364968 11.13891172 11.43879185 9.862637358 10.41785251 10.52356196 10.3858624 9.850186838 10.31854281 10.28886607 9.434628228 10.85720347 8.873444113 11.85408918 10.23601419 7.820178962 7.8008999 10.38694025 10.56510208 9.845490051 9.6794801 10.54978467 10.27496047 7.727920455 10.01680829 10.15228484 10.04575966 10.58402294 10.75321675 11.15291858 10.37937837 12.37802385 9.493855449 10.77231457 11.14465824 10.38370429 10.09539702 10.28077077 10.53527538 9.899356923 10.99788513 9.868822555 10.90237511 9.792790294 9.792790294 11.23541594 10.4918531 8.21916852 10.13955135 9.266786541 9.682994584 10.2772874 10.76901132 11.18487534 11.51274046 9.505811554 9.927777962 11.57222651 10.75321675 10.30492167 12.90218666 10.33985 11.77355135 10.70043972 10.48381578

PDPK1 9.495855027 10.34651373 12.04302728 9.539158811 10.69174352 10.26678654 10.37395266 10.34540525 9.702172685 9.515699838 9.921840937 10.95346896 11.23541594 9.211888295 9.71596199 10.03204573 9.758223215 10.53915881 10.32192809 10.67595703 10.73724734 10.12023788 11.55506839 10.91064273 10.1711768 8.710806434 9.77478706 8.174925683 9.97441459 11.27146303 11.203348 9.612868497 7.906890596 11.36522885 9.948367232 8.214319121 10.15734694 10.20823436 9.306061689 10.72451385 10.73216743 9.124121312 9.942514505 8.519636253 10.41996018 10.20945337 9.240791332 9.415741768 10.77971936 10.97799537 9.808964175 10.12541347 9.493855449 10.56700537 10.65553072 9.707359132 8.499845887 10.27612441 10.01541505 9.477758266 10.73301532 9.625708843 9.62388149 9.990103964 9.836050355 9.868822555 10.82654849 9.529430554 9.659995892 9.810571635 10.027906 9.375039431 11.14274528 11.17679648 10.95492329 9.949826711 9.575539247 11.12863881 10.35314683 10.45738088 12.0115767 10.86805085 11.71080643 10.6110248 11.26091953

PDGFRB 10.83368075 14.32755264 14.53994636 13.37503943 14.54152016 11.4918531 12.7934001 15.41739105 15.49766475 11.07079181 12.99258434 13.83269226 13.21992477 13.47623991 11.45275603 14.14569337 13.46849677 15.49507427 13.25281324 12.81317994 13.78626963 12.4540422 14.32558655 13.32544601 13.68770373 9.987264012 13.73057628 11.02444712 13.25340433 11.90048948 13.480664 13.16270602 13.39914438 12.2261111 13.37272905 12.23989682 13.5112585 16.52110964 13.10197567 12.30349538 13.25103853 10.63026713 12.8027183 10.87267488 12.64317977 14.20342448 13.91008053 13.22249305 14.7016855 13.50084188 12.0764816 14.32199854 13.55110741 12.40087944 14.03221796 14.03634535 12.07481046 12.26649376 13.14306428 11.29289676 13.07948478 13.11406765 11.84117119 13.75279876 11.96866679 13.43058322 13.26385602 11.81378119 10.79441587 13.44604941 13.71166697 12.01297365 12.96181141 14.10238584 13.69696753 11.10525378 12.04882741 14.90025361 13.12428289 10.51865316 14.9657392 12.47801117 14.43090986 15.01993816 13.33007655

PDGFRA 10.48280796 12.0175044 12.35837648 13.39459667 14.71080643 11.87459719 13.33091688 13.9168125 14.14449893 7.531381461 12.24495839 13.25915477 11.88798213 12.37014248 11.36850646 13.48809118 12.36440828 13.91606605 13.66266838 12.5144675 11.22460468 13.20945337 13.11536884 13.09819661 12.26678654 9.019590728 12.33650656 9.060695932 12.2632692 12.95855272 14.0261776 12.13538865 10.63026713 14.01079033 15.17234937 9.339850003 11.7300455 13.71413817 12.14656868 12.31741261 11.64700864 9.786269628 12.34540525 10.48784003 14.02833777 11.78258876 13.14354265 12.70994538 13.38194845 15.17109859 13.07915141 13.01402047 13.22430321 10.43462823 13.98450747 13.2632692 10.32080055 13.15797845 14.47364234 13.88025314 13.5512276 15.03505681 8.794415866 13.14847658 11.712527 12.74357219 12.70994538 12.08613623 12.76217438 12.52037314 12.41071633 12.2261111 13.19982568 14.25584001 12.34429591 10.90388185 12.70108982 13.01436924 13.32460249 11.77725532 12.83011903 14.69571766 12.5844928 14.16859378 12.83387836

PDGFB 9.876516947 10.96650545 13.03032228 7.820178962 11.12734954 8.438791853 9.348728154 10.1176431 10.10197567 8.731319031 9.614709844 12.0317012 10.3586512 9.733015322 9.857980995 9.911391988 10.4314976 9.888743249 11.94544384 10.91438513 11.76818432 10.37177664 10.29920802 10.69783636 11.20762447 7.357552005 10.033423 9.236014192 10.95782756 10.91737208 10.78953364 11.05324713 9.826548487 11.68956125 10.20579325 8.092757141 10.01959073 11.76611476 10.8917837 10.47876962 9.513727596 8.027905997 10.41362793 9.199672345 10.9403136 11.73089465 8.64385619 9.479780264 10.84235034 10.94251451 10.50878516 9.837627933 9.689997971 8.997179481 10.75154406 9.37721053 10.92035286 10.93295289 10.01122726 10.05799172 11.32192809 10.6128685 9.147204925 8.285402219 10.93369065 10.75655632 11.52894242 9.355351096 9.152284842 10.95855272 11.37340896 9.52160044 10.46658634 12.24049322 9.980139578 9.211888295 10.93221475 9.447083226 10.00422047 7.499845887 11.42311591 10.62935662 11.15860969 9.361943774 11.3392933

PCNA 12.99523717 11.57648435 12.48280796 12.51693112 12.62684976 11.45738088 11.75697323 12.58120058 11.03686045 8.361943774 11.69827058 12.36604895 13.12379809 9.836050355 12.33147682 11.46607646 11.85720347 12.17679648 12.24019505 13.05629901 12.18951573 13.39016896 11.75154406 11.56795608 12.45840661 11.46199095 11.02375435 11.71938882 12.53600432 14.03694628 12.88836274 10.99929539 10.31174832 13.35672706 12.14561378 10.51865316 11.53527538 11.44966454 11.27554256 11.94727165 12.63753055 10.8008999 11.83526092 11.06137119 12.36358725 11.08148344 11.12282799 10.11894107 12.93239932 13.03187348 12.75905594 12.89235308 11.01471793 11.98690862 13.77581601 10.18858885 11.2039597 12.35782688 11.60130653 12.30092449 13.22430321 11.51175265 11.48482289 12.36249181 11.46862404 12.14625045 11.48733762 11.28135051 12.57837269 12.00597536 13.36249181 11.22158712 12.82674708 13.49585503 12.86476651 11.27670602 11.79441587 14.80211242 12.21735191 12.1221809 12.84842733 12.04780555 12.71681946 11.81297947 12.60663654

PCMT1 11.51175265 10.79603961 11.85642553 10.08480839 11.6956633 9.859534786 11.99329224 11.88988417 11.28135051 9.908392621 12.30862345 11.69653291 12.71317168 9.920352855 10.81938079 10.84313591 10.63390341 11.4767462 12.24911345 11.72664392 11.62798978 11.65239686 12.55554777 11.26091953 11.32699117 11.59712143 10.72451385 11.01122726 11.92851838 12.24198315 11.2485206 11.68299458 9.733015322 12.02894204 11.73131903 10.88340699 10.05392588 11.26795708 10.90914305 10.36303963 12.43983088 10.23840474 11.05528244 9.154818109 11.26502894 10.58965115 9.917372079 9.815383296 11.83051521 12.00947875 11.16364968 11.90651484 9.797661526 12.24911345 12.07380685 10.7984718 9.62935662 10.95710204 11.10066234 10.89254282 11.9668659 11.24079133 12.06777046 11.23002044 11.04439412 10.74483384 11.94580958 10.05528244 11.23840474 11.24198315 11.56843119 11.30149619 11.35424938 12.87113518 12.29519496 10.53430288 10.95564991 12.80150629 12.08547246 12.35810171 12.45069577 12.24703742 12.96036401 11.30149619 11.62525222

PCK1 1 0 0 4.247927513 0 0 1 2 2.584962501 1.584962501 0 1 5.169925001 1 5.64385619 0 0 2 1 0 0 1 4.807354922 0 0 0 1.584962501 2 1 2 1 2 0 2 1 3 0 0 2 2.807354922 1 2.584962501 6.599912842 1.584962501 0 0 1 0 0 1 0 0 0 4.392317423 2 0 1 0 0 1.584962501 0 0 0 0 1.584962501 3.584962501 2.807354922 0 1.584962501 1 1 0 1.584962501 1 1 1 0 2.584962501 2.807354922 1 2 0 3.807354922 0 1

PARP1 12.89671082 12.43123641 12.76321237 11.89368074 13.36877926 11.84979602 12.05866825 12.3143 12.58543205 11.63617144 12.76155123 12.60501645 14.33998915 10.55554777 11.3777528 13.12815547 12.54206454 12.35700209 12.45198378 12.82714419 13.04011845 13.50890893 12.56605404 12.45609768 12.12928302 12.15291858 12.71617641 11.24614677 13.12783315 12.82773965 12.49585503 12.7281331 10.39981196 13.54303182 13.70681975 10.78953364 11.35974956 12.00702727 11.23361968 11.34540525 13.67131991 12.70800613 12.19752388 11.66266838 12.72344764 11.85564717 11.43931146 9.930737338 13.26620091 13.36522885 12.08314687 13.66088727 11.22821744 13.53138146 13.60351044 11.01889562 12.5553081 11.53867395 12.26062556 12.06877828 12.9248125 13.17850925 12.84705735 12.14561378 11.52894242 12.40381093 13.22037833 10.89935692 12.20457114 12.13249973 13.07028869 11.75530489 12.31458324 13.69620687 13.41005379 11.19167615 12.06238349 14.42901437 13.39954497 12.99894295 13.80069771 12.4419067 14.62067804 12.58237724 13.04644195

PAPPA 4.392317423 6.918863237 9.611024797 7.011227255 8.303780748 8.625708843 7.636624621 9.509775004 9.899356923 6.044394119 9.575539247 6.06608919 8.317412614 7.539158811 9.709083813 8.483815777 7.330916878 7.562242424 8.832890014 11.21067134 10.49884921 6.357552005 9.465566405 8.515699838 5.247927513 3.321928095 9.216745858 5.321928095 10.44501485 5.044394119 10.25974326 8.027905997 4.392317423 10.95564991 5.807354922 7.366322214 7.888743249 10.32755264 5.832890014 6.539158811 8.727920455 7.276124405 8.199672345 6.832890014 9.276124405 6.918863237 9.278449458 8.499845887 7.554588852 10.11894107 8.076815597 7.169925001 5.491853096 6.14974712 7.672425342 6.247927513 10.58965115 8.731319031 6.768184325 8.495855027 6.882643049 9.350939182 4.459431619 3 8.511752654 7.055282436 10.26561505 9.022367813 4.64385619 9.252665432 10.16239133 7 11.66088727 10.00702727 6.807354922 8.413627929 7.584962501 10.88874325 6.820178962 9.041659152 10.83447105 6.169925001 8.651051691 10.29462075 10.38478375

NUDT1 12.07347215 10.26091953 10.12412131 9.902375114 12.18456545 9.199672345 11.61378946 9.497851837 10.5028318 9.381542951 12.31826034 10.87805091 9.567956075 10.83051521 10.33539035 11.71338651 10.34429591 10.68211676 12.55026581 11.07414146 9.603626345 11.48984796 12.68759439 10.69957245 11.51026967 12.9813889 10.10983065 11.83051521 10.14974712 10.22641219 9.592457037 11.19844504 10.24911345 11.23481743 12.01576349 8.791162889 10.93073734 8.721099189 11.83486604 8.266786541 10.95201316 11.11504365 9.449148645 7.46760555 10.98370619 10.32867493 7.665335917 9.68474862 11.18858885 12.18580462 11.23720996 11.84352853 9.419960178 11.21067134 10.85486838 9.055282436 10.76818432 10.16113188 10.47065887 11.47775827 11.54448152 10.81378119 10.88034881 8.912889336 10.58308277 10.73724734 10.30263892 10.71510401 9.124121312 9.14974712 12.00947875 10.29347165 11.04575966 12.89671082 11.32418055 10.35314683 11.1176431 12.62319564 10.87805091 12.14784089 12.7054164 10.84470576 10.169925 9.531381461 10.60640521

NRG1 3.169925001 4 9.022367813 3 3.906890596 3 2.321928095 2.584962501 5.781359714 4.087462841 4.64385619 4 8.383704292 3 7.761551232 1.584962501 5.584962501 4.64385619 1.584962501 3.700439718 6.727920455 3 6.977279923 7.584962501 3.321928095 1 7.523561956 4.754887502 6.247927513 8.392317423 3 2.807354922 7.417852515 3.584962501 0 5.614709844 1 7.721099189 2.807354922 8.169925001 4.754887502 0 6.189824559 6.321928095 5.392317423 5.614709844 2.321928095 5.807354922 4.523561956 5.129283017 2.584962501 6.64385619 3.700439718 3.459431619 3.906890596 8.113742166 8.566054038 6.523561956 5.832890014 5.357552005 6 4.392317423 5.882643049 5.754887502 4.807354922 10.09671515 7.312882955 1 3.807354922 4.64385619 4.321928095 2 3.807354922 8.515699838 2.584962501 1 3.584962501 2 4.321928095 1 6.714245518 2.321928095 9.407267764 10.01262454 9.481799432

NR3C1 10.67507492 11.66888498 13.24019505 12.03960452 12.3984765 10.9893945 11.42574042 12.66422504 11.6379833 7.77478706 11.2485206 11.59851781 12.83683936 10.64385619 10.13570929 12.11926538 11.78912605 12.30178196 11.24792751 11.29347165 11.23122118 12.17586138 13.3469292 12.31741261 11.58918297 9.50779464 11.36139553 9.77148947 11.48381578 11.59011917 11.4767462 12.13089227 9.77478706 12.48003281 13.15545073 10.8963324 11.32979634 11.77889848 11.97154355 11.02236781 11.74819285 10.37612539 11.45327063 10.06069593 11.72238064 11.29519496 10.34096276 9.4325419 11.47522678 12.02132703 11.40087944 11.30092449 10.74399286 12.21097568 13.00790327 10.81137469 9.584962501 10.90237511 11.73935871 9.689997971 12.04439412 10.62021983 10.75739001 11.42048661 10.75154406 11.47167521 12.18611424 10.59245704 10.8917837 10.99293834 11.93110683 10.25384748 12.26356264 12.27641524 12.07079181 10.6635581 10.42626475 13.48167332 11.43567026 10.80574387 13.21962232 11.90501086 13.06726628 11.00140819 11.95564991

NOG 5.832890014 3.169925001 7.942514505 2.807354922 7.400879436 6.754887502 5.832890014 6.044394119 4.584962501 5.459431619 10.15608308 9.802516365 8.960001932 7.451211112 8.13442632 6.930737338 7.936637939 1 3.584962501 8.060695932 6.14974712 5.832890014 9.837627933 10.19475685 5.523561956 7.491853096 4.247927513 8.87036472 3.459431619 4 9.047123912 8.243173983 5.169925001 9.177419538 11.60733031 6.321928095 8.588714636 5.247927513 7.761551232 7.475733431 8.924812504 9.763212367 6.988684687 7.076815597 8.731319031 6.977279923 0 7.294620749 6.169925001 7.417852515 8.082149041 3 5.700439718 7 7.857980995 3.700439718 7.672425342 5.357552005 5.584962501 5.807354922 5.285402219 3.906890596 8.204571144 6.700439718 8.596189756 6.392317423 10.57553925 5.781359714 2 7.900866808 8.118941073 5.247927513 8.409390936 3.169925001 8.942514505 4.64385619 8.758223215 4.584962501 8.087462841 4.392317423 9.93516505 6.672425342 5.044394119 4.392317423 7.321928095

NGFR 7.515699838 9.095397023 8.174925683 5.807354922 11.56890615 5.727920455 8.442943496 12.52454172 4.392317423 7.076815597 9.73470962 9.84862294 13.51668495 8.921840937 6.988684687 11.57412044 11.21188829 7.857980995 6.169925001 9.902375114 11.78053977 8.184875343 9.868822555 8.857980995 7.622051819 6.491853096 7.622051819 8.335390355 11.48834228 8.854868383 8.515699838 7.303780748 9.479780264 8.661778098 10.67683861 6.942514505 11.01889562 9.807354922 10.54399845 9.601770788 11.24614677 8.924812504 9.434628228 9.791162889 9.497851837 9.087462841 3.169925001 6.930737338 10.60640521 9.83447105 8.997179481 6.169925001 9.847057346 9 10.75655632 5.807354922 11.72109919 7.845490051 5.247927513 7.087462841 11.58025857 8.17990909 7.366322214 6.965784285 7.839203788 5.614709844 10.02236781 10.75905594 2.807354922 9.079484784 9.601770788 7.357552005 9.517669388 10.59152235 11.90162116 8.707359132 9.157346935 9.044394119 11.85252951 6.569855608 11.48733762 9.661778098 9.068778278 7.442943496 9.197216693

NGF 6.62935662 6.820178962 5 4.64385619 5.807354922 5.700439718 7.011227255 9.77807713 6.599912842 4.392317423 9.579315938 7.044394119 6.475733431 5.754887502 4.857980995 4.321928095 6.189824559 7.592457037 4.700439718 7.022367813 8.592457037 5.672425342 9.197216693 7.54689446 10.02928723 3 8.204571144 7.294620749 7.392317423 5.754887502 5.754887502 9.618385502 6.357552005 6.95419631 6.475733431 5.857980995 7.459431619 8.873444113 7.994353437 5.321928095 7.54689446 8.451211112 5.807354922 4.754887502 7.434628228 8.581200582 6.906890596 7.700439718 8.854868383 7.159871337 7.21916852 7.442943496 8.022367813 7.159871337 6.266786541 3.169925001 8.661778098 7.022367813 4.459431619 5.129283017 7.483815777 7.044394119 5.392317423 3.584962501 6.426264755 7.523561956 6.754887502 7.129283017 3.321928095 7.417852515 8.77478706 5.523561956 5 9.194756854 7.348728154 7.159871337 6.584962501 9.927777962 8.335390355 5.807354922 10.1959873 5.64385619 7.761551232 8.487840034 8.531381461

NFKBIA 11.36303963 11.60640521 14.1091777 13.47180221 11.39178061 10.76735685 10.63571812 11.10459875 11.28944258 9.167418146 11.59758704 11.89519657 10.70217269 10.67330908 9.779719355 11.30206767 10.6329952 11.49635449 12.38801729 11.63979289 11.74986943 12.99806149 12.33231633 11.15545073 12.39499851 9.164906927 11.32811389 10.77066389 12.53381639 10.94251451 11.12023788 12.12573633 10.14465824 13.06356361 12.58590145 9.84862294 10.77971936 11.25679839 10.59712143 10.29920802 11.05188866 10.39446269 11.67198327 9.247927513 11.26033152 12.11211366 10.37395266 10.05528244 11.74609438 12.26883437 11.32867493 13.02721489 10.57837269 11.45018025 13.33231633 11.14847658 11.02997735 10.93442804 11.13378441 12.72045804 12.54303182 11.81738343 11.04916787 9.905387005 11.14911199 11.54882191 13.15560884 10.06069593 11.61424973 11.89708913 11.64835758 10.94251451 11.30606169 12.24079133 11.36905201 11.82456103 11.65821148 11.85369942 12.37829486 11.26150731 13.90651484 11.2632692 11.93774162 11.22580994 11.75279876

NFKB2 10.34207467 10.27029533 12.74315139 9.698704667 10.83999107 7.73470962 10.05663772 10.78708632 10.68650053 8.118941073 9.972979786 11.47116713 9.821773982 9.214319121 9.383704292 8.851749041 9.531381461 9.890264277 11.45686774 10.50581155 10.4252159 10.89102419 11.35480034 10.23601419 10.34429591 9.087462841 9.057991723 9.924812504 10.85408918 10.18115226 10.31514956 9.894817763 9.33315535 11.33315535 11.15734694 9.47370575 8.876516947 10.45327063 9.714245518 9.796039609 9.681238412 10.01541505 10.56605404 8.243173983 10.34872815 11.06406908 9.074141463 10.52943055 9.958552715 11.02928723 10.26678654 10.9893945 9.121533517 10.19475685 11.27670602 10.22037833 11.24019505 9.932214752 10.1176431 9.481799432 10.93663794 11.57601187 10.07146236 9.567956075 10.25266543 10.50779464 11.83328544 9.247927513 10.62205182 9.144658243 10.74146699 9.424166289 10.91587938 11.65999589 11.19660213 9.77148947 11.15418521 10.89481776 11.98121049 10.06204614 11.84352853 11.10590851 11.68343329 9.523561956 11.39981196

NFKB1 10.22037833 10.88264305 12.39097501 11.44966454 11.04848687 9.567956075 10.76818432 10.91811785 11.42416629 8.962896005 10.2644426 11.13506795 10.4807902 9.236014192 9.184875343 10.88340699 10.509775 10.906139 10.55554777 10.77396337 11.21310422 10.71424552 12.37856581 10.39660478 11.40514146 7.930737338 11.32249154 8.503825738 11.857981 11.169925 10.8273427 11.96758653 9.77478706 11.7198166 11.07881795 8.95419631 10.48984796 11.84156435 10.31854281 9.194756854 10.15228484 10.32305476 11.03204573 7.539158811 10.13699111 10.57837269 10.99647349 10.47978026 10.73216743 11.28135051 10.71080643 12.16459272 10.30263892 10.43567026 11.75238065 10.77478706 9.782998209 9.957102042 9.909893084 9.136991112 10.86573327 11.20884399 8.491853096 11.19475685 10.18487534 10.39231742 10.77561028 8.682994584 10.54496443 9.854868383 10.906139 10.30149619 10.09803208 11.33483193 11.02583167 9.368506462 10.24792751 12.12767197 11.64880695 10.57648435 11.77930897 10.47573343 12.38747887 11.82336724 11.00912879

NFE2L2 10.77478706 11.30663136 13.0617087 11.18239435 12.72749507 10.21067134 12.02063276 11.98192399 11.66888498 9.487840034 11.81458247 12.60640521 11.72792045 10.53915881 10.78953364 10.77396337 11.08148344 11.36741475 11.01541505 12.37150441 11.8004955 11.2737956 12.92444139 12.01820018 12.21613956 10.21310422 11.05934446 9.962896005 11.67991988 12.32249154 11.6188443 11.31628153 10.00702727 12.41679753 12.21158415 9.936637939 11.31118066 11.35204343 11.37068741 11.01611184 10.92407019 11.24257869 11.37937837 9.651051691 12.07981809 10.95346896 11.26678654 9.271463028 12.27554256 11.89292222 11.47421294 11.55602699 10.35645197 12.27117119 13.46543886 10.78299821 10.28655776 11.80856203 11.50481899 10.96072599 11.68035952 11.69435789 11.04165915 12.26091953 11.17866485 11.08746284 11.68123841 9.586839788 10.41574177 11.12799432 11.6128685 10.89254282 12.30235333 12.34512799 11.8963324 10.79197682 11.22219114 12.56033283 11.58167136 11.81297947 13.00176003 12.92444139 12.99753235 11.43514934 11.6343573

NFE2L1 13.69696753 13.68288489 14.54049133 13.60848586 13.68145805 12.77499291 14.28994683 13.30648896 13.56045226 12.28308835 13.97853172 13.33692491 14.83669145 12.22370008 12.44060967 13.61010206 13.96893673 13.9275928 14.52000474 14.0221944 13.73036399 14.268542 14.91363743 13.89557528 14.01070293 12.78197437 13.92230565 12.7208855 14.04422334 15.28983879 13.9627153 14.2708793 12.88073144 13.89349115 14.84553905 11.57127862 13.44035012 13.93129155 12.78606538 12.84725314 14.23227101 13.26751824 13.5829652 11.99929539 13.76673594 13.36386098 12.81878187 13.22370008 14.61171646 14.56771846 12.5851973 14.62998265 13.02686921 14.92030633 14.65418847 13.7251532 13.1616043 12.9909903 12.85389431 13.15133372 14.4502447 14.33657629 13.50009495 13.02669633 13.66077588 13.83437229 13.71596199 13.07162995 12.4540422 12.47548013 13.19798453 13.23840474 13.56236169 14.18564978 14.09242682 12.79380649 13.16867212 15.41791843 14.48582931 13.8247599 14.63736073 14.29584067 14.87555739 13.80513926 14.09753838

NCOR2 13.33427329 13.2632692 14.878003 13.57919807 12.54327354 13.91737208 13.70390357 14.79456817 14.43430244 12.82316818 14.23937477 14.78253757 14.42331291 11.89746734 12.58824615 13.11162475 13.39754094 14.54803919 13.33511117 13.13298162 13.46607646 14.30904996 15.13606991 13.80936621 12.84077792 12.80876312 14.09027786 12.19198451 13.71756933 14.37252502 14.78443037 14.64728977 13.1374715 13.5946745 13.14942959 13.52417438 13.02807872 15.16447487 11.87651695 14.53855271 14.25096453 13.93783356 12.60871685 11.33315535 13.69620687 13.33622759 12.5228267 11.906139 14.32635926 14.13169622 12.80332392 15.2039597 13.38613194 13.48419353 12.91363743 14.65910396 13.69468435 12.75070699 13.11162475 12.42809841 13.47357892 14.14831769 12.97727992 12.57222651 12.1424262 13.5044466 13.91045536 11.99788513 12.24049322 11.73259144 13.25502857 13.080651 12.5943246 15.02401418 14.54834029 12.85564717 12.27321281 14.71730156 14.32656993 14.42081554 15.12254493 13.41494944 14.93728186 13.74220416 13.86940106

NCOR1 13.77941158 14.87627712 13.69370474 12.2511865 13.25827158 13.03514275 12.40087944 14.65222878 13.41798433 13.27321281 15.78542692 12.67330908 14.47756856 11.80735492 12.04131692 16.52659093 12.72557928 13.18192869 12.13185696 12.9275928 12.88073144 14.7557743 13.4318893 12.82057788 11.58683979 15.77525018 15.40660363 13.43931146 12.18827975 14.14879432 13.08331311 12.7412563 11.51569984 13.4603279 13.93654593 11.33147682 14.26356264 12.66910651 12.38882454 11.52747701 13.06491114 15.04814625 12.67617748 10.76652891 13.68562484 13.50183718 13.03977585 13.78053977 14.57110082 12.34706766 13.09424267 14.25944905 12.12702704 14.83999107 13.17959813 13.10033382 13.01471793 14.32586758 11.88569637 12.2261111 11.85174904 12.81157539 12.94471206 13.31132259 11.84392105 15.71083333 13.39754094 12.17773097 12.99152185 13.52906447 12.76942464 11.88455213 12.41309898 13.83081227 13.15371035 12.86244364 13.51926767 15.12214045 12.72387422 12.70908381 16.33561715 12.3984765 13.67606726 13.11748077 13.5834354

NBN 12.06743436 11.55314928 12.13859179 10.15101654 11.12734954 10.41679753 11.29462075 11.30492167 11.75822321 9.95419631 11.891404 11.46250227 13.27496047 10.43462823 9.394462695 11.942881 11.02167404 11.57080444 10.79603961 11.80896417 11.25915477 11.13249973 12.09901899 11.17305246 11.19290922 9.036173613 12.14306428 9.299208018 11.43775207 12.56033283 11.46096776 12.26532202 9.259743264 12.19813805 12.09638573 7.754887502 10.81938079 11.78381676 10.15101654 11.33091688 12.02513956 10.72621816 11.06272077 9.124121312 11.53381639 11.23361968 10.74819285 10.04575966 11.39499851 11.77684423 11.62798978 11.57601187 10.49385545 10.96144969 12.01785233 11.32136443 9.548821908 11.16490693 10.84705735 11.98370619 11.26033152 11.00211178 9.779719355 12.16364968 9.805743872 10.54689446 11.37829486 9.981567282 10.50183718 11.02721489 11.88836274 10.52061868 10.31854281 12.06844242 11.8864587 10.89860139 11.34872815 12.66733334 12.25797706 11.9068906 12.68540583 11.29174628 12.27990072 11.6635581 12.08812569

MYC 10.79603961 11.96506276 12.074476 11.93369065 11.69174352 12.24970606 11.76735685 12.15481811 11.22881869 12.24911345 11.43410693 13.13040968 12.91363743 10.69522829 9.854868383 11.9068906 10.98655315 11.7548875 11.50531536 12.73280339 11.75739001 12.90067816 14.80468564 12.29203399 11.77848786 15.77172158 11.28366717 11.43567026 11.47319838 11.35480034 12.32277318 12.3140167 9.236014192 12.04336911 11.34040649 10.50481899 10.43879185 12.77581601 10.8963324 12.24079133 12.40087944 13.00615073 10.46352437 11.19044202 12.13057056 12.11471839 11.71338651 9.554588852 11.2911707 11.82892983 11.32418055 11.87498135 11.18053081 13.82545572 12.43201985 12.43175875 11.93994645 11.31741261 10.27612441 13.99850229 11.36741475 12.21977355 14.00158412 10.78053977 13.28929847 10.7903485 11.60964047 9.839203788 10.76901132 11.72152646 13.11471839 12.67815996 12.06911406 12.51051694 13.79045032 11.81016994 11.81738343 14.31741261 14.1102386 13.1692987 13.42849104 10.52943055 13.05171876 12.47015044 11.33035672

MXI1 9.763212367 9.782998209 11.48633225 10.09275714 11 9.915879379 8.8008999 10.40726776 9.810571635 9.28771238 10.61654884 11.38424412 10.57270023 8.930737338 10.3376219 9.636624621 8.73470962 10.39553414 10.72109919 9.802516365 9.951284715 11.32699117 11.85291959 11.56652978 10.20823436 11.65865779 9.571752644 9.041659152 9.584962501 11.35259523 10.68299458 11.62662165 7.693486957 10.84077792 10.74986943 8.924812504 8.527477006 10.45018025 10.70994538 9.86727874 12.61723786 11.79603961 9.199672345 9.579315938 11.7809498 10.40194612 10.21310422 8.554588852 10.6411486 9.519636253 9.567956075 10.79441587 9.483815777 10.67771964 12.183015 8.266786541 8.813781191 10.47876962 10.30606169 9.552669098 10.35645197 9.726218159 11.9505559 8.531381461 11.02306125 10.48381578 11.36358725 8.784634846 9.670656249 9.485829309 10.7548875 9.002815016 11.1842555 11.48230378 11.74230944 10.34762137 10.46658634 11.65463603 11.30092449 10.97513146 11.25148241 9.746514321 11.89935692 8.87036472 9.923327485

MXD1 7.515699838 8.717676423 11.49485558 10.2644426 10.2911707 9.731319031 9.794415866 11.15671514 9.77148947 7.826548487 8.851749041 10.07414146 9.807354922 8.503825738 10.18115226 8.154818109 8.700439718 9.709083813 9.709083813 10.63390341 9.763212367 9.899356923 11.57742883 11.48884435 9.689997971 8.139551352 9.339850003 7.22881869 9.469641817 10.81137469 11.05663772 11.09539702 7.285402219 10.8864587 10.04984855 9.335390355 7.285402219 9.982993575 8.434628228 10.12928302 10.65194861 10.28540222 9.169925001 8.194756854 10.33091688 9.698704667 8.960001932 7.64385619 9.691743519 9.900866808 9.62935662 10.2632692 9.22881869 9.845490051 10.30149619 9.022367813 7.700439718 10.23122118 10.03479896 9.469641817 9.611024797 10.12412131 10.69696753 8.768184325 9.566054038 8.005624549 10.41151099 7.50779464 9.177419538 9.063395081 8.326429487 8.184875343 9.693486957 11.65731845 10.14593215 9.326429487 9.440869168 10.13570929 10.53527538 10.60455323 10.45532722 10.36632221 11.85720347 9.224001674 10.10852446

MTOR 11.5028318 11.5674808 12.28308835 10.4325419 12.1376316 11.76487159 11.60501645 12.033423 11.84666568 10.80896417 11.48683502 12.64678369 13.14895316 10.12928302 11.18797059 11.70951466 11.23541594 11.95637616 11.7652861 11.80009099 10.67242534 13.14082977 11.96578428 11.74483384 11.15671514 10.3553511 11.6128685 10.61010206 12.07581338 12.9909903 12.11439305 11.30492167 9.656424863 12.56176526 11.80534083 11.08812569 10.97010589 11.73724734 10.41996018 11.19414124 11.95346896 11.46301341 11.00492268 10.07547915 12.79603961 11.18115226 10.93590168 9.729620744 11.56224242 12.47116713 11.72024426 12.49235395 10.60733031 11.89671082 12.6295843 10.66977089 10.78545247 10.81778312 11.24079133 11.01959073 11.67065625 11.34207467 12.37802385 11.38370429 11.08613623 11.23002044 11.5137276 9.876516947 11.06002035 10.41679753 11.72109919 10.34540525 12.24644372 12.49984589 12.02410078 11.65284497 11.41785251 12.75905594 12.13891172 11.7198166 13.21021472 11.66489167 13.04899765 11.88874325 12.6682202

MT1E 10.92184094 8.550746785 10.87421293 10.86186234 8.864186145 8.392317423 10.4429435 10.83289001 10.29920802 5.426264755 7.383704292 11.81738343 8.951284715 8.727920455 9.733015322 9.719388821 6.266786541 11.36139553 13.0221944 12.52233632 8.654636029 11.4252159 11.17866485 11.1376316 11.05528244 8.049848549 10.5574637 12.91101741 10.52454172 11.58214198 8.906890596 10.99788513 10.07948478 10.84077792 6.22881869 9.079484784 10.31741261 12.62136509 11.18177344 5.523561956 9.422064766 10.47065887 9.719388821 8.103287808 11.05934446 7.982993575 7.813781191 10.12412131 11.47370575 10.6411486 10.37503943 12.45429929 7.77478706 12.51101135 10.59898297 11.08679969 7.417852515 7.294620749 9.696967526 11.17430154 11.1221809 11.01541505 11.13635034 11.67683861 10.09011242 8.495855027 9.924812504 8.599912842 11.87920032 8.921840937 9.294620749 11.70476824 10.64655871 9.342074668 10.02928723 10.82575383 12.73872562 9.689997971 11.95455985 11.23541594 10.80413102 10.31174832 9.726218159 10.22881869 8.640244936

MSRA 6.820178962 7.554588852 9.515699838 7.960001932 10.11894107 4.906890596 6.658211483 8.005624549 8.060695932 7.46760555 7.139551352 8.897845456 8.139551352 6.375039431 6 7.294620749 6.894817763 8.741466986 8.21916852 9.499845887 8.348728154 10.09803208 8.353146825 8.442943496 8.784634846 6.64385619 5.977279923 8.294620749 8.515699838 6.845490051 9.577428828 9.162391329 7.266786541 7.592457037 9.818582177 6.491853096 6.475733431 7.64385619 9.796039609 8.554588852 5.64385619 8.266786541 8.764871591 7.108524457 7.055282436 8.092757141 5.857980995 6.686500527 8.400879436 9.152284842 7.636624621 9.796039609 7.54689446 7.721099189 10.67507492 6.700439718 7.813781191 8.022367813 8.50779464 5.64385619 9.681238412 7.539158811 8.731319031 5.882643049 8.73470962 7.781359714 8.243173983 4.459431619 8.62935662 8.071462363 8.857980995 7.276124405 7.247927513 8.447083226 9.011227255 8.854868383 9.992938336 6.459431619 6.392317423 6.22881869 10.215533 8.194756854 10.56224242 6.614709844 7.707359132

MLH1 9.918863237 9.607330314 11.23062093 9.074141463 11.34983409 7.832890014 9.831307244 10.89026428 10.33539035 7.95419631 9.826548487 10.357552 11.40886044 8.426264755 9.805743872 9.438791853 9.638435914 10.25738784 10.62844554 10.62935662 10.15355203 11.06474276 10.13057056 9.857980995 10.28886607 8.903881846 9.129283017 8.686500527 10.83289001 11.34762137 10.23122118 9.696967526 9.09011242 11.23062093 10.62113611 8.098032083 8.903881846 9.807354922 9.204571144 9.052568051 10.34651373 8.768184325 9.917372079 8.098032083 10.59712143 10.04028972 8.982993575 8.370687407 10.64835758 10.28424575 8.696967526 10.92184094 9.071462363 9.154818109 10.89405985 9.560332834 9.262094845 9.828136484 9.936637939 8.851749041 11.10590851 9.74819285 9.992938336 10.04848687 9.668884984 9.596189756 10.66711154 7.700439718 10.38694025 9.346513733 9.799281622 9.442943496 10.69696753 11.19905882 9.829722735 9.541096615 9.459431619 11.88607758 9.881113961 10.14082977 11.58590145 9.539158811 11.33650656 10.08613623 10.36522885

MIF 13.4228532 10.67771964 12.14720492 11.01402047 12.24614677 10.89026428 11.49835061 11.8591465 11.027906 10.32642949 14.13899169 12.72131284 12.31118066 11.03066714 9.238404739 10.93590168 10.47269084 11.8761332 12.27554256 11.06002035 11.37177664 11.92221272 13.84166262 12.03307881 12.32727194 12.89652162 10.78053977 13.84038455 11.15671514 12.19690944 12.2822197 11.96470186 11.94580958 11.29576893 10.95201316 11.46505617 10.67507492 12.9895719 11.41045135 10.75655632 12.08646799 14.02063276 10.21188829 10.52356196 12.11406765 12.55242895 9.647458426 11.83130724 11.86341196 11.38963134 10.62205182 12.68343329 10.14847658 12.83506349 11.06204614 11.37991982 12.02410078 10.5849625 11.93774162 12.71080643 10.94909716 11.32642949 12.53843146 10.21067134 11.23122118 11.3140167 13.49072555 9.695228291 9.388017285 11.46352437 10.41996018 10.94104761 11.65731845 12.82137539 10.75154406 9.884170519 12.05528244 13.04046097 13.79441587 12.39606956 14.34533594 12.35590164 13.45712433 10.60917874 12.00877875

MED1 10.53915881 10.43462823 11.82972274 10.96938652 10.78626963 10.36741475 11.20762447 10.93442804 10.53527538 10.40726776 10.86186234 10.36303963 12.85272456 9.82336724 9.744833837 10.7903485 10.85642553 11.74357219 11.25974326 11.55314928 10.80251637 11.71596199 12.48280796 10.80815977 10.83447105 10.15101654 10.74146699 8.370687407 10.97369737 12.712527 11.25974326 11.8587581 9.211888295 11.88417052 11.64475759 8.861086906 10.59712143 11.56319627 10.01262454 11.27612441 11.23481743 10.169925 10.21310422 8.422064766 11.52307183 9.618385502 10.72621816 8.566054038 11.29174628 11.06541613 10.13057056 10.98156728 10.03066714 11.89481776 11.74525414 11.18797059 9.430452552 10.07280253 10.00702727 10.29462075 10.64835758 10.43462823 10.63843591 11.26737193 10.45738088 10.46352437 11.07881795 9.769837844 9.519636253 9.850186838 10.64745843 10.34540525 10.84235034 11.69174352 11.72195361 10.23242093 9.970105891 12.84705735 11.89102419 11.23421868 11.99647349 10.60733031 12.07246761 11.23780747 11.64970527

MDM2 11.17554955 10.85330956 14.19829156 10.99859043 11.8591465 10.65374078 11.27029533 11.45686774 11.16490693 9.353146825 10.52061868 10.90086681 11.98049664 10.37829486 11.05120894 10.78463485 10.74819285 11.28193003 11.67595703 12.07179752 11.11113567 11.945078 17.04320889 11.71724801 10.74399286 10.65015421 10.96794671 9.084808388 11.29174628 12.35314683 11.88187871 11.3089078 8.588714636 12.1864238 11.83526092 10.24317398 9.743151394 10.97226185 11.03686045 11.75405237 12.40726776 11.13378441 11.05596023 9.903881846 11.79441587 11.83920379 10.17741954 8.918863237 11.70692764 11.67991988 10.60547952 10.28193003 10.25029842 11.48028532 12.53454607 9.810571635 9.189824559 11.73131903 10.73724734 10.80170836 11.63026713 11.21674586 11.1382718 10.95637616 10.58777752 10.71681946 11.67639789 9.876516947 10.53332973 11.05120894 11.36358725 9.985841937 10.85096815 11.81217731 11.48028532 11.42574042 10.6635581 12.98584194 11.18673329 10.85642553 12.7054164 10.93737382 13.04899765 11.1234748 11.73470962

MAX 11.26678654 11.1382718 12.41811614 11.00492268 12.39767463 10.95637616 12.10099078 11.28771238 12.10295989 10.28771238 11.99823782 11.97333862 11.62479546 10.21916852 10.24792751 11.40194612 11.02652344 11.57601187 11.93994645 12.02271457 11.86147468 11.73597904 12.70865284 10.83368075 11.4419067 11.2772874 11.16050175 9.674192268 12.58965115 11.66222331 11.2997804 11.8917837 10.09407769 12.05120894 12.96974625 9.824958741 11.95673915 11.92295599 11.01611184 10.52356196 11.77725532 11.44656641 11.41415668 9.440869168 11.53867395 11.1401907 10.10983065 10.02513956 11.56510208 11.95528665 11.66888498 11.83249448 10.51569984 12.23631323 13.34775976 11.24495839 11.20823436 10.80413102 10.26209485 11.84313591 12.00105627 11.61148594 10.78790256 10.9068906 11.07748336 11.12412131 12.0202855 10.09803208 11.54496443 11.51422091 12.37612539 10.79603961 11.08945048 12.43593065 11.58355293 10.72536626 11.80896417 13.20043888 11.26737193 10.44811631 12.40434329 11.62250945 12.41626974 12.29318423 11.58072965

MAPT 7.022367813 5.247927513 6.285402219 4.087462841 6.339850003 4.169925001 5.672425342 6.741466986 5.321928095 4.700439718 5.247927513 4.459431619 11.35093918 4 7.434628228 7.924812504 6.523561956 8.573647187 4.523561956 4.95419631 5.584962501 10.03204573 7.813781191 6.658211483 4.857980995 5.285402219 8.379378367 2.807354922 6.321928095 4.584962501 5.426264755 8.174925683 4.459431619 6.781359714 6.794415866 7.614709844 3.321928095 7.357552005 3.807354922 7.912889336 6.700439718 2.807354922 9.656424863 7.475733431 5.129283017 5.426264755 5.754887502 3.906890596 4.247927513 5.523561956 5.209453366 5.977279923 6.022367813 9.09011242 8.276124405 6.658211483 5.584962501 4.700439718 3.321928095 6.14974712 7.383704292 8.011227255 7.54689446 4.857980995 4.321928095 5.321928095 5.727920455 4.64385619 6.539158811 4.321928095 3.906890596 5.906890596 8 4.857980995 8.164906927 4.807354922 4 5.727920455 7.622051819 7.622051819 8.483815777 7.076815597 6.965784285 6.108524457 6.303780748

MAPK9 10.10590851 9.761551232 11.29519496 9.079484784 11.60686784 8.787902559 9.54689446 10.71080643 9.988684687 9.06608919 9.992938336 10.93295289 11.29001885 9.317412614 9.499845887 10.25266543 9.893301531 9.607330314 10.28771238 10.37286506 10.12799432 10.74986943 11.50878516 10.87344411 10.92109709 8.810571635 9.656424863 9.645658432 10.48381578 11.18735207 11.04848687 10.09407769 8.813781191 10.56890615 11.12670447 9.368506462 9.686500527 10.57931594 10.21674586 9.505811554 10.54689446 9.815383296 9.990103964 8.209453366 10.62205182 9.647458426 9.584962501 8.438791853 10.19967234 10.27844946 9.99859043 10.68825031 8.885696373 10.46862404 11.59198977 8.86727874 9.025139562 9.84862294 10.23601419 10.22761594 9.964340868 10.14974712 10.74651432 9.01402047 10.27146303 10.08746284 10.60269887 8.820178962 10.39874369 9.529430554 10.97154355 9.17990909 10.82257083 10.89026428 10.8917837 8.948367232 8.982993575 12.02963233 10.40726776 10.12670447 12.44682484 9.766528909 11.56414949 10.18239435 10.39767463

MAPK8 8.962896005 9.294620749 11.13570929 10.11634396 11.03617361 9.139551352 9.575539247 11.50729912 9.344295908 8.392317423 9.727920455 10.32305476 10.68035952 9.269126679 9.781359714 9.511752654 9.943979914 10.71166697 9.607330314 10.04984855 9.674192268 10.6329952 10.70390357 9.990103964 11.11634396 8.787902559 9.586839788 10.1711768 10.12670447 11.93036775 10.92332749 10.13314221 8.758223215 10.27262978 11.2911707 8.442943496 9.290018847 10.55842071 8.95419631 10.09011242 11.01471793 10.52649924 9.769837844 8.717676423 10.96794671 9.103287808 9.310612782 8.535275377 10 10.12153352 10.32755264 10.76818432 8.744833837 9.317412614 12.00842862 9.554588852 9.301496195 9.620219826 9.651051691 10.83920379 11.509775 10.49485558 10.71767642 9.766528909 10.16867212 9.914385132 10.24079133 9.236014192 9.865733271 8.927777962 10.61746747 8.945443836 10.15987134 11.36577564 10.82336724 10.45840661 9.154818109 11.18920683 10.00281502 10.00422047 11.3777528 9.364134655 12.19290922 9.826548487 9.942514505

MAPK3 11.06272077 11.25325658 11.63617144 10.00702727 11.52012755 9.87958325 10.88111396 10.32418055 10.73470962 9.850186838 9.914385132 12.40114618 11.14529533 8.495855027 9.847057346 10.4918531 10.32530546 10.72024426 10.80493767 10.14082977 11.76071995 11.51076417 12.22671322 10.92703717 10.78217919 10.18982456 9.887220615 10.44397954 10.97727992 11.44242519 12.22972009 9.717676423 10.15608308 10.36741475 10.50481899 9.074141463 10.28655776 11.08281434 10.43045255 11.30149619 10.69435789 11.5137276 10.30263892 8.842350343 10.203348 10.26561505 8.810571635 10.40194612 10.81698362 11.08480839 10.45738088 11.19229281 9.915879379 11.19414124 11.67507492 10.10983065 11.26912668 10.12670447 10.48381578 11.88607758 11.20945337 10.62844554 10.43879185 9.405141463 10.5137276 11.3459596 11.05596023 9.008428622 9.799281622 9.691743519 11.63072216 10.32755264 10.79522797 11.66489167 11.43514934 9.539158811 10.1382718 11.20579325 10.80896417 10.45532722 12.60455323 11.68387187 12.48809118 10.35974956 11.23481743

MAPK14 10.80896417 10.99435344 12.26620091 10.49685378 11.90199819 10.70217269 11.62113611 11.51323411 11.60640521 9.991521846 11.25148241 11.15038197 12.26356264 9.826548487 10.70217269 10.19105921 11.52796564 11.60547952 11.36194377 12.03204573 11.63617144 11.96614491 12.4812949 10.70735913 10.92109709 9.787902559 11.16302064 9.622051819 11.8181827 12.94251451 11.59712143 11.33203655 9.45532722 12.16396409 11.31174832 9.47370575 10.46352437 11.39874369 10.21310422 11.46607646 11.96289601 10.50779464 11.04916787 9.105908509 12.11991379 11.01611184 11.07079181 11.63390341 11.77930897 12.00105627 11.52894242 12.12379809 10.47269084 11.39767463 12.7548875 10.64205169 10.13185696 12.24019505 10.85096815 11.02097994 11.7903485 11.39231742 11.06474276 10.80009099 10.8008999 10.8963324 12.3376219 11.02721489 10.98513037 10.61378946 11.62433855 10.37395266 10.91587938 12.25000227 12.20640391 10.90312868 10.32979634 12.69827058 11.35314683 11.46199095 12.67397152 11.70476824 12.87229011 12.23541594 11.83486604

MAP3K5 8.285402219 9.659995892 10.84313591 6.658211483 7.912889336 6.14974712 8.184875343 8.87036472 9.622051819 7.523561956 8.87958325 8.787902559 9.01402047 5.95419631 7.266786541 8.97441459 8.611024797 8.727920455 7.707359132 8.971543554 9.515699838 7.076815597 9.152284842 8.022367813 7.451211112 7.906890596 7.942514505 6.857980995 9.057991723 6.686500527 7.339850003 7.139551352 7.562242424 8.622051819 10.86650621 8.842350343 5.044394119 9.558420713 8.693486957 9.025139562 8.588714636 8.016808288 9.400879436 7.888743249 7.209453366 8.413627929 7.693486957 6.807354922 9.62935662 8.654636029 8.276124405 8.22881869 7.651051691 9.095397023 9.417852515 8.276124405 9.831307244 7.942514505 8.262094845 9.041659152 9.339850003 8.54303182 6.820178962 10.96506276 7.451211112 8.784634846 9.413627929 6.599912842 9.63481105 7.930737338 8.113742166 7.491853096 7.266786541 10.04028972 9.192292814 6.569855608 8.17990909 9.758223215 7.523561956 8.144658243 11.60917874 10.36741475 9.984418459 8.84862294 8.873444113

LRP2 3.700439718 4.807354922 3.807354922 2.321928095 5.977279923 3.321928095 3.700439718 3.906890596 1 4.169925001 2.321928095 4.321928095 7.108524457 4 7.285402219 5.459431619 5.209453366 2 2.321928095 5.247927513 4.169925001 11.59758704 3.459431619 4.392317423 5.700439718 4.64385619 3.169925001 3 4.169925001 3.459431619 3.700439718 4.247927513 3.321928095 3.700439718 3 6.554588852 5.832890014 3.700439718 3.321928095 4.392317423 9.483815777 4.247927513 1 8.519636253 2 2.321928095 2.584962501 5.087462841 4.857980995 3.459431619 3.321928095 2.584962501 3.584962501 4.087462841 5.523561956 1.584962501 1.584962501 3.584962501 2 5.285402219 3 3.807354922 2.807354922 1 5.285402219 5.321928095 3.321928095 4 2.807354922 4.64385619 3.459431619 2.584962501 3.169925001 4.321928095 6.672425342 5.426264755 4.64385619 2.584962501 2 8.22881869 5.209453366 2 5.727920455 2 4.392317423

LMNB1 11.58730873 11.20151134 10.88798213 10.62388149 12.28598011 11.56414949 10.6926155 10.82177398 11.47167521 10.19229281 11.82892983 11.68737568 13.39499851 9.77807713 10.44708323 11.81297947 12.07881795 10.72024426 11.90989308 11.94434603 10.7903485 13.18688801 12.01889562 11.47065887 12.12250448 10.36303963 10.74819285 10.62113611 11.60408986 12.93884446 12.3278333 10.70130646 9.531381461 12.27233818 12.22972009 10.72280753 10.85018684 11.7300455 11.24257869 11.68999797 13.42953751 10.38478375 11.86302471 10.59991284 12.2227949 11.28771238 10.18115226 9.584962501 11.78708632 12.41943355 10.91886324 12.33483193 10.06474276 11.40886044 12.47623991 9.766528909 9.74819285 11.03273453 11.23541594 11.21856324 11.84549005 11.36850646 11.18363538 12.49510551 10.31174832 11.10000522 11.9436137 10.11243951 11.17367714 11.72579227 12.59269062 11.60316268 13.22173815 12.7934001 11.63481105 11.42731284 11.55074679 14.26003742 11.82892983 11.9068906 11.41732512 10.86650621 13.56736196 11.33706434 12.41177578

LMNA 14.53296463 14.85379687 16.07070797 16.61585949 14.19429517 13.59816884 13.90218666 15.78920248 15.41101438 13.67518521 15.06280507 15.55281917 12.08945048 13.16081685 14.15947719 14.81277897 14.13298162 15.11097261 15.88819624 14.36036702 14.57843166 16.61565837 16.46377979 13.51840728 14.45494183 12.36905201 15.50506719 14.55248899 14.79116289 15.37021061 15.35238833 16.30518894 14.29440536 15.73288287 14.64593983 13.20242997 14.09217902 15.70690067 13.7432566 14.211356 14.54852091 15.0546891 14.26678654 12.71231204 13.75049764 14.32916565 14.10754403 13.88254753 16.15121478 15.55650605 14.55889898 16.87226606 14.691689 15.45394577 15.03531461 15.75238065 14.58848041 14.20770072 14.50928017 14.17320865 14.93488872 14.7561393 13.20502956 13.88560105 13.61470984 14.76870125 14.47294463 14.08273119 15.04435143 13.46429047 14.70482226 15.21295229 14.06995318 15.79603961 15.48633225 13.21234439 13.94462056 15.98726401 15.46336472 15.86744767 16.01963416 15.34869358 15.82060281 15.22298353 14.80498807

LEPR 7.894817763 9.758223215 11.78953364 8.475733431 9.28771238 4.247927513 6.209453366 8.092757141 8.768184325 4.087462841 9.672425342 8.951284715 9.252665432 7.839203788 7.459431619 8.139551352 9.095397023 9 9.400879436 8.417852515 10.20212382 8.751544059 8.076815597 8.438791853 9.859534786 7.622051819 6.189824559 7.607330314 9.084808388 11.74062404 9.948367232 4.64385619 7.108524457 9.594324604 8.21916852 9.044394119 8.6794801 8.784634846 7.076815597 9.139551352 10.30492167 5.044394119 8.651051691 8.199672345 9.321928095 9.294620749 7.189824559 6.129283017 9.434628228 10.47471995 6.95419631 7 8.224001674 9.269126679 10.59991284 6.539158811 5.700439718 8.936637939 9.652844973 8.184875343 10.64024494 7.700439718 6.942514505 10.51175265 7.832890014 9.036173613 10.53915881 7.592457037 9.036173613 8.596189756 10.23002044 6.700439718 8.294620749 9.616548844 7.988684687 7.741466986 7.276124405 7.761551232 6 4 10.46862404 8.092757141 9.612868497 7.442943496 10.51076417

LEP 1.584962501 2 4.906890596 1 1 0 0 2.321928095 3.459431619 0 2 1 3.700439718 0 4.700439718 3.169925001 2 2.321928095 3.700439718 1 1 1 5.554588852 4.087462841 5.044394119 1 8.174925683 2.584962501 3.169925001 2.584962501 3.321928095 12.1792871 1 2.807354922 5.321928095 6.108524457 0 3.169925001 2 1 3 2.807354922 5.087462841 5.857980995 2 5.64385619 0 2.321928095 3.459431619 2.584962501 1 6.22881869 3 4.247927513 6.22881869 1.584962501 1 4.459431619 2.584962501 3.584962501 1 3.807354922 1 1 2 4.321928095 4.95419631 1.584962501 2.807354922 2 0 0 1.584962501 5.169925001 4.459431619 1 1 3 3.807354922 2.321928095 4.087462841 1 7.209453366 1 4.321928095

KL 4.754887502 6.794415866 6.266786541 4.584962501 6.539158811 0 6.569855608 5.64385619 5.906890596 4.523561956 4.700439718 7.055282436 6.64385619 5.321928095 5.285402219 4.700439718 6.339850003 6.741466986 7.139551352 7.400879436 6.672425342 5.882643049 5.64385619 6.339850003 6.672425342 2 5.584962501 1 6.189824559 6.741466986 6.942514505 2 2.321928095 9.294620749 6.022367813 7.22881869 4.754887502 5.209453366 5.700439718 6 4.906890596 5.614709844 6.539158811 7.357552005 7.832890014 6.62935662 4.700439718 4.584962501 6.357552005 5.392317423 7.087462841 6.794415866 5.169925001 4.247927513 7.50779464 5.554588852 4.700439718 6.303780748 7.22881869 4.321928095 6.129283017 5.044394119 6.285402219 5.459431619 7.562242424 5.247927513 6.14974712 5.247927513 3.584962501 5.930737338 6.044394119 5.169925001 7.6794801 7.531381461 7.209453366 5.754887502 4.523561956 7.977279923 5.392317423 2.807354922 6.781359714 7.321928095 9.278449458 5.807354922 7.8008999

KCNA3 5.491853096 2.584962501 4.807354922 2 0 1 3.584962501 3.807354922 4.087462841 1.584962501 2 1.584962501 7.876516947 0 4.754887502 2 2.321928095 2.321928095 2.807354922 4.754887502 4.807354922 2.807354922 3.459431619 2.321928095 2 0 3.459431619 1 2.584962501 1.584962501 3.321928095 0 3.906890596 2.321928095 4.906890596 1.584962501 0 2 0 4.392317423 0 2.584962501 5.754887502 1 1.584962501 4 1 2 2 2.807354922 3.321928095 3.169925001 0 3.700439718 4.700439718 3.321928095 2.584962501 3 2.321928095 3.321928095 1.584962501 2 1.584962501 3.321928095 2.807354922 3.906890596 3.459431619 1 1 0 1 2 2.584962501 4.459431619 2.321928095 1.584962501 1 2 0 0 5.523561956 2 3.906890596 4.857980995 6.523561956

JUND 12.76570049 12.15860969 13.93562549 13.78932986 11.25325658 13.07280253 12.44423844 14.32593783 11.83762793 11.4252159 12.55865987 14.21181227 13.33035672 10.3858624 11.89368074 11.16113188 11.00632608 12.08447624 13.08131699 12.47471995 12.79644526 12.82634986 12.76570049 12.52331691 11.99576715 10.97656412 11.49485558 12.26941893 12.07881795 11.94178124 14.08464232 12.20518233 11.23900176 13.33957168 12.57601187 12.02721489 10.91811785 12.80069771 11.24317398 14.18897512 12.45763738 14.92536899 11.71424552 11.15987134 12.11309098 12.93461233 10.64475759 11.64024494 11.85720347 12.29375901 11.9248125 11.95819018 10.68035952 12.12863881 10.96361862 11.76859788 13.07112712 11.92666663 11.13314221 14.07464324 12.3984765 13.12331312 11.76859788 10.31967212 13.45776561 11.34651373 14.56146696 12.16710448 11.92666663 12.12509054 12.77663865 11.85564717 11.28944258 13.02444712 11.57837269 10.89405985 13.46199095 13.16207657 13.41071633 13.58660526 13.04302728 12.46709603 14.6084281 11.32418055 12.86399264

JUN 11.44914865 12.42652685 14.72605847 14.08986423 11.54206454 12.32080055 12.82615121 14.00650141 13.30335267 12.12799432 13.34137983 15.15208674 12.90275194 10.81217731 10.89935692 12.59082093 11.04575966 14.18464293 11.87958325 10.55362929 11.64024494 13.0871313 14.38235383 12.60686784 12.19506456 9.06608919 13.44397954 13.11569395 12.46786024 13.35548875 13.21962232 13.12928302 11.87229011 13.39164637 11.45121111 13.16050175 12.15291858 13.20319504 10.43671154 13.1492708 13.40514146 12.77045742 13.1382718 9.975847968 11.18982456 13.5849625 13.33049678 10.39016896 12.44216597 13.11569395 11.80815977 14.55973557 13.61815605 12.16867212 12.85311458 14.0664256 12.28106067 11.40141288 11.98832965 15.32863987 13.94763694 13.29347165 11.18363538 13.90961187 13.08679969 13.72462043 15.08435166 11.23302043 13.13971122 13.04319821 12.42469119 12.03823313 10.91288934 15.53147284 15.20311855 11.54496443 11.17305246 14.45936758 13.98156728 14.89992332 13.19614103 11.61424973 14.52943055 13.54339438 13.90265775

JAK2 8.854868383 8.86727874 11.97906787 8.839203788 9.074141463 9.060695932 9.381542951 8.164906927 10.25738784 7.076815597 8.262094845 9.266786541 8.640244936 10.9432474 7.74819285 8.836050355 9.324180547 9.422064766 8.312882955 10.06069593 9.049848549 8.888743249 10.18611424 8.994353437 8.164906927 5.285402219 8.714245518 6.491853096 9.554588852 10.21916852 9.247927513 8.985841937 7.569855608 10.27496047 10.25266543 8.169925001 7.912889336 10.32418055 10.04575966 8.405141463 9.417852515 8.17990909 10.31174832 8.447083226 9.691743519 8.8008999 10.89935692 6.686500527 9.627533884 10.25856603 9.656424863 9.162391329 8.640244936 11.18735207 10.04439412 8.885696373 7.159871337 9.328674927 8.696967526 8.816983623 9.177419538 10.07815081 8.588714636 9.357552005 8.744833837 8.665335917 9.451211112 7.982993575 10.25738784 8.942514505 10.77725532 8.17990909 11.03617361 9.515699838 10.56985561 8.154818109 8.573647187 9.951284715 11.34151882 8.199672345 10.38262403 11.14210706 10.857981 10.14082977 9.831307244

IRS2 6.87036472 9.033423002 11.16867212 10.80251637 6.14974712 8.321928095 7.491853096 8.611024797 8.982993575 6.832890014 7.876516947 8.794415866 10.47065887 5.247927513 5.977279923 8.965784285 7.768184325 8.971543554 7.033423002 6.569855608 7.50779464 8.503825738 7.988684687 7.658211483 7.912889336 7.95419631 7.851749041 5.584962501 7.768184325 5.426264755 9.601770788 8.64385619 6.129283017 8.118941073 9.884170519 9.71596199 9.438791853 8.569855608 7.64385619 11.92629599 6.169925001 9.479780264 8.535275377 7.011227255 5.95419631 9.06608919 6.686500527 7.584962501 7.6794801 8.744833837 8.82336724 7.523561956 6.906890596 8.577428828 9.829722735 8.807354922 6.988684687 8.092757141 5.882643049 9.027905997 10.00842862 8.388017285 6.87036472 8.303780748 6.321928095 10.09934781 9.905387005 9.002815016 9.182394353 8.654636029 8.744833837 7.977279923 5.906890596 8.54689446 10.96506276 6.189824559 6.845490051 4.169925001 8.22881869 7.614709844 10.81458247 7.658211483 10.15101654 8.942514505 8.417852515

IRS1 10.93810933 10.7548875 11.8372337 12.01192607 10.86418614 12.02997735 12.11374217 13.32403987 11.34762137 11.40087944 11.92295599 12.84058125 13.08563843 10.73047014 10.39767463 10.3858624 10.66444728 11.87459719 11.37123213 10.96361862 11.08414401 10.73386272 13.53199058 11.033423 11.47725232 9.243173983 11.31967212 8.921840937 10.68737568 13.97853172 11.54689446 10.6110248 9.233619677 11.53284291 11.28713519 9.988684687 10.75405237 10.78381676 11.16741815 10.78871833 10.39874369 12.16018658 9.839203788 9.731319031 11.51175265 10.90312868 11.19044202 8.797661526 11.3459596 11.36632221 10.73978061 10.94909716 11.03411115 13.18239435 11.92925841 10.88264305 10.80574387 11.18239435 10.31854281 10.99647349 10.96289601 11.24376903 14.00492268 9.675957033 11.17617315 11.49685378 11.8494051 10.44914865 9.813781191 10.24079133 11.35038674 10.32305476 11.41626974 12.43853198 12.74546425 11.24317398 10.027906 14.20135818 11.21249639 11.26150731 12.68846888 11.57648435 10.63481105 11.12670447 12.61079417

INSR 12.91363743 12.78422586 12.97244137 12.04371086 12.69544581 12.33873638 12.15734694 12.3834343 11.7368247 11.12412131 10.87036472 12.84156435 12.56534013 12.36249181 11.58120058 13.93119919 12.52918651 13.20487677 12.21977355 12.5943246 12.02928723 13.04797591 14.79085755 12.69108919 12.34872815 11.74357219 12.90501086 10.01541505 12.15260174 13.30035256 12.5992155 12.29605584 10.53624722 12.64024494 13.45455634 10.79360331 13.21112782 12.98584194 12.20975796 12.53915881 11.56224242 12.41679753 11.07079181 10.92332749 12.34845154 12.56009396 10.76071995 11.96470186 12.44992242 12.26414934 12.9674064 12.05052891 12.1176431 11.86534664 12.99382294 11.57695666 11.21856324 12.76611476 11.30320995 12.25089054 13.11910324 12.49909844 11.44604941 11.82813648 12.72451385 12.37068741 12.60339453 12.23391921 12.14942959 12.91326343 12.97226185 11.71639079 12.65955 13.86573327 15.69452113 12.72834571 13.24198315 13.22370008 11.83526092 12.76611476 13.40554038 11.4314976 13.11227659 11.73174329 13.50419829

INS 0 0 0 0 0 0 0 0 0 0 0 0 0 0 1 0 0 0 0 0 0 0 0 0 0 0 0 0 0 0 0 0 0 0 0 0 0 0 0 0 0 0 0 0 0 0 0 0 0 1 0 0 1.584962501 0 0 0 0 0 0 1 1 0 0 0 0 0 0 0 0 0 0 0 0 0 1 0 0 0 0 0 1 0 0 0 0

IL7R 10.41996018 10.17866485 11.00492268 6.044394119 8.28077077 6.475733431 8.912889336 6.820178962 10.08480839 6.62935662 9.766528909 8.942514505 7.562242424 9.197216693 9.025139562 11.87152026 10.99506047 7.169925001 7.942514505 9.805743872 10.60269887 9.278449458 12.91606605 10.22037833 10.13570929 4 8.523561956 7.74819285 11.5526691 12.36084708 10.86727874 9.008428622 8.22881869 12.01402047 13.52625469 9.46760555 9.116343961 7.851749041 6.988684687 7.491853096 11.7477734 5.807354922 10.10983065 5.491853096 9.103287808 10.15101654 8.118941073 9.209453366 11.28019079 10.5137276 9.424166289 8.8008999 8.247927513 9.917372079 9.285402219 8.388017285 7.651051691 11.14465824 10.99435344 11.33035672 8.252665432 12.46760555 8.912889336 4.247927513 9.544964433 9.361943774 9.843921051 6.930737338 7.011227255 8.997179481 9.513727596 8.28077077 9.799281622 11.13699111 6.807354922 7.599912842 8.379378367 8.791162889 13.42429753 7.011227255 9.805743872 10.62021983 8.375039431 11.62250945 12.95728346

IL7 6.741466986 6.754887502 8.144658243 7.622051819 8.813781191 6.794415866 5.700439718 5.554588852 7.948367232 2 6.64385619 6.087462841 6.108524457 5.700439718 5.727920455 7.129283017 8.016808288 6.459431619 7.727920455 5.832890014 5.491853096 5.129283017 7.882643049 4.584962501 8.224001674 1.584962501 7.238404739 5.285402219 8.159871337 8.64385619 4.523561956 5.285402219 5.209453366 9.192292814 5.614709844 4.700439718 3.906890596 5.285402219 3.459431619 5.426264755 8.405141463 4.087462841 7.434628228 7.118941073 7.098032083 7.658211483 4.523561956 5.392317423 6.741466986 7.971543554 8.082149041 6.965784285 5.95419631 5.459431619 7.294620749 5.64385619 6.768184325 5.285402219 6.768184325 8.77148947 8.214319121 6.965784285 2 5 5.64385619 6.95419631 7 6.266786541 5.247927513 4.459431619 5.584962501 3.906890596 3.459431619 7.754887502 2.584962501 2.584962501 3 8.016808288 5.930737338 2 9.09011242 4.392317423 5.781359714 6.794415866 7.658211483

IL6 2.584962501 2.321928095 6.832890014 9.611024797 1 0 3.584962501 4.247927513 6.768184325 3.700439718 3.459431619 6.459431619 3.906890596 1 4.087462841 0 2.584962501 2.807354922 3.807354922 3 3.700439718 2.321928095 5.700439718 1.584962501 6.321928095 0 0 1.584962501 6.189824559 1 1 4.523561956 1.584962501 5.700439718 3.321928095 9.982993575 1 4.754887502 3.906890596 1 2.584962501 1.584962501 7.592457037 2 1 6.357552005 3.169925001 4.247927513 3.321928095 2 7.813781191 6.247927513 1 4.247927513 4.087462841 0 7.554588852 4.169925001 4.169925001 4.247927513 1.584962501 8.957102042 3.459431619 1.584962501 5.285402219 3.584962501 6.06608919 0 7.285402219 2.584962501 2.321928095 2 0 7.21916852 2.807354922 1 1 3.321928095 6 1.584962501 3 1 6.977279923 4 6.247927513

IL2RG 12.02271457 8.459431619 11.1234748 4.64385619 8.113742166 4.523561956 7.434628228 7.6794801 9.17990909 5.459431619 6.584962501 9.257387843 6.794415866 7.087462841 6.741466986 7.636624621 7.483815777 6.266786541 9.696967526 9.717676423 9.541096615 8.262094845 8.214319121 7.942514505 8.651051691 5.357552005 6.569855608 7.686500527 9.77148947 6.491853096 8.076815597 7.238404739 9.820178962 9.955649908 9.436711542 7.614709844 3.700439718 8.45532722 6.375039431 8.082149041 7.159871337 5.357552005 10.88034881 5.727920455 8.939579214 8.985841937 6 6.339850003 8.794415866 8.820178962 8.22881869 8.820178962 6.14974712 8.303780748 8.714245518 6.599912842 8.463524373 8.703903573 7.87036472 8.082149041 10.16867212 8.689997971 7.665335917 9.54303182 8.592457037 8.77807713 10.60084211 5.700439718 8.807354922 7.076815597 8.271463028 7.375039431 6.977279923 9.306061689 6.442943496 6.768184325 10.44914865 6.658211483 10.12023788 2.807354922 9.579315938 7.693486957 10.70303839 8.487840034 9.917372079

IL2 2.584962501 0 1.584962501 2 0 0 0 0 1 0 1.584962501 0 2.321928095 0 1.584962501 0 2 0 0 0 0 1 1 0 0 0 0 0 0 0 0 0 0 0 0 4.321928095 0 0 0 0 1.584962501 0 1.584962501 2.321928095 3.321928095 0 0 1 0 1 0 0 0 0 2 2 0 0 0 2.584962501 0 0 0 0 2.321928095 1 1.584962501 1.584962501 0 0 0 0 1 1 1 0 1 0 0 1 1 0 0 0 0

IKBKB 9.577428828 10.07414146 11.56033283 8.044394119 10.34207467 8.903881846 9.224001674 10.92925841 9.505811554 8.118941073 10.30606169 10.59525748 10.46147945 7.832890014 9.296916207 9.566054038 9.084808388 10.2644426 10.58683979 10.15228484 10.80009099 11.41468524 11.11699368 10.97154355 9.868822555 9.612868497 9.603626345 9.019590728 10.56890615 10.25266543 10.43567026 9.20701432 8.016808288 11.08148344 10.19352536 9.48984796 9.997179481 9.491853096 10.42311591 9.997179481 10.12670447 9.52160044 10.02236781 9.68474862 10.47978026 9.815383296 8.22881869 9.796039609 10.18239435 10.89330153 10.1176431 11.10525378 8.243173983 9.157346935 11.65821148 9.618385502 9.426264755 9.908392621 10.14082977 9.539158811 10.39124359 10.00702727 10.73893668 9.603626345 10.27496047 11.5112585 10.57270023 8.375039431 9.707359132 9.804131021 10.21188829 9.681238412 11.04439412 11.21613956 10.49685378 10.51569984 10.10459875 11.00982862 10.58590145 9.06608919 12.56510208 10.45532722 11.52601011 10.23840474 10.59058705

IGFBP3 11.90538701 13.58096514 12.42731284 12.09011242 10.87190524 11.73724734 13.60188683 14.30042406 13.1049263 12.00877875 14.18208393 14.11479971 13.20838679 10.59152235 11.52356196 11.95637616 11.57553925 14.6941402 13.64419428 10.9068906 13.19414124 12.87958325 14.59927362 12.46913302 13.26620091 9.569855608 12.47065887 11.53235593 13.36016123 12.56605404 12.22941969 16.59289497 11.92369888 13.01906943 12.18704271 11.96542357 13.27525154 12.85953479 12.71360131 12.03101191 12.08878824 13.11812998 11.15038197 10.08214904 11.87113518 13.74051864 11.08679969 12.58167136 13.98388429 13.03514275 13.30620413 16.46366805 11.9690267 12.37123213 13.81708358 12.78586111 12.81016994 12.20487677 12.1091777 10.6794801 13.50506719 14.96208263 11.04848687 11.78871833 12.99488374 12.87075 11.3553511 12.32727194 13.11064643 12.68321395 12.49285462 11.33594857 11.42836017 16.12990682 14.62245226 11.56367296 13.07531201 14.06491114 16.01550217 13.06491114 13.99461861 16.04514106 14.31330822 13.69892166 13.60813929

IGFBP2 9.68474862 9.025139562 8.539158811 8.049848549 12.30178196 5.672425342 9.807354922 11.14210706 12.79907921 8 13.38572761 10.4314976 13.91429169 8.06608919 5.727920455 8.022367813 10.71080643 5.614709844 11.71553306 10.08214904 12.0202855 13.67120932 11.45224124 12.69370474 8.417852515 13.12541347 11.75863964 11.37721053 10.62296694 7.022367813 8.022367813 9.982993575 7.312882955 10.56414949 14.38815186 11.06002035 8.011227255 14.09027786 9.11113567 11.99399979 12.62067804 10.05256805 9.495855027 11.77437527 9.548821908 9.627533884 5.321928095 7.238404739 13.00263924 10.67507492 10.41679753 8.361943774 7.74819285 11.69392249 11.7903485 6.988684687 8.918863237 7.515699838 10.99223026 7.54689446 9.465566405 9.361943774 11.50034397 5.754887502 12.41890673 10.78135971 12.44708323 9.388017285 5.754887502 10.81698362 9.28077077 6.942514505 6.794415866 12.09407769 9.041659152 7.781359714 11.77848786 11.66444728 12.73872562 8.535275377 10.6891244 11.28771238 14.54574882 11.32474311 10.34207467

IGF2 14.42803296 15.17531563 11.40779885 10.64295422 11.73724734 13.52490895 13.63265447 14.15805737 12.67639789 13.56629193 16.4214896 13.83021808 19.83059722 11.91326343 10.98441846 17.80388516 13.72728233 13.77663865 11.7477734 12.62182295 14.14847658 13.16537811 12.73513288 12.83782501 15.24725999 10.92777796 12.84549005 11.29806257 14.05180371 11.45018025 14.80120313 10.29232163 15.2250191 10.29232163 10.4325419 13.55242895 13.93313737 12.64813285 11.13699111 16.88376494 12.93276839 15.24000867 12.6851868 13.25856603 14.85091933 13.27554256 7.960001932 13.50357732 13.04661247 11.6379833 14.60501645 10.28424575 12.09341756 15.48601793 14.09473751 14.82709456 19.79649913 14.48097949 7.108524457 10.70649602 14.05069894 12.37883671 9.768184325 15.22403937 11.78994113 14.06735032 16.55424408 12.92740761 14.64346165 12.07848442 14.33259606 12.20792945 12.35369821 13.43019117 15.49647933 11.45994385 13.64610864 17.21613008 11.00702727 17.20208555 17.14890353 15.40221267 18.07100663 17.35471427 13.59479112

IGF1R 11.97262086 10.63390341 11.76030413 11.51076417 11.74230944 12.94982671 11.28135051 10.82097669 11.13314221 9.271463028 11.8989792 15.72533963 14.16976845 10.14465824 11.96289601 11.21613956 11.36029843 11.61332905 11.55889898 12.22098285 11.2179577 11.40407714 12.06305796 16.17027718 10.30947635 14.83110928 11.80976813 9.703903573 10.08214904 14.27743271 11.44553222 11.44035012 9.751544059 12.40194612 12.44656641 10.38694025 9.820178962 11.29404631 10.24079133 12.62821768 11.17866485 11.49235395 11.84509796 11.61378946 13.15133372 11.63617144 12.75801496 9.390168956 12.03548645 12.28944258 11.91737208 11.53332973 11.07280253 13.64622117 11.81698362 10.99929539 9.982993575 11.45121111 9.049848549 11.24733418 12.30263892 9.733015322 16.93990055 10.57270023 11.45172627 12.78729043 12.56985561 11.14529533 10.34540525 11.70217269 11.88455213 9.977279923 13.03221796 11.52845411 12.6332223 12.9248125 10.8008999 12.68409111 9.113742166 12.72408746 13.14513609 10.47167521 11.74609438 12.48507456 12.1401907

IGF1 6.044394119 6.044394119 6.569855608 5.459431619 6.209453366 4.169925001 7.366322214 7 5.554588852 3.700439718 5.754887502 5.807354922 4.523561956 3.321928095 5.459431619 7.442943496 5.491853096 5.781359714 3 8.654636029 6.672425342 5.285402219 7.294620749 4.321928095 4.087462841 5.129283017 6.266786541 7.108524457 6.894817763 5.554588852 4.392317423 4.523561956 4.700439718 7.199672345 7.651051691 6.50779464 8.266786541 7.554588852 5.584962501 4.700439718 3.584962501 3.321928095 8.640244936 5.832890014 6.64385619 5.554588852 5.95419631 6.285402219 5.754887502 4 5.426264755 5.727920455 4.392317423 4.95419631 6.845490051 6.108524457 9.283088353 5.781359714 1 4.906890596 5.700439718 5.727920455 4.087462841 6.930737338 5.209453366 7.199672345 4.857980995 3.459431619 5.357552005 6.794415866 6.95419631 6.087462841 5.832890014 6.129283017 6.475733431 5.087462841 7 3.459431619 2.807354922 1 6.754887502 4.64385619 6.554588852 5.832890014 6.189824559

IFNB1 0 0 0 0 0 0 0 0 0 0 1 0 1 0 1.584962501 2 0 0 1 0 1 0 1.584962501 0 1 0 0 0 1 1 0 0 2.584962501 1 1.584962501 0 0 0 0 0 0 0 0 0 0 0 0 0 0 0 0 1 0 5.832890014 0 0 0 1 0 0 0 0 0 0 1.584962501 1 0 0 0 2 2.584962501 0 0 1.584962501 1.584962501 0 0 0 2.584962501 0 0 0 1 2.584962501 1

HTT 12.05866825 11.91923579 13.66466949 11.86805085 11.88340699 10.53915881 12.13987106 11.8917837 11.89671082 10.11634396 11.84627391 12.34651373 12.41626974 10.74230944 10.66799854 12.09539702 11.11504365 12.52184578 12.94873224 12.16867212 11.62159404 12.81498294 11.88683971 12.1341054 12.60455323 11.4046094 12.43514934 10.45635442 12.42101286 13.52392944 13.47471995 11.55650605 10.12412131 12.50680344 11.22460468 10.93221475 12.03204573 11.95164899 10.55554777 12.30919211 12.21431912 11.22580994 11.59851781 9.250298418 11.69435789 11.669328 11.6379833 11.15481811 12.08214904 12.91382439 11.96434087 12.84431346 11.08081753 10.81618368 13.50021946 11.07481046 11.50680344 11.80332392 11.36139553 12.39365859 12.09836113 11.57884439 11.49385545 12.2179577 11.48934624 11.39927792 12.45069577 10.88874325 11.07948478 10.77148947 11.23601419 11.02583167 11.91026796 11.83249448 11.80856203 10.46658634 11.6110248 12.18766137 10.97727992 12.16804527 13.63469763 11.78586111 14.04763517 12.56938096 12.62662165

HTRA2 10.1959873 10.18982456 10.75572215 9.257387843 11.86650621 9.995767151 10.19967234 11.20089861 10.39660478 8.751544059 9.960001932 10.83605036 11.97727992 9.417852515 9.337621902 9.741466986 9.52160044 10.36741475 10.73724734 10.41362793 10.04302728 11.26385602 11.65418847 9.493855449 9.859534786 10.28077077 9.985841937 10.39553414 10.25620869 10.91288934 10.20212382 9.995767151 8.54689446 10.68825031 10.46658634 8.754887502 8.994353437 10.28771238 10.19844504 9.074141463 10.10721708 10.12670447 9.430452552 8.54689446 10.08480839 10.04302728 8.985841937 9.949826711 10.39339046 10.73216743 10.83447105 11.47522678 8.654636029 10.20089861 11.48179943 9.975847968 9.743151394 9.714245518 10.19475685 10.32979634 10.42311591 11.04234338 10.44086917 10.215533 10.07414146 10.59712143 11.21735191 8.731319031 11.10263189 10.09934781 10.3858624 9.481799432 11.02583167 11.64250303 11.0409746 9.945443836 10.26678654 11.7300455 10.60733031 10.96722626 11.55602699 10.86727874 11.45378506 10.22881869 11.26209485

HSPD1 13.77076712 12.69892166 14.09275714 12.05968245 14.40194612 12.10427113 12.90951811 12.56486399 13.30520676 12.42836017 12.97566887 13.7522761 16.11843419 13.0175044 12.46352437 13.31868402 12.85135865 12.7198166 14.80498807 14.82227206 13.19521839 13.22596053 14.82082715 13.17648485 13.69740201 15.11549076 12.99770875 13.59933174 13.45417075 13.06524782 12.82754119 12.97692207 11.2911707 14.47313495 13.58037635 11.39016896 12.62753388 11.90726625 13.66333572 13.2485206 13.69130733 13.33455263 12.55698496 12.31146452 14.04028972 13.24525558 12.68123841 12.78463485 13.90811111 13.85389431 12.24525558 13.81928099 11.906139 14.56408993 14.00702727 11.6188443 12.25059451 13.68474862 13.57281863 13.69870467 13.48909532 13.35411161 14.51076417 12.26649376 13.82704492 12.61171646 14.18021998 10.93073734 12.24376903 13.13330279 13.25281324 12.20273604 14.36153261 14.53988579 14.31125163 12.57056729 14.14051027 14.77740944 14.20495316 12.23601419 14.40347811 13.55973557 14.48287097 13.25148241 13.49297976

HSPA9 12.96054501 12.77704979 14.32122348 12.9432474 13.48909532 12.01436924 12.60060985 14.33713405 13.30848124 12.6411486 12.51051694 12.46862404 15.42134166 12.22339841 11.8917837 13.9894832 13.38801729 13.06844242 14.15149228 13.36481862 12.53430288 13.9893945 14.62393863 13.91503906 13.49160261 13.66400276 12.76030413 12.24495839 13.53418127 13.34637522 12.56271943 13.66200072 11.31684718 13.85291959 13.95328707 11.00070427 11.81498294 12.67771964 13.16976845 11.56890615 13.47116713 11.83328544 12.52649924 11.44914865 13.15734694 12.92035286 11.90048948 10.74986943 12.94708898 13.72120602 12.41758884 13.59537405 11.64700864 13.73481545 14.40886044 12.23092109 12.04200131 12.43905168 12.91811785 12.23391921 13.30206767 12.6359448 13.06120241 13.21280033 13.07313738 12.45738088 13.63515127 11.5674808 12.04302728 12.43097517 13.603974 12.62067804 13.91513245 14.67165163 12.82137539 12.53649007 12.62981194 15.03183041 13.67386113 13.02635053 14.69740201 12.86457308 13.58049413 13.39954497 13.66077588

HSPA8 15.10623576 15.03840463 16.3508874 13.26868819 16.27546981 14.30092449 15.01266818 14.7190679 15.5635538 13.56057167 14.42160465 15.48815396 16.26673165 14.99147756 14.60136457 15.62719187 15.49210354 15.30417304 15.81848232 16.60584119 15.78825952 16.36053849 15.76945047 15.87661287 16.21025278 16.24278335 15.35314683 13.5980525 16.49266689 16.59114246 15.86672353 15.29228568 14.31139356 16.59949157 15.17023805 13.58848041 13.32867493 15.40480895 14.85744649 15.18169581 15.31886052 14.70514637 15.12088584 13.88445674 15.89495983 14.63049466 14.23399408 14.57370635 16.30730756 15.90573955 15.09098075 16.06890421 14.15979252 16.10371809 16.62924277 14.5535693 13.70638809 15.51209229 15.05862598 14.44985795 15.64185419 15.40879411 16.06653071 15.29816999 15.3098671 14.69951823 15.74727515 13.23302043 15.13422575 14.83229668 15.11918431 14.67082219 16.0159159 16.48195706 15.76867541 15.11105414 13.43097517 16.87724818 15.7345244 15.3974741 17.28489638 15.80332392 16.49163392 15.61519856 16.04929552

HSPA1B 12.83743083 10.65999589 10.83289001 11.91401133 9.505811554 10.2644426 10.36194377 10.28771238 10.87958325 9.533329732 11.20212382 11.78422586 13.56116859 7.994353437 12.74672424 9.911391988 9.411510988 11.17492568 14.53180787 9.658211483 11.16364968 10.357552 12.78729043 12.72792045 10.21431912 13.11064643 10.90989308 10.96000193 10.86805085 11.66711154 12.40939094 10.93884446 9.259743264 10.93369065 9.240791332 9.569855608 11.93295289 11.9918761 8.060695932 12.48658366 11.30777003 10.06204614 10.49984589 9.786269628 10.20701432 11.09341756 8.807354922 7.665335917 11.25620869 11.14465824 10.34096276 12.60802375 9.375039431 12.10033382 12.01052811 9.35974956 10.76321237 11.10000522 10.11634396 11.05460432 11.54930337 10.23720996 13.53284291 9.592457037 12.76756377 12.38559281 14.79735756 8.857980995 10.29576893 9.894817763 10.5943246 9.539158811 10.70476824 13.59642273 13.09110476 10.78463485 8.519636253 12.53211237 11.36577564 11.8317031 11.50729912 10.14847658 13.40500847 10.94397991 11.89254282

HSPA1A 13.41626974 11.36796071 11.22098285 11.88607758 10.04028972 10.61562964 10.28308835 10.13955135 11.25207404 9.995767151 11.17741954 12.08945048 10.98299357 8.73809226 13.05765334 10.19229281 10.01541505 11.26971112 14.5488821 10.23242093 10.97513146 10.83605036 12.77766628 12.8474489 10.34762137 11.93295289 11.08613623 10.76570049 10.47573343 11.09737377 11.66666784 10.83683936 9.269126679 10.87190524 10.10066234 9.392317423 12.33063682 11.35810171 8.622051819 6.894817763 11.5863707 10.4419067 11.05934446 10.10721708 10.9505559 11.169925 9.124121312 8.209453366 11.12153352 11.16804527 10.20212382 12.87036472 9.796039609 11.47370575 11.28250931 9.364134655 10.73470962 11.71810471 10.65731845 11.994707 11.17367714 10.60917874 13.12137163 9.729620744 12.69653291 12.4540422 13.52894242 9.77807713 10.9248125 10.53624722 10.666224 9.573647187 10.4604559 13.44060967 13.0891194 10.60269887 10.33315535 12.10950422 11.33427329 11.8963324 11.87267488 10.66444728 13.43918158 11.25797706 12.23092109

HSP90AA1 15.55904841 15.00785949 16.34144933 15.4604239 15.98339447 14.57281863 15.80576906 14.55116751 15.94859537 15.86203191 14.45249866 15.59385794 19.06425069 14.51594618 15.63237047 16.35197444 15.38747887 15.75104711 16.56458121 15.9836394 14.98330539 16.93576359 15.94542097 15.95094313 15.58631205 16.54342459 17.55120507 14.50983685 15.94866381 16.82920244 15.24926163 15.80715364 13.67727919 16.50774819 14.9744594 14.66016307 15.56000437 15.50034397 14.84441155 16.22883748 16.24295078 14.90703148 15.29001885 14.74030781 16.8352856 14.98704191 14.11853558 14.24903936 16.13018844 15.89360964 14.55446894 17.54303182 14.3177659 15.33500646 15.8369626 15.37537888 13.49822593 15.51489894 15.72456714 15.07455962 15.81870699 15.52150843 15.65918761 15.91394123 15.19036485 15.10807518 16.72371427 14.11634396 15.98728622 15.92624966 16.08333389 15.79880085 15.74942428 16.53579175 16.46197497 15.44015543 16.02093655 17.8473082 15.72991278 16.35262971 17.18541749 15.25303493 17.11393543 15.81723351 16.22390743

HSF1 13.13570929 12.7652861 12.49685378 11.00842862 11.98156728 11.40673648 12.06877828 12.98637538 12.27670602 9.479780264 11.21674586 13.38720958 13.4372319 11.1692987 10.99364606 12.56795608 11.81418188 11.71510401 12.76134346 11.46454575 12.49934763 12.15766273 13.7077905 12.21886591 11.65508345 12.2179577 12.01889562 11.97333862 12.01402047 12.39606956 12.57577558 11.38532318 10.90312868 12.03514275 12.63571812 10.14465824 11.43358544 13.01140199 11.94141447 12.17866485 12.2273151 12.0664256 10.66444728 10.19721669 11.87498135 12.01576349 8.933690655 12.033423 11.99116751 12.46913302 12.06204614 12.8474489 10.34762137 11.4655664 11.43306377 12.13859179 12.52796564 11.22761594 11.15608308 13.21598794 11.28886607 12.17023805 11.34096276 11.19660213 11.82416321 11.84666568 13.05069894 10.9708249 10.66888498 11.16427844 12.59479112 11.68167766 12.87344411 13.00544911 12.69631556 12.04302728 11.70476824 13.60188683 12.47649308 12.34817487 13.79116289 11.79968636 13.56450678 12.52503135 12.69913863

HRAS 13.05290763 10.09671515 10.78463485 9.103287808 12.12315143 9.862637358 10.10459875 10.86418614 11.02167404 8.721099189 10.36084708 10.31741261 10.92851838 8.781359714 9.240791332 10.30606169 9.252665432 10.58965115 12.1091777 11.44759986 10.37829486 11.29001885 10.96578428 10.28077077 10.87113518 11.14656868 9.627533884 12.17492568 10.99788513 11.03479896 9.807354922 8.741466986 8.84862294 11.02859678 9.4325419 8.813781191 11.19537221 9.978710459 9.661778098 9.335390355 11.17180229 10.68999797 9.159871337 8.611024797 10.53624722 9.726218159 8.519636253 10.27029533 10.73131903 12.01715639 9.548821908 12.15006458 8.968666793 10.70822173 10.33539035 10.25738784 10.51274046 10.3586512 9.428360173 10.85720347 10.84784036 10.89784546 10.25384748 9.350939182 10.88569637 11.98156728 10.75238065 9.428360173 10.20089861 10.01541505 11.69653291 10.12670447 10.65731845 11.48230378 10.74903138 9.636624621 11.29289676 12.65999589 10.72877085 10.62113611 11.98370619 10.02652344 10.33985 10 11.10590851

HOXC4 9.499845887 7.055282436 8.290018847 7.321928095 6.807354922 9.379378367 8.276124405 9.693486957 8.741466986 8.842350343 9.139551352 9.388017285 10.15608308 8.511752654 8.816983623 9.194756854 9.184875343 8.566054038 10.45327063 9.068778278 8.885696373 8.353146825 10.61378946 10.13570929 8.139551352 9.192292814 7.139551352 7.409390936 7.199672345 9.182394353 9.529430554 8.654636029 7.17990909 8.857980995 9.807354922 5.95419631 9.447083226 8.826548487 9.670656249 9.428360173 10.18735207 9.041659152 8.558420713 6.266786541 9.888743249 8.82336724 8.491853096 9.319672121 8.764871591 11.29863541 9.457380879 8.97441459 7.499845887 9.721099189 10.55458885 8.405141463 5.727920455 6.584962501 6.741466986 8.991521846 9.967226259 9.605479518 9.952741247 6.14974712 9.271463028 9.084808388 8.703903573 6.584962501 5.392317423 8.957102042 9.509775004 7.599912842 8.413627929 8.768184325 9.411510988 9.392317423 9.977279923 10.43462823 6.62935662 9.405141463 9.927777962 8.413627929 10.45635442 8.813781191 9.527477006

HOXB7 9.495855027 9.33315535 9.965784285 4.807354922 10.39767463 7.417852515 6.475733431 10.32192809 8.566054038 7.87036472 9.854868383 9.285402219 10.36413466 8.299208018 9.398743692 10.11504365 9.537218401 7.539158811 10.28540222 9.941047606 9.194756854 7.46760555 9.957102042 10.38046107 9.87036472 9.438791853 10.6110248 9.342074668 9.337621902 10.4918531 9.649256178 9.226412193 8.28077077 10.033423 10.39874369 8.654636029 7.366322214 10.39553414 9.226412193 9.103287808 9.579315938 8.717676423 7.942514505 7.022367813 9.741466986 8.214319121 8.312882955 9.124121312 9.703903573 8.588714636 9.154818109 10.41574177 7.14974712 9.622051819 10.27029533 8.906890596 9.11113567 9.152284842 8.312882955 9.816983623 8.451211112 7.820178962 9.405141463 7.924812504 9.807354922 9.56414949 9.769837844 8.640244936 9.290018847 7.491853096 10.40301202 8.686500527 9.541096615 9.552669098 10.30492167 8.87036472 10.09275714 10.22037833 9.144658243 9.436711542 10.97369737 9.103287808 9.733015322 9.82336724 8.447083226

HMGB2 12.03204573 11.36358725 11.06002035 12.70282201 14.76658067 11.65060302 11.93847694 11.48532619 11.43514934 9.997179481 12.7934001 10.92407019 14.51982051 11.48482289 12.12282799 10.6110248 11.73386272 11.84117119 12.73745862 12.45095346 11.2737956 11.9918761 13.20594594 11.07079181 12.32895536 11.52061868 11.33315535 13.06827446 12.17430154 13.76175898 11.12282799 11.62296694 10.20457114 12.54448152 11.64745843 9.577428828 12.76362735 12.23421868 11.33817925 12.11309098 12.67815996 11.99576715 12.18735207 10.24436384 12.16616308 11.29232163 11.46913302 10.57175264 12.47902235 13.39030333 12.39339046 13.39003457 11.61746747 12.04131692 12.712527 11.02167404 10.68035952 11.79806672 10.25502857 12.72109919 12.32051852 11.63843591 11.38963134 12.95292321 10.54399845 13.00070427 11.7809498 11.07881795 12.32614856 12.53259944 13.48469704 12.47978026 12.74083483 12.37883671 13.23974768 11.91326343 11.72621816 15.0661733 12.69435789 12.61815605 12.63481105 10.7903485 13.34360214 11.82017896 12.57056729

HMGB1 12.87459719 12.96614491 14.18905236 12.59758704 13.19183034 12.2772874 12.22309669 13.29088282 12.8482317 11.13506795 13.41296672 12.56866869 15.05295007 11.85525783 12.49535539 13.84578405 12.84999144 12.80735492 13.63242727 13.41982854 12.72494012 12.93976285 13.50469487 12.69283342 14.19075065 12.99700302 11.76279726 13.11439305 13.21856324 14.77143788 12.96632519 13.17757526 11.74483384 14.18045311 13.91671921 11.94104761 14.00105627 12.6411486 13.62650758 13.04695345 14.46537509 12.53308634 13.15196787 11.85291959 13.96938652 13.44009052 12.32839444 11.34096276 14.22535809 14.27372276 12.70994538 13.22490609 12.4429435 13.16364968 14.81963027 12.01889562 11.82535634 13.02063276 13.02306125 13.52405192 13.55230886 13.43632115 12.6891244 13.67661826 12.95201316 12.98477446 14.07531201 11.77519873 13.17601727 13.57506646 13.65988443 12.23481743 13.70530839 14.39827608 13.56414949 13.14497682 13.5965392 14.80715364 13.30534928 13.00246344 14.32642949 13.0869655 14.22339841 12.25089054 13.81398155

HIF1A 11.47421294 12.5252761 14.25007631 13.42429753 13.3278333 11.81097322 13.19306328 12.66688971 14.15339369 11.70000615 13.56938096 13.00737773 13.12896095 11.47573343 11.77766628 12.91793144 13.31387503 12.94214792 13.05545192 12.97727992 13.24183423 14.50227242 14.4523056 13.07380685 13.32319553 11.11699368 13.38221872 10.73724734 13.55698496 13.58613609 13.53770375 14.19290922 11.32586758 14.63628475 15.00593151 13.04028972 11.92444139 13.80996905 12.49210354 12.36686859 14.3668003 12.13955135 12.88817245 10.06743436 13.40726776 12.56771846 12.80956718 10.66533592 13.5635538 13.50791849 12.71317168 13.55218876 12.11243951 12.98192399 15.32242112 12.89311189 11.38208359 12.37991982 13.49610478 12.6379833 13.05341684 13.64453229 12.60663654 12.92592526 13.3586512 12.56795608 13.5849625 10.78135971 13.24510699 13.03066714 13.56664869 12.45352787 13.04388171 14.2791027 12.64205169 12.48028532 12.62662165 14.22987027 13.12298972 11.44242519 14.50165062 14.29598412 14.55949659 14.32220984 13.22354925

HIC1 6.658211483 9.28077077 10.79279029 8.511752654 7.876516947 7.434628228 8.988684687 12.92851838 10.13699111 6.918863237 10.78953364 11.09077405 10.85018684 7.451211112 7.614709844 8.312882955 8.511752654 9.659995892 7.73470962 8.184875343 9.764871591 8.658211483 10.27844946 9.59058705 9.283088353 7.912889336 9.328674927 7.022367813 9.055282436 8.54689446 10.14338321 8.463524373 9.09011242 9.030667136 9.172427509 9.005624549 7.651051691 11.73343908 8.247927513 9.011227255 9.37286506 8.636624621 9.079484784 8.108524457 8.154818109 10.0768156 10.58683979 7.599912842 8.945443836 9.943979914 8.335390355 10.65284497 9.850186838 7.06608919 9.968666793 10.71080643 9.339850003 8.54689446 7.74819285 9.487840034 9.142107057 10.76818432 8.299208018 9.271463028 8.654636029 8.710806434 10.23840474 8.6794801 9.921840937 9.815383296 8.891783703 7.62935662 9.079484784 9.674192268 8.897845456 7.46760555 10.06608919 7.539158811 8.731319031 8.262094845 9.390168956 8.668884984 10.9432474 11.38370429 9.95419631

HESX1 6.022367813 4.906890596 5.491853096 1 5 1.584962501 4.807354922 4.087462841 4.807354922 1.584962501 5.95419631 4.64385619 6.108524457 2.584962501 3 4.857980995 3 4.247927513 4 5.754887502 5.64385619 2 4.906890596 4.523561956 3.584962501 4.247927513 3.584962501 3.169925001 4.247927513 3.906890596 2.584962501 3.169925001 2 4 6.247927513 5.700439718 1 5.285402219 2.807354922 4.459431619 4.700439718 2 5.426264755 4.321928095 3.700439718 5.614709844 3.321928095 2.807354922 4.169925001 4.523561956 5 5 3 5.832890014 5.247927513 2.807354922 3.906890596 5.882643049 2 3 5.781359714 3.700439718 4 5.087462841 4.754887502 4.857980995 5.321928095 4.087462841 4.584962501 4.169925001 5.392317423 4.392317423 4.700439718 6.459431619 4.700439718 2.807354922 5.426264755 5.754887502 5.285402219 7.761551232 6.807354922 4 6.845490051 4 5.044394119

HELLS 8.285402219 8.845490051 10.26912668 9.83447105 9.894817763 9.871905238 9.577428828 9.618385502 9.011227255 7.434628228 10.49385545 10.52258153 10.55362929 8.169925001 10.46658634 9.301496195 9.618385502 10.24079133 10.54882191 11.10197567 9.56414949 11.26033152 10.51471405 9.753216749 9.350939182 9.192292814 10.23361968 7.592457037 10.25620869 12.49909844 10.74483384 9.413627929 7.971543554 11.03066714 10.71338651 9.392317423 9.159871337 9.961449694 9.903881846 11.37503943 11.16364968 9.501837185 10.07414146 9.537218401 11.35974956 9.618385502 9.741466986 8.290018847 10.39874369 9.850186838 10.09671515 10.84470576 9.703903573 9.129283017 12.07848442 8.092757141 8.588714636 10.07146236 10.52552081 9.005624549 10.91438513 10.11504365 10.72792045 10.53915881 9.599912842 9.491853096 10.32867493 9.647458426 10.39339046 9.717676423 10.77396337 9.975847968 12.40726776 10.60733031 11.26620091 11.63934071 10.13185696 13.15813629 10.75321675 10.39231742 10.9505559 9.049848549 12.19105921 9.238404739 10.46454575

HDAC3 11.08613623 10.96650545 11.67991988 9.584962501 12.11861669 9.681238412 10.38909352 11.47065887 10.87574935 10.47978026 11.05596023 11.04439412 12.05052891 9.37286506 9.157346935 11.48179943 10.83999107 11.38532318 11.93553341 10.93663794 10.54399845 11.73301532 12.51224664 11.56795608 11.48582931 10.45840661 10.45840661 10.29462075 11.41626974 11.1959873 10.69522829 10.82177398 9.727920455 11.21735191 11.86263736 10.29001885 11.30549179 11.34040649 10.47167521 10.01402047 11.05188866 9.797661526 10.21310422 9.461479447 10.99364606 10.61746747 9.447083226 10.25738784 10.84705735 11.67991988 10.55937709 11.65150022 9.121533517 10.87574935 12.05392588 9.4325419 10.86959384 10.15101654 10.22158712 10.81057163 11.20884399 10.56033283 10.44811631 10.54978467 10.96578428 11.17617315 11.27612441 9.652844973 10.04984855 10.15101654 11.59944798 10.76238204 11.21916852 11.57553925 11.30606169 9.840777924 10.02513956 12.85038221 11.06002035 10.68123841 12.48003281 10.44397954 11.98726401 11.00702727 11.18363538

HDAC2 10.94690627 11.50382574 12.64700864 10.73555602 12.7561393 11.02721489 11.59245704 12.66688971 11.76570049 11.32136443 11.38801729 12.63526466 13.96190182 10.75321675 11.74483384 11.85447883 11.20823436 12.17679648 12.48305998 12.62548055 11.54303182 12.51717725 13.04814625 11.54930337 12.51865316 12.84999144 11.6329952 11.60594244 12.80473605 13.64813285 12.43697175 11.97441459 9.987264012 12.97584797 12.09770296 10.98085361 11.08679969 12.12928302 10.77148947 12.30748545 12.72685676 11.51026967 11.79116289 10.24555271 12.04473563 11.45635442 11.08945048 11.10131915 12.73978061 12.55650605 11.74357219 12.29059489 10.71080643 12.68189723 13.1313747 11.52991853 9.675957033 12.50804233 11.5526691 11.03066714 13.09308739 11.67904019 12.73237945 12.21036695 11.59385794 11.60547952 13.05629901 10.37503943 11.55602699 12.58918297 12.62021983 12.01367161 12.11309098 12.75843144 13.27044134 11.52552081 11.94178124 13.55889898 12.17305246 11.61378946 13.27000325 12.14847658 13.20074538 12.19136771 12.32699117

HDAC1 11.99576715 11.65642486 12.20089861 10.87958325 12.63934071 11.71166697 11.28077077 11.98726401 12.12638183 10.72877085 12.0950673 11.94471206 13.57589373 10.72877085 10.75739001 11.78871833 11.77066389 12.1582941 13.01593767 12.16270602 11.30206767 12.97495227 12.35121532 10.96506276 12.27932039 11.04712391 11.84705735 11.55698496 12.53235593 13.26047854 11.41732512 11.39927792 10.01959073 12.64385619 12.22550872 11.16113188 11.88187871 11.71896091 11.52258153 11.28828934 12.25532369 10.66088727 11.30320995 9.893301531 12.24168529 11.25797706 10.98584194 10.22881869 11.87690059 12.74986943 12.25797706 13.06305796 9.773139207 11.24079133 12.9893945 11.02444712 11.55362929 11.20457114 10.44708323 11.53964351 12.89349115 12.05562137 12.55626654 11.37937837 11.60316268 12.69479315 12.4429435 10.34872815 11.56367296 11.17055104 11.91401133 11.35590164 12.09209641 13.31811909 12.30320995 11.59525748 11.95128471 13.68068917 12.15608308 11.99647349 13.17975362 11.98334993 13.20991023 11.69479315 12.72728233

HBP1 9.105908509 10.32192809 11.75572215 10.04984855 11.20945337 9.677719642 10.5980525 11.03273453 10.58308277 8.965784285 10.06069593 10.26912668 11.25561875 9.54689446 9.144658243 10.84549005 9.97441459 11.06002035 10.27844946 10.86495992 10.2772874 10.38694025 11.97118427 10.857981 10.2227949 8.717676423 10.61746747 8.164906927 10.29347165 11.32811389 11.05052891 10.54012804 8.885696373 11.24317398 11.36959735 8.426264755 9.312882955 9.97441459 9.840777924 10.02652344 9.544964433 10.12670447 10.15734694 9.905387005 11.02306125 9.717676423 10.44708323 8.294620749 10.96866679 10.87881728 10.65910396 10.38154295 9.601770788 11.00492268 11.84862294 10.52258153 8.154818109 10.07012094 9.586839788 9.011227255 10.97226185 10.25148241 11.24376903 10.35314683 9.722807531 10.09934781 11.04302728 9.01402047 10.28193003 9.763212367 10.81297947 9.562242424 10.30149619 11.15481811 11.40514146 9.978710459 8.438791853 11.19167615 10.54785851 10.00281502 12.07848442 11.64610864 11.63571812 11.16804527 10.84470576

GTF2H2 9.754887502 6.794415866 8.515699838 4.584962501 10.2911707 7.17990909 8.103287808 7.022367813 6.62935662 7.159871337 6.285402219 7.044394119 8.927777962 5.321928095 6.044394119 8.027905997 8.499845887 6.584962501 8.622051819 7.607330314 7.044394119 9.368506462 6.906890596 6.965784285 8.174925683 5.95419631 9.310612782 7.960001932 7.303780748 8.092757141 8.022367813 6.781359714 6.321928095 8.554588852 6.569855608 7.21916852 5 6.357552005 6.988684687 5.807354922 7.584962501 7.700439718 7.366322214 5.977279923 7.348728154 7.592457037 5.554588852 5.491853096 7.994353437 7.888743249 6.321928095 7.276124405 4.857980995 8.06608919 9.445014846 5.857980995 7.247927513 6.303780748 5.614709844 5.807354922 7.129283017 2.807354922 7.06608919 8.550746785 7.022367813 9.005624549 7.54689446 4.087462841 8.960001932 7.055282436 7.357552005 8.14974712 7.693486957 7.539158811 6.857980995 7.960001932 6.14974712 8 6.741466986 8.247927513 7.055282436 7.714245518 7.22881869 8.614709844 8.266786541

GSTP1 14.74719646 12.93461233 13.39151212 11.80936621 13.99541385 12.17367714 12.77252078 13.95909634 11.47015044 12.23451809 13.19491072 13.86350875 13.39097501 12.14974712 11.90951811 13.08381171 13.23481743 12.61401961 13.80936621 14.09729146 12.49285462 11.63843591 14.63588813 14.033423 13.3586512 15.0317012 11.6105635 14.34810569 13.54496443 14.25140844 14.16341382 10.99223026 12.4429435 13.48142105 14.78043724 12.05833003 13.09687984 12.33203655 13.93452019 11.76694294 13.31967212 14.20411259 12.64001893 12.19629475 13.30078153 12.13795173 11.59665567 12.07748336 13.54532651 14.2087678 12.46403515 12.33957168 11.42992974 14.00886627 14.42626475 11.52552081 12.31797782 12.93645391 12.544723 12.75217155 13.14210706 13.72547277 14.87670878 11.16239133 14.0612868 12.6941402 12.92314175 12.14656868 11.93221475 12.1401907 14.11788656 13.14274528 13.42574042 13.25207404 13.46684121 13.70584834 14.21682163 14.25414285 13.60490066 13.59991284 14.86713392 13.32361776 14.62861641 11.72494012 12.78545247

GSTA4 10.77148947 11.30549179 9.390168956 8.30833903 11.42101286 9.733015322 10.34983409 11.16364968 10.35974956 9.269126679 12.16081685 10.08480839 10.43358544 8.573647187 9.224001674 8.791162889 8.271463028 10.31967212 8.933690655 10.63390341 10.43983088 9.465566405 11.4046094 7.994353437 9.002815016 10.10721708 9.154818109 9.108524457 9.99859043 11.55554777 10.59525748 10.58214198 8.836050355 10.45018025 10.88034881 8.204571144 6.554588852 9.73978061 9.781359714 9.769837844 10.14338321 10.28308835 9.063395081 7.475733431 10.55170826 9.136991112 9.103287808 8.957102042 9.843921051 10.28771238 10.73724734 9.95419631 8.144658243 11.20945337 11.48984796 9.259743264 9.238404739 10.48683502 8.64385619 9.659995892 10.6411486 9.276124405 10.09934781 9.14974712 9.575539247 9.539158811 10.00422047 8.475733431 8.948367232 9.839203788 9.686500527 9.493855449 9.83447105 10.29347165 11.13506795 9.108524457 9.903881846 11.98299357 9.677719642 9.025139562 13.47535346 9.238404739 11.06608919 10.14338321 9.967226259

GSS 12.11178774 10.90914305 11.35700209 8.647458426 12.42416629 9.764871591 10.58683979 11.00422047 10.96794671 9.071462363 10.59991284 11.3392933 11.98441846 8.962896005 9.972979786 10.74146699 10.58025857 11.10656294 11.24614677 10.7968508 10.58590145 11.23780747 10.95201316 10.65642486 11.32192809 10.83762793 10.85252951 11.69740201 12.45635442 10.06204614 11.39016896 10.17492568 9.533329732 11.87690059 11.43671154 8.714245518 9.562242424 10.41679753 9.599912842 10.13955135 10.82177398 10.33315535 9.773139207 8.326429487 10.73809226 10.43358544 8.971543554 10.12928302 10.99010396 11.39606956 10.98584194 11.78667803 9.501837185 10.82575383 12.10983065 9.763212367 10.98868469 10.32192809 10.49285462 10.76404222 11.36741475 10.82017896 10.95201316 10.36413466 10.70217269 10.9432474 11.86882255 9.927777962 10.03891899 10.21067134 11.15418521 10.56224242 11.18735207 11.48431942 11.32699117 8.396604781 11.02097994 12.01924322 10.92999806 10.65731845 11.87805091 10.62570884 11.75780667 10.97297979 11.97297979

GSR 11.16239133 11.39606956 13.0202855 11.3426303 12.15797845 11.4807902 10.34207467 12.81678368 10.54109662 10.72536626 11.29289676 11.08148344 11.66799854 9.764871591 10.25029842 11.36522885 11.88874325 12.44449729 11.28135051 11.14593215 11.69522829 13.16914208 11.54689446 12.54133866 11.33371443 10.92851838 10.83447105 11.42416629 12.00597536 12.18177344 12.78033471 10.86805085 9.368506462 13.03600185 13.25959616 10.57364719 11.79400965 10.87344411 11.62296694 11.7077905 13.23346989 12.43045255 11.49135207 9.407267764 10.74399286 10.60269887 9.978710459 9.941047606 12.70649602 12.14625045 11.28828934 11.88912366 10.22400167 12.07848442 13.63208641 9.995767151 9.306061689 11.66533592 12.29174628 12.05663772 12.38154295 11.30720081 12.72302093 12.18394547 10.87498135 12.08945048 12.04575966 10.20823436 9.918863237 10.6794801 11.38154295 11.65105169 12.13282101 12.93036775 11.1792871 10.7548875 11.70347105 13.09720914 12.87555739 10.47065887 12.52110964 11.9505559 12.15069929 10.55842071 12.65082737

GSK3B 10.33203655 10.65194861 12.50432245 10.48884435 11.80413102 10.76901132 11.32979634 11.51914479 11.66044165 9.055282436 10.66888498 11.52160044 12.79299359 10.05528244 10.21310422 10.10328781 10.18487534 11.5574637 11.36686859 11.45224124 10.73809226 11.67991988 11.96325736 11.15797845 11.08214904 9.781359714 10.83130724 9.381542951 11.60594244 12.6263935 11.66977089 11.39392668 9.348728154 11.4918531 11.48331195 9.945443836 10.83683936 12.45866293 10.18239435 12.0406322 11.33539035 11.3140167 10.84862294 9.469641817 12.25177826 10.15608308 10.56414949 10.57175264 11.74357219 11.59105477 10.45840661 11.67109873 10.6617781 11.23242093 12.69217958 11.25266543 9.882643049 10.73978061 10.80815977 10.77643303 11.76155123 11.77684423 11.82892983 11.06272077 10.95419631 10.89708913 11.27786855 10.18982456 10.19105921 10.60177079 10.89557528 10.02513956 12.11894107 11.19352536 12.36686859 11.30092449 10.95346896 12.14815877 11.53138146 11.71510401 12.86727874 11.98761931 13.65698342 11.39606956 11.65060302

GSK3A 11.3553511 11.09341756 12.02652344 11.00912879 12.1091777 11.10066234 10.9822806 11.85447883 11.14784089 9.751544059 11.18611424 12.58096514 12.16427844 9.422064766 11.37014248 10.89102419 11.12541347 11.39070637 11.58683979 11.52012755 11.78790256 11.61746747 11.70476824 12.12282799 11.35974956 10.77396337 10.88034881 10.58214198 11.80735492 12.34235251 12.05494342 10.61010206 9.938109326 11.74567432 11.88149638 10.14974712 10.09934781 12.13249973 10.38694025 11.71510401 11.85408918 10.65821148 10.47471995 9.689997971 11.54109662 10.95710204 10.24436384 10.81458247 12.33985 11.66666784 10.92555444 11.66400276 10.91363743 11.61930296 12.27496047 11.57601187 11.97369737 10.27496047 10.40194612 10.89708913 12.52698821 11.95927751 10.68999797 10.31174832 11.24970606 10.86650621 12.23511671 9.797661526 11.01402047 10.83605036 11.06877828 11.37395266 10.99223026 12.08646799 11.21067134 11.3426303 11.93479659 12.11113567 12.12088584 11.75655632 12.857981 11.82177398 13.2816403 11.25148241 11.3553511

GRN 14.89187861 15.28778451 17.26509306 12.09967655 15.91281918 11.81898154 13.83318659 14.88269081 15.37754947 11.47775827 14.24896526 15.50133962 13.84392105 13.7285583 11.98619759 14.0799014 14.77617597 14.70978388 17.21533392 15.58792398 15.638747 16.04307002 14.63543472 14.45449209 16.18070562 13.50866138 14.40554038 14.40593919 16.96796921 14.50276966 15.60802375 14.46294953 13.46237446 16.13272062 16.11885998 12.23989682 13.37829486 15.40457614 13.19275514 14.14720492 13.74472874 13.05917544 14.41963106 12.56581611 14.68376224 15.85404047 13.03101191 13.79482197 15.8327417 16.29079285 13.57908018 15.59947704 13.46071185 15.02180415 15.29181821 14.22490609 15.13049012 14.74646184 15.11060565 13.51224664 16.36671494 15.82361603 14.69207057 12.70022295 14.69539143 15.48582931 16.38759666 14.40500847 13.3927199 14.45936758 14.48261891 14.56301747 13.46926024 16.74237523 14.85296834 13.09490241 14.11821111 14.56700537 13.90058382 12.83031713 16.5687429 16.07206978 15.07389051 14.45146871 15.42623199

GRB2 13.75801496 12.31741261 13.77807713 11.6956633 13.21340804 12.08878824 12.32755264 12.50779464 13.2039597 10.86341196 12.61010206 13.11015702 12.38073161 10.87498135 12.1234748 12.03204573 11.91886324 12.48305998 13.24153633 12.6379833 12.68255574 13.78729043 13.12767197 12.66999228 12.55842071 12.0101784 12.43671154 11.90124403 12.81758329 14.2729942 12.10656294 12.67308819 11.25502857 12.90951811 13.34360214 10.66711154 11.98832965 12.43358544 12.54158066 11.98655315 12.87805091 11.86031105 12.48909532 10.70735913 12.21765483 12.70886834 11.37721053 10.9708249 12.84646981 13.60825482 12.28077077 13.5315033 11.09275714 12.36550227 13.70574037 12.35369821 12.40593919 12.23750875 12.39339046 12.08015131 12.76321237 12.82495874 12.04712391 11.65194861 11.92962828 12.42888355 13.45468485 10.87267488 12.48633225 12.14433959 12.02513956 11.78912605 12.80574387 13.88397967 12.63707766 11.82217246 12.27466934 13.64216454 12.77252078 11.88264305 13.68299458 12.17398937 13.66600203 12.35782688 13.36741475

GPX4 13.39325637 12.99982388 14.09432515 11.74399286 14.29784769 11.93258387 13.35259523 14.71553306 12.78197437 11.49235395 11.95782756 13.71885391 13.34471201 11.33371443 12.51766939 12.82634986 12.80170836 12.67131991 14.03643121 12.84881852 13.0086037 13.60095823 13.23421868 12.37449615 13.33455263 12.92239857 12.79177338 14.23564031 13.22339841 12.69696753 13.73375682 12.51594618 12.28857774 13.58425789 14.04874229 11.436191 12.95437809 12.92109709 12.7084373 12.01332267 12.60686784 13.41084881 12.13987106 11.01122726 12.41098127 13.01976445 9.831307244 12.1424262 12.77889848 13.83180205 12.55314928 13.58930003 11.71938882 12.92888844 12.94690627 12.31373335 12.84764464 11.98334993 12.18239435 12.23780747 13.10164745 13.56200386 12.25738784 11.35369821 12.46709603 14.42265613 14.32094154 11.44656641 12.13923157 12.38046107 14.38458141 12.58683979 12.48053778 14.24309959 11.79116289 12.26532202 13.74072944 14.32460249 13.64080979 12.86669938 14.24317398 13.14370208 13.30206767 12.11861669 13.60686784

GPX1 13.67849011 13.67109873 15.70970312 9.821773982 13.83308774 11.58072965 12.41071633 13.68540583 13.57104155 10.35204343 13.0970445 14.01558928 12.76113565 11.78176951 11.62067804 12.37558251 12.90199819 12.36604895 15.13032924 13.48997336 13.98886217 13.22686372 13.00615073 12.7071434 14.25340433 13.66522487 11.9248125 13.99284985 14.58754314 13.23406895 13.61321393 11.98761931 13.07798397 14.0557061 13.65329295 9.733015322 11.65597786 13.2772874 12.96542357 12.17959813 11.52503135 11.81458247 13.11390492 10.36959735 12.78586111 13.98477446 11.01052811 12.16679075 13.6079082 14.24324838 12.52478655 14.21621536 11.28828934 12.40407714 12.90707843 12.85467362 14.18681065 12.76217438 12.77128312 12.28193003 13.96244419 14.11113567 12.07948478 12.03479896 13.49335512 13.75572215 14.87046105 12.01889562 12.58308277 12.88588699 13.15054064 12.86205613 11.54737656 14.98521934 12.41679753 11.57222651 13.61298365 13.27699674 13.16349244 11.37937837 14.87478928 13.76766721 13.83841608 12.57459353 13.67375074

GHRHR 0 0 1 0 0 0 3 2 2 1 2 1 3.321928095 0 5.554588852 1.584962501 2.321928095 4.321928095 1 0 2.321928095 1 3 1 1.584962501 0 3.459431619 1 2.584962501 2 1 4.392317423 1.584962501 3 0 6.189824559 3.459431619 2 0 2.321928095 1.584962501 3.169925001 1 3.807354922 1 0 2 0 1.584962501 3 2 0 2.321928095 0 3.321928095 3.807354922 1.584962501 1 1 1 3.321928095 3.169925001 1 0 2 2.321928095 1.584962501 0 1 0 1 1 2 1 5.584962501 2.321928095 1 0 3.169925001 2 2 3.459431619 2.584962501 4.584962501 1

GHRH 2.584962501 0 1 0 0 1 0 0 0 0 1 1 3.169925001 0 3.700439718 1.584962501 0 0 1 1 0 1 0 2.807354922 0 2 1 0 0 0 0 0 1 1.584962501 0 1.584962501 0 2 0 0 2.807354922 1 0 4.95419631 0 1 0 0 1.584962501 1.584962501 0 1 2.584962501 0 0 0 0 2 0 0 0 0 1 0 2 1.584962501 1 0 0 0 0 0 0 0 1.584962501 0 2.584962501 2 0 1 1 0 1 0 2.321928095

GHR 8.86727874 10.71510401 10.63843591 7.888743249 9.497851837 10.74986943 10.53138146 9.541096615 10.15608308 10.69435789 10.31854281 10.64565843 10.60362634 10.64205169 10.54012804 12.39124359 11.17492568 11.18735207 9.577428828 9.558420713 10.27961058 10.69435789 13.55901852 10.26912668 9.278449458 8.238404739 8.781359714 8.531381461 9.915879379 12.09605624 10.30720081 8.968666793 7.266786541 11.60640521 12.7542612 9.030667136 11.19290922 10.22400167 9.656424863 9.197216693 10.357552 9.535275377 9.257387843 8.257387843 11.33259606 9.147204925 6.599912842 7.665335917 11.78626963 10.44086917 10.78790256 7.826548487 9.967226259 10.79522797 11.60316268 8.550746785 10.50183718 9.550746785 10.69174352 9.46760555 11.22701419 10.24317398 7.312882955 8.027905997 8.159871337 10.17741954 10.04575966 9.829722735 5.832890014 9.902375114 11.20823436 9.357552005 10.32755264 12.18611424 11.95419631 8.73809226 8.703903573 11.36796071 8.721099189 11.0175044 12.43201985 9.63481105 11.85720347 10.34207467 11.33873638

GH1 0 0 0 1.584962501 0 1.584962501 0 1 0 0 0 0 1.584962501 1 3.584962501 0 1 0 1.584962501 0 1 1 3.321928095 0 0 0 0 0 1 0 2.321928095 0 0 0 2 0 0 0 0 1 1 1 1 3.169925001 0 0 0 0 0 1 0 0 1.584962501 0 2.807354922 0 0 1 0 0 0 0 0 0 1 1.584962501 0 0 0 0 0 0 1.584962501 1.584962501 1 0 1.584962501 0 1.584962501 0 1 1 1.584962501 1.584962501 1

GDF11 11.11504365 11.25148241 10.21188829 10.19229281 13.66200072 10.20701432 11.42574042 12.42809841 10.8494051 11.20518233 11.24614677 10.65642486 11.95019135 9.364134655 11.1382718 13.30734314 10.69957245 12.40832974 10.87036472 10.82177398 11.38532318 11.61010206 12.45712433 14.22836778 11.03686045 10.91363743 11.78504371 10.92777796 10.6617781 10.857981 10.87958325 10.32530546 8.930737338 10.32192809 12.65015421 9.710806434 10.59245704 10.97154355 10.07012094 11.07614753 12.86011703 11.34983409 9.583082768 8.30833903 11.94068065 11.14593215 9.722807531 9.854868383 11.64745843 11.45378506 10.84549005 10.76155123 10.48280796 11.64205169 13.06911406 11.04302728 11.52160044 10.2772874 10.76570049 10.509775 11.43306377 11.24138736 12.16018658 9.736401931 10.53235593 11.81658371 11.65910396 9.131856961 9.686500527 10.17242751 11.4812949 10.44086917 10.74062404 12.7026056 11.47623991 10.16867212 11.17055104 11.77889848 10.01122726 10.5574637 12.65508345 9.915879379 13.02375435 11.83011903 11.8153833

GCLM 9.71596199 9.240791332 11.11504365 10.31174832 9.588714636 9.095397023 9.409390936 9.398743692 10.84705735 8.038918989 9.751544059 10.56128795 10.25148241 7.569855608 9.47370575 9.535275377 10.27844946 9.485829309 9.677719642 9.980139578 10.56605404 9.861086906 9.703903573 9.933690655 9.095397023 9.250298418 9.847057346 8.154818109 9.796039609 9.951284715 9.73978061 10.45429929 7.754887502 10.46658634 11.27204652 8.194756854 10.01820018 9.642051693 9.197216693 9.09011242 9.674192268 9.019590728 10.18487534 8.724513853 10.11113567 9.856425529 9.082149041 9.366322214 9.73809226 10.61654884 8.243173983 9.967226259 8.693486957 8.942514505 10.19475685 9.527477006 8.370687407 9.977279923 9.457380879 10.42731284 10.07280253 9.674192268 9.197216693 9.571752644 9.442943496 9.447083226 10.60733031 7.794415866 9.517669388 8.965784285 9.649256178 8.784634846 10.07547915 11.15860969 9.105908509 8.581200582 9.052568051 10.15734694 10.0768156 9.647458426 11.53478921 8.651051691 11.51963625 8.930737338 10.11634396

GCLC 10.20212382 10.15734694 11.23361968 10.04302728 10.06608919 8.696967526 9.027905997 11.13955135 11.32530546 7.965784285 10.83447105 10.47775827 11.91886324 8.50779464 9.776433032 9.11113567 9.159871337 9.930737338 10.25738784 11.00281502 10.51076417 11.09077405 10.57364719 10.25620869 9.361943774 8.554588852 9.108524457 7.442943496 10.34318572 10.94982671 9.921840937 10.56510208 7.266786541 10.1382718 12.07246761 8.388017285 6.584962501 9.271463028 8.442943496 9.28077077 10.17990909 9.306061689 9.531381461 7.622051819 11.23720996 10.63208641 9.324180547 8.45532722 10.46862404 10.07146236 9.550746785 9.792790294 8.14974712 8.169925001 11.33371443 8.581200582 8.087462841 10.58214198 9.807354922 9.055282436 10.83209885 9.675957033 10.46964182 8.918863237 9.618385502 9.513727596 10.16741815 7.888743249 8.475733431 9.306061689 9.21916852 9.240791332 10.06069593 12.26707927 10.06204614 10.07948478 9.174925683 10.94763694 9.797661526 10.02928723 11.81938079 10.28193003 11.6930513 10.02375435 11.02444712

FOXO4 8.405141463 9.129283017 10.11894107 9.247927513 9.786269628 8.204571144 9.409390936 10.9068906 9.987264012 8.194756854 8.113742166 9.779719355 11.60316268 7.845490051 8.271463028 8.554588852 8.535275377 9.861086906 11.44035012 9.625708843 8.665335917 10.05799172 10.80735492 9.411510988 8.876516947 7.686500527 9.558420713 8.199672345 9.197216693 11.66266838 9.379378367 9.571752644 7.864186145 9.741466986 10.7968508 7.276124405 8.693486957 9.612868497 8.675957033 9.515699838 9.419960178 8.982993575 9.37721053 6.62935662 8.471675214 9.01402047 8.434628228 8.707359132 9.366322214 9.86727874 8.810571635 10.27844946 8.921840937 9.361943774 10.06474276 9.513727596 8.326429487 8.909893084 8.483815777 9.303780748 9.73809226 9.876516947 9.682994584 10.16867212 8.942514505 9.550746785 9.851749041 9.002815016 9.837627933 9.209453366 9.01402047 9.294620749 8.930737338 10.13185696 10.92999806 9.136991112 9.48984796 9.103287808 9.095397023 8.962896005 10.21310422 10.01541505 10.89860139 9.594324604 9.746514321

FOXO3 9.594324604 11.36905201 12.60014522 11.93516505 11.46352437 10.58402294 11.0175044 11.91550596 11.33650656 10.79116289 12.22580994 12.47851685 12.5992155 9.640244936 11.07414146 11.52405192 10.1959873 12.46071185 10.77561028 11.32136443 10.14593215 11.56510208 13.46454575 11.35149141 10.83130724 10.69696753 11.85018684 9.533329732 10.71681946 13.02184752 11.85369942 11.50432245 9.436711542 12.06877828 12.03823313 10.13057056 10.21310422 11.7026056 10.16239133 12.31938988 11.40301202 11.29059489 11.06541613 8.807354922 11.87843415 11.02513956 10.32080055 9.505811554 11.73259144 13.6851868 11.27612441 11.21188829 10.69348696 12.24763088 12.20487677 11.52796564 10.23122118 11.06204614 10.82495874 10.06069593 12.15703107 10.7548875 12.01192607 12.39954497 10.29462075 11.04575966 13.14990586 10.83051521 10.37286506 11.95346896 11.10787091 10.76901132 11.38747887 11.52601011 12.66333572 10.49685378 11.12863881 12.5674808 11.49135207 11.50481899 12.15481811 11.53478921 12.52991853 11.52649924 11.53332973

FOXO1 8.312882955 9.400879436 10.13442632 11.18177344 10.1711768 8.339850003 7.839203788 9.789533645 9.912889336 7.607330314 8.640244936 10.53818893 10.51865316 8.535275377 8.764871591 8.921840937 8.921840937 9.35974956 9.315149562 9.758223215 9.837627933 8.816983623 9.649256178 10.68211676 8.965784285 7.21916852 8.189824559 7.321928095 9.908392621 12.64137442 10.49085088 8.379378367 7.813781191 9.824958741 11.08281434 6.845490051 8.741466986 9.837627933 8.640244936 10.22400167 9.796039609 8.413627929 10.16867212 9.463524373 11.17430154 8.985841937 8.829722735 8.169925001 9.296916207 10.09934781 9.958552715 9.457380879 8.491853096 8.409390936 10.05799172 9.233619677 7.118941073 9.638435914 7.531381461 8.781359714 10.11243951 8.348728154 9.187352073 9.920352855 9.948367232 9.060695932 10.34318572 8.252665432 9.712527 8.864186145 9.245552706 7.721099189 9.805743872 10.64295422 9.960001932 8.816983623 8.54689446 11.58730873 9.430452552 8.647458426 10.67242534 8.903881846 11.29576893 9.982993575 9.744833837

FOXM1 11.47623991 11.06810648 10.40087944 12.5282099 13.2632692 10.80815977 13.00123225 12.66777684 12.39687232 10.20823436 12.99063583 12.42442876 11.68956125 10.68299458 10.02097994 12.43514934 11.8989792 11.77725532 12.11211366 12.32755264 12.16333519 12.1176431 11.39392668 11.80372753 11.26033152 10.91662592 12.06035818 11.0768156 11.46658634 12.75822321 12.23451809 11.4252159 10.39016896 13.63651134 11.1234748 11.78790256 11.32811389 12.01052811 11.55650605 12.71080643 12.38235383 11.90312868 10.92851838 9.28771238 12.28395649 10.28308835 11.55410915 11.215533 12.19198451 14.15703107 11.5137276 14.16616308 10.83447105 10.75655632 11.89254282 10.88187871 11.6956633 11.19721669 11.8622499 12.39927792 11.35149141 11.51076417 11.88760142 11.43410693 9.2644426 12.03376711 10.56700537 9.909893084 11.32361776 10.34429591 11.79238361 10.87036472 12.70238916 13.01941698 11.96217303 12.35093918 10.77889848 13.9202598 11.63481105 12.40567333 12.86244364 10.87805091 13.67319864 11.17367714 12.05052891

FOS 8.463524373 11.71080643 14.66550247 12.98477446 12.6635581 10.84627391 9.59058705 11.16239133 12.71660514 11.90162116 11.97190275 15.37429236 9.909893084 12.75530489 12.03754695 11.56414949 9.28771238 9.614709844 13.10164745 10.38154295 12.74336181 9.971543554 13.08878824 12.82535634 11.75196241 7.77478706 11.46658634 9.930737338 11.13185696 8.353146825 12.16018658 11.78790256 7.994353437 12.59245704 10.75988818 13.26062556 9.882643049 10.05934446 10.98584194 9.312882955 11.07614753 9.957102042 12.9316609 8.596189756 10.99929539 12.32164629 10.49285462 10.56890615 10.48984796 10.26678654 10.04984855 10.01402047 9.008428622 9.68474862 12.6851868 9.493855449 10.57837269 11.31910758 9.221587121 15.56066122 10.30720081 10.01122726 12.41732512 8.016808288 14.90322284 12.28540222 14.34962679 8.754887502 8.562242424 14.84058125 12.70822173 9.560332834 11.36577564 15.8197051 12.87286723 13.67275681 9.481799432 11.51520703 11.65374078 11.98690862 10.73470962 10.77148947 14.67292251 9.627533884 14.49498055

FLT1 9.826548487 11.49085088 12.18332522 10.07815081 11.38262403 9.519636253 10.77643303 11.12605912 11.29174628 9.815383296 10.40301202 11.88187871 11.69696753 10.70303839 11.28655776 11.5464122 11.81498294 11.67991988 10.39446269 12.18487534 12.40301202 11.73513288 12.34457332 11.32923574 11.65374078 8.451211112 11.24792751 8.194756854 11.26795708 12.46147945 12.18146288 10.54109662 9.988684687 11.19352536 11.54399845 9.579315938 11.9918761 11.69087101 11.76942464 10.60455323 9.445014846 9.645658432 10.7903485 8.948367232 11.34318572 11.86611979 10.5137276 9.461479447 11.85018684 11.14082977 12.04405253 11.28308835 10.95710204 10.68035952 12.6209071 10.28193003 9.939579214 12.63775695 10.06339508 11.38208359 11.27204652 11.67551604 10.63208641 9.894817763 12.73237945 10.36741475 12.11178774 9.857980995 10.36084708 11.37721053 11.0721326 10.02928723 11.8029202 12.97763769 11.28655776 10.90162116 11.55554777 11.47927503 11.04780555 8.700439718 13.03307881 10.34207467 12.51693112 10.79197682 12.27670602

FGFR1 13.33650656 13.65004199 16.16824119 14.32804375 14.62153681 11.96108789 14.11983275 15.65393666 13.71874691 12.14625045 14.24064229 15.04793333 13.84058125 11.52845411 13.13024878 12.97727992 13.23062093 15.54987489 15.17039455 14.15632013 13.46849677 14.88202205 16.43162818 15.39030333 13.34457332 11.47269084 13.22475539 11.37883671 15.18708139 13.20304206 15.38373804 13.06793848 12.11926538 14.84828061 13.45056691 12.20731943 12.9275928 14.84028619 14.00632608 15.08397787 13.65731845 16.80949182 12.80856203 12.87709238 14.17835362 12.48230378 12.90350531 13.50034397 15.07066605 15.23440582 14.9645665 14.64835758 13.40194612 14.80649928 14.62667868 13.52049591 14.18611424 14.13506795 14.75028827 15.44601709 14.96718122 15.40104616 13.3620808 12.72706956 13.86244364 14.04003281 13.92277021 12.74672424 11.40939094 13.65150022 13.06861036 14.48520038 14.54677391 15.01789582 15.4515975 14.66539144 14.33845784 15.72222052 14.4872748 14.57860856 16.75282489 16.59851781 13.07748336 14.49516798 13.67220432

FGF23 0 2 1.584962501 1 4.523561956 1.584962501 4.247927513 0 0 0 0 1 4.64385619 0 4.807354922 5.754887502 4.321928095 1 1 1 5.357552005 1 2.321928095 2.807354922 0 0 0 1 0 0 0 5.169925001 0 6.129283017 1 6.129283017 0 1 1 3.584962501 3.584962501 4.807354922 1 3.321928095 2 3.700439718 0 1 4.459431619 1.584962501 7.539158811 1.584962501 3.584962501 1 4 0 0 4.087462841 0 4.95419631 0 4.584962501 0 0 1.584962501 4.247927513 0 1 1.584962501 0 0 2.321928095 2.584962501 4.247927513 3.459431619 3 0 1 2.321928095 0 3.459431619 11.2644426 2.807354922 2 3.321928095

FGF21 0 1 0 2 0 1.584962501 5.357552005 5.209453366 2 0 0 1 3.169925001 1.584962501 4.392317423 5.95419631 0 1 3 3.169925001 1.584962501 0 6.044394119 2.807354922 0 1.584962501 2.584962501 1 0 2.807354922 1.584962501 3.807354922 0 2.807354922 1 5.169925001 0 2 2.321928095 0 2 0 0 2 0 0 0 0 2.807354922 3.700439718 0 1 2 5.129283017 1.584962501 0 2.584962501 1 0 1 0 1 0 0 2.321928095 1.584962501 2.807354922 0 0 0 0 3 1.584962501 2.584962501 1 0 0 3.584962501 2.807354922 2.584962501 2.584962501 0 3.459431619 0 0

FEN1 11.77725532 10.34207467 10.11113567 11.12928302 11.79156991 10.29462075 10.52747701 11.23959853 10.27496047 8.761551232 10.86495992 10.48582931 10.67065625 9.392317423 10.57742883 10.7548875 10.9822806 10.86727874 11.44449729 12.2848241 10.11504365 12.53065018 11.04984855 10.99293834 11.52356196 10.87190524 10.9068906 10.98299357 11.4429435 13.06726628 11.32361776 10.91960824 9.84862294 12.78238399 11.8963324 9.645658432 10.30263892 10.71166697 11.06272077 10.027906 11.7883105 10.36741475 10.92555444 8.826548487 11.25148241 10.47775827 9.978710459 9.436711542 11.37991982 12.27554256 10.09143539 12.72045804 9.751544059 10.90237511 12.70908381 9.030667136 10.39553414 11.39927792 11.01611184 10.5526691 10.64295422 11.51175265 10.9822806 11.07614753 10.41996018 10.58120058 10.64925618 9.874981348 12.65955 11.11374217 12.15355203 10.65553072 12.11439305 12.06944977 11.70043972 11.51668495 11.67595703 13.09143539 11.76445696 11.203348 12.66977089 10.30263892 10.73724734 11.39124359 11.69913863

FAS 7.22881869 8.016808288 11.77066389 9.721099189 9.721099189 6.459431619 8.438791853 9.964340868 9.661778098 8.092757141 7.73470962 7.06608919 7.011227255 5.95419631 5.64385619 8.794415866 8.038918989 10.44501485 8.682994584 8.159871337 9.592457037 10.31061278 11.29863541 7.257387843 7.787902559 5.807354922 10.26091953 7.426264755 9.902375114 7.247927513 9.394462695 10.52160044 7.924812504 10.26678654 7.672425342 8.071462363 9.763212367 10.14593215 8.8008999 8.139551352 8.044394119 7.942514505 9.832890014 4.807354922 5.285402219 7.924812504 10.21916852 6.087462841 9.348728154 9.303780748 8.400879436 10.10590851 7.906890596 10.23361968 10.8008999 9.463524373 7.839203788 6.686500527 9.424166289 6.988684687 9.348728154 4.64385619 5.807354922 9.335390355 7.21916852 8.682994584 10.17866485 7.392317423 9.525520809 6.569855608 8.97441459 9.317412614 5.930737338 9.290018847 10.04165915 5.584962501 5.357552005 9.861086906 10.62021983 9.082149041 10.509775 9.271463028 8.854868383 10.28193003 9.415741768

ESR1 6 6.392317423 8.744833837 7.159871337 8.071462363 5.169925001 8.689997971 6.820178962 7.781359714 3.169925001 7.622051819 8.751544059 8.651051691 5.321928095 8.447083226 7.672425342 6.906890596 8.864186145 6.820178962 7.721099189 7.64385619 6.754887502 7.768184325 7.607330314 5.426264755 4.247927513 7.857980995 6.266786541 7.6794801 8.876516947 6.794415866 8.727920455 5.426264755 8.276124405 7.357552005 8.033423002 6.62935662 6.247927513 6.539158811 6.686500527 6.108524457 5.754887502 7.851749041 6.285402219 7.499845887 6.894817763 5.285402219 7.139551352 7.098032083 7.54689446 6.62935662 6.832890014 6.022367813 7.614709844 8.675957033 8.483815777 5.672425342 6.06608919 6.321928095 5 7.948367232 6.044394119 5.807354922 5.930737338 5.554588852 7.087462841 6.392317423 6.918863237 6.022367813 7.129283017 6.129283017 6.807354922 6.459431619 8.882643049 7.348728154 6.727920455 6.741466986 5.129283017 5.727920455 8.864186145 8.45532722 8.361943774 7.033423002 7.076815597 7.475733431

ERCC8 8.438791853 7.768184325 8.888743249 6.794415866 10.27029533 6.599912842 8.247927513 8.438791853 8.693486957 7.787902559 8.86727874 8.475733431 10.65374078 7.375039431 6.882643049 8.535275377 8.426264755 8.682994584 9.290018847 8.361943774 8.357552005 8.011227255 9.652844973 8.430452552 8.696967526 7.06608919 9.328674927 7.17990909 8.829722735 9.77807713 8.303780748 8.434628228 6.965784285 9.103287808 8.897845456 6.807354922 9.108524457 8.603626345 8.74819285 7.813781191 8.64385619 8.353146825 8.082149041 7.321928095 9.055282436 8.276124405 8.124121312 8.603626345 8.851749041 9.285402219 8.361943774 8.45532722 7.409390936 8.124121312 10.90839262 7.491853096 6.807354922 7.948367232 8.233619677 7.199672345 8.927777962 7.247927513 8.479780264 7.965784285 8.8008999 8.463524373 8.971543554 6.942514505 7.876516947 8.049848549 9.033423002 8.252665432 8.957102042 8.939579214 8.290018847 7.523561956 8.055282436 10.25856603 8.339850003 7.569855608 9.68474862 8.829722735 10.4604559 8.912889336 8.900866808

ERCC6 6.918863237 8.523561956 9.768184325 7.044394119 8.672425342 6.392317423 8.607330314 10.07280253 8.611024797 7 7.971543554 8.335390355 8.977279923 7.577428828 7.95419631 8.027905997 7.491853096 8.797661526 6.022367813 8.383704292 7.442943496 9.13442632 9.918863237 8.539158811 8.330916878 7.076815597 8.73470962 7.539158811 7.924812504 9.865733271 8.945443836 8.721099189 7.06608919 8.787902559 8.994353437 7.912889336 5.882643049 8.985841937 7.285402219 8.535275377 9.483815777 8.945443836 7.832890014 7.442943496 9.116343961 7.584962501 8.144658243 7.539158811 8.194756854 8.005624549 8.744833837 8.471675214 7.768184325 8.303780748 9.661778098 7.321928095 7.665335917 7.098032083 7.768184325 7.392317423 9.09011242 8.271463028 8.299208018 7.592457037 7.459431619 8.335390355 8.672425342 7.366322214 7.721099189 7.159871337 8.164906927 7 8.573647187 9.019590728 9.842350343 8.262094845 7.392317423 9.73978061 8.74819285 9.108524457 10.15860969 7.614709844 10.3858624 8.483815777 8.22881869

ERCC5 12.18394547 9.121533517 10.59712143 9.027905997 9.909893084 8.016808288 8.876516947 9.686500527 9.381542951 7.665335917 10.04848687 9.169925001 9.428360173 8.873444113 9.385862401 9.681238412 8.721099189 10.05934446 9.812177306 9.906890596 8.741466986 9.419960178 10.18982456 9.459431619 9.457380879 8.361943774 9.579315938 8.924812504 9.675957033 10.32530546 8.942514505 9.126704473 7.06608919 10.88264305 10.67419227 9.182394353 8.665335917 8.159871337 9.231221181 9.231221181 9.868822555 9.485829309 9.164906927 7.348728154 10.02097994 9.294620749 7.95419631 7.011227255 9.645658432 10.15860969 9.457380879 10.62479546 8.581200582 9.37286506 10.86727874 7.994353437 7.257387843 9.667111542 8.661778098 7.787902559 10.42101286 9.129283017 8.515699838 9.836050355 9.77148947 9.891783703 9.961449694 9.105908509 9.06608919 9.6794801 11.73216743 8.581200582 10.6635581 10.28655776 11.31797782 9.055282436 11.05731488 11.95019135 9.929258409 8.675957033 10.71853288 8.727920455 10.22037833 10.40194612 10.06339508

ERCC4 8.499845887 9.154818109 9.746514321 7.562242424 9.499845887 8.224001674 9.573647187 9.722807531 9.167418146 8.693486957 8.588714636 9.449148645 10.56224242 7.055282436 8.357552005 9.312882955 8.794415866 9.278449458 8.243173983 9.194756854 8.417852515 9.147204925 9.392317423 9.11113567 8.294620749 8.044394119 9.294620749 6.87036472 8.204571144 9.779719355 9.493855449 8.62935662 7.087462841 9.878050913 9.071462363 6.977279923 8.607330314 9.159871337 8.049848549 8.968666793 9.417852515 7.577428828 9.01402047 6.409390936 8.491853096 8.076815597 8.174925683 8.451211112 9.296916207 9.283088353 8.581200582 8.647458426 9.057991723 9.917372079 9.483815777 7.839203788 9.063395081 9.273795599 8.960001932 7.607330314 9.144658243 8.515699838 8.262094845 9.276124405 8.46760555 8.357552005 9.144658243 7.17990909 8.303780748 8.30833903 7.900866808 7.971543554 9.276124405 9.756556323 9.174925683 7.95419631 7.562242424 9.583082768 9.164906927 9.189824559 10.71681946 9.562242424 10.14210706 10.20089861 9.440869168

ERCC3 11.96506276 10.30606169 11.6411486 10.47876962 11.82217246 9.703903573 10.7548875 11.7198166 10.09011242 9.733015322 11.30947635 12.22037833 11.70087316 10.10983065 10.10066234 9.83447105 10.40301202 10.39124359 11.49034951 11.712527 10.18735207 12.16239133 11.96072599 11.027906 11.31004468 10.357552 11.03960452 10.62479546 11.47927503 12.06440596 11.48582931 9.977279923 8.994353437 11.80735492 11.44501485 10.00281502 9.797661526 10.24317398 10.73216743 10.38909352 11.45738088 10.23840474 9.463524373 9.726218159 11.49934763 10.2467406 9.905387005 8.861086906 11.2632692 11.70087316 10.65910396 11.17367714 9.943979914 11.32699117 12.27699674 9.554588852 10.52747701 10.41574177 10.87113518 11.22941969 11.22400167 10.75988818 11.09209641 10.36741475 10.77478706 11.03479896 11.15860969 10.04984855 9.914385132 10.11504365 10.90388185 10.41996018 11.72749507 11.83999107 11.92962828 11.22701419 11.0721326 11.68211676 10.75154406 9.426264755 12.02444712 11.40992124 12.32923574 11.05596023 11.80009099

ERCC2 10.59618976 10.16867212 11.48230378 8.820178962 11.46862404 9.810571635 9.603626345 10.37177664 10.35093918 9.513727596 10.62662165 11.70476824 11.16302064 9.379378367 9.413627929 10.07280253 10.44397954 10.2737956 10.63026713 10.40939094 10.59339112 11.00632608 10.40620501 10.74062404 10.60640521 11.04780555 10.71938882 9.573647187 12.24049322 11.92221272 10.76652891 9.71596199 8.980139578 10.76071995 11.93663794 9.063395081 8.503825738 11.15860969 10.53430288 11.11243951 11.04028972 9.616548844 9.647458426 9.057991723 10.24436384 9.62388149 9.837627933 9.980139578 10.75739001 10.51076417 10.06877828 11.29174628 9.071462363 10.41996018 10.712527 10.39016896 10.96722626 9.618385502 9.675957033 8.939579214 10.35645197 10.54592977 10.06069593 10.11243951 10.9248125 10.12282799 10.77971936 8.611024797 10.71080643 9.558420713 11.06474276 10.42836017 9.978710459 11.41996018 10.97513146 9.668884984 10.73555602 11.92999806 10.53235593 10.42836017 11.90876788 10.87728413 12.17274002 11.32530546 10.44397954

ERCC1 11.47522678 10.85486838 11.97010589 10.56510208 12.4228532 11.01402047 10.88111396 11.80735492 10.95274125 9.943979914 11.04848687 11.61700823 11.28019079 9.812177306 10.54496443 11.71553306 10.70130646 11.64160022 12.79197682 11.19967234 11.06810648 11.08148344 11.96253456 11.45891921 11.19905882 11.22037833 10.61470984 10.45943162 11.67683861 11.84509796 11.40726776 10.60917874 9.63481105 11.60640521 11.48280796 9.537218401 9.636624621 12.05934446 11.55074679 11.15671514 11.18239435 10.46147945 10.14210706 9.761551232 11.00982862 9.912889336 9.824958741 11.03823313 11.91587938 11.56890615 10.59898297 11.32192809 10.25384748 11.86069903 11.00982862 11.25384748 11.39606956 9.991521846 10.74230944 11.30549179 11.72877085 11.33539035 10.16741815 10.21188829 11.37232095 10.82336724 11.4314976 9.398743692 11.66088727 10.2911707 10.8008999 10.99647349 10.88417052 11.84901407 11.23361968 11.00912879 11.90876788 12.32108252 11.76487159 11.75028827 11.86843676 11.77190208 12.30263892 10.58777752 11.01471793

ERBB2 12.09967655 10.8864587 11.54496443 6.22881869 12.67617748 10.94982671 11.69043456 10.95564991 11.13570929 10.16490693 12.45224124 10.96722626 12.9436137 9.236014192 7.417852515 8.459431619 11.05120894 8.682994584 11.65999589 11.05188866 12.17523765 12.06440596 12.04234338 9.517669388 11.9225844 12.33566949 7.569855608 10.18735207 11.81137469 7.936637939 12.08081753 9.73809226 9.712527 11.77272695 12.60686784 9.815383296 10.1176431 12.50432245 10.84392105 12.15987134 10.99506047 10.86186234 9.926295995 8.348728154 10.82813648 8.491853096 6.22881869 9.754887502 11.5028318 10.4807902 10.70130646 10.76404222 9.236014192 11.90914305 10.70735913 11.60594244 11.88493365 9.575539247 6.672425342 7.087462841 11.19352536 10.66711154 11.48934624 10.66888498 10.75070699 11.98690862 11.88073144 9.5980525 9.169925001 10.22641219 12.05663772 10.97584797 10.94982671 11.436191 11.64745843 9.197216693 10.79116289 13.30591924 11.71080643 10.80251637 13.23586465 10.68474862 13.31755394 11.89216331 9.301496195

EPS8 11.77971936 10.97226185 11.85759229 12.47446646 12.43905168 11.74062404 12.42206477 11.3553511 11.53089398 10.65999589 11.56224242 10.83051521 12.05460432 10.03617361 10.78053977 11.76983784 11.43827206 11.65955 9.987264012 11.12605912 12.17148958 10.99929539 12.59175608 10.79603961 9.09011242 7.569855608 11.63888838 9.236014192 11.23242093 12.86747181 10.6635581 12.33371443 9.503825738 12.67198327 11.1959873 10.15860969 11.19167615 12.10230382 11.14656868 10.93516505 11.96542357 10.25620869 11.49585503 7.948367232 10.25738784 10.29232163 10.40832974 10.63026713 11.69653291 11.44553222 11.38963134 11.09671515 10.95782756 11.28828934 12 11.40673648 10.29576893 10.45018025 11.07481046 9.946906274 11.06743436 10.42206477 8.247927513 11.32867493 7.64385619 12.27146303 9.828136484 9.607330314 12.66466949 10.05392588 11.03411115 10.54978467 10.16741815 12.87094261 11.61378946 10.61470984 9.209453366 13.10787091 11.2039597 11.13699111 11.32305476 10.86959384 12.96884676 10.68387187 11.96614491

EPOR 8.519636253 7.864186145 8.689997971 6.727920455 8.816983623 6.977279923 9.14974712 9.709083813 7.199672345 6.569855608 8.375039431 8.781359714 8.550746785 8.055282436 7.22881869 8.164906927 7.383704292 8.092757141 9.057991723 7.569855608 8.724513853 8.189824559 11.84705735 9.631177056 7.754887502 5.129283017 7.321928095 8.451211112 8.252665432 7.77478706 9.335390355 8.209453366 7.46760555 9.221587121 9.592457037 8.894817763 9.871905238 8.857980995 8.224001674 8.199672345 7.076815597 8.727920455 7.238404739 5.977279923 8.21916852 7.924812504 8.714245518 7.491853096 7.876516947 9.301496195 8.74819285 8.118941073 7.06608919 7.651051691 9.581200582 6.857980995 7.930737338 8.417852515 8.353146825 8.912889336 8.033423002 9.856425529 7.417852515 6.459431619 7.50779464 8.787902559 8.438791853 7.531381461 8.194756854 7.658211483 9.177419538 7.569855608 8.936637939 10.6635581 8.781359714 6.426264755 8.515699838 8.804131021 8.252665432 8.988684687 9.147204925 9.28077077 8.960001932 7.74819285 8.724513853

EP300 10.06743436 11.30492167 12.99594377 12.03891899 11.20151134 11.10525378 11.06204614 13.0950673 11.59011917 10.35204343 12.37204883 13.08530647 13.25915477 11.11048331 11.41626974 11.81977993 11.57175264 12.25679839 10.60177079 12.06911406 11.7026056 12.60339453 12.99399979 12.80413102 11.19844504 9.519636253 11.63617144 9.605479518 10.58308277 13.38895904 12.91830423 11.50680344 10.63117706 12.48708634 12.44346161 10.4807902 10.34096276 12.57908018 10.88034881 12.25797706 12.19321732 11.30378075 11.2772874 9.605479518 12.77622739 11.30606169 11.26620091 10.08480839 11.47623991 12.82932634 11.25384748 11.53430288 11.23302043 12.47294463 12.62548055 11.7968508 10.89254282 10.90086681 11.83683936 12.08547246 11.34706766 11.09473751 11.4767462 12.0721326 9.975847968 12.1049263 12.42127591 9.59058705 10.34762137 11.04028972 11.47927503 10.30833903 12.03411115 12.93184554 12.64250303 11.35038674 10.50779464 12.0764816 11.79806672 11.9403136 13.8559391 11.77272695 13.98049664 11.47927503 12.67991988

EMD 11.712527 10.59991284 11.53430288 10.05256805 12.16616308 11.03273453 10.90312868 11.44449729 10.60177079 10.27496047 12.19844504 12.769218 11.55794229 10.09143539 10.60640521 11.30947635 10.54689446 11.31231575 11.71295682 11.57412044 12.31882522 10.89102419 11.98903964 12.66222331 11.42626475 11.20089861 10.77478706 11.80170836 11.64610864 12.18549492 11.52356196 10.96289601 10.36741475 11.08812569 11.66489167 9.924812504 10.8494051 11.8419574 10.78626963 11.37232095 10.46147945 11.14656868 10.55170826 9.727920455 10.68825031 11.2137118 9.84862294 10.98085361 11.64835758 12.26941893 10.51471405 11.78790256 9.707359132 11.3376219 11.64745843 10.69696753 11.86495992 10.35204343 10.28308835 11.52894242 11.17367714 10.90011206 11.24614677 9.810571635 10.64925618 11.47218312 11.40407714 10.71596199 11.08148344 10.70390357 11.84117119 11.19475685 10.78708632 11.14146856 11.51520703 10.74146699 11.39124359 12.97172316 11.3885555 10.80735492 12.47471995 11.63117706 13.54170165 11.20762447 12.68846888

ELN 6 8.810571635 8.632995197 9.946906274 8.784634846 14.50202373 12.04814625 14.25384748 8.54689446 14.59146391 10.23601419 11.86457308 12.23541594 8.076815597 7.087462841 11.30149619 8.108524457 11.78258876 7.409390936 9.698704667 10.98441846 10.63026713 14.25355206 8.016808288 7.988684687 7.584962501 13.53503231 10.78626963 11.76321237 10.52061868 9.710806434 12.6570951 12.03204573 12.53211237 9.761551232 10.99647349 12.97136392 9.951284715 9.874981348 11.29519496 10.49785184 11.6891244 11.05460432 8.257387843 9.426264755 8.873444113 10.55170826 5.459431619 12.38909352 9.717676423 11.81137469 7.098032083 12.40087944 11.76818432 12.5696183 12.17211493 11.10328781 9.45532722 6 8.707359132 9.700439718 12.10721708 5.426264755 7.599912842 6.87036472 8.861086906 11.49735289 11.98868469 11.53381639 8.335390355 10.43358544 10.87958325 8.28077077 9.463524373 14.26099302 11.35093918 8.194756854 14.22196466 8.550746785 7.882643049 12.0454184 12.41256985 9.562242424 12.66066448 10.11504365

EIF5A2 8.266786541 7.426264755 9.636624621 7.357552005 9.789533645 8.442943496 8.603626345 9.503825738 9.310612782 8.103287808 8.082149041 7.839203788 10.00982862 7.339850003 8.854868383 7.965784285 7.754887502 8.64385619 9.544964433 9.337621902 9.136991112 8.092757141 8.994353437 8.527477006 9.033423002 7.599912842 8.413627929 8.17990909 8.839203788 11.21977355 8.651051691 8.912889336 6.442943496 9.453270634 10.14338321 6.894817763 9.616548844 8.511752654 8.409390936 8.658211483 8.54689446 9.73809226 8.129283017 7.22881869 9.019590728 8.539158811 8.038918989 7.400879436 8.77148947 9.194756854 7.636624621 8.861086906 7.693486957 9.027905997 10.39553414 8.339850003 6.14974712 8.092757141 8.921840937 9.087462841 8.820178962 9.605479518 8.370687407 7.592457037 8.491853096 7.303780748 8.379378367 6.882643049 7.787902559 6.857980995 8.159871337 7.894817763 9.28077077 10.21188829 9.77807713 7.754887502 11.25738784 9.142107057 9.48984796 9.392317423 11.03686045 9.294620749 10.42206477 8.854868383 8.430452552

EGR1 8.558420713 12.15355203 14.96158535 12.17804233 13.29505143 10.30035256 11.25029842 13.29001885 13.63922764 12.36796071 12.94233122 14.46658634 12.72408746 12.74840253 12.60663654 12.00035218 10.66088727 11.68211676 13.66910651 12.05426514 13.39204904 10.62388149 12.90162116 14.13691103 13.14895316 9.162391329 10.6794801 9.501837185 11.40514146 11.05188866 12.40274562 11.13955135 10.68211676 12.16961188 10.23601419 13.58566677 11.18735207 13.50581155 11.71166697 11.39713981 13.04882741 9.773139207 11.55554777 9.095397023 12.00457162 12.80976813 10.22520744 11.79197682 11.80735492 12.02271457 10.73724734 10.54978467 10.09934781 10.55650605 12.57955165 11.32530546 12.34429591 10.87113518 11.45429929 14.90180968 11.82535634 12.04473563 13.18161817 10.43879185 14.41613777 12.29519496 13.79988869 9.820178962 8.554588852 14.33434313 12.92888844 10.65731845 12.97620609 14.98570855 12.67441298 12.16490693 10.04302728 12.61401961 11.5526691 13.04148804 12.80755618 11.20884399 14.67838007 10.46352437 14.44772899

EGFR 9.54689446 10.64835758 11.65642486 10.30263892 9.108524457 10.68562484 11.34318572 13.18812518 12.15450169 9.689997971 11.71338651 10.87498135 12.97888918 10.04712391 7.523561956 11.37558251 10.16867212 12.23391921 4.459431619 7.888743249 8.997179481 8.169925001 11.98299357 8.257387843 10.48179943 7.366322214 12.85096815 8.124121312 9.818582177 11.47370575 9.946906274 14.90576305 9.501837185 11.97835296 10.70908381 10.3376219 11.62159404 12.80654962 9.177419538 11.14784089 9.97441459 10.73301532 12.03994716 6.882643049 6.523561956 10.15860969 10.90162116 8.689997971 11.92740761 10.8963324 10.00140819 12.80130419 11.29576893 9.985841937 12.25355206 12.0406322 8.082149041 7.794415866 11.52160044 7.832890014 11.68737568 11.23182118 8.442943496 11.30320995 8.539158811 10.90463462 11.32418055 9.324180547 12.03032228 8.842350343 9.375039431 9.436711542 10.88034881 12.80715364 12.18208393 8.562242424 7.139551352 13.48091639 10.69522829 11.67815996 12.00316651 10.47978026 9.661778098 13.22761594 10.86031105

EGF 3.807354922 3.321928095 4.169925001 7.276124405 0 3.807354922 4 2.321928095 6.392317423 1.584962501 6.614709844 1.584962501 8.370687407 2 6.459431619 4.247927513 7.930737338 1 2.584962501 5.781359714 7.139551352 8.098032083 6.614709844 0 3.459431619 7.442943496 2.321928095 3.459431619 4.857980995 6.820178962 3.321928095 8.84862294 5.321928095 4.321928095 6.285402219 6.247927513 4.523561956 6.894817763 4.459431619 5.321928095 4.584962501 7.539158811 8.326429487 6.62935662 1.584962501 5.426264755 6.375039431 2.584962501 6.700439718 6.50779464 4.087462841 4.321928095 4.247927513 7.266786541 7.276124405 5.321928095 3.169925001 2 2 5.523561956 6.375039431 5.392317423 2.584962501 4 3.321928095 7.044394119 4 5.977279923 1.584962501 2.807354922 3.459431619 3.700439718 5.209453366 3.807354922 6.491853096 2.807354922 4.754887502 6.672425342 6.857980995 7.888743249 5.169925001 5.64385619 9.497851837 5.857980995 4.857980995

EFEMP1 9.019590728 7.483815777 8.707359132 7.960001932 12.63707766 10.47065887 9.556506055 7.554588852 10.97656412 9.601770788 10.69957245 10.39553414 11.19844504 6.754887502 8.276124405 9.283088353 5.781359714 5.209453366 9.681238412 12.02928723 8.820178962 7.400879436 11.70822173 10.71338651 8.45532722 8.335390355 5.584962501 9.147204925 10.93957921 7.266786541 5.209453366 6.832890014 7.813781191 10.28077077 6.285402219 6.303780748 6.044394119 12.01367161 7.523561956 11.42678889 8.353146825 9.552669098 11.42469119 1.584962501 11.48984796 9.842350343 7.700439718 6.375039431 11.41468524 7.022367813 7.515699838 7.960001932 8.611024797 10.95637616 12.4478581 8.471675214 9.807354922 6.409390936 8.294620749 6.781359714 11.98263713 3 4.95419631 4.523561956 9.896332404 10.75572215 8.918863237 3.459431619 3.700439718 6.658211483 5.906890596 8.124121312 10.55937709 10.30149619 11.42101286 9.541096615 8.366322214 13.080651 5.882643049 10.32867493 12.34401844 3.807354922 10.36194377 5.857980995 10.21916852

EEF2 17.57071551 15.75733792 17.92169568 15.2207184 17.20999587 16.44595245 16.47100832 18.22922721 16.29842062 17.27716933 15.29795514 16.57815153 17.06826396 15.90446999 15.65676002 17.05806573 15.66302989 16.65106571 17.4625502 17.24189007 16.0263073 16.92247988 18.55218876 16.07455962 16.10554026 17.44193912 16.53392282 15.98441846 16.96226342 18.51594618 16.93655743 16.77649729 15.03312184 17.32948103 16.75019666 14.38039342 16.43698801 17.11439305 17.17894684 15.88867191 16.02375435 17.14448897 15.31132259 14.38976576 16.44420608 15.8492096 14.89339634 14.94311001 15.95451441 17.1896412 15.11248023 16.47124653 15.75759836 16.92725713 15.55865987 16.27599715 15.80450919 15.60200286 16.66914804 15.28695477 16.40995438 16.78527365 16.71339994 14.89330153 16.38673821 15.91176647 17.18907167 14.72099235 15.82550541 15.13707119 17.36998918 16.57087854 16.10862655 17.29418097 18.34966998 16.34322042 16.77982193 18.31257722 17.48319385 17.35244868 18.01341537 16.43755703 16.94495222 15.79700284 16.69569048

EEF1E1 9.519636253 8.335390355 8.882643049 7.276124405 10.54012804 7.894817763 8.124121312 8.50779464 8.491853096 7.139551352 7.768184325 8.588714636 9.640244936 6.700439718 7 7.794415866 7.294620749 8.614709844 9.77148947 9.100662339 9.306061689 9.477758266 9.434628228 7.73470962 8.851749041 10.25856603 7.257387843 8.550746785 10.88417052 10.01541505 10.13314221 7.839203788 7.169925001 7.845490051 8.238404739 7.554588852 8.689997971 9.339850003 8.495855027 8.603626345 8.62935662 7.303780748 8.603626345 9.299208018 8.348728154 8.049848549 8.016808288 6.62935662 8.689997971 8.985841937 8.95419631 8.839203788 7.686500527 8.326429487 11.10131915 6.321928095 7.851749041 7.864186145 7.930737338 8.194756854 10.12282799 8.233619677 9.958552715 8.668884984 9.812177306 8.184875343 9.394462695 6.614709844 9.2644426 8.700439718 8.945443836 8.658211483 8.011227255 9.491853096 8.614709844 8 9.299208018 11.14974712 9.616548844 8.693486957 10.38909352 8.948367232 10.17990909 8.842350343 8.977279923

EEF1A1 17.43479924 16.73198188 18.59387252 15.89183116 18.39227549 16.02043744 16.49430872 18.27433265 17.12730923 17.24715798 16.33441297 16.94773966 18.39264026 17.37487816 16.37177664 17.98649204 16.32923574 17.63602271 19.08001334 17.51668495 17.07731645 17.63832985 19.297017 17.02531262 17.33728216 18.32914375 16.74770785 16.73162398 17.83159796 18.29072986 17.32448823 16.7603821 15.54200407 17.65887392 17.03889756 14.89292222 16.91074812 17.56766647 17.38170347 16.79761087 16.70567288 16.44674409 16.75086397 15.04648458 17.58087683 16.91755856 16.11315204 16.16655541 17.19716869 17.78201918 16.41369403 16.98606423 16.08187868 17.55883173 17.65652263 16.92512555 15.73068241 15.9778836 17.02348365 16.37320502 17.75209312 16.66810937 18.45067966 16.70395763 17.33817054 17.44523314 18.43084045 15.94727165 16.38759666 16.82692083 17.72877085 17.15545073 17 18.38076965 18.82511407 16.56915841 16.69166174 18.22345969 17.81390642 17.31689137 18.98598365 17.0050214 18.80162627 17.15279977 17.15456103

E2F1 10.94471206 10.17866485 9.079484784 10.10590851 10.88340699 10.19721669 10.53332973 9.878050913 9.4325419 8.413627929 10.9822806 10.79197682 10.82892983 8.413627929 10.51569984 9.62388149 10.00281502 10.65910396 11.39767463 10.60455323 10.17492568 11.51520703 10.06474276 11.36905201 10.51766939 10.51471405 9.722807531 11.00492268 10.55842071 11.41890673 11.47269084 8.794415866 9.328674927 11.19537221 10.87113518 9.99859043 10.32530546 9.541096615 9.854868383 10.50878516 10.53527538 10.80815977 9.558420713 9.394462695 11.07280253 10.39553414 8.682994584 9.13442632 10.63390341 11.45018025 9.579315938 11.42101286 9.233619677 9.727920455 11.45121111 9.172427509 10.4655664 9.727920455 10.54978467 12.18177344 10.29232163 11.46913302 11.09737377 10.09803208 10.03617361 11.34983409 11.02583167 10.28771238 11.00842862 9.859534786 11.08812569 9.705632387 11.57080444 11.35314683 10.99929539 9.874981348 9.807354922 11.91699905 11.29920802 11.20518233 11.70043972 9.978710459 10.99435344 9.652844973 11.32586758

DLL3 9.303780748 3.459431619 3.169925001 1.584962501 1 0 3.321928095 3.321928095 4 3.584962501 2 5.523561956 9.174925683 2 5.392317423 2 5 4.247927513 3.169925001 3 9.063395081 3.700439718 3.459431619 3.169925001 2.584962501 6.022367813 3.700439718 3.459431619 3.169925001 4.247927513 2 5.392317423 3.700439718 4.321928095 7.357552005 6.794415866 1 4 1 7.665335917 3.321928095 6.614709844 1 6.584962501 6.022367813 2 4.087462841 3.169925001 2.321928095 8.636624621 3.169925001 6 1.584962501 3 4.459431619 6.129283017 4.64385619 2.584962501 1.584962501 4.247927513 2 6.189824559 7.73470962 1 4.087462841 2.807354922 7.266786541 2.807354922 4.95419631 4.459431619 0 6.339850003 6.266786541 4.247927513 4.392317423 3 3.169925001 5.491853096 4.700439718 3.169925001 8.977279923 1 7.584962501 5.614709844 4.247927513

DGAT1 12.25532369 11.75739001 11.41151099 8.927777962 11.29174628 9.960001932 10.67771964 10.66088727 10.32642949 8.581200582 10.10459875 12.45968776 11.09209641 10.04439412 9.842350343 11.09671515 10.06608919 10.9708249 11.37937837 10.09803208 11.14656868 10.79360331 12.13731139 11.14210706 10.61654884 9.840777924 10.36850646 10.85018684 10.77971936 11.70476824 11.43514934 10.15355203 9.136991112 10.63843591 10.72366094 7.857980995 9.988684687 12.41256985 10.86263736 10.33539035 9.794415866 10.38478375 9.238404739 8.417852515 10.38909352 10.93295289 8.060695932 9.226412193 10.71767642 11.08878824 11.31628153 10.6926155 9.661778098 9.719388821 11.10131915 10.86805085 10.91064273 9.763212367 9.802516365 11.18797059 10.55362929 11.13314221 8.945443836 9.743151394 10.04028972 10.72024426 11.94141447 9.847057346 9.483815777 10.28771238 11.31854281 11.14529533 11.91811785 11.80534083 11.38370429 10.96000193 10.34318572 11.73470962 11.14082977 11.29863541 13.13875177 10.74567432 12.74251997 11.64340528 11.83447105

DDIT3 9.874981348 9.033423002 9.689997971 10.92184094 13.73819784 7.375039431 9.917372079 12.03891899 8.997179481 6.807354922 10.51274046 9.157346935 11.29001885 8.930737338 10.16239133 9.544964433 9.754887502 9.939579214 11.5980525 10.15734694 9.681238412 9.390168956 11.45738088 14.4494711 11.2737956 8.326429487 9.658211483 10.10721708 10.40087944 9.523561956 9.243173983 12.6836526 7.882643049 9.243173983 11.57506646 8.672425342 8.813781191 9.233619677 8.962896005 8.154818109 9.415741768 9.912889336 8.005624549 6.882643049 10.46862404 9.689997971 7.426264755 8.199672345 9.994353437 10.05663772 10.34318572 11.06944977 7.77478706 9.250298418 11.84156435 10.15101654 9.381542951 9.221587121 8.21916852 9.766528909 10.28540222 9.789533645 10.09407769 8.810571635 9.348728154 9.59058705 9.73809226 7.584962501 8.262094845 8.842350343 10.4767462 8.184875343 9.044394119 11.75154406 9.596189756 10.12928302 9.62935662 11.69783636 9.400879436 8.366322214 11.18053081 9.103287808 10.04439412 10.37829486 9.758223215

DBN1 13.50183718 13.05358654 13.19090494 9.702172685 13.85943772 10.3140167 12.24317398 13.48217771 12.9248125 12.00597536 13.27233818 13.56997425 14.57062658 11.21188829 11.05392588 12.48205163 12.61769703 11.85018684 12.42836017 12.03754695 13.58167136 12.78463485 13.372457 12.43540982 12.06743436 13.40341153 12.34429591 12.69631556 12.19075065 12.86476651 13.69588075 10.96217303 10.87190524 11.42574042 12.08181628 10.72962074 11.60686784 13.58378795 11.85486838 12.13506795 12.47319838 12.44320258 11.29462075 10.89784546 12.59175608 12.12120972 11.8837888 12.87036472 12.98013958 12.99258434 12.01436924 12.42992974 10.77889848 13.59175608 12.91195368 11.77190208 13.12976598 12.33371443 11.70130646 12.77930897 12.02583167 12.39473063 12.88264305 10.64565843 12.80433273 13.5020859 13.72344764 10.40832974 10.64565843 11.69913863 13.25841881 11.27786855 12.64498286 13.74283572 13.55362929 10.55650605 12.28626896 13.12379809 12.59549061 12.17679648 15.0444795 12.10328781 14.19198451 12.83644491 13.24644372

CTNNB1 13.68999797 14.29476432 15.22007597 14.00035218 16.0588796 12.95746485 13.98147809 15.14106935 14.58137714 12.71102162 14.42724736 14.42711639 15.21074743 14.0172434 14.26062556 13.88302507 14.29332795 14.32516489 14.95687524 15.92051569 14.17305246 16.5121386 14.87982253 14.03428313 15.44743843 13.277578 13.50432245 12.48331195 15.1704728 15.96131403 15.19175324 13.42665788 12.54375685 16.32921822 15.4893776 12.96776663 12.80534083 15.05930221 14.88049231 13.42639581 13.56641086 12.83368075 14.14306428 13.02410078 15.21503998 14.36365568 13.6209071 12.0871313 15.05256805 14.88907611 15.23290804 14.82605188 13.57919807 14.43567026 15.16101375 14.24740836 13.21143206 15.31967212 13.85087051 15.0104844 15.76090183 14.07689908 14.42377249 14.30506422 15.80680133 14.24829822 15.01872179 12.69130733 12.92072502 14.02756048 14.78647385 14.06297367 16.30031681 15.63956682 14.74288834 14.01741741 13.77930897 15.97280034 14.14712541 14.53478921 15.83925301 14.38976576 14.92939712 14.92202684 14.96090695

CTF1 4.321928095 4.247927513 7.22881869 5.882643049 0 3.169925001 6.14974712 5.906890596 5.554588852 3.807354922 7.22881869 6 7.876516947 3.169925001 4.169925001 3.700439718 4.584962501 7.73470962 4.857980995 4.321928095 6.700439718 6.820178962 8.640244936 4.857980995 4.523561956 2.807354922 5.523561956 3.321928095 4.754887502 5.882643049 6.321928095 5.169925001 6.189824559 5.554588852 7.820178962 4.807354922 2 7.942514505 2.807354922 8.049848549 5.392317423 4.087462841 6.357552005 3.459431619 3.321928095 4.700439718 4.321928095 6.14974712 5.459431619 5.129283017 4.807354922 6.50779464 6.189824559 4.807354922 7.851749041 7.055282436 8.873444113 4.087462841 2.584962501 3.584962501 8.339850003 6.409390936 1.584962501 6.820178962 3.459431619 5.459431619 8.113742166 5.554588852 6.95419631 5.357552005 4.169925001 6.658211483 4.807354922 5.700439718 4.459431619 2.807354922 2.584962501 2.584962501 4.807354922 1 8.164906927 4.392317423 10.12153352 8.527477006 5.426264755

CSNK1E 12.49335512 12.15196787 13.06861036 12.81057163 12.60432156 11.61378946 12.15987134 12.73386272 11.59712143 10.73809226 13.03290668 12.8837888 13.93498083 10.95346896 13.08131699 12.60663654 11.58072965 12.68299458 12.83308774 12.20701432 12.11666886 13.44009052 13.95419631 13.41508153 12.71810471 11.93369065 12.4767462 12.1582941 12.12992693 13.63015334 13.16254868 11.78463485 10.93663794 12.57364719 13.03600185 11.75070699 11.2366122 12.74945047 11.23122118 11.9854862 12.71317168 11.87728413 11.26737193 10.33985 13.20426546 12.67860014 11.37612539 12.22309669 11.82575383 13.17008154 12.12379809 12.67793982 11.74441341 12.31174832 13.07998471 11.9465408 12.54424001 11.65463603 11.45738088 12.31118066 12.97674311 12.14402087 12.19567979 12.28742381 11.87920032 13.13346335 13.30263892 10.97727992 12.26590801 12.18518517 12.26356264 11.5980525 12.54761755 12.99276135 13.01244995 11.39285404 12.36002402 13.00982862 12.75175325 12.0721326 14.31741261 12.17897601 14.04993361 11.91251514 12.97459384

CREBBP 11.64565843 12.29720288 13.14402087 12.10066234 12.11048331 12.67308819 12.6411486 12.79705352 12.05460432 11.82535634 12.11439305 13.53272118 13.86920825 10.74819285 11.40301202 11.74861218 11.93369065 12.52012755 11.66533592 12.39392668 12.58331787 12.16018658 13.79725622 12.65172443 11.95419631 11.15038197 12.2467406 10.51668495 11.41045135 13.43527959 13.27423254 12.19813805 10.85564717 12.66111003 11.95673915 11.6110248 12.07146236 13.03874756 11.16741815 13.3411018 12.66644594 11.8583696 11.37503943 9.625708843 11.96722626 11.62890115 11.17492568 11.15418521 12.53113774 12.91867693 11.66533592 12.20365389 12.18797059 13.21704891 12.10950422 11.77231457 11.12153352 11.86418614 11.31628153 12.63390341 12.33119688 11.6956633 11.94727165 11.73089465 11.24257869 12.01576349 13.08646799 11.8587581 11.07280253 11.3376219 11.80735492 11.09407769 12.37259303 12.99770875 13.760616 11.91849059 11.13635034 13.3047791 12.35562639 12.36057278 14.09934781 12.15576694 14.00921629 12.56890615 13.18626903

CREB1 10.25148241 10.23361968 11.91811785 9.900866808 10.53040634 10.25738784 11.0986901 10.712527 11.1692987 9.303780748 10.79116289 10.54689446 12.25532369 9.938109326 10.18735207 10.05528244 10.23601419 10.78463485 10.09934781 10.82892983 10.41679753 10.31854281 11.70606427 10.59339112 10.61930296 9.189824559 10.56224242 8.82336724 10.23361968 11.79400965 10.62021983 10.93442804 8.693486957 11.30947635 11.04302728 8.584962501 9.967226259 11.00982862 10.66799854 10.7968508 11.30720081 10.69522829 10.62113611 9.319672121 12.18580462 10.27496047 10.47978026 8.238404739 10.98655315 10.89784546 10.41996018 11.08214904 10.01262454 11.50878516 11.84901407 10.07547915 8.87036472 11.07881795 9.924812504 9.897845456 10.72280753 10.55074679 10.16364968 10.52356196 10.32755264 10.55554777 11.33539035 8.965784285 9.864186145 10.45532722 10.5028318 9.403012024 11.56985561 11.79928162 11.73089465 10.21067134 10.27612441 11.857981 10.39231742 10.4918531 12.48834228 10.99859043 12.57648435 11.15291858 10.90839262

COQ7 9.079484784 9.247927513 10.11894107 8.73470962 10.03066714 7.599912842 8.784634846 8.791162889 8.77807713 8.405141463 8.86727874 8.535275377 10.70043972 7.584962501 8.103287808 9.638435914 8.885696373 9.164906927 9.992938336 8.8008999 8.033423002 9.539158811 9.537218401 8.64385619 9.027905997 9.353146825 8.596189756 9.182394353 9.233619677 9.226412193 8.758223215 8.62935662 7.169925001 9.285402219 9.361943774 7.118941073 9.131856961 8.523561956 8.022367813 8.927777962 9.689997971 8.194756854 8.816983623 7.366322214 9.159871337 9.224001674 8.209453366 7.189824559 9.688250309 9.457380879 8.607330314 9.129283017 8.851749041 9.294620749 9.511752654 8.813781191 8.17990909 9.095397023 9.022367813 8.071462363 9.968666793 8.832890014 8.710806434 9.413627929 8.876516947 8.74819285 10.01820018 7.321928095 8.632995197 8.751544059 8.851749041 8.348728154 9.361943774 9.804131021 9.887220615 8.022367813 8.861086906 9.797661526 9.202123824 8.968666793 10.46658634 9.057991723 10.01122726 9.733015322 9.392317423

CNR1 7.74819285 7.918863237 9.962896005 3.321928095 8.930737338 0 3.584962501 7.475733431 3.584962501 3.169925001 6.285402219 6.266786541 9.033423002 5 5.700439718 4.523561956 9.398743692 2.321928095 1.584962501 2.807354922 5.285402219 3.459431619 4 5.209453366 9.908392621 5.247927513 3.321928095 5.554588852 2.584962501 11.27437815 6.614709844 0 2.584962501 7.491853096 2.807354922 3.584962501 5.321928095 3 4.906890596 8.77148947 6.209453366 3.584962501 5.700439718 5.857980995 1 6.409390936 0 2.807354922 3.321928095 6.392317423 4.459431619 0 3.459431619 4.700439718 7.330916878 2.807354922 11.02375435 6.794415866 4.807354922 5.554588852 9.695228291 0 5.285402219 5.087462841 4.392317423 6.599912842 8.939579214 2 2.584962501 3.321928095 5.857980995 3.584962501 1.584962501 7.383704292 4 4.95419631 2 3.169925001 3.321928095 6.820178962 10.00422047 6.22881869 4.754887502 3.700439718 5.584962501

CLU 14.28569119 10.87344411 10.37721053 10.41468524 13.86997933 10 11.75112558 10.57648435 10.57648435 13.32572707 10.16239133 11.509775 12.81338039 10.09803208 14.35059393 11.73428623 14.58953412 12.65015421 12.35974956 12.01715639 12.96506276 15.69558174 14.35459376 14.23399408 11.85057755 9.77148947 11.22400167 11.12863881 12.10525378 14.18518517 13.59979664 15.08381171 9.28771238 13.73534447 16.89544511 9.99859043 12.31118066 8.087462841 14.89458096 13.56854995 12.77375737 12.00105627 10.85642553 9.74819285 10.88187871 9.978710459 6.87036472 10.31741261 13.99814966 12.66088727 14.28467953 12.69718478 11.47725232 13.67463366 14.00579996 13.10705357 10.36850646 12.60802375 11.93369065 12.91886324 14.82520725 10.83526092 9.400879436 7.247927513 11.97835296 11.35590164 15.03570122 11.35700209 12.34651373 12.36440828 12.6926155 11.79725622 10.85330956 12.94672355 13.30563429 11.98013958 12.7700444 12.45224124 14.59152235 11.45635442 13.98468547 14.62256665 12.86263736 12.65127597 12.58824615

CLOCK 10.2227949 10.23242093 12.14082977 10.62479546 12.25177826 11.58918297 11.6128685 11.97871046 12.4807902 9.513727596 10.90463462 11.34040649 11.76942464 9.828136484 11.05731488 11.55314928 10.86031105 11.56414949 10.8917837 10.41890673 11.09539702 11.34096276 12.16804527 11.82376528 10.81137469 10.14847658 12.15987134 8.573647187 10.99364606 12.79156991 10.97154355 11.2227949 9.52160044 11.80170836 11.67683861 9.076815597 10.12670447 12.02167404 10.79603961 10.92035286 11.5863707 10.28886607 11.00562455 8.797661526 12.10983065 10.39767463 10.80896417 10.69348696 11.30263892 11.76071995 11.45943162 11.62205182 10.83999107 11.2137118 12.26590801 10.89860139 8.948367232 11.58730873 11.39767463 11.51323411 11.54834029 12.65620138 10.84470576 11.69130733 10.28771238 10.89481776 11.05120894 9.926295995 9.854868383 10.69696753 11.4604559 11.15987134 11.203348 11.92999806 11.35149141 10.02928723 11.20701432 12.43801209 11.14656868 11.09605624 13.09093942 11.06406908 13.01941698 12.32023645 11.93626986

CISD2 10.87574935 10.26795708 10.79522797 9.952741247 11.48984796 9.011227255 9.972979786 9.753216749 10.67330908 8.049848549 10.78463485 10.46658634 11.92109709 8.523561956 9.584962501 10.64295422 10.48884435 10.62570884 11.31854281 10.64565843 10.89405985 10.52649924 11.51569984 10.88417052 10.84549005 10.06474276 10.39874369 9.700439718 11.44138803 11.41943355 10.99717948 11.10066234 8.980139578 10.79928162 10.96144969 9.911391988 9.477758266 11.3858624 10.23959853 9.575539247 10.76901132 9.705632387 10.28886607 8.189824559 11.02859678 10.04848687 9.556506055 9.271463028 10.53527538 10.94178124 10.70735913 11.29174628 9.77148947 10.666224 11.79156991 10.81378119 9.266786541 9.865733271 8.74819285 10.13699111 10.78053977 10.75572215 9.859534786 10.73131903 10.30149619 9.843921051 10.65463603 9.481799432 10.38370429 10.65553072 10.79116289 10.21310422 10.64925618 11.21127995 10.52552081 9.936637939 11.18611424 12.80231441 10.33315535 10.41045135 11.13121391 9.878050913 11.55314928 10.75238065 10.96434087

CHEK2 8.599912842 8.124121312 9.092757141 7.924812504 9.77148947 7.357552005 8.982993575 9.21916852 8.224001674 6.832890014 9.658211483 8.503825738 10.18982456 7.851749041 7.118941073 9.656424863 8.54303182 8.562242424 8.686500527 8.434628228 8.592457037 9.06608919 9.784634846 9.071462363 8.321928095 9.413627929 8.581200582 8.885696373 8.724513853 8.764871591 9.142107057 7.906890596 7.50779464 10.20579325 10.01959073 7.924812504 7.700439718 8.082149041 8.632995197 8.082149041 8.611024797 8.668884984 8.569855608 7.426264755 9.807354922 7.894817763 7.965784285 9.036173613 8.361943774 9.828136484 8.326429487 8.885696373 7.044394119 8.243173983 11.28540222 6.727920455 8.97441459 7.754887502 8.603626345 8.463524373 8.861086906 8.189824559 9.278449458 9.583082768 8.016808288 9.712527 9.25502857 6.375039431 8.965784285 8.573647187 8.891783703 8.312882955 9.428360173 9.736401931 9.442943496 8.129283017 8.379378367 9.68474862 9.121533517 8.918863237 10.8587581 8.607330314 10.23361968 8.592457037 9.324180547

CETP 5.426264755 7.118941073 7.087462841 5.491853096 7.960001932 3.906890596 5.882643049 5.357552005 6.768184325 2.584962501 3.906890596 8.417852515 6.189824559 4.906890596 5.64385619 5.754887502 6.459431619 5.044394119 6.539158811 6.475733431 8.592457037 6.129283017 6.714245518 6.906890596 7.276124405 4.247927513 4.95419631 6.22881869 7.247927513 7.06608919 6.357552005 4.700439718 6.108524457 7.569855608 8.807354922 5.426264755 3.700439718 8.005624549 6.475733431 5.95419631 7.366322214 1 7.409390936 5.285402219 6.459431619 6.459431619 6.087462841 5.426264755 7 7.189824559 6.375039431 6.108524457 5.614709844 5.807354922 8.22881869 3.700439718 7.400879436 6.285402219 5.209453366 4.459431619 7.257387843 6.491853096 6.285402219 5.930737338 7.434628228 7.651051691 6.409390936 5.672425342 6.942514505 7.930737338 7.139551352 5.672425342 6.14974712 7.74819285 6.189824559 5.044394119 6.523561956 6.426264755 4.857980995 2.584962501 6.832890014 4.906890596 7.539158811 6.614709844 7.672425342

CEBPB 11.39231742 12.13506795 12.60269887 11.30492167 10.16113188 10.49085088 10.509775 13.80433273 11.96325736 10.72536626 11.93553341 13.51002236 11.34928123 10.20701432 9.531381461 11.25029842 11.22520744 11.45738088 11.54206454 10.69957245 12.33063682 11.37883671 12.74230944 12.63503787 12.074476 9.759888183 10.14210706 11.59898297 10.56605404 10.81618368 12.38073161 10.1176431 9.398743692 11.57884439 12.72770778 12.60455323 11.12734954 11.37612539 9.878050913 10.67330908 11.43931146 10.87036472 10.62296694 9.098032083 11.01680829 12.44863257 10.60640521 10.96722626 11.56652978 11.83091128 11.31854281 12.22158712 10.61562964 11.07146236 11.07614753 10.58871464 12.35810171 9.933690655 11.1234748 11.90011206 10.45943162 12.9627153 10.47775827 9.172427509 10.69957245 11.53138146 13.15228484 10.70649602 10.03754695 10.48984796 12.19105921 10.48280796 11.45018025 12.77458118 11.24079133 10.28077077 11.72024426 10.53430288 13.04046097 11.16239133 12.72643106 11.32474311 11.66444728 11.1692987 12.70606427

CEBPA 8.977279923 10.02375435 9.724513853 5.672425342 8.882643049 6.845490051 7.700439718 10.14465824 9.955649908 7.577428828 9.681238412 10.79197682 8.076815597 5.321928095 6.06608919 8.74819285 9.226412193 7.87036472 5.832890014 9.044394119 10.04165915 7.87036472 8.77807713 7.451211112 6.882643049 6.700439718 9.769837844 6.832890014 9.247927513 6.794415866 9.197216693 8.562242424 7.584962501 7.434628228 10.32642949 5.882643049 4.700439718 8.584962501 7.400879436 9.430452552 7.554588852 6.894817763 10.04165915 6.426264755 8.312882955 9.611024797 5.044394119 7.139551352 8.375039431 7.46760555 9.64385619 8.321928095 8.636624621 7.864186145 11.95128471 7.857980995 8.700439718 7.864186145 5 6.686500527 8.049848549 9.233619677 5.906890596 7.375039431 7.383704292 9.485829309 10.61470984 8.348728154 7 8.285402219 8.535275377 8.77478706 8.139551352 10.65910396 8.321928095 6.988684687 9.854868383 10.09934781 7.238404739 7.864186145 10.48482289 8.379378367 9.577428828 9.283088353 9.677719642

CDKN2B 8.430452552 6.658211483 10.56224242 7.857980995 8.599912842 10.03479896 10.36522885 10.06743436 10.67595703 7.965784285 4.523561956 9.845490051 9.4325419 5.209453366 9.403012024 9.62388149 6.50779464 10.00562455 8.686500527 10.2227949 8.28077077 10.70390357 9.596189756 9.019590728 7.948367232 7.22881869 10.00702727 7.73470962 9.353146825 12.10197567 9.6794801 10.6183855 6.303780748 10.47167521 8.290018847 6.832890014 10.45635442 10.34429591 6.247927513 8.665335917 9.098032083 10.08480839 8.129283017 6.50779464 9.887220615 7.936637939 9.749869427 5.554588852 10.33985 10.22881869 10.10852446 9.573647187 8.266786541 9.812177306 10.14720492 9.693486957 10.40832974 10.6926155 5.044394119 7.864186145 9.495855027 9.422064766 7.912889336 9.005624549 9.411510988 9.627533884 10.1711768 8.515699838 4.807354922 9.784634846 10.36741475 7.936637939 10.13699111 8.103287808 9.763212367 10.24317398 9.479780264 9.828136484 9.529430554 8.471675214 10.70303839 10.26091953 9.269126679 10.59245704 10.57364719

CDKN2A 12.24287637 6.599912842 8.988684687 12.12379809 11.90914305 13.07296997 12.95909634 11.0409746 13.3274123 10.65999589 5.426264755 13.47826403 11.22941969 4.754887502 11.03686045 5.491853096 5.392317423 12.53794636 12.89519657 12.53235593 11.40779885 13.01402047 13.36741475 11.6956633 11.29059489 13.49997042 11.63026713 13.05375622 12.50952761 14.5383102 11.94617524 13.1221809 6.06608919 12.35066299 11.26561505 10.77066389 12.05596023 13.1135794 3.321928095 11.93590168 12.59058705 13.44151772 10.85642553 10.70303839 13.07280253 12.1205619 12.40354468 5.930737338 12.99647349 13.64846994 12.69631556 13.33119688 10.82416321 11.40087944 12.4807902 13.0877943 13.43840202 12.47269084 6.727920455 12.96668569 12.03891899 13.68376224 12.6188443 10.92777796 11.891404 11.78994113 7.46760555 12.31826034 5.459431619 11.81658371 13.15987134 11.96866679 12.36331346 8.214319121 14.00869122 11.60733031 13.10623576 13.6217085 13.82485932 12.38208359 13.11536884 11.53430288 12.87229011 13.09011242 13.95900575

CDKN1A 11.02928723 11.4604559 14.97428014 13.00912879 13.15624112 11.75655632 10.79766153 11.73555602 12.27815903 10.01541505 11.50382574 12.93258387 12.88054013 11.47725232 11.33483193 11.75070699 12.59851781 12.00667672 14.47744207 12.86244364 12.28424575 13.8094667 13.81217731 11.89368074 13.03634535 7.592457037 9.656424863 11.77396337 13.06187743 12.88302507 12.96144969 11.67771964 10.71080643 13.59642273 11.4429435 12.06069593 11.83051521 11.16176174 10.83130724 12.55218876 13.59502432 11.51422091 11.41837972 10.59152235 12.75634783 12.95201316 10.32530546 12.91232801 12.78524811 13.55146795 11.22520744 11.83209885 10.51076417 12.26356264 11.91774501 10.29347165 11.00140819 12.94141447 12.29519496 12.1959873 13.17882044 12.80493767 12.13217838 10.77971936 12.68671937 11.49635449 13.15180936 11.79968636 11.85759229 12.22309669 11.57364719 9.721099189 11.77930897 12.68540583 11.70822173 11.90501086 11.90275194 14.9677216 11.8181827 12.18146288 13.13795173 12.16302064 12.4003458 10.41996018 12.60200286

CDK7 9.575539247 8.577428828 10.25620869 7.483815777 10.19844504 7.383704292 8.721099189 9.301496195 9.607330314 8.21916852 9.100662339 8.214319121 9.854868383 7.531381461 8.14974712 8.86727874 8.87958325 9.640244936 10.12153352 9.447083226 8.77807713 9.978710459 10.53235593 9.573647187 9.6794801 8 9.656424863 8.851749041 9.531381461 9.961449694 8.836050355 8.903881846 7.832890014 10.0768156 10.45635442 7.813781191 8.005624549 8.312882955 9.368506462 7.781359714 9.033423002 8.103287808 8.658211483 7.918863237 9.339850003 8.826548487 8.118941073 8.209453366 8.991521846 10.45943162 8.73470962 9.688250309 7.409390936 8.689997971 10.85018684 7.577428828 8.071462363 8.721099189 8.618385502 6.754887502 9.290018847 8.539158811 9.400879436 7.392317423 9.862637358 9.353146825 9.703903573 7.14974712 8.451211112 8.405141463 10.17492568 8.614709844 9.54689446 10.09011242 9.405141463 7.761551232 9.703903573 10.32867493 9.982993575 8.903881846 10.02236781 8.891783703 10.01680829 9.269126679 9.459431619

CDK1 10.41468524 9.505811554 9.763212367 9.527477006 11.62113611 9.118941073 10.97584797 11.1401907 10.03754695 8.804131021 11.3858624 10.04165915 10.64565843 9.187352073 10.06069593 9 10.47370575 10.29691621 10.54785851 11.36741475 11.67595703 11.23840474 11.06204614 10.75070699 12.80231441 10.76652891 9.447083226 10.15734694 10.88187871 12.33483193 11.70865284 10.0768156 8.957102042 11.31288296 10.52454172 9.47370575 11.07948478 10.32530546 10.76818432 11.02444712 11.23062093 10.91513245 10.63843591 9.209453366 12.22972009 9.428360173 9.784634846 8.303780748 10.99929539 11.41732512 10.84392105 11.50432245 9.447083226 9.990103964 11.86534664 8.611024797 9.417852515 10.27844946 11.18239435 10.63662462 11.15924065 10.58590145 11.74146699 11.44086917 10.23242093 10.71767642 10.19352536 8.139551352 11.01680829 9.946906274 11.83486604 9.746514321 12.33650656 12.77704979 11.64205169 11.3858624 10.34096276 12.79725622 11.29519496 11.00702727 11.72195361 9.885696373 12.21310422 9.364134655 11.44397954

CDC42 13.12072388 13.33301555 14.57423872 13.51693112 13.83298888 13.17648485 13.70800613 13.81858218 14.5195134 12.0657527 13.54049133 13.80180938 14.52888139 12.35287105 12.44966454 13.45365647 13.41005379 13.84960057 14.37924297 14.02643699 13.89453359 13.97862109 14.27503325 14.22302125 13.43045255 12.42206477 13.3379006 12.47952767 14.02833777 14.31946044 13.73301532 13.81898154 12.38720958 14.76632185 15.1074623 12.22400167 13.00772811 13.95174004 13.52307183 12.98513037 14.46371594 13.48167332 13.75175325 11.00281502 14.1155314 13.54460227 13.02686921 11.67021364 13.50989869 14.40494196 13.77386037 14.54924319 13.03926179 13.56724311 14.90886168 12.91923579 12.89954574 13.19660213 12.75551354 13.42337858 14.78570788 13.480664 12.87670878 13.67275681 13.15513445 14.29741785 14.10246786 12.13570929 13.74115094 12.93129155 14.06693007 12.9918761 13.37490363 14.69201607 13.80120313 13.04899765 13.40061264 14.83644491 13.62548055 12.6163191 14.51193792 13.60883234 14.62986885 13.83890845 13.96821678

CCNA2 10.56224242 9.560332834 9.805743872 9.949826711 11.89595389 10.23242093 10.66533592 11.0721326 10.62021983 9.259743264 11.77107674 10.1711768 12.27175481 9.652844973 8.682994584 10.38370429 10.95710204 10.24436384 10.53915881 11.3459596 11.23302043 10.37721053 11.56652978 10.80735492 10.94397991 10.5943246 10.48582931 10.40087944 11.09077405 12.669328 11.3586512 10.27262978 9.579315938 11.81938079 10.89708913 10.02513956 10.59152235 11.91288934 10.66888498 10.82416321 11.02928723 10.97010589 10.57648435 8.285402219 12.03032228 9.945443836 10.70217269 9.689997971 10.83368075 11.76777065 10.42940674 11.48431942 9.832890014 10.09275714 11.15291858 9.603626345 8.930737338 9.847057346 9.430452552 11.15291858 10.13699111 10.6635581 11.06743436 11.23481743 9.908392621 10.61378946 10.32192809 9.306061689 10.09934781 10.39016896 11.94580958 11.1376316 11.2179577 11.82177398 11.25561875 10.31741261 10.73470962 13.10901442 11.39820926 11.6128685 12.09374766 10.17866485 12.46147945 10.74986943 11.44914865

CAT 10.31741261 10.33650656 11.99964774 9.776433032 11.945078 9.335390355 9.829722735 11.23361968 11.06676193 9.688250309 8.475733431 10.29462075 11.14656868 9.430452552 8.527477006 9.850186838 9.700439718 10.98085361 9.52160044 11.22400167 10.26209485 11.15228484 12.23989682 9.851749041 10.49984589 9.169925001 9.972979786 9.079484784 10.69609817 11.57837269 10.52552081 10.47573343 9.022367813 11.95782756 11.64790807 8.214319121 10.94836723 11.69217958 9.370687407 10.29347165 10.8273427 10.03617361 11.32699117 7.442943496 9.5980525 9.627533884 9.337621902 8.471675214 10.72109919 10.95855272 10.22520744 11.27786855 9.952741247 10.96144969 11.41943355 10.21674586 9.17990909 10.02928723 10.02097994 9.485829309 11.04644195 10.98085361 9.461479447 10.55458885 10.10459875 10.59152235 11.03411115 8.82336724 10.60362634 9.773139207 10.92407019 10.44086917 10.12928302 11.22037833 11.91587938 8.422064766 10.01262454 11.17367714 10.38154295 10.08081753 12.42678889 10.60177079 11.83605036 10.51175265 11.10590851

CACNA1A 6.475733431 7.118941073 7.475733431 5.930737338 5.321928095 5.672425342 9.505811554 8.442943496 7.77478706 4.64385619 8.45532722 10.4604559 10.69348696 5.209453366 7.832890014 8.86727874 8 7.22881869 7.022367813 6.820178962 7.906890596 7.169925001 9.871905238 7.375039431 4.807354922 4.906890596 8.562242424 6.06608919 5.977279923 6.475733431 6.50779464 8.033423002 5 8.710806434 9.049848549 8.861086906 9.419960178 8.640244936 6.977279923 8.686500527 8.055282436 9.8008999 6.87036472 7.74819285 7.569855608 6.339850003 6.857980995 8.700439718 6.108524457 8.139551352 8.194756854 6.857980995 6.95419631 5.584962501 9.221587121 9.727920455 7.693486957 6.820178962 7.348728154 8.909893084 7.8008999 8.761551232 9.515699838 5 5.727920455 7.876516947 8.487840034 9.451211112 9.038918989 7.209453366 8.714245518 6.22881869 4.584962501 8.741466986 12.60153868 5.491853096 9.670656249 4.087462841 5.169925001 9.231221181 11.1234748 8.174925683 9.381542951 8.087462841 6.459431619

C1QA 14.75389573 13.53247769 13.7856568 9.095397023 12.10164745 8.392317423 11.46199095 13.40087944 14.20373035 7.906890596 11.1234748 14.09481996 9.596189756 8.400879436 9.674192268 12.36905201 12.42259043 11.09143539 9.908392621 13.42140741 13.36536557 11.48381578 11.94068065 10.39016896 11.18673329 10.74986943 10.82177398 12.05154884 12.50804233 10.56890615 11.00492268 10.57175264 12.20640391 10.33985 15.04217235 7.77478706 8.413627929 11.16239133 12.13153547 10.45532722 9.68474862 8.988684687 14.29073886 8.689997971 11.29576893 12.66511381 9.098032083 11.99116751 11.3140167 10.19352536 12.03720374 12.55985504 10.15228484 11.78586111 12.33594857 10.39016896 14.12702704 11.99506047 7.900866808 10.35645197 11.89330153 12.90011206 9.8008999 9.938109326 11.70433597 13.78043724 14.06852639 7.451211112 12.75405237 11.80493767 12.46480098 12.33371443 11.18363538 14.81723351 10.45429929 10.59152235 13.67154107 10.22761594 9.942514505 7.434628228 13.29935114 11.62021983 12.90670273 11.30663136 13.81698362

BUB3 11.47167521 10.9432474 12.42914517 11.03754695 12.90651484 10.25384748 10.5372184 12.47040468 11.54158066 10.56795608 12.25236977 11.74230944 13.25532369 10.43671154 11.29404631 10.93295289 11.50878516 11.64205169 12.18487534 12.38936246 12.28366717 12.78176951 12.54351522 11.68299458 12.02652344 11.43201985 11.14082977 11.23122118 12.2822197 13.73174329 11.51076417 11.63934071 10.52258153 12.54303182 12.48482289 10.67595703 10.53915881 12.08048446 12.17180229 11.65194861 12.52405192 11.54206454 11.32418055 10.64205169 13.24584977 11.25797706 11.19044202 10.32642949 12.06069593 12.17586138 11.75988818 12.47750532 10.57080444 12.19260105 14.23847938 11.07614753 11.09473751 11.43879185 11.51471405 12.90463462 12.45943162 11.99329224 12.77540452 12.07146236 11.88950396 11.7874945 12.12960501 11.28135051 11.6379833 11.19721669 12.90858026 11.57412044 13.03789009 13.47801117 13.26458921 11.81177606 11.76859788 13.93276839 12.12734954 12.73216743 12.84058125 11.04371086 13.43462823 11.1692987 12.25738784

BUB1B 10.96722626 9.328674927 9.13442632 9.434628228 10.857981 10.18611424 9.799281622 11.7619667 9.885696373 9.131856961 11.99081308 10.12023788 11.43201985 8.632995197 9.348728154 9.903881846 9.45532722 9.632995197 10.63662462 10.97799537 10.31174832 10.75655632 11.97799537 9.584962501 10.45635442 10.86959384 9.693486957 10.38801729 10.53138146 12.48884435 11.24733418 9.491853096 8.30833903 11.29576893 10.17367714 9.394462695 10.08613623 10.42940674 9.712527 11.01402047 11.78586111 10.09143539 9.189824559 8.754887502 11.27554256 8.918863237 9.62935662 7.434628228 10.57553925 11.92332749 10.25148241 11.02652344 9.799281622 9.810571635 11.24079133 7.977279923 8.845490051 10.18363538 10.08878824 10.36632221 10.45943162 9.709083813 10.92109709 11.67551604 9.681238412 10.05528244 9.87958325 9.292321633 9.33315535 9.627533884 10.55074679 10.01541505 11.46454575 11.39767463 10.71938882 10.07414146 10.14338321 12.93221475 11.16741815 10.87498135 10.91513245 9.751544059 12.14561378 10.33315535 10.91363743

BSCL2 8.957102042 8.8008999 9.862637358 7.46760555 9.194756854 7.700439718 8.495855027 10.14465824 8.405141463 6.832890014 8.569855608 8.845490051 8.049848549 7.339850003 8.330916878 9.06608919 8.243173983 9.37286506 9.269126679 9.157346935 7.87036472 9.503825738 9.921840937 9.385862401 9.204571144 8.129283017 8.21916852 8.174925683 8.271463028 10.43983088 9.071462363 8.194756854 7.169925001 9.933690655 9.920352855 6.807354922 8.108524457 9.116343961 8.924812504 7.741466986 8.169925001 8.994353437 8.13442632 8.016808288 9.139551352 8.8008999 7.754887502 7.539158811 8.960001932 9.485829309 8.632995197 9.328674927 7.22881869 9.601770788 9.839203788 7.499845887 7.076815597 8.285402219 8.73470962 7.312882955 8.839203788 9.315149562 9.729620744 8.891783703 8.055282436 9.144658243 9.224001674 8.087462841 9.5980525 7.599912842 9.002815016 7.977279923 8.243173983 9.022367813 9.686500527 9.060695932 9.157346935 9.422064766 9.5980525 9.113742166 10.54592977 9.868822555 9.717676423 9.095397023 10.54496443

BRCA2 8.082149041 8.459431619 9.681238412 7.46760555 8.471675214 8.523561956 8.625708843 9.079484784 8.74819285 8.092757141 9.216745858 8.682994584 9.008428622 8.982993575 8.816983623 8.447083226 9.294620749 8.511752654 8.312882955 9.370687407 9.240791332 8.672425342 9.695228291 8.888743249 9.400879436 8.625708843 8.379378367 8.577428828 8.257387843 10.49785184 9.036173613 9.044394119 8.247927513 10.31514956 9.86727874 7.930737338 7.6794801 8.980139578 8.689997971 8.810571635 10.28193003 8.632995197 8.832890014 8.447083226 10.31854281 8.768184325 8.479780264 7.426264755 10.32530546 11.06069593 7.971543554 8.005624549 8.262094845 8.73470962 8.86727874 8.290018847 7.826548487 8.965784285 9.278449458 10.42416629 8.438791853 8.581200582 8.876516947 10.23840474 8.071462363 8.960001932 8.8008999 7.434628228 8.744833837 7.918863237 9.453270634 7.813781191 9.726218159 10.07414146 8.611024797 8.471675214 7.554588852 10.16867212 9.579315938 10.06608919 10.37937837 8.471675214 10.36413466 9.108524457 9.74819285

BRCA1 9.695228291 9.815383296 9.686500527 8.46760555 10.30947635 9.688250309 9.971543554 9.100662339 9.344295908 8.813781191 10.59991284 9.625708843 11.06541613 8.238404739 8.816983623 10.50680344 10.67154107 10.44397954 9.874981348 10.67507492 9.240791332 11.30263892 10.85564717 9.550746785 10.1176431 9.942514505 11.16302064 9.052568051 10.16364968 11.64835758 10.75405237 10.74567432 8.28077077 11.01122726 11.35974956 8.618385502 11.50928017 9.709083813 9.398743692 10.04302728 11.02236781 9.182394353 9.164906927 8.731319031 10.42731284 9.164906927 9.649256178 8.400879436 10.83130724 10.71338651 8.948367232 10.75822321 10.07146236 9.95419631 10.7548875 9.469641817 8.569855608 8.885696373 9.515699838 10.42626475 9.087462841 11.58355293 10.31288296 9.988684687 8.918863237 9.943979914 9.449148645 8.366322214 10.05256805 8.686500527 9.714245518 9.535275377 10.2737956 10.78626963 10.60733031 9.48984796 8.807354922 12.0871313 10.99717948 10.26678654 11.48280796 9.22881869 10.70303839 10.59618976 10.45738088

BMI1 10.37068741 10.08214904 11.98263713 10.24555271 11.6343573 10.46760555 11.07012094 11.33985 10.76901132 8.857980995 9.99859043 12.23720996 11.79197682 8.375039431 10.10590851 9.396604781 9.828136484 10.97513146 11.33427329 11.22037833 11.47927503 11.55314928 10.94909716 12.02928723 10.906139 10.37503943 10.04165915 9.121533517 10.55554777 12.28800089 11.05663772 11.03891899 7.787902559 11.37232095 11.69609817 8.861086906 10.92999806 11.1382718 9.712527 11.45584091 10.66533592 11.31004468 10.46352437 9.154818109 12.12120972 10.48784003 10.42416629 10.56510208 10.81778312 11.27903014 10.77313921 9.908392621 9.714245518 11.13057056 11.13891172 10.81778312 9.915879379 10.77725532 10.91513245 11.12863881 12.40832974 11.21977355 11.15671514 10.81618368 10.8153833 10.53332973 12.14465824 8.918863237 10.9248125 9.990103964 10.69783636 9.584962501 11.05934446 11.7232343 11.66799854 10.57270023 10.39553414 12.01192607 9.980139578 10.56890615 12.88626815 9.945443836 12.64182597 10.07414146 11.59991284

BLM 9.826548487 7.918863237 8.400879436 6.087462841 9.285402219 8.661778098 8.930737338 7.721099189 8.344295908 7.266786541 10.06608919 9.413627929 10.78626963 8.027905997 8.727920455 9.113742166 9.519636253 8.842350343 9.164906927 9.781359714 9.758223215 8.820178962 9.245552706 9.236014192 9.076815597 10.13699111 9.839203788 9.28077077 8.939579214 10.07146236 9.659995892 8.885696373 7.46760555 10.12282799 9.902375114 8.370687407 9.084808388 8.550746785 10.28771238 9.139551352 10.01402047 7.832890014 8.400879436 8.503825738 10.68387187 9.2644426 8.797661526 6.087462841 10.23122118 10.53040634 8.22881869 9.469641817 7.826548487 8.335390355 10.18363538 6.87036472 8.640244936 8.689997971 8.139551352 9.652844973 9.247927513 8.257387843 9.233619677 9.278449458 7.948367232 10.357552 9.434628228 6.794415866 8.447083226 9.105908509 11.30606169 8.108524457 10.53430288 9.656424863 8.370687407 9.797661526 8.686500527 10.17367714 9.157346935 9.434628228 9.722807531 7.330916878 10.56033283 9.62388149 10.82654849

BDNF 8.379378367 7.357552005 5.95419631 7.577428828 11.48733762 3.169925001 5.087462841 6.686500527 5.930737338 5.781359714 8.108524457 6.375039431 8.370687407 5.95419631 8.154818109 7.022367813 7.409390936 7.087462841 8.724513853 10.78053977 6.108524457 9.596189756 6.044394119 9.118941073 8.751544059 7.199672345 6.339850003 5.523561956 8.668884984 10.87267488 8.409390936 5.129283017 3.906890596 10.56033283 10.97297979 6.285402219 5.700439718 6.339850003 6.459431619 7.044394119 6.614709844 7.54689446 6.14974712 6.209453366 7.741466986 6.794415866 8.317412614 6.894817763 7.247927513 8.475733431 6.321928095 4.523561956 6.475733431 3.584962501 8.209453366 5.247927513 3.906890596 10.82654849 5.807354922 4.857980995 7.894817763 7.303780748 9.422064766 6.523561956 9.97441459 7.426264755 9.491853096 6.392317423 5.754887502 6.339850003 8.942514505 5.129283017 9.509775004 9.781359714 5.169925001 6 9.14974712 5.285402219 3.906890596 6.392317423 9.22881869 4 5.044394119 5.209453366 10.07414146

BCL2 8.033423002 8.668884984 10.60825482 9.060695932 9.645658432 9.236014192 9.5980525 10.33650656 9.084808388 4.584962501 9.236014192 10.59712143 9.978710459 8.794415866 7.159871337 9.375039431 10.02513956 8.573647187 8.164906927 9.465566405 8.930737338 9.063395081 9.620219826 8.016808288 9.172427509 6.266786541 9.321928095 5.781359714 9.981567282 10.50382574 9.908392621 8.055282436 7.741466986 10.01262454 10.93295289 7.614709844 9.759888183 8.8008999 8.957102042 10.55074679 10.24911345 8.611024797 9.810571635 7.266786541 8.087462841 9.312882955 8.918863237 7.199672345 8.271463028 10.07547915 10.215533 9.071462363 9.982993575 8.317412614 9.501837185 7.74819285 8.531381461 9.396604781 7.918863237 7.238404739 10.93737382 8.721099189 7.64385619 9.257387843 8.930737338 8.980139578 9.700439718 9 8.129283017 8.503825738 10.60547952 7.942514505 9.154818109 10.04712391 10.11504365 9.769837844 8.924812504 10.79441587 7.912889336 8.62935662 11.87152026 9.144658243 9.714245518 9.501837185 10.51175265

BAX 11.31004468 10.91587938 12.53576138 10.40620501 11.8004955 9.936637939 10.34429591 11.27903014 11.1382718 7.73470962 11.06608919 11.34207467 11.86766485 9.182394353 10.52454172 10.79197682 11.11374217 10.87113518 12.05358654 11.06944977 10.96578428 12.02928723 11.30606169 11.05731488 11.53089398 11.09275714 10.38370429 11.03479896 12.01192607 11.92703717 10.86108691 10.4767462 9.487840034 11.54978467 12.27058734 9.733015322 9.082149041 10.40939094 10.6329952 9.878050913 11.57270023 10.39553414 10.96578428 9.917372079 10.62570884 11.2848241 9.398743692 9.35974956 10.93737382 11.64610864 10.84313591 12.23631323 9.537218401 11.38262403 12.22128502 9.871905238 11.45635442 10.29001885 10.23601419 9.627533884 11.58824615 12.02340784 10.05799172 9.746514321 11.28250931 11.26385602 12.35342254 9.426264755 11.28886607 10.90388185 11.50581155 10.84627391 10.11374217 12.60269887 10.99647349 10.32305476 11.91513245 12.10787091 11.31797782 10.80251637 12.25915477 11.52747701 11.39660478 10.49485558 11.94580958

BAK1 10.82416321 10.41996018 11.8474489 9.596189756 11.2179577 9.670656249 11.03754695 11.66888498 11.28250931 8.994353437 10.70994538 11.25856603 10.89784546 9.194756854 9.434628228 9.743151394 9.861086906 10.52845411 11.62844554 11.07748336 11.04780555 10.81378119 11.05528244 9.73978061 10.37395266 9.981567282 10.05934446 11.01959073 11.30092449 11.52552081 10.94251451 9.77478706 9.238404739 11.54930337 10.73470962 8.668884984 9.625708843 10.41890673 9.493855449 9.361943774 11.01402047 10.8153833 10.18487534 8.118941073 10.97799537 10.30492167 9.537218401 8.73809226 10.10983065 11.59105477 10.38801729 11.71724801 9.73809226 11.08347933 11.06272077 9.736401931 10.83605036 10.09934781 10.12023788 10.12412131 10.98726401 11.21067134 10.12282799 9.315149562 10.33315535 10.44397954 11.19475685 9.337621902 9.766528909 9.967226259 10.83368075 9.71596199 9.741466986 11.36577564 10.62570884 9.503825738 10.97154355 11.08015131 10.78790256 10 11.8864587 10.95855272 11.36959735 10.81618368 10.99788513

ATR 9.139551352 9.764871591 11.34096276 8.479780264 10.46658634 9.477758266 10.17242751 9.764871591 9.939579214 7.409390936 9.22881869 9.923327485 10.79766153 8.832890014 8.951284715 9.25502857 9.579315938 9.914385132 9.87036472 10.93295289 10.10066234 10.78871833 11.00422047 9.659995892 10.06069593 9.479780264 9.933690655 8.405141463 10.56985561 10.7984718 9.936637939 9.73470962 7.238404739 11.66888498 9.982993575 8.661778098 10.66888498 9.812177306 9.259743264 9.908392621 9.46760555 9.920352855 9.424166289 9.049848549 10.67419227 9.77148947 10.19967234 8.348728154 10.49785184 10.57364719 9.622051819 10.01820018 9.28771238 10.73386272 11.38046107 9.696967526 8.243173983 11.97584797 9.686500527 10.02652344 10.16113188 10.80815977 10.05934446 9.519636253 10.17866485 9.620219826 9.654636029 8.379378367 8.927777962 8.965784285 9.686500527 8.829722735 10.23481743 10.22400167 11.18487534 10.23840474 8.703903573 10.78790256 10.7548875 10.29001885 11.6794801 10.33427329 11.6851868 10.34983409 10.46964182

ATM 9.908392621 11.12153352 13.01000352 9.638435914 11.31741261 11.23840474 11.60408986 11.1221809 10.94251451 12.42416629 11.37666806 11.59105477 11.72792045 11.72152646 11.86495992 11.8917837 11.37232095 11.30549179 9.845490051 12.75258972 10.73047014 11.30947635 13.13683095 12.2039597 10.84470576 9.682994584 11.48331195 9.396604781 10.44604941 12.17710804 10.88798213 11.78053977 9.527477006 12.30833903 12.1424262 10.17741954 10.10328781 12.04200131 11.31458324 10.99717948 11.33650656 11.71724801 10.88493365 10.39553414 12.37585398 10.60733031 11.03823313 10.32979634 11.51914479 11.74146699 11.906139 11.03617361 10.46147945 11.53040634 12.34346334 11.183015 8.717676423 11.78871833 10.58214198 11.62981194 10.82892983 11.60362634 11.215533 11.84117119 11.21613956 10.82654849 11.69696753 10.74399286 11.54399845 11.58402294 11.25266543 10.28886607 11.83683936 12.19813805 12.15228484 11.50432245 12.03926179 12.34374092 11.84392105 11.73893668 12.99647349 12.01122726 13.42088131 12.1376316 11.86418614

ATF2 10.04028972 10.47876962 11.76321237 9.698704667 11.13570929 10.32979634 11.28077077 10.60084211 10.67507492 9.154818109 10.75572215 11.28655776 11.85018684 10.18858885 9.997179481 9.794415866 9.930737338 10.46862404 10.42731284 11.38963134 10.18487534 10.15101654 12.22580994 11.2227949 10.93073734 9.030667136 9.807354922 9.477758266 10.33203655 10.87190524 10.75321675 10.45327063 8.82336724 11.666224 11.41890673 9.691743519 10.40726776 10.57270023 10.36522885 10.94544384 10.86805085 11.30492167 10.2632692 8.980139578 12.06709819 9.703903573 10.84627391 8.044394119 11.05934446 10.92035286 10.25974326 10.73216743 9.45532722 12.08613623 12.25207404 9.392317423 8.982993575 11.1842555 10.63481105 11.15165083 10.62753388 10.32080055 10.80896417 10.68035952 10.41362793 10.36632221 11.16050175 8.784634846 9.315149562 10.50779464 10.64024494 9.611024797 11.41309898 11.47015044 11.83407595 10.07815081 10.90312868 11.74945047 10.53527538 11.93626986 11.63934071 11.66400276 12.40514146 10.68035952 10.59712143

ARNTL 8.330916878 7.741466986 8.797661526 8.703903573 10.60362634 8.73809226 9.25502857 9.769837844 9.019590728 7.475733431 8.558420713 8.581200582 9.321928095 8.159871337 7.312882955 7.971543554 7.614709844 8.77148947 8.906890596 9.64385619 8.891783703 8.791162889 10.64925618 8.475733431 8.087462841 4.247927513 8.562242424 7.74819285 8.764871591 9.698704667 8.550746785 9.216745858 7.845490051 9.535275377 7.813781191 8.049848549 7.95419631 8.810571635 6.672425342 8.622051819 8.700439718 8.285402219 9.172427509 5.584962501 8.129283017 7.62935662 7.257387843 6.832890014 8.710806434 8.744833837 8.721099189 10.08347933 7.912889336 7.857980995 9.381542951 7.392317423 7.366322214 8.276124405 8.361943774 4.807354922 9.100662339 8.710806434 8.108524457 8.672425342 8.238404739 7.46760555 8.857980995 7.426264755 8.438791853 7.238404739 6.918863237 7.936637939 8.426264755 8.527477006 9.310612782 7.50779464 7.055282436 11.11048331 8.044394119 8.271463028 9.955649908 8.396604781 9.776433032 8.73809226 8.562242424

ARHGAP1 12.01924322 12.03789009 13.55710466 12.2868465 12.5020859 11.34651373 12.63481105 14.16702606 12.99612036 11.38801729 12.6188443 13.38167813 11.84156435 11.38154295 10.45943162 12.42914517 11.25856603 13.42324725 12.4046094 12.57412044 12.17554955 12.50556348 13.52625469 11.56985561 11.42311591 10.81217731 11.82336724 10.9432474 12.33539035 13.23376945 12.62798978 12.18673329 11.43983088 13.53065018 12.57127862 11.32755264 11.27844946 13.18967015 11.15924065 11.85408918 11.88607758 12.8390069 11.81578354 9.236014192 11.49235395 12.20518233 11.28598011 10.74062404 12.94489504 13.12557491 11.87267488 14.38269157 11.68299458 12.509775 12.12088584 12.42127591 11.96578428 11.66311331 11.56176526 11.30092449 13.12992693 12.85739789 10.95492329 11.63617144 11.95383268 12.11341661 12.34096276 11.50928017 12.33985 11.17990909 12.30206767 12.0454184 11.81898154 13.14990586 12.86553997 10.857981 11.97477307 14.76906299 12.27204652 13.07497766 13.90679667 12.96722626 13.50394993 12.40806432 12.68759439

AR 3.906890596 6.392317423 8.611024797 7.383704292 8.108524457 7.303780748 10.00140819 7 9.139551352 8.247927513 6.108524457 8.707359132 5.523561956 8.707359132 8.588714636 6.614709844 8.379378367 9.06608919 8.569855608 5.392317423 6.285402219 10.15228484 11.74315139 10.54206454 8.503825738 8.933690655 5.523561956 7.62935662 9.348728154 13.50171281 9.169925001 7.960001932 6.918863237 10.06339508 11.81977993 8.438791853 7.714245518 7.475733431 10.23122118 10.22520744 9.424166289 9.584962501 9.54689446 6.129283017 5.087462841 5.781359714 4.087462841 7.055282436 9.652844973 6.741466986 7.8008999 6.06608919 6.977279923 9.370687407 10.49085088 6.599912842 7.948367232 7.658211483 9.746514321 3.459431619 8.252665432 8.451211112 5.247927513 11.98192399 5.321928095 5.882643049 7.636624621 7.592457037 2.584962501 6.614709844 4.754887502 6.906890596 8.483815777 6.977279923 11.23302043 5.727920455 5.857980995 10.03066714 5.285402219 9.797661526 7.285402219 9.722807531 9.531381461 5.523561956 10.30833903

APTX 9.609178738 9.22881869 10.29347165 9.211888295 10.00842862 8.8008999 9.731319031 9.727920455 10.23481743 7.569855608 9.216745858 9.348728154 10.04848687 8.475733431 8.918863237 9.319672121 8.903881846 9.707359132 10.58308277 10.36303963 9.579315938 10.41468524 10.96361862 8.836050355 9.415741768 10.15608308 9.14974712 9.321928095 10.23601419 10.91587938 9.11113567 10.203348 8.271463028 10.34096276 9.411510988 7.832890014 9.560332834 9.52160044 9.09011242 8.118941073 9.366322214 9.859534786 8.965784285 7.409390936 11.17804233 8.451211112 9.027905997 9.758223215 10.36522885 10.53430288 9.665335917 11.01122726 8.550746785 9.939579214 10.49085088 9.247927513 9.233619677 9.667111542 9.14974712 9.86727874 10.13314221 9.943979914 10.17741954 9.22881869 9.903881846 9.957102042 9.818582177 7.357552005 9.049848549 9.063395081 10.61194694 9.317412614 9.766528909 11.436191 10.76321237 8.581200582 9.342074668 10.84862294 10.75405237 9.612868497 10.67595703 9.796039609 10.34651373 10.57175264 10.02097994

APP 14.31153547 14.480664 15.9684193 14.92207331 15.76766721 15.41452008 15.78199997 16.11746048 15.28792877 15.14425992 15.24900231 15.56134762 15.8180079 14.33462246 13.77035418 15.27029533 15.18920683 16.79291736 14.85135865 15.36074423 15.35614243 16.05214347 17.48337494 14.96929657 14.49391798 13.37598969 16.48895415 13.85496575 15.87718826 17.14855602 15.78379119 16.72310095 13.49735289 16.04852945 16.53019294 11.7477734 15.15378951 16.31373335 14.65329295 15.36830183 15.79667339 14.89111915 14.07246761 13.5315033 15.04486367 14.38397423 15.56955897 13.04848687 16.12583721 16.42927596 16.92360604 16.33671575 15.92918905 15.21405345 17.03752551 15.77001858 14.62382435 14.823417 14.48469704 15.41805024 16.98921708 15.90122046 15.15612259 15.82639952 15.1561621 15.02084976 15.38501977 14.27895756 13.91036166 14.91415152 16.32790345 16.36959735 14.8349154 15.80054606 17.10714554 14.37897215 15.19059634 17.52173845 16.00176003 16.48287097 16.37931067 15.73391567 18.46745431 16.40046255 16.1106872

APOE 13.94150617 15.56949964 14.59304091 11.89254282 12.973518 11.93516505 12.8077574 15.23234597 16.13953137 9.784634846 14.01828713 14.88788697 12.60894781 12.94727165 11.215533 14.43332463 15.78601432 11.98013958 13.76373108 13.94471206 14.45436356 13.05545192 11.52503135 14.27888499 12.71381608 12.6682202 13.06928192 15.03462704 15.08646799 12.09110476 13.4926043 12.52845411 11.6343573 12.93939556 17.80981836 10.46964182 12.98352807 13.72568578 13.50345309 11.86534664 12.6359448 9.791162889 15.66619626 10.40832974 13.34374092 15.0525256 10.027906 12.07714952 13.87517339 14.17757526 13.26751824 13.89073927 12.50804233 13.59304091 16.53521461 12.39473063 14.11683128 13.53138146 11.37721053 12.57317378 13.25192616 15.7704058 11.49884921 12.4003458 13.09786753 15.12137163 16.70746699 13.20273604 10.99435344 14.22505677 12.47065887 11.38478375 12.5667676 16.62339571 13.38302922 12.26121345 15.52374571 10.94178124 13.29174628 8.731319031 13.81738343 13.91045536 15.84894074 13.86399264 15.51896045

APOC3 2 0 1.584962501 0 0 1 1 0 0 0 2.807354922 4.584962501 0 1 3.807354922 2 2.321928095 2.584962501 1 1.584962501 0 1.584962501 1 1.584962501 1.584962501 0 1 0 2 0 1 0 0 2 3 4.754887502 0 1 0 0 2.584962501 1.584962501 0 4.906890596 0 0 0 0 0 0 1.584962501 0 1.584962501 0 2 0 0 0 0 3.700439718 0 2 0 0 1 0 1.584962501 2.584962501 1.584962501 3.169925001 2 0 0 3 0 0 2.807354922 3.906890596 0 0 1 1 1.584962501 0 1

APEX1 13.56200386 12.66222331 12.7986743 11.12799432 13.49822593 11.92999806 11.91026796 14.07121094 13.48934624 11.47522678 12.85506312 12.51840728 14.80670065 11.46913302 11.74104558 12.79055214 12.02479338 12.3818133 13.1179677 12.64069684 12.22881869 14.15180936 13.51101135 11.32361776 12.38694025 12.95619463 12.31061278 11.84549005 13.2973462 13.86408939 13.66921726 12.91587938 11.11958962 13.54375685 13.86021404 10.79116289 11.81698362 12.19383333 12.31203206 11.54012804 13.00615073 12.52160044 11.83289001 10.74399286 12.55314928 12.0768156 11.93700593 11.54592977 12.58777752 13.46722343 12.09077405 13.31415836 11.55937709 13.30121037 13.98832965 12.12476754 13.07196507 12.9432474 12.16081685 11.87113518 12.8957646 12.32474311 14.03565827 12.66378045 11.88455213 13.28381183 13.15908294 10.97727992 12.8474489 12.92907344 13.60848586 12.0344551 12.22249305 14.46505617 14.62342429 11.85330956 12.73914771 15.46106372 12.55482864 12.0385761 15.07773369 12.19537221 13.34374092 13.1341054 13.35397382

AKT1 12.35369821 13.03239017 13.42652685 12.16239133 13.19491072 12.23421868 13.26693291 13.09143539 12.84764464 12.80493767 13.44592013 13.67485431 12.71531855 12.50556348 13.37476781 13.26224169 12.47218312 13.24866884 14.13586958 13.17274002 12.54737656 13.96479209 14.18394547 13.82843404 13.40554038 11.98868469 13.06322653 12.16679075 13.30135329 15.16478911 12.87690059 12.906139 11.61424973 13.5834354 12.77725532 12.12023788 13.04148804 12.89424936 12.62616532 12.18115226 13.58754314 13.22415245 11.72664392 11.08547246 14.45320632 12.47952767 11.70865284 12.26356264 13.55134778 14.05273785 12.91513245 14.01384605 11.70087316 16.07846357 13.74220416 12.57080444 13.14098949 12.29375901 12.66977089 12.39580187 12.94178124 13.29318423 11.77025093 12.71789058 12.95346896 13.24480978 13.15734694 11.87382857 12.77971936 12.62319564 14.23691111 12.53235593 13.38572761 15.02574517 13.74041323 12.64925618 13.5024589 14.43501908 12.61079417 12.85233443 13.95619463 13.32361776 13.67815996 12.48834228 13.52649924

AIFM1 11.07146236 10.12928302 11.33147682 8.921840937 11.45789384 8.939579214 9.897845456 11.41785251 10.4252159 9.64385619 10.23242093 10.25620869 11.40620501 9.396604781 9.481799432 11.13121391 10.44397954 10.59058705 11.0721326 11.30549179 10.60177079 11.08679969 11.44914865 10.49884921 11.25207404 11.05120894 11.32867493 10.51668495 11.8989792 11.17180229 9.501837185 11.80332392 8.73470962 11.28886607 11.42940674 8.071462363 8.487840034 9.503825738 9.936637939 8.558420713 10 10.23959853 10.05528244 7.339850003 10.35974956 10.17990909 9.721099189 9.787902559 10.91064273 12.00527366 9.794415866 11.88531506 9.37721053 10.80251637 12.02686921 9.475733431 10.25148241 10.12670447 10.69435789 8.479780264 10.78790256 10.56890615 11.27844946 9.874981348 10.69087101 10.85486838 10.8153833 8.569855608 10.29920802 10.55170826 11.33203655 10.7984718 9.682994584 11.86495992 11.39231742 9.977279923 10.66799854 11.11829223 10.51865316 10.33650656 11.36084708 10.68299458 11.40673648 11.09011242 10.46760555

AGTR1 3 7.707359132 7.515699838 6.62935662 8.194756854 1.584962501 7.055282436 7.95419631 7.807354922 3.906890596 10.09539702 8.675957033 8.900866808 7.321928095 5 7.321928095 6.714245518 9.611024797 5.044394119 8.918863237 7.330916878 3.459431619 7.686500527 7.257387843 2.584962501 2.584962501 9.487840034 6.426264755 9.276124405 0 9.430452552 8.54689446 6.794415866 8.87958325 8.426264755 6.357552005 7.876516947 9.485829309 2 6.727920455 6.977279923 7.851749041 8.558420713 4.906890596 3.700439718 7.375039431 8.366322214 5.930737338 9.177419538 7.754887502 4.459431619 10.63571812 7.787902559 3.906890596 11.09209641 2 5.614709844 10.93810933 8.326429487 3.584962501 8.531381461 9.689997971 5.087462841 5.614709844 5.491853096 6.554588852 1.584962501 2 10.89254282 2.584962501 3.321928095 6.584962501 8.596189756 8.894817763 8.271463028 4.857980995 5.64385619 6.807354922 4.857980995 3.807354922 7.813781191 7.044394119 7.607330314 5.754887502 7.044394119

AGPAT2 11.43983088 10.33091688 11.49485558 8.159871337 10.53624722 9.719388821 10.43879185 11.53332973 10.54012804 10.18611424 11.29232163 11.11634396 9.964340868 8.22881869 9.147204925 10.30720081 10.50680344 9.631177056 9.35974956 9.350939182 9.519636253 10.10721708 12.09374766 8.330916878 9.517669388 9.519636253 9.695228291 11.11894107 10.01541505 7.813781191 8.724513853 10.87728413 9.35974956 9.661778098 9.144658243 9.5980525 10.14847658 10.10983065 9.991521846 9.836050355 10.42731284 11.67021364 9.457380879 10.36413466 8.985841937 10.24555271 8.962896005 8.495855027 10.8008999 10.41151099 9.946906274 11.7984718 8.885696373 11.86186234 8.651051691 10.59618976 10.92035286 8.758223215 8.668884984 7.383704292 9.859534786 11.6329952 10.30263892 6.569855608 10.00702727 9.172427509 11.01402047 9.030667136 10.18239435 9.339850003 11.58590145 9.881113961 8.87958325 11.19290922 10.60733031 8.741466986 10.92109709 11.72962074 12.10000522 11.42992974 12.81117397 10.33427329 11.55794229 10.14847658 10.04712391

ADCY5 4.459431619 3.169925001 6.14974712 3.807354922 7.14974712 2.584962501 7.459431619 4.321928095 6.686500527 4.700439718 4.523561956 4.247927513 9.586839788 5.247927513 6.50779464 5 5 5.169925001 4.087462841 3.906890596 5.832890014 5.64385619 6.62935662 5.129283017 4.523561956 3.700439718 6.820178962 2.321928095 5.584962501 5.247927513 5.882643049 5.087462841 5.044394119 5.64385619 4 5.906890596 5.357552005 7.864186145 4.392317423 9.821773982 3.321928095 6.894817763 6.044394119 5.392317423 5.285402219 3.459431619 3 5.247927513 3.906890596 5.169925001 5.247927513 4.247927513 1.584962501 5.523561956 9.124121312 8.271463028 7.022367813 4.247927513 3.906890596 3.321928095 5.672425342 5.754887502 4.321928095 3 4.64385619 5.129283017 5.700439718 4.64385619 4.247927513 4.392317423 4.169925001 5.129283017 4.807354922 1.584962501 8.174925683 3.807354922 4.247927513 5.285402219 5.491853096 8.060695932 10.53527538 8.262094845 8.614709844 5.930737338 7.303780748

ABL1 11.61792656 12.1424262 13.22505677 12.29318423 11.66133275 12.61493985 12.21765483 12.71660514 12.84646981 11.42940674 13.44268437 13.05494342 13.95592229 10.17866485 11.15860969 11.48582931 11.8419574 13.06777046 11.85447883 12.41574177 12.32023645 12.95601308 13.47027756 11.26150731 11.39713981 10.84156435 13.25192616 10.7548875 12.0313566 13.15608308 13.20304206 13.14354265 11.18549492 12.73470962 11.71080643 12.09967655 10.48179943 13.07196507 10.63026713 12.24911345 13.18951573 12.82436213 11.72494012 10.56795608 11.91923579 12.03720374 12.65396464 10.04028972 12.75759836 12.79745889 11.7368247 13.35590164 11.77930897 12.52356196 11.82495874 12.82217246 11.65687173 11.1382718 11.24733418 11.08746284 11.74483384 12.68584381 11.46199095 12.13217838 10.97010589 11.69174352 12.24911345 11.39874369 11.84509796 11.04371086 12.58730873 11.19475685 11.17242751 12.52405192 12.11958962 11.02583167 11.40886044 13.89206842 12.50133962 12.74672424 13.38235383 10.90312868 13.98103206 13.44798721 12.77272695

A2M 13.60952505 15.38939607 16.84933179 15.09465504 18.21638116 15.37347693 14.12734954 13.87805091 15.91517914 13.95546829 14.53557915 16.16551551 15.2708428 15.46409899 12.16804527 15.56667842 14.41084881 13.77014767 16.012319 16.88494557 15.91786154 15.45763738 16.74823217 16.73031091 14.9685318 13.52454172 13.12896095 13.10476254 16.43216669 14.07998471 15.25565563 16.94708898 13.1744576 17.22291751 15.27175481 13.55290921 14.35300895 14.19129059 15.03424014 15.98821868 15.89522024 14.46243837 16.78290865 11.28828934 16.20926296 14.18874337 11.95383268 15.60385812 14.7160692 16.34360214 15.24332277 13.69555456 14.96185662 15.10693093 16.34089324 13.01122726 14.5822008 13.90717234 14.30961846 14.57919807 18.60113239 13.89671082 17.16916166 15.56964797 15.9575782 14.62924277 15.43182403 11.90726625 12.00527366 16.4416636 15.23631323 14.46122363 13.39593572 16.84912406 15.8778353 16.71782366 14.83234613 15.31938988 16.04526907 13.70455212 17.10788113 15.45648276 17.15061005 14.4010795 15.23219605

**Supplement Table 2. 91 prognosis-related genes using TARGET expression profile data**

gene HR HR.95L HR.95H pvalue

1 CETP 0.740021029 0.584459467 0.936987345 0.012401292

2 ESR1 0.71118685 0.529790189 0.954692531 0.023293399

3 TERT 1.304765892 1.13234464 1.503441596 0.000234409

4 FOXM1 0.647554792 0.432355364 0.969867022 0.034995098

5 JUN 0.699250571 0.504121122 0.969908499 0.032114125

6 LMNA 0.614858789 0.4191675 0.901909931 0.012842069

7 CAT 0.526520407 0.368123227 0.753073205 0.000442877

8 MAPK3 0.62187956 0.400150004 0.966473029 0.034724453

9 SERPINE1 0.755877535 0.57838368 0.987840543 0.040407925

10 PARP1 0.58017245 0.382447047 0.88012203 0.010451261

11 AGTR1 0.803891925 0.697920435 0.925954012 0.002473092

12 BAX 0.671872701 0.469631585 0.961206486 0.02951747

13 CTF1 0.807466938 0.662063777 0.984803699 0.034761013

14 STAT3 0.634399759 0.416261133 0.966852348 0.034280063

15 NCOR2 0.530018922 0.338976473 0.828730249 0.005374095

16 KCNA3 0.69225555 0.528039925 0.907540745 0.007763541

17 GRN 0.639465527 0.48028804 0.851397757 0.002202314

18 RAE1 0.623171886 0.393893917 0.98590809 0.043321216

19 GSK3A 0.569796543 0.35487742 0.914873932 0.019899946

20 APEX1 0.641498753 0.42249308 0.974029325 0.037209733

21 POLA1 0.635143955 0.416752555 0.967979295 0.034742577

22 PDGFB 0.683722232 0.483106395 0.967646246 0.031908

23 UBE2I 0.54044996 0.340363217 0.858160179 0.009098071

24 XRCC6 0.617103378 0.396964309 0.95932196 0.031996664

25 FAS 0.656533401 0.515696241 0.83583333 0.000636406

26 PDGFRB 0.696312852 0.509175354 0.952229099 0.023422408

27 BAK1 0.545265777 0.355315763 0.836762111 0.005510536

28 HTT 0.575550904 0.376776728 0.879191358 0.010600875

29 MAP3K5 0.551994672 0.395323191 0.770757003 0.000485406

30 SIRT6 0.495875963 0.319769777 0.768968766 0.001726904

31 C1QA 0.771435929 0.629680189 0.945104202 0.01224603

32 IGF2 0.847286272 0.723294804 0.99253309 0.040091296

33 ERCC2 0.496611493 0.307962513 0.800821413 0.004091169

34 PLCG2 0.641194974 0.470766309 0.873322893 0.004814482

35 PML 0.457892419 0.302503667 0.693100583 0.000221478

36 LEPR 0.808003639 0.653207834 0.999482625 0.049445113

37 SQSTM1 0.613771494 0.40872637 0.921681287 0.018616834

38 GRB2 0.588050269 0.367006703 0.942225623 0.027287735

39 NFKB1 0.578838842 0.410997014 0.815223453 0.001752552

40 HDAC1 0.602681911 0.379901324 0.956104816 0.031507099

41 SIRT3 0.571696377 0.373796794 0.874370119 0.009901769

42 PIK3R1 0.664283675 0.471755837 0.935383871 0.019155013

43 ABL1 0.52066803 0.329578382 0.822551517 0.005154734

44 ERCC8 0.558425971 0.356224337 0.875402191 0.011081423

45 EGFR 0.740678822 0.617934578 0.88780453 0.001165109

46 ARHGAP1 0.49220529 0.3346744 0.723885806 0.000316057

47 ZMPSTE24 0.614786675 0.405003948 0.933232029 0.022344705

48 PDPK1 0.629036942 0.412469755 0.959312698 0.031328563

49 PTPN1 0.492668344 0.315964773 0.768193537 0.001786865

50 UCP2 0.748723243 0.58037211 0.965908742 0.025955384

51 CEBPA 0.675791432 0.527521725 0.865735074 0.001929942

52 STK11 0.559551874 0.338232173 0.925690471 0.023784277

53 MLH1 0.589633573 0.395419817 0.879237042 0.009561656

54 TP53BP1 0.635505262 0.41972868 0.962209536 0.032194826

55 GCLM 0.587552021 0.380657624 0.906897314 0.016339893

56 ERCC4 0.491398161 0.33400212 0.722965929 0.000310171

57 IL7 0.694673512 0.559553431 0.862422178 0.000963013

58 AIFM1 0.659170408 0.469155718 0.926143731 0.016296967

59 GSS 0.623271497 0.429597438 0.904259022 0.012773785

60 PTPN11 0.565769369 0.369487638 0.866321213 0.008790988

61 HESX1 0.709850365 0.545977463 0.922909048 0.010496864

62 PPARG 0.722532748 0.605850779 0.861686722 0.000298593

63 EPS8 0.493739303 0.359730105 0.677670554 1.25E-05

64 HDAC3 0.522716079 0.310966411 0.8786547 0.014358706

65 CNR1 0.846927583 0.727302763 0.986227973 0.032479286

66 HIF1A 0.64520553 0.457986531 0.908957246 0.012215575

67 MTOR 0.601373947 0.370159587 0.977012716 0.039986441

68 NBN 0.673599455 0.48917506 0.92755388 0.015490464

69 BCL2 0.731178325 0.570497377 0.937115165 0.013400135

70 TERF2 0.494747879 0.307891948 0.795004435 0.00363803

71 TAF1 0.549622779 0.356635381 0.847042147 0.006683468

72 GTF2H2 0.609184489 0.460379058 0.806087364 0.00052338

73 FOXO4 0.624420244 0.416141818 0.936941744 0.022932582

74 GPX1 0.695959766 0.523604098 0.925050048 0.012539928

75 CREBBP 0.635278204 0.403682925 0.999741063 0.049869297

76 SIN3A 0.517006494 0.30382233 0.879776396 0.015007388

77 STAT5B 0.470116293 0.310632442 0.711481801 0.000356875

78 ARNTL 0.481970583 0.342384871 0.678463515 2.87E-05

79 TP63 0.710213169 0.546899701 0.922294791 0.010266883

80 NFE2L1 0.574021963 0.364258767 0.904580052 0.016750557

81 GPX4 0.581916122 0.39749394 0.851903234 0.005366305

82 COQ7 0.509608604 0.314213783 0.826510305 0.006290208

83 MAPK14 0.576563209 0.379189764 0.876672224 0.010007088

84 APOE 0.760654045 0.608360531 0.951071852 0.01639226

85 MAX 0.604828655 0.38631045 0.946952645 0.027930401

86 CDC42 0.54164999 0.354530358 0.827530578 0.004576608

87 PPM1D 0.523437003 0.3312212 0.827200362 0.005563482

88 PSEN1 0.508594713 0.322024162 0.803258305 0.003737701

89 STAT5A 0.510543636 0.354292518 0.735705077 0.000310366

90 PEX5 0.676068305 0.466389389 0.980014476 0.038777823

91 IL2RG 0.724145627 0.57627624 0.909957504 0.005611624

**Supplement Table 3. 34 aging-related genes with a significant difference**

Gene P.val

PIK3R1 0.035027015

TERT 0.004895423

GPX4 0.031858698

PPARG 0.032872776

PTPN11 0.025265512

ERCC4 0.002493314

PML 5.85E-05

BAX 0.021807484

AIFM1 0.037751592

LEPR 0.013230351

GPX1 0.034493523

CAT 0.028228176

FOXO4 0.030240199

MAPK14 0.006057505

CDC42 0.019901878

CEBPA 0.023054999

AGTR1 0.021931833

BCL2 0.006354429

SQSTM1 0.005964086

APOE 0.00558209

FAS 0.036849714

GRN 0.010239014

ZMPSTE24 0.046465473

EPS8 0.037755432

C1QA 0.028386567

CNR1 0.035811664

UCP2 0.017561528

HIF1A 0.014603595

SIRT6 0.009675473

GTF2H2 0.009212164

STAT5A 0.000345784

PARP1 0.017221276

TERF2 0.035127976

PDGFRB 0.038909192
